# Supplementary material for: Community health worker–facilitated telehealth for moderate–severe hypertension care in Kenya and Uganda: A randomized controlled trial
Source: PLoS Med. 2025 Jun 5;22(6):e1004632. doi: 10.1371/journal.pmed.1004632 (PMC12165344; doi:10.1371/journal.pmed.1004632)
Supplement: S1 File — (PDF) [file pmed.1004632.s001.pdf]

Supplemental Materials

Supplemental Tables 2

Table A. Implementation strategies employed for Telehealth and Clinic-based hypertension care 2

Table B. Characteristics of persons with severe hypertension identified on community screening stratified by linkage vs non-linkage to the community primary care clinic 4

Table C. Results and interpretation of sensitivity analyses varying the approach to covariate adjustment 6

Table D. Results and interpretation of sensitivity analyses varying the approach to missing data 8

Table E. Number of medications prescribed as of week 24 among those with controlled blood pressure at week 24, stratified by baseline hypertension severity and trial arm 9

Table F. Number and type of hypertension medications prescribed over 48 weeks* 10

Table G. Medication combinations prescribed at last attended visit 11

Table H. Hypertension medication adherence by participant 12

Table I. Hypertension medication adherence across all post-baseline visits 12

Table J. Adverse events 13

Table K. Hypertension Severity 14

Supplemental Figures 15

Figure A. Mean SBP across study visits 15

Figure B: Hypertension control at 24 weeks by presence of co-morbidities 16

Figure C: Hypertension control at 48 weeks by presence of co-morbidities 17

Figure D. Time to hypertension control 18

Figure E. Facilitators of hypertension care engagement 19

Figure F. Barriers to hypertension care engagement 20

References 21

Supplemental Tables

Table A. Implementation strategies employed for Telehealth and Clinic-based hypertension care

Strategy*	Description	Telehealth arm	Clinic arm
Provide community-based services	CHW home visits for BP measurement, and delivery of hypertension medications based on clinician prescription; visits every 4-12 weeks	X	
Provide telemedicine consultation; Use technology to provide real-time support; Alter/improve communication between providers	During each home visit, CHWs facilitate telemedicine consultation with clinician based in primary health center	X	
Provide health education	CHW counseling on lifestyle modification and hypertension medication adherence at each visit	X	
Change payment methods for health workers	Stipend payment to CHWs to compensate for work delivering hypertension intervention†	X	
Provide community-based services	CHW home-based hypertension screening at baseline	X	X
Provide transportation	One-time transport reimbursement to patients to facilitate linkage from community screening to primary care clinic for initial clinic visit	X	X
Adjust patient/consumer fees	Hypertension medications provided free of charge to patients at each visit	X	X
Change physical structure and equipment	BP cuff provision in clinics and for CHWs one time at study baseline	X	X
Conduct ongoing training	Training of CHWs and clinicians on hypertension at study baseline and 6-month refresher training	X	X
Provide training on patient-centered communication	Clinician and CHW training on patient-centeredness, friendly care at baseline	X	X
Increase the quantity of medication dispensed	Dispensation of 3-month medication supply at visits when BP controlled	X	X
Institute or improve procurement and storage systems	Enhancement of hypertension medication supply chain to avoid stock-outs throughout study	X	X
Introduce/alter health information systems	Use of electronic health record for hypertension visits, allowing review of longitudinal clinical data by clinicians at each visit	X	X
Provide guidance document	Standardized country-guideline based hypertension treatment algorithm to	X	X

	simplify treatment decisions for clinicians		
Provide multiple types of services by one provider	Provision of both hypertension and diabetes care during same visit	X	X
Provide multiple types of services by one provider	Provision of both hypertension and HIV care during same visit		X

* Implementation strategies used for the two trial arms, based on the Expert Recommendations for Implementing Change (ERIC)[1] and Effective Practice and Organization of Care (EPOC)[2] implementation strategy compilations, as further refined in a recent review by Lujintanon et al.[3]
† Approximately $40 US dollars per month in 2022-2023. Stipend amount is same as that adopted by the Kenya Ministry of Health for Community Health Promoters beginning January 2024 (after completion of trial).
Abbreviations: Community Health Worker, CHW; Blood Pressure, BP; Human immunodeficiency virus, HIV.

Table B. Characteristics of persons with severe hypertension identified on community screening stratified by linkage vs non-linkage to the community primary care clinic

	Not linked (N=52)	Linked (N=214)
**Age (years)**		
Mean (SD)	56 (15)	62 (13)
Median (Q1, Q3)	51 (43, 67)	62 (51, 72)
**Sex**		
Female	35 (67.3%)	149 (69.6%)
Male	17 (32.7%)	65 (30.4%)
**Blood Pressure Ever Measured**		
Yes	23 (44.2%)	138 (64.5%)
No	29 (55.8%)	76 (35.5%)
**Hypertension History**		
Yes	13 (25.0%)	93 (43.5%)
No	39 (75.0%)	121 (56.5%)
**Hypertension Meds (Last 30 Days)**		
Yes	9 (17.3%)	43 (20.1%)
No	4 (7.7%)	50 (23.4%)
Missing	39 (75.0%)	121 (56.5%)
**Diabetes Diagnosis**		
Yes	5 (9.6%)	21 (9.8%)
No	47 (90.4%)	193 (90.2%)
**HIV Status***		
Yes	2 (3.8%)	22 (10.3%)
No	21 (40.4%)	102 (47.7%)
Missing	29 (55.8%)	90 (42.1%)
**Average Systolic BP (mmHg)**		
Mean (SD)	160 (19)	166 (17)
Median (Q1, Q3)	161 (146, 166)	164 (158, 174)
**Average Diastolic BP (mmHg)**		
Mean (SD)	103 (12)	100 (11)
Median (Q1, Q3)	102 (96, 108)	100 (92, 107)
**Hypertension Stage**		
BP <140/90 mmHg	0 (0%)	0 (0%)
BP 140-159/90-99 mmHg	0 (0%)	2 (0.9%)†
BP 160-179/100-109 mmHg	35 (67.3%)	150 (70.1%)
BP ≥180/110 mmHg	17 (32.7%)	62 (29.0%)

* Self-reported HIV status to community health worker. HIV testing was conducted after linkage to the clinic and is not depicted in this table.

† Two participants had mild hypertension at community-based screening but self-presented to the clinic and had blood pressure ≥160/100 mmHg on repeat measurement at the clinic and were therefore eligible for the study.

Abbreviations: Standard Deviation, SD; First Quartile, Q1; Third Quartile, Q3; Human immunodeficiency virus, HIV; Blood Pressure, BP.

Table C. Results and interpretation of sensitivity analyses varying the approach to covariate adjustment

	INTERVENTION	CONTROL	EFFECT
**HTN control at 24 weeks**			
Primary analysis	77% (69,85%)	51% (42,60%)	26% (14,38%); p<0.001
Unadjusted	77% (68,85%)	52% (42,62%)	25% (12,37%); p<0.001
Adjustment for HTN grade	77% (69,85%)	52% (42,61%)	25% (12,38%); p<0.001
Adjustment for SBP	76% (68,85%)	52% (42,62%)	24% (11,37%); p<0.001
**HTN control at 48 weeks**			
Primary analysis	86% (79,92%)	44% (34,54%)	42% (30,53%); p<0.001
Unadjusted	86% (79,93%)	44% (34,54%)	42% (30,53%); p<0.001
Adjustment for HTN grade	86% (79,92%)	44% (34,54%)	42% (30,53%); p<0.001
Adjustment for SBP	86% (79,93%)	45% (35,54%)	41% (29,53%); p<0.001

Abbreviations: Hypertension, HTN; Systolic blood pressure, SBP.

The primary analysis used TMLE with Adaptive Pre-specification to select the adjustment approach maximizing precision, while protecting Type-I error and returning estimates of the marginal effect. (See below for brief overview of TMLE).[4] For hypertension control at 24 weeks (primary outcome), the approach selected adjustment for all candidates (age, sex, baseline hypertension severity, and country) and was, as expected, more precise than alternative approaches. Specifically, the relative variance of the primary analysis to the unadjusted effect estimator was 89.6%; this would translate into an 11.4% savings in sample size.[4–6] Also as expected, the results were similar when using adaptive adjustment (primary analysis) and when using fixed adjustment for baseline hypertension grade and for baseline systolic blood pressure (SBP).

Results were also robust to the analytic approach for hypertension control at 48 weeks (secondary outcome). For this secondary endpoint, the primary analytic approach (TMLE with Adaptive Pre-specification) selected adjustment for baseline hypertension grade. Therefore, the results for the primary approach are essentially equivalent to the sensitivity analysis with forced adjustment for baseline hypertension grade.

Here, we provide a brief overview of TMLE. Let W denote the baseline covariates, A be an indicator of being in the intervention arm, and Y be the outcome. With the following steps, TMLE returns estimates of the marginal effect on the absolute scale:

$$\mathbb{E}[\mathbb{E}(Y|A=1,W)] - \mathbb{E}[\mathbb{E}(Y|A=0,W)]$$

1. Estimate the expected outcome given the trial arm and covariates (a.k.a., the outcome regression): $\mathbb{E}(Y|A,W)$
2. Estimate the known propensity score: $\mathbb{P}(A=1|W)$
3. Target initial estimates of the outcome regression using information in the propensity score: $\widehat{\mathbb{E}}^*(Y|A,W)$
4. Obtain targeted predictions of the expected outcome under the intervention and control for all units: $\widehat{\mathbb{E}}^*(Y|A=1,W_i)$ and $\widehat{\mathbb{E}}^*(Y|A=0,W_i)$ for $i=1,\ldots,N$.
5. Obtain a point estimate by averaging the targeted predictions and contrasting on the scale of interest:

$$\frac{1}{N}\sum_{i=1}^{N}\widehat{\mathbb{E}}^{*}(Y|A=1,W_i)-\frac{1}{N}\sum_{i=1}^{N}\widehat{\mathbb{E}}^{*}(Y|A=0,W_i)$$

6. Obtain inference with the estimated influence curve (function).

For binary or bounded continuous outcomes, the logistic link function is recommended for the outcome regression and was used in all analyses. Importantly, TMLE is robust to specification of the working models for the outcome regression or propensity score.

In the primary analysis, we implemented TMLE with Adaptive Pre-specification to data-adaptively select the combination of estimators of the outcome regression and propensity score that maximize empirical efficiency, without sacrificing Type-I error control. Further details on the methodology are available in Balzer et al. *Biometrics* 2024.[4] Further details for this project are provided in the Statistical Analysis Plan.

The selected adjustment approach for each endpoint were as follows.

	**Outcome regression**	**Propensity score**
HTN control at 24 weeks	Main terms*	Age
HTN control at 48 weeks	HTN severity	HTN severity
Moderate-severe HTN at 24 weeks	NA**	NA**
Moderate-severe HTN at 48 weeks	NA**	NA**
Retention in care at 24 weeks	Country	HTN severity
Retention in care at 48 weeks	Sex	Unadjusted
Mean SBP at 24 weeks	Main terms*	Age
Mean SBP at 48 weeks	Main terms*	Unadjusted
Time in care	Country	Unadjusted

Abbreviations: Hypertension, HTN; Systolic blood pressure, SBP.
*Adjusting for all candidates (age, sex, baseline HTN severity, and country) with main terms logistic regression.
**As specified in the Statistical Analysis Plan, we did not use TMLE with Adaptive Pre-specification for the proportion with moderate-severe HTN. Instead, we used an alternative TMLE to flexibly adjust for missing data.

Table D. Results and interpretation of sensitivity analyses varying the approach to missing data

	INTERVENTION	CONTROL	EFFECT
**HTN control at 24 weeks**			
Primary analysis	77% (69,85%)	51% (42,60%)	26% (14,38%); p<0.001
Exclude missing	78% (70,86%)	54% (44,63%)	25% (12,37%); p<0.001
TMLE for missing data	78% (70,86%)	53% (43,62%)	26% (13,38%); p<0.001
**HTN control at 48 weeks**			
Primary analysis	86% (79,92%)	44% (34,54%)	42% (30,53%); p<0.001
Exclude missing	90% (84,96%)	49% (39,59%)	41% (30,53%); p<0.001
TMLE for missing data	90% (84,96%)	49% (39,59%)	41% (30,53%); p<0.001

Abbreviations: Hypertension, HTN; Targeted maximum likelihood estimation, TMLE.

The primary analysis conservatively assumed that participants without blood pressure measures were uncontrolled. We conducted two sensitivity analyses to examine the robustness of our missing data approach: (1) exclude participants with missing outcomes, and (2) use TMLE to flexibly adjust for participants with and without blood pressure measures. As shown in the above Table, these sensitivity analyses yielded very similar results for effectiveness on hypertension control at week 24 (primary endpoint) and at week 48 (secondary endpoint).

Table E. Number of medications prescribed as of week 24 among those with controlled blood pressure at week 24, stratified by baseline hypertension severity and trial arm

	Telehealth intervention arm			Clinic-based arm		
**Hypertension severity at study enrollment**	**BP 140-159/90-99 (N=40)**	**BP 160-179/100-109 (N=19)**	**BP ≥180/110 (N=16)**	**BP 140-159/90-99 (N=20)**	**BP 160-179/100-109 (N=22)**	**BP ≥180/110 (N=11)**
**Number of medications**						
Mean (SD)	1.30 (0.72)	1.68 (0.58)	2.06 (0.44)	1.40 (0.68)	1.91 (0.61)	2.09 (0.70)
Median [Min, Max]	1 [0, 3]	2 [1, 3]	2 [1, 3]	1 [0, 2]	2 [1, 3]	2 [1, 3]
**Number of medications**						
0	5 (12.5%)	0 (0%)	0 (0%)	2 (10.0%)	0 (0%)	0 (0%)
1	19 (47.5%)	7 (36.8%)	1 (6.3%)	8 (40.0%)	5 (22.7%)	2 (18.2%)
2	15 (37.5%)	11 (57.9%)	13 (81.3%)	10 (50.0%)	14 (63.6%)	6 (54.5%)
3	1 (2.5%)	1 (5.3%)	2 (12.5%)	0 (0%)	3 (13.6%)	3 (27.3%)
**Calcium channel blocker**	28 (70.0%)	16 (84.2%)	14 (87.5%)	14 (70.0%)	21 (95.5%)	11 (100%)
**Thiazide diuretic**	7 (17.5%)	5 (26.3%)	7 (43.8%)	4 (20.0%)	5 (22.7%)	2 (18.2%)
**ACE/ARB**	4 (10.0%)	3 (15.8%)	5 (31.3%)	4 (20.0%)	3 (13.6%)	4 (36.4%)

Abbreviations: Blood Pressure, BP; Standard Deviation, SD; Angiotensin-converting enzyme inhibitors, ACE; Angiotensin receptor blockers, ARB.

This table depicts the number and type of medications required to achieve hypertension control at the 24-week study visit.

Table F. Number and type of hypertension medications prescribed over 48 weeks*

	Intervention (N=98)	Control (N=102)	Overall (N=200)
**Number of medications***			
Mean (SD)	1.6 (0.8)	1.7 (0.7)	1.6 (0.7)
Median (Q1, Q3)	2 (1, 2)	2 (1, 2)	2 (1, 2)
**Number of medications**			
0	9 (9.2%)	7 (6.9%)	16 (8.0%)
1	30 (30.6%)	25 (24.5%)	55 (27.5%)
2	51 (52.0%)	63 (61.8%)	114 (57.0%)
3	8 (8.2%)	7 (6.9%)	15 (7.5%)
**Calcium channel blocker†**	73 (74.5%)	79 (77.5%)	152 (76.0%)
**Thiazide diuretic†**	30 (30.6%)	31 (30.4%)	61 (30.5%)
**ACE/ARB†**	18 (18.4%)	17 (16.7%)	35 (17.5%)

* maximum number of drugs prescribed at any clinical hypertension visit (either telehealth or in-clinic)
† proportion prescribed each drug class at any given visit over 48-week study
Abbreviations: Standard Deviation, SD; First Quartile, Q1; Third Quartile, Q3; Angiotensin-converting enzyme inhibitors, ACE; Angiotensin receptor blockers, ARB.

Table G. Medication combinations prescribed at last attended visit

Study Arm	Hypertension medications (total daily dose in milligrams)*	n	%
Control	Lifestyle counseling (no medications)	13	12.7%
	Nifedipine: 40	7	6.9%
	Nifedipine: 20	6	5.9%
	Bendroflumethiazide: 5	3	2.9%
	Hydrochlorothiazide: 25	3	2.9%
	Nifedipine: 40; Hydrochlorothiazide: 25	25	24.5%
	Nifedipine: 20; Bendroflumethiazide: 5	12	11.8%
	Nifedipine: 40; Bendroflumethiazide: 5	12	11.8%
	Amlodipine: 10; Losartan: 50	3	2.9%
	Amlodipine: 5; Losartan: 50	2	2.0%
	Bendroflumethiazide: 5; Captopril: 50	2	2.0%
	Nifedipine: 80; Hydrochlorothiazide: 25	2	2.0%
	Bendroflumethiazide: 10; Captopril: 50	1	1.0%
	Bendroflumethiazide: 5; Captopril: 25	1	1.0%
	Nifedipine: 40; Bendroflumethiazide: 10	1	1.0%
	Nifedipine: 40; Captopril: 50	1	1.0%
	Nifedipine: 80; Hydrochlorothiazide: 50	1	1.0%
	Nifedipine: 40; Bendroflumethiazide: 5; Captopril: 25	3	2.9%
	Nifedipine: 40; Bendroflumethiazide: 5; Captopril: 50	2	2.0%
	Amlodipine: 5; Bendroflumethiazide: 5; Losartan: 50	1	1.0%
	Nifedipine: 20; Bendroflumethiazide: 5; Captopril: 25	1	1.0%
Intervention	Lifestyle counseling (no medications)	10	10.2%
	Nifedipine: 40	14	14.3%
	Nifedipine: 20	7	7.1%
	Bendroflumethiazide: 5	4	4.1%
	Hydrochlorothiazide: 25	4	4.1%
	Nifedipine: 40; Hydrochlorothiazide: 25	23	23.5%
	Nifedipine: 20; Bendroflumethiazide: 5	14	14.3%
	Bendroflumethiazide: 5; Captopril: 25	7	7.1%
	Nifedipine: 20; Captopril: 25	2	2.0%
	Amlodipine: 10; Hydrochlorothiazide: 80	1	1.0%
	Amlodipine: 5; Losartan: 50	1	1.0%
	Nifedipine: 20; Losartan: 50	1	1.0%
	Nifedipine: 40; Bendroflumethiazide: 5	1	1.0%
	Nifedipine: 40; Hydrochlorothiazide: 60	1	1.0%
	Nifedipine: 40; Hydrochlorothiazide: 90	1	1.0%
	Nifedipine: 20; Bendroflumethiazide: 5; Captopril: 25	7	7.1%

* prescribed at last attended clinical hypertension visit (either telehealth or in-clinic) prior to week 48 study visit

Table H. Hypertension medication adherence by participant

Within-participant mean self-reported adherence across all visits, among participants with at least one post-baseline visit and prescribed ≥1 hypertension medication. Adherence assessed at each visit by asking the number of days hypertension medications were taken over the prior 7 days. In the telehealth arm, adherence was assessed by CHWs. In the clinic-based control arm, adherence was assessed by clinicians.

	Intervention (N=92)	Control (N=85)	Overall (N=177)
**Number of days meds taken over past week**			
Mean (SD)	5.7 (1.7)	5.9 (1.9)	5.8 (1.8)
Median (Q1, Q3)	6 (5, 7)	7 (6, 7)	7 (5, 7)
Missing adherence assessment for all visits	2 (2.2%)	30 (35.3%)	32 (18.1%)

Abbreviations: Standard Deviation, SD; First Quartile, Q1, Third Quartile, Q3.

Table I. Hypertension medication adherence across all post-baseline visits

Across all post-baseline visits where participant had previously been prescribed ≥1 hypertension medication. Adherence assessed at each visit by asking the number of days hypertension medications were taken over the prior 7 days.

	Intervention (N=417)	Control (N=322)	Overall (N=739)
**Number of days meds taken over past week**			
Mean (SD)	5.6 (2.2)	6.1 (1.9)	5.8 (2.1)
Median [Min, Max]	7 (4, 7)	7 (6, 7)	7 (5, 7)
Missing adherence assessment at current visit	128 (30.7%)	141 (43.8%)	269 (36.4%)

Abbreviations: Standard Deviation, SD.

Table J. Adverse events

				Baseline					Blood pressure at last follow-up visit		SAE	SAE Description
Study arm	Country	Age	Sex	SBP	DBP	HTN emergency	Creatinine (mg/dl)	GFR	SBP	DBP		
Intervention	Kenya	70	F	194	118	True	0.86	61	129	88	hospitalization	Hospitalized for stroke (symptoms: right-sided hemiparesis; likely ischemic based on non-contrast CT).
Intervention	Kenya	73	M	178	100	False	11.47	4	143	79	hospitalization, death	Patient with GFR 4 ml/min at study enrollment was hospitalized for symptoms of volume overload. No dialysis available and died during hospitalization.
Intervention	Kenya	70	M	165	89	False	1.18	77	126	72	hospitalization	Hospitalization for inguinal hernia repair.
Intervention	Kenya	72	F	202	111	True	0.61	60	137	85	death	Death due to presumed congestive heart failure. Developed difficult breathing and chest pain between home visits, presented to hospital where records showed volume overload and pulmonary edema. Died the day following admission.
Control	Uganda	89	F	162	85	False	0.96	57	105	66	death	Elderly female (89 years) who died at home after brief illness

Abbreviations: Systolic Blood Pressure, SBP; Diastolic Blood Pressure, DBP; Glomerular Filtration Rate, GFR; Computed Tomography, CT.

Table K. Hypertension Severity

Hypertension Stage	Intervention n (%)	Control n (%)
Enrollment		
BP 140-159/90-99 mmHg	49/98 (50%)	38/102 (37%)
BP 160-179/100-109 mmHg	23/98 (23%)	40/102 (39%)
BP ≥180/110 mmHg	26/98 (27%)	24/102 (24%)
Week 24		
BP <140/90 mmHg	75/98 (77%)	53/102 (52%)
BP 140-159/90-99 mmHg	14/98 (14%)	22/102 (22%)
BP 160-179/100-109 mmHg	4/98 (4%)	14/102 (14%)
BP ≥180/110 mmHg	3/98 (3%)	10/102 (10%)
Missing	2/98 (2%)	3/102 (3%)
Week 48		
BP <140/90 mmHg	84/98 (86%)	45/102 (44%)
BP 140-159/90-99 mmHg	7/98 (7%)	33/102 (32%)
BP 160-179/100-109 mmHg	1/98 (1%)	11/102 (11%)
BP ≥180/110 mmHg	1/98 (1%)	3/102 (3%)
Missing	5/98 (5%)	10/102 (10%)

This table provides underlying data displayed in Figure 2. Week 24 and 48 blood pressure measurements taken by research assistants not involved in delivering telehealth or clinic-based hypertension care. Abbreviations: Blood pressure, BP. Millimeters of mercury, mmHg.

Supplemental Figures

Figure A. Mean SBP across study visits

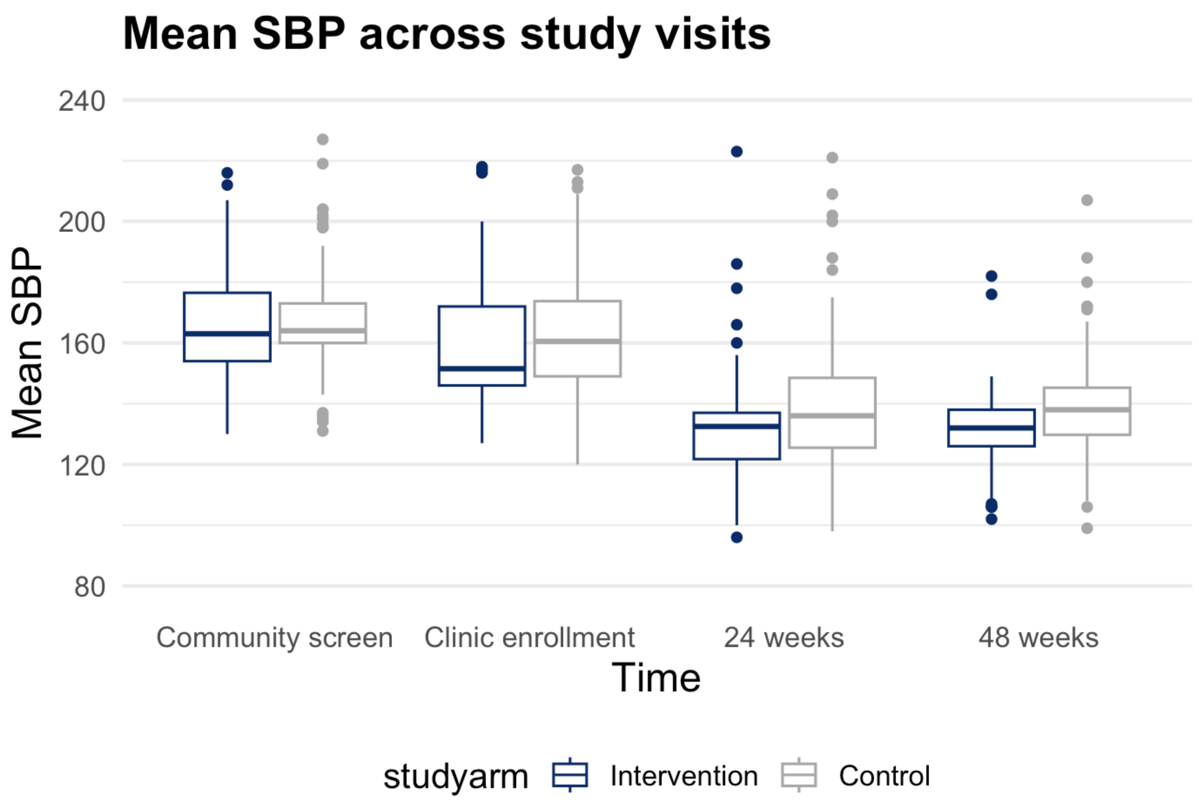

Distribution of average blood pressure by participant at each time point. For each participant, mean SBP indicates the average of 2nd and 3rd systolic blood pressure measurement. Abbreviations: Systolic blood pressure, SBP.

Figure B: Hypertension control at 24 weeks by presence of co-morbidities

Hypertension control at week 24, by comorbidity status

	N	Intervention	Control	Risk difference (95% CI)	p–value
**All**	200	77% (69,85)	51% (42,60)	0.26 (0.14,0.38)	<0.01
**HIV**					
With HIV	27	58% (28,88)	47% (20,74)	0.12 (–0.29,0.52)	0.56
Without HIV	147	79% (70,88)	51% (40,61)	0.29 (0.15,0.42)	<0.01
**Diabetes (DM)**					
With DM	15	75% (41,109)	57% (15,99)	0.18 (–0.36,0.72)	0.49
Without DM	185	77% (68,86)	51% (42,61)	0.26 (0.13,0.39)	<0.01
**Chronic Kidney Disease (CKD)**					
With CKD	45	68% (48,88)	35% (15,54)	0.33 (0.06,0.61)	0.02
Without CKD	155	79% (70,88)	56% (47,66)	0.23 (0.09,0.36)	<0.01
**Presence of any co–morbidity (HIV, DM, CKD)**					
At least 1 co–morbidity	77	68% (52,83)	45% (29,61)	0.23 (0.01,0.44)	0.04
No co–morbidities	123	82% (73,91)	56% (45,67)	0.27 (0.12,0.41)	<0.01

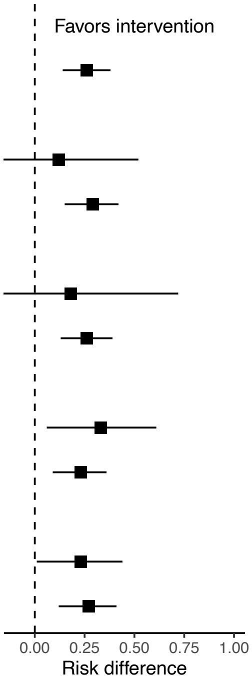

Supplemental sub-group analysis of hypertension control at 24 weeks assessed by research assistants in all study participants. The HIV sub-group was pre-specified in the statistical analysis plan; all other sub-groups were *post hoc* analyses requested during peer review. Abbreviations: Human immunodeficiency virus, HIV; Diabetes Mellitus, DM; Chronic Kidney Disease, CKD.

Figure C: Hypertension control at 48 weeks by presence of co-morbidities

Hypertension control at week 48, by comorbidity status

	N	Intervention	Control	Risk difference (95% CI)	p–value
**All**	200	86% (79,92)	44% (34,54)	0.42 (0.30,0.53)	<0.01
**HIV**					
With HIV	27	75% (49,101)	47% (20,74)	0.28 (−0.09,0.66)	0.13
Without HIV	147	89% (83,95)	45% (33,56)	0.45 (0.32,0.58)	<0.01
**Diabetes (DM)**					
With DM	15	75% (41,109)	71% (33,110)	0.04 (−0.48,0.55)	0.88
Without DM	185	87% (80,93)	42% (32,52)	0.45 (0.33,0.57)	<0.01
**Chronic Kidney Disease (CKD)**					
With CKD	45	73% (54,92)	39% (19,59)	0.34 (0.06,0.61)	0.02
Without CKD	155	90% (83,96)	45% (34,56)	0.45 (0.32,0.57)	<0.01
**Presence of any co–morbidity (HIV, DM, CKD)**					
At least 1 co–morbidity	77	76% (62,89)	48% (32,63)	0.28 (0.07,0.49)	<0.01
No co–morbidities	123	92% (86,98)	41% (29,53)	0.51 (0.38,0.64)	<0.01

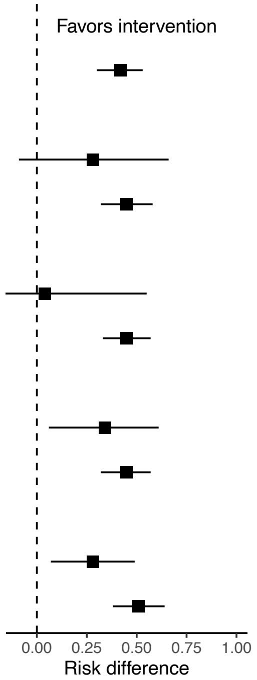

Supplemental sub-group analysis of hypertension control at 48 weeks assessed by research assistants in all study participants. The HIV sub-group was pre-specified in the statistical analysis plan; all other sub-groups were *post hoc* analyses requested during peer review. Abbreviations: Human immunodeficiency virus, HIV; Diabetes Mellitus, DM; Chronic Kidney Disease, CKD.

Figure D. Time to hypertension control

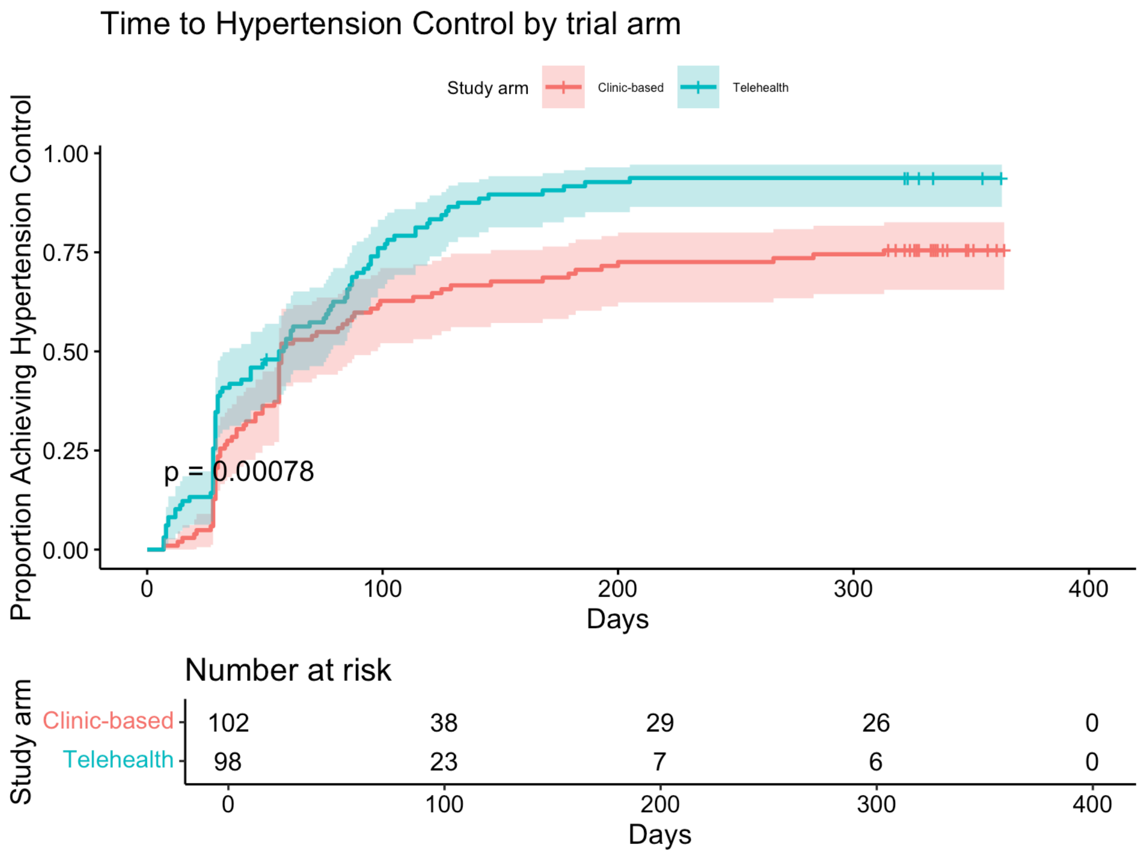

Time to hypertension control using data from clinical hypertension visits (telehealth or clinic-based). Note this *post hoc* analysis, requested during peer review, may be influenced by differential measurement by trial arm due to higher levels of retention in care (and therefore more measurement) in the telehealth intervention arm.

Figure E. Facilitators of hypertension care engagement

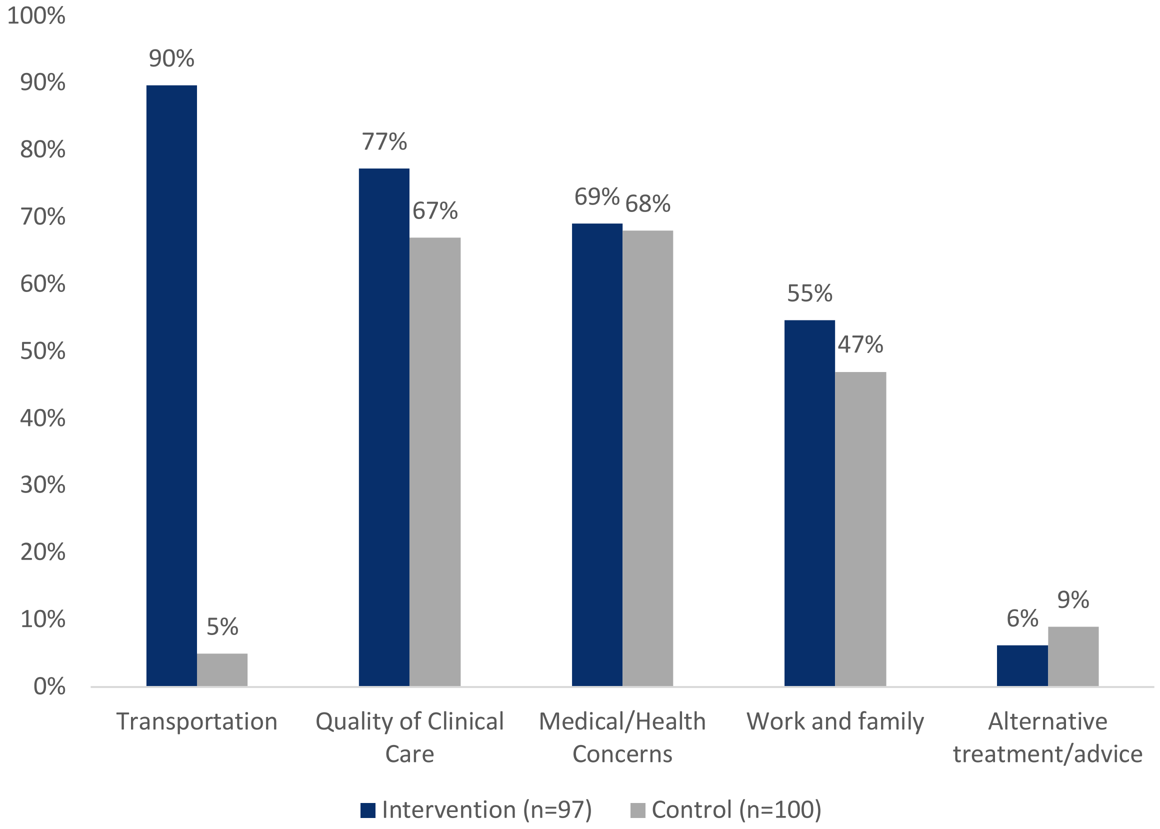

Figure F. Barriers to hypertension care engagement

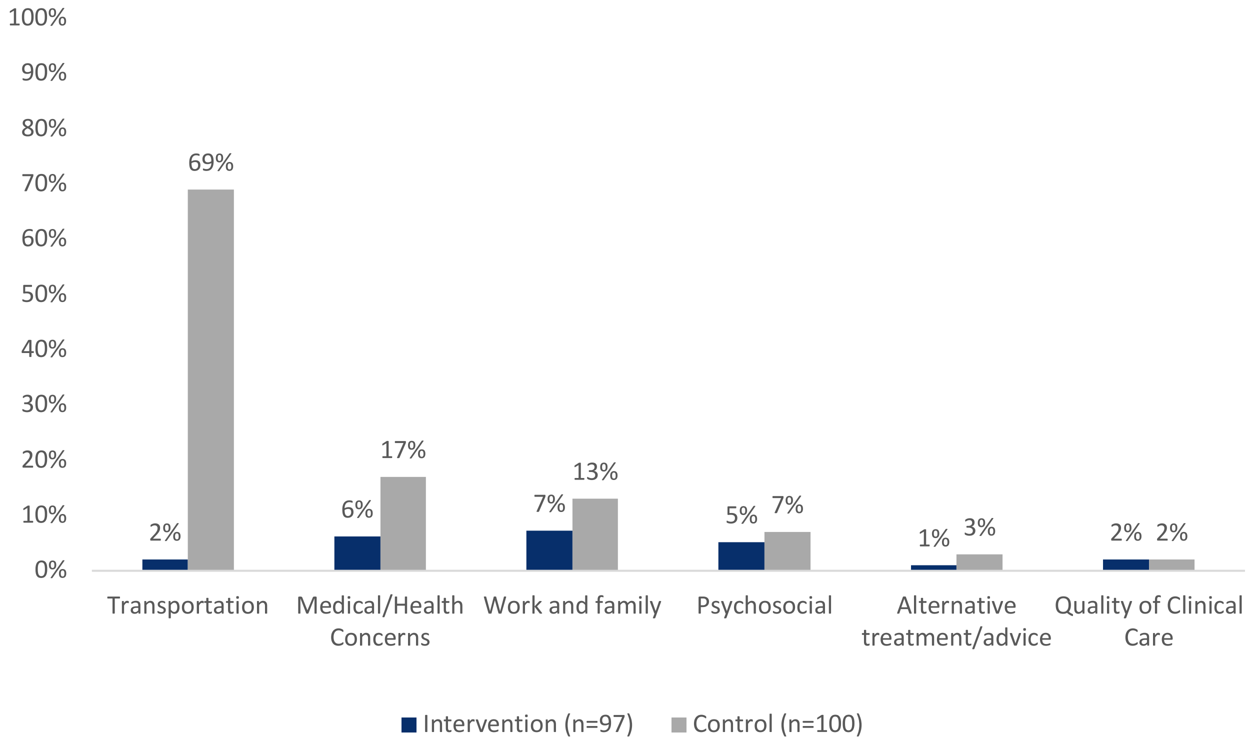

References

1. Powell BJ, Waltz TJ, Chinman MJ, Damschroder LJ, Smith JL, Matthieu MM, et al. A refined compilation of implementation strategies: results from the Expert Recommendations for Implementing Change (ERIC) project. Implement Sci IS. 2015;10: 21. doi:10.1186/s13012-015-0209-1

2. Cochrane effective practice and organisation of care. Available: https://epoc.cochrane.org/epoc-taxonomy

3. Lujintanon S, Eshun-Wilson I, Tourneau NL, Beres L, Schwartz S, Baral S, et al. Implementation strategies to improve HIV care cascade outcomes in low- and middle-income countries: a systematic review from 2014 to 2021. J Int AIDS Soc. 2024;27: e26263. doi:10.1002/jia2.26263

4. Balzer LB, Cai E, Godoy Garraza L, Amaranath P. Adaptive selection of the optimal strategy to improve precision and power in randomized trials. Biometrics. 2024;80: ujad034. doi:10.1093/biomtc/ujad034

5. Benkeser D, Díaz I, Luedtke A, Segal J, Scharfstein D, Rosenblum M. Improving precision and power in randomized trials for COVID-19 treatments using covariate adjustment, for binary, ordinal, and time-to-event outcomes. Biometrics. 2021;77: 1467–1481. doi:10.1111/biom.13377

6. van der Vaart AW. Asymptotic Statistics. Cambridge: Cambridge University Press; 1998. doi:10.1017/CBO9780511802256
