# Supplementary material for: Community health worker–facilitated telehealth for moderate–severe hypertension care in Kenya and Uganda: A randomized controlled trial
Source: PLoS Med. 2025 Jun 5;22(6):e1004632. doi: 10.1371/journal.pmed.1004632 (PMC12165344; doi:10.1371/journal.pmed.1004632)

Protocol Synopsis: SEARCH Sapphire Phase A Pilot Randomized Study of Community Health Worker facilitated telehealth for moderate/severe hypertension (NCT 04810650)

The SEARCH Sapphire Phase A Pilot Randomized study of community health worker facilitated telehealth for moderate/severe hypertension is one of a suite of randomized pilot studies in Phase A of the SEARCH Sapphire study. This synopsis is for the Phase A hypertension study only.

Study overview

The SEARCH Sapphire Phase pilot randomized study of community health worker facilitated telehealth for moderate/severe hypertension is evaluating whether a community-based hypertension care model, involving clinician telehealth and lay health worker support, improves hypertension control compared to standard clinic-based care. Conducted in rural Kenya and Uganda, the study enrolled adults aged 40 years and older with moderate/severe hypertension, randomizing them to either the community-based intervention or clinic-based control standard of care. The primary outcome is hypertension control at 24 weeks, with secondary outcomes including retention in care, mean systolic blood pressure levels at 24 and 48 weeks, and hypertension control at 48 weeks.

Rationale

Hypertension is underdiagnosed and undertreated in Sub-Saharan Africa. Untreated hypertension leads to stroke, myocardial infarction, kidney failure, heart failure and other complications. Interventions that improve diagnosis and treatment can reduce cardiovascular morbidity and mortality. Persons at highest risk for these complications have moderate/severe hypertension.

Study Hypothesis:

The hypothesis that community-based hypertension care (intervention) will improve hypertension control compared to clinic-based, patient-centered care (control) in adults aged 40 years and greater with moderate/severe hypertension (≥160/100 mmHg). The study seeks to evaluate whether an integrated community-based approach, which includes clinician telehealth, lay health worker blood pressure measurement, and medication delivery, is more effective than the standard clinic-based approach.

Study Objectives

The primary objective of this pilot study is to determine whether the intervention improved hypertension control at 24 weeks. Secondary endpoints include retention in care, mean systolic blood pressure at 24 and 48 weeks, and hypertension control at 48 weeks. We will also

characterize changes in hypertension severity and evaluate predictors HTN control and retention.

Study Population

The population of interest comprises non-pregnant adults, aged 40 years and greater if blood pressure was elevated at both community-based and clinic measurement (≥140 mmHg systolic or ≥90 mmHg diastolic) and moderate-severely elevated on at least one of these measurements (≥160 mmHg systolic or ≥100 mmHg diastolic).

Study Intervention

After community-based screening by community health workers, individuals with moderate/severe hypertension were referred to the nearest government-run clinic for clinical assessment and enrollment. Following enrollment and completion of an initial clinic visit, participants were randomized to the intervention (n=98) or control (n=102) conditions. To ensure balance between arms, randomization was stratified by country and sex and was implemented by an independent statistician using a stratified random block design with random block sizes 2 and 4.

Intervention participants received home-based, follow-up care for hypertension, which consisted of blood pressure measurement and adherence assessment by a community health worker, a telehealth visit with a clinician, and medication dispensation by the community health worker according to clinician orders. Clinicians had the option to request the participant attend an in person visit if medically indicated. Control participants received clinic-based integrated, patient-centered hypertension care. Treatment guidelines for use of antihypertensive medication for both study arms were based on standard country guidelines.

Study outcomes

Primary: Hypertension control at 24 weeks
Secondary: Retention in care, mean systolic blood pressure at 24 and 48 weeks, hypertension control at 48 weeks.

In addition, we will report and describe implementation outcomes including intervention fidelity; delivery of hypertension care in both arms, as well as barriers and facilitators of hypertension care engagement.

Sample size and power

Sample size and power calculations were based on standard formulas for a two-sample test of proportions and done with power.prop.test in R.13 We expect these calculations to be conservative, because of the precision gained through stratified randomization and through covariate adjustment during the analysis.

We estimated 200 participants (~100/arm) would provide 80% power to detect at least a 20% absolute increase in hypertension control from 40% under the standard-of-care at 24 weeks

(i.e., 6 months). Even with 20% fewer participants enrolled (from 100 to 80 participants/arm) and lower or higher control under the standard-of-care, these calculations suggest we would be well-powered to detect at least a 22% absolute increase in control.

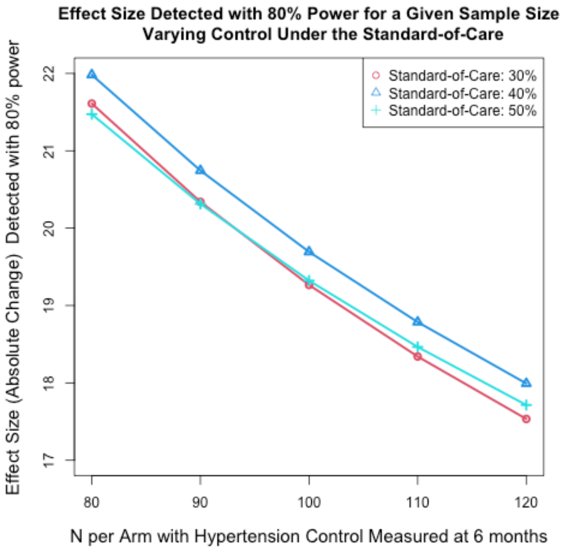

Supplement: S2 File — (PDF) [file pmed.1004632.s002.pdf]
