# Supplementary material for: Community health worker–facilitated telehealth for moderate–severe hypertension care in Kenya and Uganda: A randomized controlled trial
Source: PLoS Med. 2025 Jun 5;22(6):e1004632. doi: 10.1371/journal.pmed.1004632 (PMC12165344; doi:10.1371/journal.pmed.1004632)

CLINICAL HYPERTENSION TREATMENT PROTOCOL - KENYA

Introduction
This protocol summarizes clinical hypertension treatment guidelines from the 2018 Kenya National Guidelines for Cardiovascular Diseases Management.

Evaluation
Patients with hypertension should be evaluated for associated co-morbidities that may increase risk of complications including myocardial infarction, heart failure, chronic kidney disease, peripheral vascular disease, retinopathy, or cerebrovascular accident.

All patients should be evaluated for the following (when available):

- Cardiovascular (CVD) risk factors:
  - Smoking
  - Diabetes mellitus (fasting glucose ≥7.0 mmol/L or random glucose 11.1 mmol/L)
  - Age (men ≥55 years, women ≥65 years)
  - Obesity (Body Mass Index ≥30 kg/m^2)
- CVD Complications
  - Coronary heart disease
  - Heart failure
  - Chronic kidney disease (eGFR <60 ml/min/1.73m^2 or ≥1+ proteinuria on urine dipstick)
  - Stroke or transient ischemic attack (TIA)
  - Advanced retinopathy (hemorrhages, exudates, or papilledema)

Management
Per current Ministry of Health Guidelines, the goal of hypertension treatment is to achieve blood pressure control **<140/90 mmHg** and address associated risk factors and complications. CVD Complications should be treated by or in consultation with a physician specialist.

Every clinic visit should include counseling on healthy lifestyle, including the following:

- Avoidance of alcohol
- Tobacco cessation
- Physical exercise: at least 30 minutes of moderate-intensity aerobic exercise 5-7 days/week
- Diet: increased fruit and vegetable intake, reduced saturated fat, avoid added salt and high salt food
- Weight reduction for overweight (Body Mass Index [BMI] 25-29.9 kg/m^2) and obese (BMI ≥30 kg/m^2) hypertensive patients

Recommended thresholds for pharmacologic treatment initiation:

- **Blood pressure 140-159/90-99 mmHg and no CVD risk factors**: lifestyle counseling for 4-12 weeks. If blood pressure remains ≥140/90, pharmacologic treatment should be initiated.
- **Blood pressure 140-159/90-99 mmHg AND CVD risk factors, target-organ damage, and/or CVD complications:** lifestyle counseling and concurrent pharmacologic treatment initiation
- **Blood pressure ≥160/100 mmHg:** lifestyle counseling and initiation of 2-drug therapy

Treatment Algorithm for Patients with Hypertension in Kenya*

BP 140-159/90-99

Step 1 — **Lifestyle counseling**

4-12 weeks†

Step 2 — If SBP ≥140 or DBP ≥90: *INITIATE starting dose CCB‡*
Nifedipine LA 40mg daily

4 weeks

Step 3 — If SBP ≥140 or DBP ≥90: *ADD starting dose thiazide*
Nifedipine LA 40 mg daily + HCTZ 25mg daily

4 weeks

Step 4 — If SBP ≥140 or DBP ≥90: *INCREASE to full dose CCB*
Nifedipine LA 80 mg daily + HCTZ 25mg daily

4 weeks

Step 5 — If SBP ≥140 or DBP ≥90: *INCREASE to full dose thiazide*
Nifedipine LA 80 mg daily + HCTZ 50mg daily

4 weeks

Step 6 — If SBP ≥140 or DBP ≥90: *ADD starting dose ACE/ARB*
Nifedipine LA 80 mg daily + HCTZ 50mg daily + Enalapril 10 mg daily

4 weeks

Step 7 — If SBP ≥140 or DBP ≥90: *INCREASE to full dose ACE/ARB*
Nifedipine LA 80 mg daily + HCTZ 50mg daily + Enalapril 20mg daily

4 weeks

Step 8 — If SBP ≥140 or DBP ≥90
CONTINUE therapy and
REFER to Physician specialist

BP ≥160/100

Initiate Steps 1-3 simultaneously:

Lifestyle counseling

and

Nifedipine LA 40 mg daily +
HCTZ 25 mg daily
(2-drug therapy)

4 weeks (→ Step 4)

If BP <140/90: continue current dose and follow-up in 12 weeks.

Check adherence in a nonjudgmental way at each visit. If nonadherent and BP ≥140/90, consider continuing current dose, adherence counseling, and 4 week follow-up.

ACE-I/ARBs are contraindicated in pregnancy
Do not use this algorithm for pregnant women with hypertension – refer to a specialist

IF SBP ≥180 or DBP ≥110 mmHg at any point: Clinician must assess for hypertensive emergency
Assess for: severe headache, confusion, chest pain, difficulty breathing, vision changes, unilateral weakness
Action: If patient offsite, immediate transportation to the clinic. Clinician to guide whether to administer immediate therapy (if concerns for acute ischemic stroke, avoid abrupt BP lowering).

Medication Class	Medication	Starting Dose	Full Dose	Possible side effects
**CCB** (Calcium Channel Blocker)	**Nifedipine LA**	**40 mg Daily**	**80 mg Daily**	• Leg edema<br>• Fatigue<br>• Headache<br>• Palpitations
	Amlodipine	5 mg Daily	10 mg Daily	
	Felodipine	5 mg Daily	10 mg Daily	
**Thiazide** diuretic	**Hydrochlorothiazide (HCTZ)**	**12.5-25 mg**	**25-50 mg**	• Hypokalemia<br>• Hyponatremia<br>• Hyperuricemia<br>• Hypocalciuria<br>• Hyperglycemia<br>• Rash<br>• Dyslipidemia
	Chlorthalidone	25 mg Daily	50 mg Daily	
	Bendroflumethiazide	5 mg	10 mg	
**ARB** (Angiotensin Receptor Blocker)	Losartan	50 mg Daily	100 mg Daily	• Cough (ACE-I only)<br>• Hyperkalemia<br>• Increased serum creatinine<br>• Angioedema
	Telmisartan	40 mg Daily	80 mg Daily	
	Valsartan	80 mg Daily	160 mg Daily	
**ACE** (ACE-inhibitor)	**Enalapril**	**10 mg Daily**	**40 mg Daily**	
	Lisinopril	10-20 mg Daily	40 mg Daily	
	Benazepril	10-20 mg Daily	40 mg Daily	

* Algorithm based on Kenya National Guidelines for CVD Management (2018)[1]. Can substitute alternative drugs from the above table if listed medications are not available. Note: ACE/ARB is recommended 2nd line drug in guidelines; consider replacing HCTZ with ACE/ARB if available and indicated

† In presence of CVD risk factors and/or complications, initiate lifestyle counseling (Step 1) & medication (Step 2) simultaneously

‡ A thiazide can be started as the initial medication if CCB is not available.

Abbreviations: SBP, Systolic Blood Pressure; DBP, Diastolic Blood Pressure; LA, Long Acting; CCB, Calcium Channel Blocker; ACE-I, Angiotensin-converting enzyme inhibitor; ARB, Angiotensin II receptor blocker; mg, milligrams

References: 1. Kenya National Guidelines for Cardiovascular Diseases Management. Division of Non-Communicable Diseases, Ministry of Health, Kenya; 2018.

CLINICAL HYPERTENSION TREATMENT PROTOCOL - UGANDA

Introduction
This protocol summarizes clinical hypertension treatment guidelines from the 2016 Uganda Clinical Guidelines.

Evaluation
Patients with hypertension should be evaluated for associated co-morbidities that may increase risk of complications including myocardial infarction, heart failure, chronic kidney disease, peripheral vascular disease, retinopathy, or cerebrovascular accident.

All patients should be evaluated for the following (when available):
- Cardiovascular (CVD) risk factors:
  - Smoking
  - Diabetes mellitus (fasting glucose ≥7.0 mmol/L or random glucose 11.1 mmol/L)
  - Age (men ≥55 years, women ≥65 years)
  - Obesity (Body Mass Index ≥30 kg/m^2)
- CVD Complications
  - Coronary heart disease
  - Heart failure
  - Chronic kidney disease (eGFR <60 ml/min/1.73m^2 or ≥1+ proteinuria on urine dipstick)
  - Stroke or transient ischemic attack (TIA)
  - Advanced retinopathy (hemorrhages, exudates, or papilledema)

Management
Per current Ministry of Health Guidelines, the goal of hypertension treatment is to achieve blood pressure control **<140/90 mmHg** and address associated risk factors and complications. CVD Complications should be treated by or in consultation with a physician specialist.

Every clinic visit should include counseling on healthy lifestyle, including the following:
- Avoidance of alcohol
- Tobacco cessation
- Physical exercise: at least 30 minutes of moderate-intensity aerobic exercise 5-7 days/week
- Diet: increased fruit and vegetable intake, reduced saturated fat, avoid added salt and high salt food
- Weight reduction for overweight (Body Mass Index [BMI] 25-29.9 kg/m^2) and obese (BMI ≥30 kg/m^2) hypertensive patients

Recommended thresholds for pharmacologic treatment initiation:
- **Blood pressure 140-159/90-99 mmHg and no CVD risk factors**: lifestyle counseling for 4-12 weeks. If blood pressure remains ≥140/90, pharmacologic treatment should be initiated.
- **Blood pressure ≥160/100 mmHg:** lifestyle counseling and initiation of 2-drug therapy

Treatment Algorithm for Patients with Hypertension in Uganda*

BP 140-159/90-99

- **Step 1** — **Lifestyle counseling**
  - 4-12 weeks
- **Step 2** — If SBP ≥140 or DBP ≥90: *INITIATE starting dose CCB‡*
  Nifedipine LA 40 mg daily
  - 4 weeks
- **Step 3** — If SBP ≥140 or DBP ≥90: *ADD starting dose thiazide*
  Nifedipine LA 40 mg daily + Bendroflumethiazide (BFTZ) 5 mg daily
  - 4 weeks
- **Step 4** — If SBP ≥140 or DBP ≥90: *INCREASE to full dose CCB*
  Nifedipine LA 80 mg daily + BFTZ 5 mg daily
  - 4 weeks
- **Step 5** — If SBP ≥140 or DBP ≥90: *INCREASE to full dose thiazide*
  Nifedipine LA 80 mg daily + BFTZ 10 mg daily
  - 4 weeks
- **Step 6** — If SBP ≥140 or DBP ≥90: *ADD starting dose ARB/ACE-I*
  Nifedipine LA 80 mg daily + BFTZ 10 mg daily + Losartan 50 mg daily
  - 4 weeks
- **Step 7** — If SBP ≥140 or DBP ≥90: *INCREASE to full dose ARB/ACE-I*
  Nifedipine LA 80 mg daily + BFTZ 10 mg daily + Losartan 100mg daily
  - 4 weeks
- **Step 8** — If SBP ≥140 or DBP ≥90
  CONTINUE therapy and REFER to Physician specialist

BP ≥160/100

Initiate Steps 1-3 simultaneously:

Lifestyle counseling

and

Bendroflumethiazide (BFTZ) 5 mg daily + Nifedipine XR 40 mg daily (2-drug therapy)

4 weeks → Step 4

If BP <140/90: continue current dose and follow-up in 12 weeks.

Check adherence in a nonjudgmental way at each visit. If nonadherent and BP ≥140/90, consider continuing current dose, adherence counseling, and 4 week follow-up.

ACE-I/ARBs are contraindicated in pregnancy
Do not use this algorithm for pregnant women with hypertension – refer to a specialist

IF SBP ≥180 or DBP ≥110 mmHg at any point: Clinician must assess for hypertensive emergency
Assess for: severe headache, confusion, chest pain, difficulty breathing, vision changes, unilateral weakness
Action: If patient offsite, immediate transportation to the clinic. Clinician to guide whether to administer immediate therapy (if concerns for acute ischemic stroke, avoid abrupt BP lowering).

Medication Class	Medication	Starting Dose	Full Dose	Possible side effects
**CCB** (Calcium Channel Blocker)	**Nifedipine LA**	**40 mg Daily**	**80 mg Daily**	• Leg edema • Fatigue • Headache • Palpitations
	Amlodipine	5 mg Daily	10 mg Daily	
	Felodipine	5 mg Daily	10 mg Daily	
**Thiazide** diuretic	**Bendroflumethiazide (BFTZ)**	**5 mg**	**10 mg**	• Hypokalemia • Hyponatremia • Hyperuricemia • Hypocalciuria • Hyperglycemia • Rash • Dyslipidemia
	Chlorthalidone	25 mg Daily	50 mg Daily	
	Hydrochlorothaizide (HCTZ)	12.5 mg	25 mg	
**ARB** (Angiotensin Receptor Blocker)	**Losartan**	**50 mg Daily**	**100 mg Daily**	• Cough (ACE-I) • Hyperkalemia • Increased serum creatinine • Angioedema
	Telmisartan	40 mg Daily	80 mg Daily	
	Valsartan	80 mg Daily	160 mg Daily	
**ACE-I** (ACE-inhibitor)	Lisinopril	20 mg Daily	40 mg Daily	
	Benazepril	20 mg Daily	40 mg Daily	

* Algorithm based on Uganda Clinical Guidelines (2016)[1]. Can substitute alternative drugs from the above table in the event listed medications are not available.

‡ A thiazide or CCB can be started as the initial medication. If both available, CCB is preferred due to lower side effect profile.

Abbreviations: SBP, Systolic Blood Pressure; DBP, Diastolic Blood Pressure; LA, Long Acting; CCB, Calcium Channel Blocker; ACE-I, Angiotensin-converting enzyme inhibitor; ARB, Angiotensin II receptor blocker; mg, milligrams

References: 1. Uganda Clinical Guidelines: National Guidelines for Management of Common Conditions. Republic of Uganda Ministry of Health; 2016.

Appendix 3. Diabetes Management Algorithm

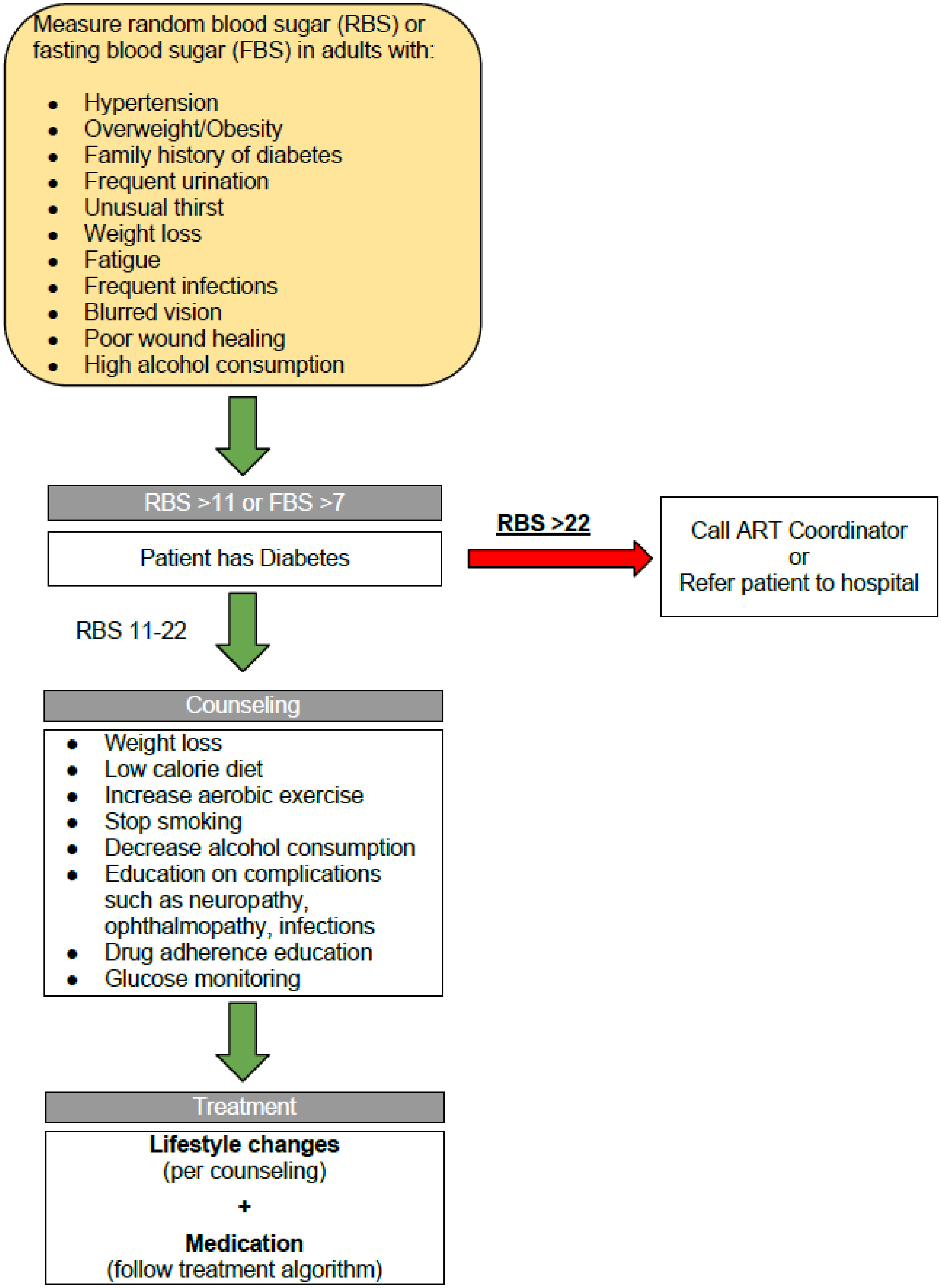

Appendix 4. Diabetes Drug Use Algorithm

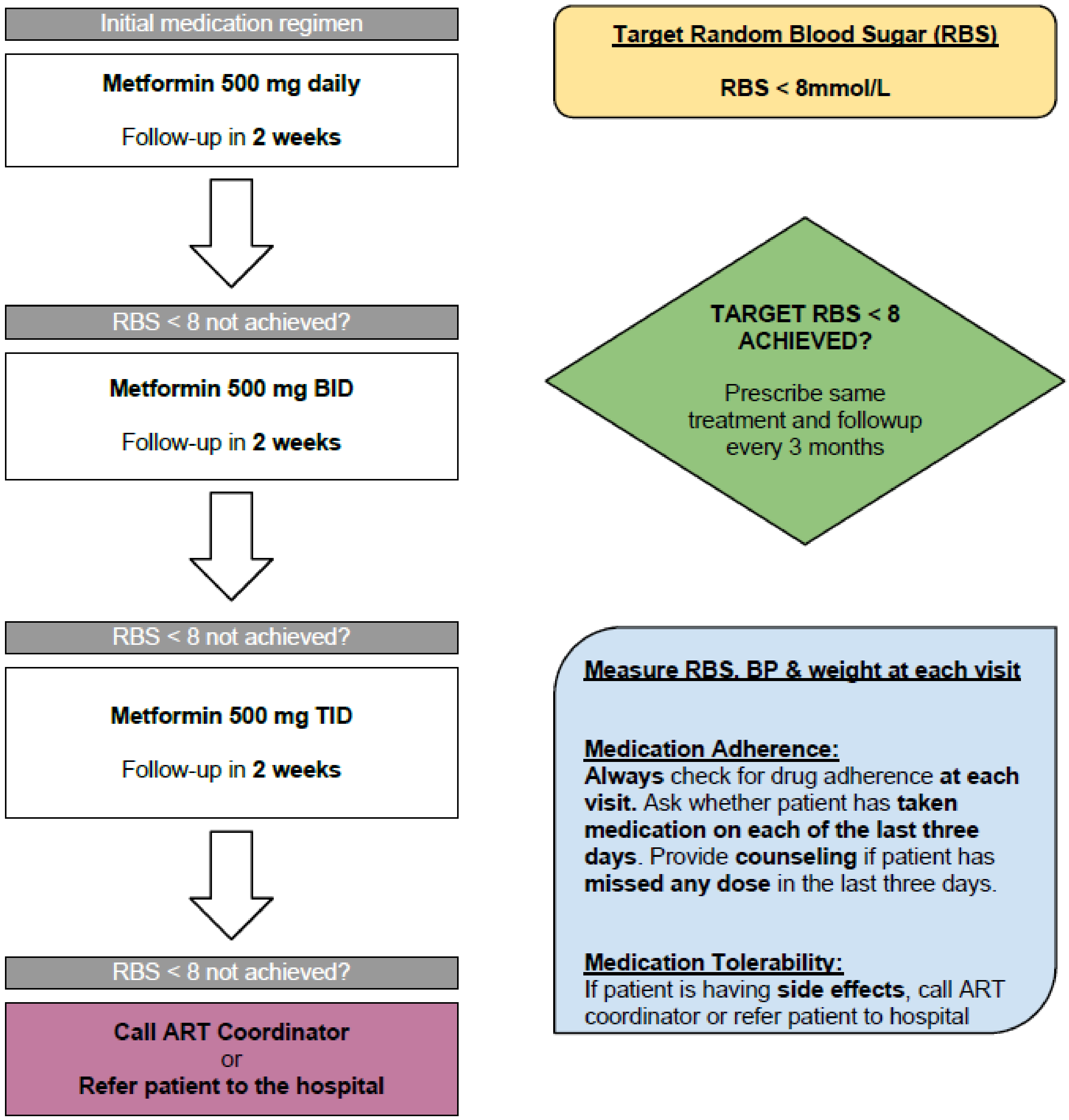

Supplement: S3 File — (PDF) [file pmed.1004632.s003.pdf]
