# Supplementary material for: Community health worker–facilitated telehealth for moderate–severe hypertension care in Kenya and Uganda: A randomized controlled trial
Source: PLoS Med. 2025 Jun 5;22(6):e1004632. doi: 10.1371/journal.pmed.1004632 (PMC12165344; doi:10.1371/journal.pmed.1004632)
Supplement: S4 File — (PDF) [file pmed.1004632.s004.pdf]

A Multisectoral Strategy to Address Persistent Drivers
of the HIV Epidemic in East Africa (SAPPHIRE)

Sustainable East Africa Research in Community Health (SEARCH) Consortium

Statistical Analysis Plan

Project: Effect of Community Health Worker Facilitated Telehealth Intervention for Severe Hypertension Management in SEARCH SAPPHIRE

Laura B. Balzer, PhD*
James Ayieko, MBChB, MPH, PhD**
Diane V. Havlir, MD***
Maya L. Petersen, MD, PhD*
Moses Kamya, MMed, MPH, PhD****
Matthew D. Hickey, MD***

for the SEARCH Collaboration

June 1, 2023

Version 1.0

*School of Public Health, University of California Berkeley, Berkeley, USA
**Kenya Medical Research Institute, Nairobi, Kenya
***Division of HIV, ID, & Global Medicine, Department of Medicine, UCSF, San Francisco, USA
**** Makerere University, Department of Medicine, Kampala, Uganda

Table of Contents

1. Overview 2

2.1 General approach for evaluating intervention effects 3

2.2 Evaluate the effect on hypertension control at week 24 and 48 4

2.3 Evaluate the effect on retention in care at week 24 and 48 5

2.4 Evaluate the effect on average systolic blood pressure at week 24 and 48 5

2.5 Evaluate the effect on time to hypertension control 5

2.6 Assess predictors of hypertension control and retention in care 5

Appendix: Power calculations 6

References 7

1. Overview

The SEARCH-Sapphire Severe Hypertension (HTN) Treatment study is an individual randomized controlled trial testing the hypothesis that community-based hypertension care (intervention) will improve control of hypertension, as compared to clinic-based, patient-centered care (control) in adults aged 40+ with severe hypertension (blood pressure ≥160/100 mmHg). The community-based intervention consists of clinician telehealth combined with lay health worker blood pressure measurement and medication delivery. The pilot trial takes place in three communities in Kenya and Uganda and began enrollment in May 2022.

After community-based screening by community health workers, individuals with severe hypertension were referred to the nearest government-run clinic for clinical assessment and enrollment. Following enrollment and completion of an initial clinic visit, participants were randomized to the intervention (n=98) or control (n=102) conditions. To ensure balance between arms, randomization was stratified by country and sex and was implemented by an independent statistician using a stratified random block design with random block sizes 2 and 4.

Intervention participants received home-based, follow-up care for hypertension, which consisted of blood pressure measurement and adherence assessment using pill count by a community health worker, a telehealth visit with a clinician, and medication dispensation by the community health worker according to clinician orders. Control participants received clinic-based integrated, patient-centered hypertension care, detailed elsewhere.[1–3] Treatment guidelines for use of antihypertensive medication for both study arms were based on standard country guidelines. Additional details of the study procedures are available in the Study Protocol.

The primary objective of this study is to determine whether the intervention improved hypertension control at 24 weeks. Secondary endpoints include retention in care, mean systolic blood pressure at 24 and 48 weeks, and hypertension control at 48 weeks. We will also characterize changes in hypertension severity and evaluate predictors HTN control and retention.

Throughout, the population of interest comprises non-pregnant adults, aged 40+ if blood pressure was elevated at both community-based and clinic measurement (≥140 mmHg systolic or ≥90 mmHg diastolic) and moderate-severely elevated on at least one of these measurements (≥160 mmHg systolic or ≥100 mmHg diastolic). Participants were excluded if they were pregnant – these individuals were immediately referred to the prenatal clinic for evaluation and treatment of hypertension in pregnancy.

We will provide descriptions of participant flow through the study (i.e., a consort diagram), measurement coverage, and baseline characteristics (e.g., country, age, sex, prior history of hypertension diagnosis and baseline treatment, comorbidities, and baseline severity of hypertension). We will also report risk factors (N and %) for uncontrolled hypertension at enrollment. We will provide these descriptive statistics, overall and by arm.

2.1 General approach for evaluating intervention effects

We will assess the intervention effect with targeted minimum loss-based estimation (TMLE), which provides precision and power gains over an unadjusted effect estimator (e.g., a t-test) by adjusting for baseline predictors of the outcome.[4–7] For a detailed review of TMLE and its relation to other effect estimators in randomized trials, we refer the reader to Colantuoni and Rosenblum.[8] For a recent demonstration of the improved precision offered by TMLE in randomized trials, we refer the reader to Balzer et al. and to Benkser et al..[9,10]

Here, we will use **TMLE with Adaptive Pre-specification**, a fully automated procedure to flexibly adjust for baseline outcome predictors, while maintaining Type-I error control.[11,12] Specifically, using 10-fold cross-validation, we will chose the optimal approach for estimating the expected outcome given the trial arm and baseline covariates (a.k.a., the outcome regression) and for estimating the conditional probability of being randomized to the intervention given the baseline covariates (a.k.a., the propensity score). Throughout, optimality is defined by using the squared (estimated) influence curve for the TMLE as loss function. Thereby, we will select the combination of estimators (adjustment variables + approach) for the outcome regression and the propensity score that minimizes the cross-validated risk estimate and, thus, maximizes empirical efficiency.

Our pre-specified, candidate adjustment variables are age, sex, baseline HTN severity as assessed at enrollment ("grade 1" as 140-159/90-99 mmHg; "grade 2" as 160-179/100-109 mmHg, and "grade 3" as ≥180/110 mmHg), country, and nothing (i.e., unadjusted). Our pre-specified candidate estimators are main terms, stepwise

regression, adjustment for a single covariate, and the mean. In sensitivity analyses, we will also implement that unadjusted effect estimator as the contrasts of average outcomes by arm.

Primary estimates will be for the study sample and on the **difference scale**: $\frac{1}{n}\sum_{i=1}^{n}[Y_i(1) - Y_i(0)]$, where $Y_i(1)$ denotes the counterfactual outcome for participant *i* under the intervention and $Y_i(0)$ denotes the counterfactual outcome for participant *i* under the control. Secondary comparisons will be on the relative scale.

For all endpoints, we will test the **null hypothesis** of no change in outcomes due to the intervention with a two-sided test at the 5% significance level. We will also report point estimates and 95% confidence intervals for each effect measure and the arm-specific average outcomes. Standard error estimation will be with the estimated influence curve, and statistical inference will follow from the Central Limit Theorem (i.e., using the standard normal distribution).[4]

For all endpoints, we will also examine the intervention effect within subgroups defined by sex, age group (<60 years vs. ≥60 years), country, baseline HTN severity, and HIV status. In subgroups with fewer than 51 participants, we will conduct unadjusted analyses.

2.2 Evaluate the effect on hypertension control at week 24 and 48

The primary endpoint is hypertension control at week 24, evaluated in all participants in a research visit at week 24 and defined as blood pressure < 140/90 mmHg on the average of the second and third measurements. Participants who miss their week 24 visit and, thus, are missing blood pressure measurement at week 24 will be assumed to be uncontrolled.

As described above, we will use TMLE with Adaptive Pre-specification to compare hypertension control between intervention and control arms and to test the null hypothesis that the intervention did not impact hypertension control. We will conduct sensitivity analyses using TMLE to adjust for differences in characteristics (e.g., arm, age sex, sex, country, baseline severity) between persons with measurements and persons with missing measurements. We may conduct an additional sensitivity analysis excluding participants with missing values.

A secondary endpoint is hypertension control at week 48, assessed in the same manner as hypertension control at week 24.

At week 24 and week 48, we will also evaluate the intervention impact on hypertension severity, defined as participants with grade 2 or higher hypertension. For the severity endpoint, our primary approach will be TMLE to adjust for differences between persons with and without measured outcomes, and the secondary analysis will exclude participants with missing outcomes.

2.3 Evaluate the effect on retention in care at week 24 and 48

Using an analogous approach, we will formally compare retention in care by trial arm at week 24 and at week 48. We define retention in care as not late for the most recent scheduled hypertension care appointment by 30 days or more. This endpoint is not subject to missingness. Within TMLE and Adaptive Pre-specification, we will use the same candidate adjustment variables and approaches as the primary endpoint. Sensitivity analyses will be unadjusted.

2.4 Evaluate the effect on average systolic blood pressure at week 24 and 48

We will assess the intervention effect on mean systolic blood pressure at week 24 and week 48. Mean systolic blood pressure will be assessed using the average of the second and third blood pressure measurement taken by research staff at these timepoints. We will use the same candidate adjustment variables and estimation approaches as the primary endpoint.

In the primary approach, we will assume persons without a blood pressure measurement at the timepoint of interest are out-of-care and their blood pressure has reverted back to baseline. In other words, we will impute missing systolic blood pressure measures with their baseline measure. In sensitivity analyses, we will control for incomplete ascertainment of blood pressure measures. Specifically, we will use TMLE to adjust for differences in characteristics between persons with measurements and persons without measurements. For comparison, we may also conduct a complete-case analysis (i.e., restricting to participants with known outcomes.)

2.5 Evaluate the effect on time to hypertension control

Using routine clinical data, we may evaluate the intervention effect on the time to hypertension control (<140/90 mmHg). To conduct such an analysis, we would use Kaplan-Meier to compare the cumulative incidence of attaining hypertension control over the first 24 weeks using routine clinical data and censoring at death.

2.6 Assess predictors of hypertension control and retention in care

We may evaluate the following predictors of hypertension control and of retention in care at 24 and 48 weeks: sex, age, country, baseline HTN severity (grade), baseline HIV status (assessed via testing and self-report), and chronic kidney disease (CKD) at baseline. In such analyses, TMLE would be used to obtain variable importance measures, capturing the amount of information that a given predictor provides after adjusting for the other predictors. Secondary analyses would be unadjusted (i.e., univariate) associations.

2.7 Implementation outcomes

We will report and describe intervention fidelity; delivery of hypertension care in both arms, as well as barriers and facilitators of hypertension care engagement. Our implementation outcomes include

- Reach: completion of ≥1 post-baseline clinical visit
- Fidelity: clinician fidelity to hypertension guideline:
    - If BP controlled: continuing medications and scheduling in 12 weeks
    - If BP uncontrolled: increasing medications (if adherence good) or continuing medications (if adherence poor) and scheduling in 2-4 weeks
- Acceptability:
    - Description of barriers and facilitators reported by participants at baseline, week 24, and week 48 by trial arm (structured survey data)*
- Cost

*If participants were missing survey responses at week 48, we carried forward responses from week 24.

Appendix: Power calculations

Sample size and power calculations were based on standard formulas for a two-sample test of proportions and done with *power.prop.test* in *R*.[13] We expect these calculations to be conservative, because of the precision gained through stratified randomization and through covariate adjustment during the analysis.

We estimated 200 participants (~100/arm) would provide 80% power to detect at least a 20% absolute increase in hypertension control from 40% under the standard-of-care at 24 weeks (i.e., 6 months). Even with 20% fewer participants enrolled (from 100 to 80 participants/arm) and lower or higher control under the standard-of-care, these calculations suggest we would be well-powered to detect at least a 22% absolute increase in control.

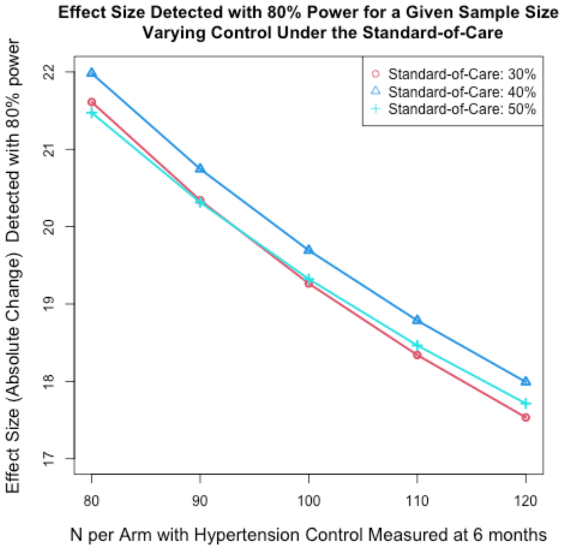

References

1. Kwarisiima D, Atukunda M, Owaraganise A, et al. Hypertension control in integrated HIV and chronic disease clinics in Uganda in the SEARCH study. *BMC Public Health*. 2019;19(1):511. doi:10.1186/s12889-019-6838-6

2. Kwarisiima D, Kamya M, Owaraganise A, et al. High rates of viral suppression in adults and children with high CD4+ counts using a streamlined ART delivery model in the SEARCH trial in rural Uganda and Kenya. *J Int AIDS Soc*. 2017;Jul 21(20).

3. Havlir DV, Balzer LB, Charlebois ED, et al. HIV Testing and Treatment with the Use of a Community Health Approach in Rural Africa. *N Engl J Med*. 2019;381(3):219-229.

4. van der Laan M, Rose S. *Targeted Learning: Causal Inference for Observational and Experimental Data*. Springer; 2011.

5. Moore KL, van der Laan MJ. Covariate Adjustment in Randomized Trials with Binary Outcomes: Targeted Maximum Likelihood Estimation. *Stat Med*. 2009;28(1):39-64. doi:10.1002/sim.3445

6. Rosenblum M, van der Laan MJ. Simple, Efficient Estimators of Treatment Effects in Randomized Trials Using Generalized Linear Models to Leverage Baseline Variables. *Int J Biostat*. 2010;6(1):Article 13. doi:10.2202/1557-4679.1138

7. van der Laan MJ, Rose S. *Targeted Learning in Data Science*. Springer; 2018.

8. Colantuoni E, Rosenblum M. Leveraging Prognostic Baseline Variables to Gain Precision in Randomized Trials. *Stat Med*. 2015;34(18):2602-2617.

9. Balzer LB, van der Laan M, Ayieko J, et al. Two-Stage TMLE to Reduce Bias and Improve Efficiency in Cluster Randomized Trials. *Biostatistics*. 2021;kxab043. https://doi.org/10.1093/biostatistics/kxab043

10. Benkeser D, Díaz I, Luedtke A, Segal J, Scharfstein D, Rosenblum M. Improving precision and power in randomized trials for COVID-19 treatments using covariate adjustment, for binary, ordinal, and time-to-event outcomes. *Biometrics*. 2021;n/a(n/a):1-15. doi:10.1111/biom.13377

11. Balzer L, van der Laan MJ, Petersen M, SEARCH Collaboration. Adaptive Pre-specification in Randomized Trials With and Without Pair-Matching. Stat Med. 2016;35(10):4528-4545. doi:10.1002/sim.7023

12. Balzer LB, Cai E, Garraza LG, Amaranath P. Adaptive Selection of the Optimal Strategy to Improve Precision and Power in Randomized Trials. https://arxiv.org/abs/2210.17453v2. Published online 2023

13. R Core Team. R: A Language and Environment for Statistical Computing. R Foundation for Statistical Computing; 2020. http://www.R-project.org
