# Supplementary material for: Community health worker–facilitated telehealth for moderate–severe hypertension care in Kenya and Uganda: A randomized controlled trial
Source: PLoS Med. 2025 Jun 5;22(6):e1004632. doi: 10.1371/journal.pmed.1004632 (PMC12165344; doi:10.1371/journal.pmed.1004632)
Supplement: S5 File — (PDF) [file pmed.1004632.s005.pdf]

A Multisectoral Strategy to Address Persistent Drivers of the HIV Epidemic in East Africa (SAPPHIRE)

Sustainable East Africa Research in Community Health (SEARCH) Consortium

Clinical Trial Phase: IV

Protocol Number: DAIDS-ES ID 38971

Funded By: National Institutes of Health

Protocol Chair: Diane Havlir, MD

Protocol Co-Chairs: Moses Kamya, MBChB, MPH, PhD
Maya Petersen, MD, PhD

Protocol Vice-Chair: Gabriel Chamie, MD, MPH

Version Number: 7.0

Version Date: 26-July-2023

National Clinical Trial (NCT) Identified Number: NCT04810650

Summary of Changes from Previous Version:

Affected Section(s)	Summary of Revisions Made	Rationale
Section 7.3.2	Updates to individual level inclusion criteria.	To clarify minor changes to inclusion criteria.
Section 9.3.2	Updated Intervention Procedures and Target Popluation table.	Updates made to reflect incorporation of previously separated intervention into LEAP intervention.
Appendix, section H	Updates to CAB-LA extension timeframe and analyses.	Minor updates to extend the timeline for participants in the CAB-LA extension arm in the event CAB-LA is not available in country, and to clarify the definition of covered time for participants on CAB-LA.
Multiple sections	Other minor clarifications and edits throughout protocol.	To clarify and correct minor points.

TABLE OF CONTENTS

1. KEY INVESTIGATOR ROLES 6
2. LIST OF ABBREVIATIONS 10
3. PROTOCOL SUMMARY 12
4. INTRODUCTION 15
4.1. Background 15
4.2. Study Hypothesis for Primary Objective 19
5. OBJECTIVES 20
5.1. Primary Objective 20
5.2. Secondary Objectives 20
6. STUDY DESIGN 21
7. STUDY POPULATION 22
7.1 Phase A 22
7.1.1. Phase A Community Level Inclusion Criteria 22
7.1.2. Phase A Community Level Exclusion Criteria 22
7.2 Phase B 22
7.2.1. Phase B Community Level Inclusion Criteria 22
7.2.2. Phase B Community Level Exclusion Criteria 22
7.3. Individual Level Inclusion Criteria for Component Interventions 22
7.3.1. Phase A Inclusion Criteria 22
7.3.2. Phase B Individual Level Inclusion Criteria – Patient-Centered Delivery 24
7.4. Recruitment Process 25
7.4.1. Phase A 25
7.4.2. Phase B 25
8. INTERVENTIONS 27
9. STUDY PROCEDURES/EVALUATIONS 29
9.1. Randomization 29
9.1.1. Phase A Randomization 29
9.1.2. Phase B Community Randomization 29
9.2. Phase A Dynamic Prevention and Dynamic Treatment Procedures 30
9.2.1. Dynamic Prevention at Outpatient Clinics, Antenatal/Family Planning Clinics, Households, and Youth Multi-Sector Interventions 30
9.2.2. Dynamic Treatment for Mobile Populations, Alcohol Users and Hypertension 36
9.2.3. Phase A Optimization 41
9.3. Phase B Intervention 41
9.3.1 Community-Enhanced Reach 42

9.3.2 Person-Centered Delivery 45
9.3.3 Overview: Data Enhanced Community Precision Health 46
9.4. Phase A Measurements 47
9.4.1. Dynamic Prevention 47
9.4.2. Dynamic Treatment 48
9.4.3. Additional controls 49
9.5. Phase B Measurements 50
9.5.1 Phase B endpoint measures and evaluation 50
9.5.2. RE-AIM Implementation Evaluation 53
9.6. Behavioral Measures 56
9.6.1. Procedures 56
9.7. Costing Evaluations 58
9.7.1. Overview 58
9.7.2. Costing team procedures 58
9.8. Mathematical Modeling 59
9.8.1. Procedures 60
9.9. Participant Discontinuation 60
10. ASSESSMENT OF SAFETY 61
10.1. Safety Assessment Overview 61
10.2. Adverse Event Procedures and Reporting Requirements 61
11. DATA HANDLING AND RECORDKEEPING 62
11.1. Data Management Responsibilities 62
11.2. Essential/Source Documents and Access to Source Data/Documents 62
11.3. Quality Control and Quality Assurance 62
12. CLINICAL SITE MONITORING 63
13. ADMINISTRATIVE PROCEDURES 63
13.1. Regulatory Oversight 63
13.2. Study Implementation 63
13.3. ClinicalTrials.gov 64
14. HUMAN SUBJECTS PROTECTIONS 65
14.1. Institutional Review Board/Ethics Committee 65
14.2. Vulnerable Participants 65
14.2.1. Pregnant Women and Fetuses 65
14.2.2. Children 65
14.2.3. Illiterate Participants 65
14.3. Informed Consent 66
14.3.1. Informed Consent Process 66
14.3.2. Assent Process 70

14.3.3. Documentation of Informed Consent 70
14.3.4. Waiver of Informed Consent 70
14.3.5. Stored Samples and Associated Data Considerations 71
14.4. Risks 71
14.5. Social Impact Events 71
14.6. Benefits 72
14.7. Compensation 72
14.8. Participant Privacy and Confidentiality 72
14.9. Study Discontinuation 73
14.10. Community Advisory Board and Other Relevant Stakeholders 73
15. STATISTICAL ANALYSIS 74
15.1 Overview 74
15.2 Individually randomized trials in Phase A 74
15.2.1 Power calculations for individual-level trials 75
15.3 Cluster randomized trials for Phase A 77
15.3.1 Power calculations for the cluster-level trial 78
15.4 Additional analyses to optimize dynamic prevention and treatment 78
15.5 Community randomized trial in Phase B 79
15.5.1 Power calculations for Phase B 80
15.6 Additional explanatory and descriptive analyses for Phase B 80
16. PUBLICATION POLICY 81
17. REFERENCES 82
18. APPENDIX Extension of Dynamic Prevention Studies and Procedures for measuring implementation on Cabotegravir Injectable Suspension (CAB-LA) 93

1. KEY INVESTIGATOR ROLES

Diane Havlir, MD
Chair, University of California, San Francisco
Address: UCSF Box 0874, San Francisco, CA 94143-0874, U.S.A.
Phone Number: +1-415-476-4082 ext. 424
Email: diane.havlir@ucsf.edu

Moses R. Kamya, MBChB, MMed, PhD
Co-Chair, Makerere University
Address: Department of Medicine, P.O. Box 7072, Kampala, Uganda
Phone Number: +256-414-533200
Email: mkamya@infocom.co.ug

Maya L. Petersen, MD, PhD
Co-Chair, University of California, Berkeley
Address: School of Public Health, 5402 Berkeley Way West, Berkeley, CA 94720-7358, U.S.A.
Phone Number: +1-510-642-0563
Email: mayaliv@berkeley.edu

Gabriel Chamie, MD, MPH
Vice-Chair, University of California, San Francisco
Address: UCSF Box 0874, San Francisco, CA 94143-0874, U.S.A.
Phone Number: +1-415-476-4082 ext. 445
Email: gabriel.chamie@ucsf.edu

Laura Balzer, PhD
Investigator and Protocol Statistician, University of California, Berkeley
Address: School of Public Health, 5317 Berkeley Way West, Berkeley, CA 94720-7358, U.S.A.
Phone Number: +1-203-558-3804
Email: laura.balzer@berkeley.edu

Andrew Phillips, MSc, PhD
Investigator, Modeling, University College London, London
Address: Institute for Global Health, Glower Street, WC1 6BT, U.K.
Phone Number: +20-7830-2886
Email: andrew.phillips@ucl.ac.uk

Florence Mwangwa, MBChB, MPH
Investigator, Adult and Adolescents Studies, Infectious Diseases Research Collaboration
Address: P.O. Box 4745, Kampala, Uganda
Phone Number: +256-779-386616
Email: fmwangwa@idrc-uganda.org

Jane Kabami, PhDc, MPH
Investigator, Infectious Diseases Research Collaboration
Address: P.O. Box 7475, Kampala, Uganda
Phone Number: +256-706-315810
Email: jkabami@idrc-uganda.org

Elijah Kakande, MBChB
Investigator, Infectious Diseases Research Collaboration
Address: P.O. Box 4745, Kampala, Uganda

Phone Number: +256-779-386616
Email: rkakande@idrc-uganda.org

Asiphas Owaraganise, MBChB
Investigator, Infectious Diseases Research Collaboration
Address: P.O. Box 4745, Kampala, Uganda
Email: asiphas@gmail.com

Elizabeth Bukusi, MBChB, M.Med, MPH, PhD
Investigator, Youth and Integration of HIV and Reproductive Health Services, Kenya Medical Research Institute
Address: Box 54840, Nairobi, Kenya
Phone Number: +254-733-617503
Email: ebukusi@rctp.or.ke

James Ayieko, MBChB, MPH, PhD
Investigator, Adult and Adolescent Studies, Kenya Medical Research Institute
Address: P.O. Box 517-20107, Njoro, Kenya
Phone Number: +254-720-925262
Email: jimayieko@gmail.com

Norton Sang, MA
Investigator, Kenya Medical Research Institute
Address: P.O. Box 35-4132, Mega City, Kisumu, Kenya
Phone Number: +254-715-778930
Email: nortonsang@gmail.com

Marilyn Nyabuti, MBChB
Investigator, Kenya Medical Research Institute and the University of Washington, Seattle
Address: P.O. Box 35-4132, Mega City, Kisumu, Kenya
Phone Number: +254-715-778930
Email: mnyanduko@gmail.com

Judith Hahn, PhD, MA
Investigator, Alcohol Use Studies, University of California, San Francisco
Address: UCSF Box 1224, San Francisco, CA 94158
Phone: +1-415-476-5815
Email: judy.hahn@ucsf.edu

Carol Camlin, PhD, MPH
Investigator, Social Sciences/Qualitative Studies, University of California, San Francisco
Address: Center for AIDS Prevention Studies, San Francisco, CA 94143, U.S.A.
Phone Number: +1-415-597-4998
Email: carol.camlin@ucsf.edu

Matthew Hickey, MD
Investigator, Hypertension Studies, University of California, San Francisco
Address: UCSF Box 0874, San Francisco, CA 94143-0874, U.S.A.
Phone Number: +1-628-206-8494
Email: matt.hickey@ucsf.edu

Starley Shade, PhD
Investigator, Cost Effectiveness, University of California, San Francisco
Address: Box 0886, San Francisco, CA 94143-0886, U.S.A.
Phone Number: +1-415-476-6362
Email: starley.shade@ucsf.edu

Harsha Thirumurthy, PhD
Investigator, Behavioral Economics Studies, University of Pennsylvania
Address: Department of Medical Ethics and Health Policy, Philadelphia, PA 19104
Phone Number: +1-215-746-0410
Email: hthirumu@upenn.edu

Edwin Charlebois, PhD, MPH
Investigator, University of California, San Francisco
Address: UCSF Box 0874, San Francisco, CA 94143-0874, U.S.A
Phone Number: +1-415-476-6241
Email: edwin.charlebois@ucsf.edu

Priscilla Hsue, MD
Investigator, Cardiovascular Studies, University of California, San Francisco
Address: UCSF Box 0846, San Francisco, CA 94110
Phone: +1+415-206-8257
Email: Priscilla.hsue@ucsf.edu

Theodore Ruel, MD
Investigator, HIV Adolescent and Young Adult Studies, University of California, San Francisco
Address: UCSF Box 0434, San Francisco, CA 94143-0136, U.S.A.
Phone Number: +1-415-476-9197
Email: theodore.ruel@ucsf.edu

Craig Cohen, MD, MPH
Investigator, University of California, San Francisco
Address: Reproductive Sciences, Box 1224., San Francisco, CA 94158 - youth and integration of HIV and reproductive health services
Phone Number: +1-415-476-5874
Email: craig.cohen@ucsf.edu

Eric Goosby, MD
Investigator, University of California, San Francisco
Address: UCSF Box 0874, San Francisco, CA 94143-0874, U.S.A.
Phone Number: +1-415-476-5483
Email: eric.goosby@ucsf.edu

Catherine Koss, MD
Investigator, University of California, San Francisco
Address: UCSF Box 0874, San Francisco, CA 94143-0874, U.S.A.
Phone Number: +1-415-476-4082
Email: catherine.koss@ucsf.edu

Urvi M Parikh, PhD
Virologist, University of Pittsburgh, Pittsburgh
Address: S830 Scaife Hall, 3550 Terrace Street, Pittsburgh, PA 15261

Phone Number: 412-648-3103
Email: ump3@pitt.edu

Melanie Bacon, RN, MPH
Investigator, National Institute of Allergy and Infectious Diseases, NIH
Address: 5601 Fishers Lane 9G74, Bethesda, MD 20892, U.S.A.
Phone Number: +1-240-627-3215
Email: mbacon@niaid.nih.gov

2. LIST OF ABBREVIATIONS

AIDS: Acquired immunodeficiency syndrome
ART: Antiretroviral therapy
CAB-LA: Long-acting cabotegravir
CE: Cost-effectiveness
CHR: Committee on Human Research
CHV: Community health volunteer
CRF: Case report form
CVD: Cardiovascular disease
DALYs: Disability-adjusted life years
DM: Diabetes mellitus
DSMB: Date Safety Monitoring Board
ELA: Empowerment and Livelihood for Adolescents
FTE: Full-time equivalent
FGD: Focus group discussion
GPS: Global positioning system
HIV: Human immunodeficiency virus
HSV: Herpes simplex virus
HTN: Hypertension
IDI: In-depth interview
IPV: Intimate partner violence
IRB: Institutional Review Board
KEMRI: Kenya Medical Research Institute
MoH: Ministry of Health
MTCT: Mother-to-child HIV Transmission
NASCOP: National AIDS and STI Control Programme
PEP: Post-exposure prophylaxis
PEth: Phosphatidylethanol
PRECEDE: Predisposing, Reinforcing, and Enabling Constructs in Educational Diagnosis and Evaluation
PrEP: Pre-exposure prophylaxis
SAP: Statistical analysis plan
RE-AIM: Reach, Effectiveness, Adoption, Implementation and Maintenance
SEARCH: Sustainable East Africa Research in Community Health
SOMREC: Makerere University School of Medicine - Research and Ethics Committee
SOPs: Standard Operating Procedures
SSA: Sub-Saharan Africa
STI: Sexually-transmitted infections
TB: Tuberculosis
TDF/FTC: Tenofovir disoproxil fumarate/Emtricitabine
TMLE: Targeted maximum likelihood estimation
TFV: Tenofovir analyte
UCSF: University of California, San Francisco
UNCST: Uganda National Council for Science and Technology
VMMC: Voluntary medical male circumcision
VHT: Village health team. This term (Uganda) used in protocol refers to equivalent CHV (community health volunteer)

3. PROTOCOL SUMMARY

Title: A Multisectoral Strategy to Address Persistent Drivers of the HIV Epidemic in East Africa (SAPPHIRE)

Sample Size:

Phase A Pilot Randomized Studies*:* 3,200 participants (2,200 HIV-negative participants, 650 HIV-positive participants, 450 hypertensive participants

***Phase B* Community Cluster Randomized trial:** 16 communities of ~10,000 persons each

Participating Sites for Phase B: 16 communities in rural western Kenya and rural western Uganda, that are geopolitical units (i.e. parish in Uganda, or sub-location in Kenya) of ~10,000 persons each (5,000 ≥15 years) within the catchment area of government health clinics and are of sufficient distance from each other to avoid contamination.

Overall Objective: Our overall objective is to determine to reduce HIV incidence and to improve community health with multi-sector, scalable interventions.

Primary Objective: Reduce HIV incidence using innovative strategies for HIV prevention and treatment to simultaneously reach “persistent driver” populations.

Secondary Objectives:

1. Evaluate and optimize individual intervention component effects, alone (Phase A) and in combination (Phase B), versus control conditions on biomedical prevention coverage and HIV viral suppression
2. Assess the effect of the intervention package on other health outcomes (mortality, tuberculosis, hypertension control, heavy alcohol use and mother-to-child HIV transmission, and pediatric immunization coverage) in Phase B
3. Evaluate behavioral and other mechanistic pathways for intervention effects on proximal mediators of HIV incidence in Phase B
4. Assess the reach, effectiveness, patient and provider adoption, fidelity and maintenance of intervention components in Phase B
5. Use final study data to inform a strategic and sustainable investment model that maximally reduces HIV incidence and improves community health for the combination interventions tested in Phase B

Study Design: This study will consist of 2 phases:

- Phase A, in which randomized trials and pilot studies will assess effectiveness, fidelity and cost and improve context-specific “fit” of prevention and treatment interventions. Combining effectiveness with implementation and costing, Phase A will optimize intervention packages with context specific adaptations in structured consultation with stakeholders.
- Phase B, which will evaluate the effects of these optimized dynamic prevention and treatment packages, on prevention coverage, population-level suppression, and HIV incidence, as well as other health outcomes, in a community randomized trial design.

Interventions in Phase A:

- Dynamic Prevention: We are evaluating ways to optimize dynamic prevention delivery with the following interventions: Provision of Dynamic Prevention intervention at 1) outpatient health centers, 2) antenatal/family planning clinics, 3) youth multisector events and 4) via village health workers interacting at households.

- Dynamic Treatment: We are evaluating services to improve HIV and hypertension care in difficult to reach populations: mobile population services; alcohol use intervention for heavy alcohol users, and hypertension interventions.

Study Schema

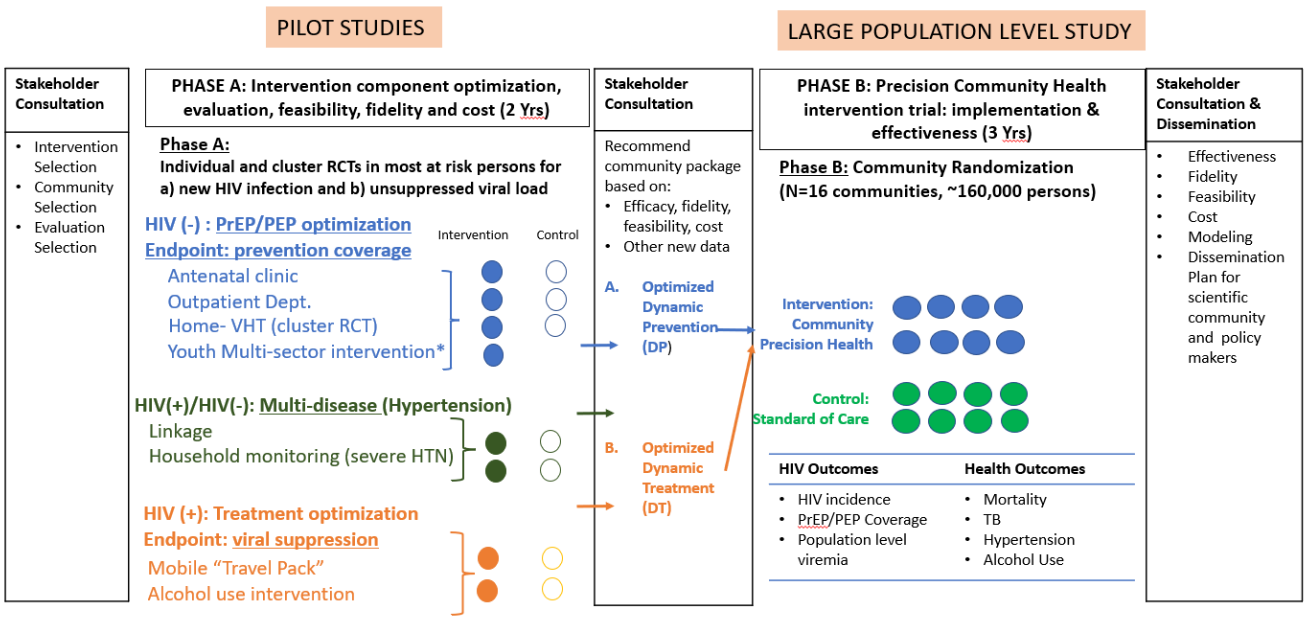

*Pilot single arm study of dynamic prevention in settings of youth clubs, boda-boda drivers

Study Endpoints:

Phase A: The primary endpoints in Phase A are: Prevention coverage (uptake and persistence of PrEP or PEP) and viral suppression (HIV RNA <400 c/mL). For hypertension intervention, the primary endpoints are linkage to care and hypertension control.

Phase B: The primary and secondary endpoints in Phase B are:

- HIV incidence
- Prevention coverage (uptake and persistence of PrEP and PEP)
- Viral suppression (HIV RNA <400 c/mL)
- Mortality
- Tuberculosis (annual incidence rate)
- Hypertension control (<140/90 mmHg)
- Heavy alcohol use
- Mother-to-child HIV transmission (infant HIV free survival at)
- Pediatric immunization coverage

Sample Size and Power, Phase A

Dynamic Prevention: Assuming a standard deviation in covered time of 35% (based on SEARCH), a two-sided hypothesis test (α=0.05), and conservatively ignoring precision gains from stratified randomization and covariate adjustment, a trial with 200 evaluable persons per arm will have 80% power to detect an approximately 10% absolute effect size (corresponding, for example, to an increase in covered time from 30% to 40%). For primary endpoint measurement for the trials at clinics, we will thus enroll up to 300 participants per arm meeting PrEP/PEP eligibility criteria (1,200 total). We also enroll up to 300 participants per arm from 16 areas serviced by village

health workers (total N=600 HIV-negative persons). For the youth intervention single arm pilots, we will enroll up to 100 persons/pilot-site to examine feasibility in primary analyses and prevention coverage in secondary analyses.

Dynamic Treatment: Assuming approximately 40% suppression (or 50% linkage in the hypertension trial) in the control arm and a two-sided hypothesis test (α=0.05), a trial of 100 persons per arm will be powered at 80% to detect a 20% increase in suppression (or linkage in the hypertension trial). For the alcohol intervention trial, we will enroll up to 200 participants living with HIV per arm, for the mobility intervention trial we will enroll 125 participants living with HIV per arm, and for the hypertension linkage intervention we will enroll 125 participants per arm. For the hypertension treatment trial, we will enroll up to 200 participants (100/arm) based on assuming 40% control in the non-intervention group for 80% power to detect an 20% or greater increase in control.

Sample Size and Power, Phase B:
With 16 communities (8/arm) each with ~5000 adults, 10% HIV prevalence, and 90% testing coverage, we anticipate being powered at 80% to detect a 40% relative reduction in annual HIV incidence (primary endpoint) from 0.5% in the standard-of-care (control) with a two-sided hypothesis test (α=0.05) and a design effect of 1.3. These power calculations were conducted with the *inctools R* package and under recency assay assumptions informed by recent validation studies: mean duration of recent infection (MDRI)=238 and false recent rate (FRR)=0%.[1-3]

4. INTRODUCTION

4.1. Background

HIV incidence in Sub-Saharan Africa (SSA) remains well above global elimination targets.[4] Despite an increasingly large, evidence-based toolkit to prevent HIV transmission, there were over 1 million new HIV infections in SSA in 2018.[4] We know from SEARCH[5] and three other recent SSA population-level studies[6-8] that reaching (and exceeding) *the UNAIDS 2020 population-level viral suppression target of 73%*[9] *is insufficient to achieve epidemic control* (**Table 1**). We also know that key sub-populations, in particular youth,[7, 10-13] men,[5, 7, 13-15] mobile populations,[16-18] and persons with heavy alcohol use[19, 20] continue to fall short of viral suppression targets, putting them at higher risk for HIV complications, mortality, and onward HIV transmission. Finally, pre-exposure prophylaxis (PrEP), an intervention that has reduced incidence in affected populations in some areas where high rates of viral suppression were already present,[21, 22] has not been effectively deployed in high risk populations (such as young women) in SSA.[23, 24] To achieve control of the HIV epidemic and mitigate its consequences, we need **new and scalable** interventions to improve delivery of prevention and treatment to **vital segments of the population that contribute to persistence of new infections.** We describe below why previous approaches that were effective for the majority of people in the ART roll-out do not work for subsets of these "persistent-driver" populations, our proposed solutions, and how a multi-component intervention trial can effectively inform stakeholders on programming and resource allocation to accelerate reductions in HIV incidence.

Barriers to effective HIV prevention and treatment outcomes among key, persistent-driver populations fall into three major themes based on epidemiologic and qualitative data from SEARCH on seroconversions (N=704), PrEP utilization (3,489 starts[25]), viral suppression sub-group analyses (N=13,529[5, 13, 19]), and other published literature. First, the majority of new infections are in youth, and in this growing, at-risk population, social and economic concerns often far outweigh health concerns on a day-to-day basis (individual barriers).[26-29] *There has been insufficient recognition that at any given moment in time, health is not a preeminent concern, particularly for youth.* Indeed, poverty itself may increase "present bias" – the tendency for short term considerations to outweigh long term ones – contributing to decision-making that harms health.[30-33] Second and third, lack of flexibility in how prevention and treatment services, respectively, have been offered to people whose needs and behavior vary from mainstream community members (e.g. mobile populations, drinkers) and may be continually changing (systems barriers) have impeded uptake and impact of these services.[34-38]

Modelling and phylogenetic studies suggest that further progress will require simultaneous and coordinated approaches in HIV treatment and prevention among persistent driver populations. Individual, "narrow" focused interventions (PrEP) for adolescent girls are important, but alone will not reach HIV elimination levels. Persistent driver populations are minority subsets of demographic populations of considerable size (e.g. while men are at elevated risk of non-suppression, most HIV-infected men are suppressed); some of the barriers they face (e.g. inflexible health systems) are shared across the demographic groups and can be integrated into a personalized public health approach that combines i) foundational changes in health care delivery systems that are scalable and affordable (e.g., re-structuring treatment visits for youth to prioritize non-health concerns) with ii) the option to access more intensive services for the targeted group of persons for whom basic one-size-fits all approaches are insufficient (e.g. intensified alcohol counseling for heavy drinkers and "travel packs" for mobile populations). Assumptions that strategies to reach these populations will be prohibitively expensive or too complex for programs to implement are unsubstantiated, and stakeholders need well-designed studies incorporating multiple interventions for persistent driver populations, and integrating intensification based on choice, to understand what works and inform resource allocation as they move beyond the "90-90-90" era towards HIV elimination.

Table 1. Comparison of population-level, Universal Test and Treat (UTT) Trials in Sub-Saharan Africa.

Trial	BCPP/Ya Tsie[8]		HPTN 071 (PopART)[7]			SEARCH[5]		ANRS 12249 (TasP)[6]	
**Country**	Botswana		South Africa/Zambia			Kenya/Uganda		South Africa	
**Prevalence**	29%		22%			4-19%		30%	
**Arm**	C	I	C	I Arm A	I Arm B	C	I	C	I
**Universal testing**	—	✓ Home, mobile	—	✓ Home + field (men, youth)	✓ Home + field (men, youth)	✓ Multi-dz Fairs/ Home	✓ Multi-dz Fairs/ Home	✓ Home	✓ Home
**Testing frequency**		Baselin e; ongoing targeted		Ongoing Annual	Ongoing ~Annual	Baseline	Annual	6 monthly	6 monthly
**Enhanced linkage**		✓		✓	✓		✓		✓
**Rapid ART Start**		✓ (from 2016)					✓		
**Universal Treatment**	✓ (from 2016)	✓ (from 2016)	✓ (from 2016)	✓	✓ (from 2016)	✓ (from 2016)	✓		✓
**Differentiated ART Delivery**				✓ (Zambia†)	✓ (Zambia†)		✓‡		
**Population viral Suppression**									
- at start	75%	70%	54%	57%	56%	42%	42%	26%	24%
- at end	83%	88%	60%	68%	75%	68%	79%	45%	46%
- difference	+8	+18	+6	+11%	+16%	+26	+37	+19	+23
**HIV Incidence**									
Annual Incidence for 100 person-years	0.92	0.59	1.55	1.06	1.45	0.27	0.25	2.27	2.11
Reduction (I vs C)	31% reduction		20% reduction			not significant, but 32% reduction in intervention arm		not significant	

			between years 1 & 3	

To address these barriers for persistent driver populations to increase prevention coverage for HIV- persons and viral suppression for HIV-infected persons, we used the empirically-validated PRECEDE model[39] where health decision-making and health-seeking behaviors can be influenced via interventions that predispose, enable, and reinforce desired engagement with prevention and treatment services (see **Section 8**, below for comprehensive description of our PRECEDE intervention framework).

Table 2. Dynamic HIV Prevention: Problems and Solutions

Prior Insight	Problem	Solution: Dynamic Prevention
PrEP can be offered as a new prevention option at health fairs	Health is not a high priority for youth and a single offering with multi-disease framework is insufficient to engage youth	Offer entry into PrEP/PEP through demand generation of "youth hubs" by integrating HIV prevention and reproductive health into existing youth development programs
PrEP can be delivered at HIV clinics	Many persons who are willing to take PrEP do not want to attend HIV clinic	Offer PrEP/PEP at antenatal, family planning and HIV clinic venues
PrEP works for motivated persons in rural areas	Coverage and persistence of PrEP (daily pill weeks prior and after exposure) is low in many at-risk persons, particularly women (less agency in sexual relations)	Offer choice of PrEP or PEP (event driven prevention) in supportive, environment where persons can go back and forth between current and new options (e.g. vaginal ring)

Dynamic HIV prevention: Our 3 Dynamic Prevention components address barriers we identified in offering PrEP to >10,000 persons with risk factors for HIV acquisition in the region (Table 2). First, we will engage youth in HIV prevention and reproductive health services by generating demand for these services within existing life skills and vocational programs (BRAC youth development clubs in Uganda and micro-finance clubs in Kenya) via integration of key health and socio-economic services. For youth, embedding HIV services within a broader, multi-sector strategy for individual and community empowerment via economic and social advancement can lead to better decision-making and also increase demand for and engagement with HIV services. Similar approaches have been used in diverse settings and may overcome present bias and other barriers faced by youth living in poverty by generating demand for services while improving future expectations and decision-making via increased opportunity.[40-47] Despite a strong evidence base that these interventions can impact self-reported behaviors, and in some cases unintended pregnancies and STIs, and recent encouraging evidence from the DREAMS program (HIV incidence reduction in non-randomized longitudinal analyses[48]), there is little rigorous evidence that these interventions can reduce HIV incidence (as noted in a systematic review of income generation interventions for HIV prevention[40]) or impact biomedical prevention (e.g. PrEP, PEP, condom use) uptake or adherence from randomized studies.

HIV prevention services that offer choice of delivery options (PrEP & PEP) and flexibility in their use and location of delivery over time allow for a dynamic, patient-centered model of prevention. This approach is responsive to the reality of an individual's changing needs, thereby facilitating continued engagement. In SEARCH, PrEP use declined over time in spite of initial enthusiasm and uptake by early adopters.[49] *In multiple settings, poor adherence to PrEP remains its Achilles' heel.*[50, 51] Rather than presenting PrEP in a similar framework to ART (i.e. lifelong use with 100% adherence for efficacy with delivery at HIV clinic), approaches that incorporate flexibility in choice and delivery location, akin to contraception options for women in developed countries, may prove more successful. Indeed, over the past four decades, increased choice in contraception and flexibility in access have been directly associated with increased use.[52, 53] Framing prevention in

Figure 1

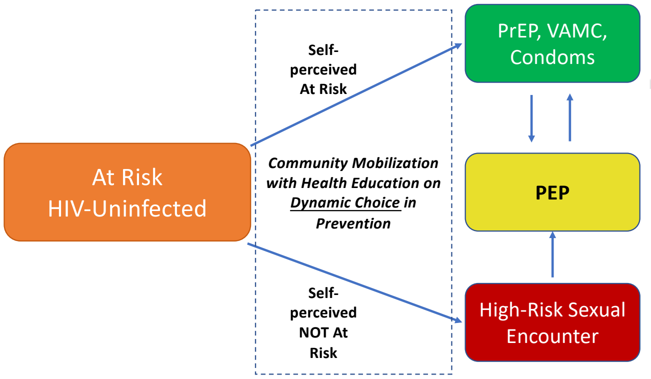

the context of healthy choices, rather than an assessment of risk, may also reduce the association of PrEP with "risky" behavior or promiscuity. Offering PEP – an event-driven option that does not require accurate prediction of risk before a potential HIV exposure (akin to a "morning after" pill), and which additionally provides protection for those with less sexual agency – is a critical innovation in flexible access to prevention services. PEP can also act as a "gateway" to PrEP and other prevention modalities (**Figure 1**), providing infrastructure to add new prevention options as they become available (e.g. the PrEP vaginal ring[54, 55]).

Dynamic HIV Treatment: offering choice and flexibility in HIV clinical care and ART access that is tailored to the needs of key populations – including youth, men, mobile, and heavy alcohol-using populations – may result in increased care engagement and viral suppression rates among these populations, compared to differentiated care models as currently conceptualized. We have identified key barriers and facilitators for engagement in treatment for each of these groups, obtained via quantitative surveys and focus group discussions among persons who have not engaged, dropped out of care or not achieved viral suppression in SEARCH and via existing literature (Table 4).[36, 56-62] Treatment outcomes are most likely to be effective when these barriers are overcome. For example in youth, using the PRECEDE framework we have piloted our proposed intervention that combines (i) a "life stage" assessment framework that addresses the many dynamic changes in relationships, school and employment present in youth (PREDISPOSING) (ii) flexible clinic access (ENABLING) and (iii) rapid viral load feedback (REINFORCING). Details on treatment interventions for other key driver populations are in the protocol (see Section 8, below).

Table 3. Dynamic HIV Treatment: Barriers and Solutions

Population	Dynamic component	Barriers	Facilitator
Youth	Life stage changes	Competing priorities, mobility, boarding school, lack of support	Provider engagement in life stage priorities, flexible clinic hours and types of visits (e.g. mobile phone, early/late and weekend hours)
Men	Work demands year round	Competing responsibilities, long clinic wait times, provider attitudes, mistrust in health system, stigma, social isolation	Flexible clinic hours & rapid visits, welcoming environment & social events, offering of men's sexual health services, integrated chronic disease (vs HIV only) care model
Mobile populations	Location	Unpredictable work schedule, enrollment entry into local health system	Visits and refills for ART outside routine schedules, easy entry to establish care at new location and to transfer to outside location
Heavy alcohol use	Alcohol intoxication	Clinic visit adherence, ART adherence	Structured alcohol counseling, "shame reduction" strategies, flexible visits and refills

Community Precision Health uses data systems and analytics to extend the reach of the health care facility into the community more effectively and more efficiently. Currently, CHVs/VHTs collect a rich quantity of data using paper records. Under the current system, however, i) entry of CHV/VHT-collected data into facility-based data systems places substantial burden on CHVs/VHTs to travel to the facility for reporting; ii) data capture may be incomplete and occur after substantial time delay, undermining its utility to guide outreach activities; and, iii) no direct data flow exists from health facilities back to VHTs/CHWs, limiting their ability to provide defaulter and linkage support to persons in need. Community Precision Health addresses these barriers by providing CHVs/VHTs and workers at outreach activities with a mobile device-based app to facilitate data capture and electronic reporting. This Community Precision Health system, which includes two-way data flow between existing country Electronic Medical Record (EMR) systems and the app, is designed to improve the ability of CHVs/VHTs to support the care cascade by providing enhanced and timely notification of persons failing to link to care or defaulting. The data system further generates a dynamic population-based database of community health outcomes, enabling the use of analytics (such as machine-learning based risk segmentation) to identify persons and local regions at higher risk of poor health outcomes (eg, risk of HIV infection, lack of viral suppression, or uncontrolled HTN) and thus providing the basis for tailoring of enhanced outreach, screening, and service referrals.

The HIV epidemic will unnecessarily claim and continue to adversely affect millions of lives unless we deploy innovative strategies that extend the gains beyond those of universal testing and ART. Universal ART must be followed by dynamic prevention and treatment

paradigms that allow for heterogeneity in patient need and preference, as well as changing preferences over time – while targeting interventions to those at greatest risk of incident and uncontrolled HIV. Furthermore, **HIV cannot be addressed in a vacuum and must be coupled with health services that are seen by community members as a priority -** including evaluation and treatment for diseases such as hypertension,[63-65] diabetes,[66] and heavy alcohol use[67], delivered in an environment that harnesses human capital among youth (the largest segment of the population in SSA[68]). In our proposal, we postulate that within this broader framework, we can successfully reduce HIV incidence, population viral load and mortality, while improving community health outcomes. We will rigorously evaluate these effects and their costs using a 2-stage optimization and factorial cluster randomized design.

4.2. Study Hypothesis for Primary Objective

Each of the Phase A studies will test a hypothesis related to the intervention:

- Dynamic Choice Prevention delivered to women seen at antenatal clinics (ANC) will improve biomedical covered time, defined as the proportion of follow-up time that a participant is protected from HIV with either PrEP or PEP.
- Dynamic Choice Prevention delivered to men and women seen at the outpatient department (OPD) will improve biomedical covered time, defined as above.
- Dynamic Choice Prevention delivered to men and women seen in the community by village health teams (VHTs) will improve biomedical covered time, defined as above.
- Dynamic Choice Treatment delivered to mobile persons with HIV will improve HIV viral suppression at 48 weeks.
- The Healthy Living Intervention delivered to persons with HIV and heavy alcohol use will improve HIV viral suppression at 24 weeks.
- A one-time transport voucher will increase linkage to hypertension care following community-based diagnosis.
- Community-based hypertension care will improve hypertension control among persons with severe hypertension.

The study hypothesis for the Phase B population level study is: A Precision Community Health Model leveraging existing facility-based outreach and community health workers, enabled by dynamic choice prevention/treatment multi-disease approaches and interactive data systems –will reduce HIV infections, deaths and improve health.

5. OBJECTIVES

5.1. Primary Objective

To reduce HIV incidence using innovative strategies for HIV prevention and treatment to simultaneously reach “persistent driver” populations.

5.2. Secondary Objectives

1. Evaluate and optimize individual intervention component effects, alone (Phase A) and in combination (Phase B), versus control conditions on biomedical prevention coverage and HIV viral suppression.
2. Assess the effect of the intervention package on other health outcomes (mortality, tuberculosis, hypertension control, heavy alcohol use, mother-to-child HIV transmission and pediatric immunization coverage) in Phase B.
3. Evaluate behavioral and other mechanistic pathways for intervention effects on proximal mediators of HIV incidence in Phase B.
4. Assess the reach, effectiveness, patient and provider adoption, fidelity and maintenance of intervention components in Phase B.
5. Use final study data to inform a strategic and sustainable investment model that maximally reduces HIV incidence and improves community health for the combination interventions tested in Phase B.

6. STUDY DESIGN

In the "optimization" Phase A, the study will conduct randomized trials and pilot studies to assess effectiveness, fidelity and cost and improve context-specific "fit" of prevention and treatment interventions. Combining effectiveness with implementation, costing and modelling outcomes, the study will optimize intervention packages with context-specific adaptations in structured consultation with stakeholders. In the "evaluation" Phase B, the study will evaluate the effects of Dynamic Prevention and Dynamic Treatment intervention packages based on data from Phase A and new advances from outside the study, alone and in combination, on HIV incidence, biomedical prevention coverage and other health outcomes, in a pair-matched community randomized design.

Study Schema

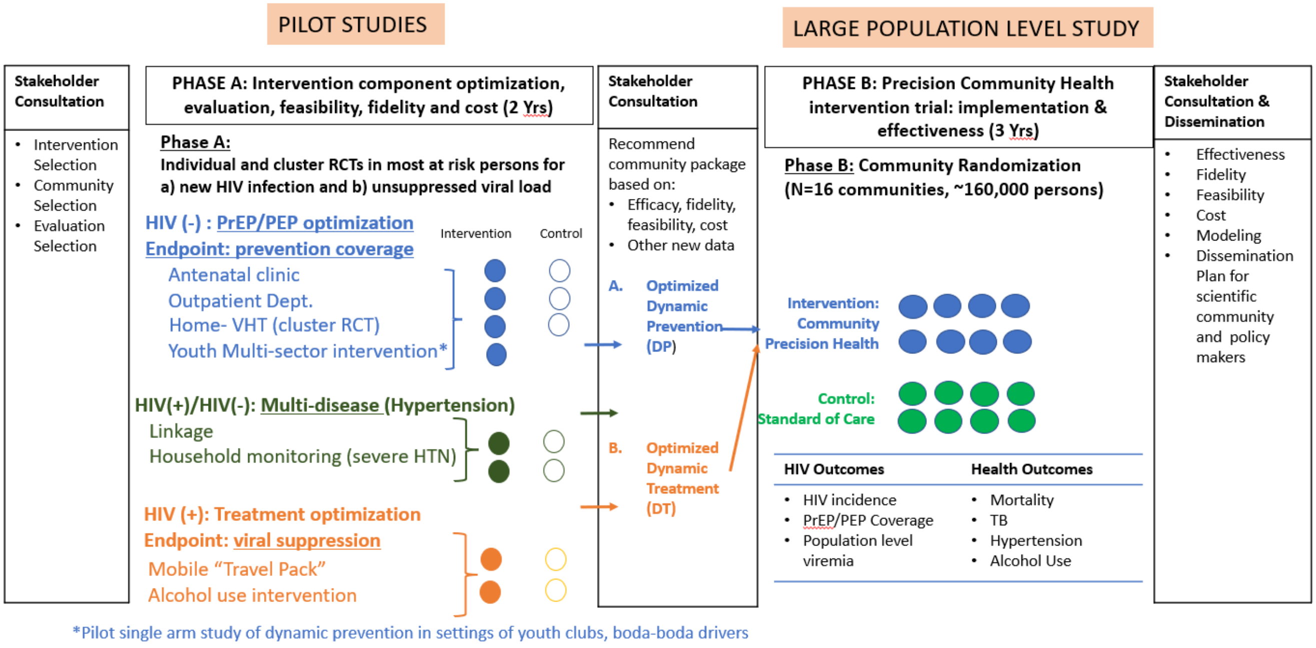

We will evaluate population-level intervention implementation outcomes guided by the RE-AIM framework,[69] elucidate mechanisms of action and component intervention effects using mixed methods and multi-level mediation analyses, and estimate incremental costs and gains using micro-costing and time-and-motion studies. Finally, the study will use the HIV Synthesis Model to model outcomes and costs of Phase B results over a period of decades to inform policy and stakeholder decisions to adopt successful strategies to achieve and sustain HIV epidemic control.

Structured stakeholder consultations are formally incorporated in the study design and leverage regular and ongoing collaborations the study team has with the HIV, non-communicable disease and general health leads in Kenya and Uganda Ministries of Health and PEPFAR implementing partners at the national and the regional level.

7. STUDY POPULATION

7.1 Phase A

The study will take place in rural Kenya and Uganda in 12 geographically dispersed communities.

7.1.1. Phase A Community Level Inclusion Criteria

1. Non-adjacent geopolitical units in western Uganda and western Kenya.
2. Most recent census population between 9,000 and 11,000 individuals.
3. Served by an ART/PrEP/PEP providing health center with antenatal and family planning services.
4. Community leader commitment for study participation and implementation.

7.1.2. Phase A Community Level Exclusion Criteria

1. Presence of ongoing community-based interventions that provide HIV prevention or treatment outside of the current in-country treatment guidelines.
2. An urban setting defined as a city with a population of 100,000 or more inhabitants.
3. Absence of a health center able to provide ART, PrEP and PEP.

7.2 Phase B

The study will take place in rural Kenya and Uganda in 16 geographically dispersed communities different from Phase A of ~10,000 persons each (5,000 persons ≥15 years).

7.2.1. Phase B Community Level Inclusion Criteria

1. Non-adjacent geopolitical units in western Uganda and western Kenya.
2. Most recent census population between 9,000 and 11,000 individuals.
3. Served by an ART/PrEP/PEP providing health center with antenatal and family planning services.
4. Community leader commitment for study participation and implementation.

7.2.2. Phase B Community Level Exclusion Criteria

1. Presence of ongoing community-based interventions that provide HIV prevention or treatment outside of the current in-country treatment guidelines.
2. An urban setting defined as a city with a population of 100,000 or more inhabitants.
3. Absence of a health center able to provide ART, PrEP and PEP.
4. Community that took part in Phase A.

7.3. Individual Level Inclusion Criteria for Component Interventions

7.3.1. Phase A Inclusion Criteria

Study Population	Inclusion Criteria	Exclusion Criteria
**Dynamic Prevention**	<u>Outpatient and antenatal/FP clinics</u><br>1. Age ≥15 years<br>2. HIV-negative<br>3. Current or anticipated sexual activity with partner(s) with known HIV-infection or unknown HIV status<br><br><u>VHT households</u><br>1. Age ≥15 years<br>2. HIV-negative<br>3. Current or anticipated sexual activity with partner(s) with known HIV-infection or unknown HIV status<br><br><u>Youth multi-sector interventions</u><br>1. Age ≥15 to ≤25 years<br>2. HIV-negative<br>3. Current or anticipated sexual activity with partner(s) with known HIV-infection or unknown HIV status<br>4. At least one of the following risk factors: School drop-out, prior pregnancy, transactional sex, orphanhood, work in sector associated with higher HIV risk (e.g. boda boda driver), drink alcohol or STI history	<u>Outpatient and antenatal/FP clinics</u><br>1. <15 years of age<br>2. Unable to provide consent or parental co-consent as per country guidelines<br>3. Participation in another Phase A RCT intervention component<br><br><u>VHT households</u><br>1 <15 years of age<br>2 Unable to provide consent or parental co-consent as per country guidelines<br>3 Participation in another Phase A RCT intervention component<br><br><u>Youth multi-sector interventions</u><br>1. <15 or >25 years of age<br>2. Unable to provide consent or parental co-consent as per country guidelines<br>3. Participation in another Phase A RCT intervention component
**Dynamic Treatment**	<u>Mobile Travel Pack intervention</u><br>1. Age ≥15 years<br>2. HIV-positive<br>3. HIV RNA non-suppression (>400 c/mL in the prior 12 months) or missed 2 visits in past 12 months or no VL measured in past 12 months<br>4. Travel outside the community ≥2 times in past 12 months<br>5. Enrolled or new to care in a study clinic<br><br><u>Alcohol intervention</u><br>1. Age ≥18 years<br>2. HIV-positive<br>3. HIV RNA non-suppression (>400 c/mL in the prior 12 months) or missed clinic visits (>2 weeks or <=90 days from last scheduled clinic visit) within past 6 months, or out of care without return to care (>90 days from last scheduled clinic visit) within past 6 months<br>4. Heavy alcohol use per AUDIT-C tool (scores of ≥4 for men, and ≥3 for women)<br>5. Enrolled or new to care in a study clinic<br><br><u>Hypertension Linkage and Treatment Interventions</u>	<u>Mobile Travel Pack intervention</u><br>1. <15 years of age<br>2. Participation in another Phase A RCT intervention component<br><br><u>Alcohol intervention</u><br>1. <18 years of age<br>2. Participation in another Phase A RCT intervention component<br>3. No access to mobile phone<br><br><u>Hypertension Linkage and Treatment Interventions</u>

	1. Age ≥25 years (linkage); age ≥40 years (treatment)<br>2. Blood pressure ≥140/90 mmHg on three repeated measurements during community-based hypertension screening for linkage study and ≥160/100 mmHg for the treatment study<br>3. Resident (by self-report) within catchment area of referral health center	1. <25 years (linkage); age <40 years (treatment)<br>2. Plan to out-migrate from the catchment area of referral health center within 30 days of screening visit<br>3. Already engaged in hypertensive care (by self-report) (linkage only)<br>4. Blood pressure measure of ≥180/110 mmHg during screening and symptoms of hypertensive emergency (linkage only)<br>5. Pregnant (treatment)<br>6. Co-morbidities that preclude home monitoring (treatment)

7.3.2. Phase B Individual Level Inclusion Criteria – Patient-Centered Delivery

Study Population	Inclusion Criteria	Exclusion Criteria
**Dynamic Prevention**	Antenatal clinics, outpatient clinics and other community locations (such as bars and youth events)<br>1. Age ≥15 years<br>2. HIV-negative<br>3. Current or anticipated risk of HIV-infection	Antenatal clinics, outpatient clinics and other community locations (such as bars and youth events)<br>1. <15 years of age<br>2. Unable to provide consent or parental co-consent as per country guidelines
**Other Patient-Centered Delivery Interventions (Dynamic Treatment)**	Life Events Assessment and Plan (LEAP) Intervention<br>1. HIV-positive<br>2. One or more of the following:<br>• Age ≥15 to ≤24 years<br>• Pregnant<br>• Breast-feeding<br>• HIV RNA non-suppression (≥400 c/mL in the prior 12 months, no HIV RNA in the last year, or diagnosed with HIV in last 4 weeks) at initiation or time of treatment failure<br><br>Youth defaulters re-engagement by VHT/CHVs<br>1. Age ≥15 to ≤24 years<br>2. Missed ≥ 2 ART treatment or HIV prevention visits<br><br>Severe Hypertension Treatment/Telehealth Intervention<br>1. Age ≥40 years<br>2. Blood pressure ≥160/100 mmHg<br>3. Resident (by self-report) within catchment area of referral health center	Life Events Assessment and Plan (LEAP) Intervention<br>1. Unable to provide consent as per country guidelines<br><br>Youth defaulters re-engagement by VHT/CHVs<br>1. <15 or >24 years of age<br>2. Unable to provide consent as per country guidelines<br><br>Severe Hypertension Treatment/Telehealth Intervention<br>1. Age <40 years<br>2. Pregnancy<br>3. Plan to out-migrate from the catchment area of referral health center within 30 days of screening visit<br>4. Current hypertensive emergency (BP >180/110 mmHg AND signs or symptoms of hypertension emergency). Note: participants are eligible once they receive

	VHT/CHV Child TB Prevention Intervention<br>1. Age ≤5 years/<br>2. At least one member of participant's household diagnosed with TB	emergency treatment and are clinically stable.<br>5. Co-morbidities that preclude home monitoring<br>6. Unable to provide consent as per country guidelines<br><br>VHT/CHV Child TB Prevention Intervention<br>1. Age >5 years

7.4. Recruitment Process

7.4.1. Phase A

Dynamic Prevention

Outpatient, Antenatal/Family Planning Clinics, Households, and Youth Multi-Sector Interventions: The study will enroll persons currently active in, or willing to receive care or take part, in 1) outpatient clinics, 2) antenatal/family planning clinics, 3) community households (VHT study) and 4) Youth multi-sector interventions. The youth multisector interventions are tailored to respond to COVID pandemic restrictions. Study leadership will meet with clinic, VHTs, and community leaders to explain the study purpose and goals. In collaboration, study staff will approach consecutive potential participants for participation in the study.

Dynamic Treatment

Heavy alcohol drinkers, mobile populations and those with untreated or severe hypertension: The study will offer Dynamic Treatment intervention components to 2 different populations with unsuppressed viral loads and/or missed visits: 1) mobile populations (travel outside community at least twice in past 12 months), 2) heavy alcohol users (AUDIT-C screening tool scores of ≥4 for men, and ≥3 for women[70, 71]). For the alcohol trial, staff will recruit from the clinic, and use bar-based mobilization, in which staff will reach out to persons attending bars in study communities and offer referral for multi-disease (e.g., hypertension, diabetes and HIV) screening at each community clinic, with additional screening for trial eligibility among adults identified with HIV. The study will also examine one intervention to promote linkage to hypertension care among persons (age ≥25), regardless of HIV status, who have elevated blood pressure (≥140/90 mmHg) identified during community-based hypertension screening and a second study to evaluate an intensive community-based hypertension treatment intervention vs standard clinic monitoring for those with severe hypertension (≥160/100) Study leadership will meet with clinic directors in the communities to explain the study purpose and goals. In collaboration with study clinic leadership, study staff will approach consecutive potential participants for participation in the study.

7.4.2. Phase B

Dynamic Prevention

HIV, Outpatient, Antenatal Health Clinics. The study will offer Dynamic Prevention to persons currently enrolled, or willing to enroll, in study community settings including HIV/PrEP clinics, family planning clinics, antenatal clinics and youth clinics. Participants will be referred to these programs from Community Mobilization activities described in Section 9.3.1, and persons who self-present to

community clinics for HIV prevention services (i.e. testing, VMMC, PrEP, PEP, condom use) or STI screening. The study will further expand uptake by including referrals from mobile outreach events, social network contacts of newly diagnosed PLHIV, PrEP and PEP users, and at youth multi-sector locations. Baseline and ongoing community sensitization for PrEP/PEP availability will be conducted.

Dynamic Treatment

Youth, Mobile Populations, Heavy Alcohol Users and Severe HTN :. A multi-faceted approach will be used to recruit participants. HIV-positive youth may be referred from youth multi-sector activities, community mobilization activities, or through recruitment at the study clinic. HIV-positive persons may be referred from community mobilization activities and social network contacts of persons already in care, as well as directly from the clinic. HIV-positive men and women over 18 years of age enrolled in care in the study clinics will be screened for alcohol use via the AUDIT-C screening tool.[70, 71] Persons with severe HTN will be referred from clinic, mobilization community events and from VHT routine HTN screening.

8. INTERVENTIONS

Prior to the implementation of intervention activities in Phase A and Phase B, the study will utilize stakeholder consultation to advise on community selection and intervention design, and, during the optimization period of Phase A, determination of which interventions piloted in the initial clinics will be implemented and expanded in Phase B.

The study's intervention components follow the PRECEDE model, based on social, cognitive and network theory that predispose, enable, and reinforce desired engagement with prevention and treatment services via their effect on health decision-making and health-seeking behaviors.[39] A description of each intervention component as it relates to the PRECEDE framework is provided in Table 4.

Table 4. PRECEDE Framework for Intervention Components

Intervention Component	PRECEDE Framework Description	Published & Unpublished Evidence supporting/ informing intervention components in PRECEDE Framework
Dynamic Prevention	<u>Dynamic Prevention in Youth Multi-Sector Interventions</u><br>· Youth Mobilization (*Predisposing*)<br>· Empowerment via Vocational & Life Skills Training (Uganda) & Micro-finance (Kenya) (*Enabling*)<br>· Integrated HIV, Reproductive Health and Alcohol Use Education (*Predisposing*)<br>· On-site HIV testing & expedited ART/PrEP/PEP referral (*Enabling*)<br>· Social environment with local mentor (*Reinforcing*)<br>· Income-generating activities (*Reinforcing*)	<u>Dynamic Prevention at Youth Multi-Sector Interventions</u><br>· SEARCH with prior success with population-wide adolescent & young adult mobilization[72]<br>· Ongoing pilot study of Youth Hubs for young women and men with integrated HIV and reproductive health services (SEARCH/BRAC).<br>· Prior evidence that vocational/life-skills training,[44, 73] micro-finance[74, 75] and social support[43, 76] can have positive impacts on sexual risk behavior and decreased teen pregnancy, early marriage and non-consensual sex in youth<br>· Feasibility of Youth Hub testing & mobile testing[77] with referral to ART, PrEP and PEP in SEARCH
	<u>Dynamic Prevention at Clinics</u><br>· Community Mobilization (*Predisposing*)<br>· Flexible access to PrEP/PEP with mobile phone access line (*Enabling*)<br>· Structured Assessment of barriers to PrEP/PEP start and adherence, with personalized plans developed in response (*Enabling*)<br>· Dynamic transitions to/from PEP & PrEP (*Reinforcing*)<br>· Psychologic support for traumatic experiences (*Enabling*)<br>· Social Network Outreach (*Predisposing*) with multi-disease screening and transport refund vouchers (*Enabling*)	<u>Dynamic Prevention at Clinics</u><br>· Prior experience with community mobilization for population-level offer of PrEP[25]<br>· Distance from clinic a barrier to PrEP in SEARCH[78] and community delivery associated with increased PrEP retention[79]<br>· SEARCH with prior experience assessing individual level barriers to PrEP during population-wide offer of PrEP,[80-82] with potential for actionable, personalized plans<br>· In a survey of 48 PrEP recipients in SEARCH, 80% felt that adding PEP would enhance prevention "coverage."<br>· Choice in & flexible access to contraception has been associated with increased use;[52, 53] and our SEARCH PEP pilot demonstrated high uptake and adherence to PEP and feasibility referral to PrEP<br>· SEARCH has pilot tested feasibility of Social

		Network Outreach with multi-disease health checks among key populations, using HIV- and HIV+ index cases: 87% (133/155) of social contacts redeemed vouchers, including new HIV+ patients who started same-day ART; all HIV- contacts referred to PrEP
Dynamic Treatment	Community Mobilization (*Predisposing*)	Community Mobilization: Demonstrated impact of SEARCH universal testing and treatment intervention on social mobilization of PLHIV as advocates for other PLHIV[83, 84]
	Tailored Differentiated Care (*Enabling/Reinforcing*) for:<br><br>*Men*: Integration of sexual health, multi-disease chronic care, provider training on U=U messaging, flexible hours, welcoming environment and social events<br><br>*Mobile Populations*: Queries about travel plans at each visit, assist with in-/out-transfers, mobile phone access to clinics and mobile minutes for unexpected travel, non-standard refill schedules and out-of-facility refills, mobile population seasonal outreach;<br><br>*Heavy Drinkers*: Alcohol screening (*Predisposing)*; two in-person counseling sessions at times of regular clinic visits (*Enabling)*; monthly booster phone calls between in-person sessions (*Reinforcing*)	Tailored Differentiated Care<br><br>*Men*: SEARCH has experience integrating sexual health into HIV testing fairs to generate demand (>30,000 fair visits) and integrating HIV and multi-disease chronic care.[5] Strong evidence base identifying barriers to men's engagement in HIV clinic.[61, 85-88] Evidence of low knowledge re: U=U.[89] Prior evaluation of facilitators to retaining men in care (with high retention).[62]<br><br>*Mobile Pops*: SEARCH has experience eliciting heterogenous forms of mobility from PLHIV,[90] identifying barriers faced by mobile populations,[61] and identifying new residents during peak periods of mobility.[77] Mobile PLHIV identified mobile phone access to providers and "travel packs" for ART to cope with unanticipated travel.<br><br>*Heavy Drinkers*: Multi-session brief alcohol interventions can significantly reduce alcohol consumption[91] and increase viral suppression in PLHIV.[92] Co-I Hahn (R01 AA0240990) conducted extensive research to adapt an efficacious brief alcohol intervention,[93] similar to one associated with decreased alcohol use in India,[94, 95] to the East African context.

9. STUDY PROCEDURES/EVALUATIONS

9.1. Randomization

9.1.1. Phase A Randomization

We will randomize individuals to the active prevention or treatment intervention or to standard-of-care as shown below.

Table 5: Randomizations in Phase A

Intervention	Total number of participants
Dynamic Prevention –Individual randomization at outpatient health center	600
Dynamic Prevention – Individual randomization at antenatal clinic	600
Dynamic Prevention –Community cluster randomization of VHTs	16 communities up to 600 persons
Dynamic Prevention- No randomization, single arm pilot of youth multi-sector intervention	Up to 400
Dynamic Treatment – Individual randomization Mobile Travel Pack	200
Dynamic Treatment – Individual randomization Alcohol Intervention	400
Hypertension Linkage- Individual randomization linkage to care	200
Hypertension Treatment – Individual randomizations- clinic vs community/home monitoring	200
Total	3200

Participants will take part in only one of the eight interventions. After screening for eligibility for one of the above intervention components, participants will be randomized at the time of enrollment via computer-generated random assignment for all of the randomized trials except for the dynamic prevention household trial. For this trial, we will randomize 16 villages (with their accompanying household reach of ~ 600 at risk persons) to intervention or control (cluster randomization). The youth multi-sector interventions will be non-randomized, single-arm pilots.

The communities which will take part in the Phase A will be located in rural western Uganda and western Kenya. These are primarily farming and fishing communities and are geographically dispersed.

9.1.2. Phase B Community Randomization

The study will randomize the 16 communities (8 Ugandan and 8 Kenyan) as detailed in the Study Schema. Randomization will be stratified by country. The communities selected for randomization will not include health facilities that took part in Phase A activities.

We will utilize an established randomization strategy that is both scientifically valid and transparent to the community stakeholders and uses local idioms to make the concept of randomization easily understood by traditional leaders and community members,[96] as conducted in the SEARCH trial.[5] Community randomization will be conceptualized as an ongoing part of the community preparedness process in partnership with the community leaders and community individuals.

For the random assignment of each community, we will employ a public lottery to achieve maximum public acceptance of the randomization results by enhancing transparency and spreading ownership of the process. For each country separately, the randomization allocation will be written on separate pieces of paper or card and folded in half to obscure the label from view. The number of cards for each level will correspond with the randomization probabilities. These cards will then be placed in a sealed box with a hand hole cut out of the top of the box. For each country separately, a computer will randomly designate ordering of community sites. Then the lottery will be conducted as a series of draws according to the computer-generated ordering. The leader with the last draw will be responsible for holding and shaking the selection box. After the drawing is complete, the host country leader will announce the interventions corresponding to each allocation. The lottery will be a public event with members of the Community Advisory Board and local leadership present along with guests from other community-based organizations and the general public to witness the lottery conducted by local chiefs or leaders. This process assures equal chance of being randomized to each level of the intervention and eliminates any residual fears of bias or rigging.

9.2. Phase A Dynamic Prevention and Dynamic Treatment Procedures

9.2.1. Dynamic Prevention at Outpatient Clinics, Antenatal/Family Planning Clinics, Households, and Youth Multi-Sector Interventions

The Dynamic Prevention intervention will include integrated PrEP and PEP services at outpatient clinics, antenatal/family planning clinics, youth multi-sector interventions and via VHT workers in community households. The procedures for each trial will include counseling on and services of PrEP/PEP, choice of service location (e.g. clinic, household, community, phone), HIV testing options (including rapid or self-test provision), option for longer (e.g. 3 month) supply of PrEP, provision of a clinical officer's or nurse's mobile telephone number for immediate PEP starts any day of the week, routine assessment of barriers to initiation or adherence to PrEP/PEP, including the offer of personalized potential solutions such as choice of in-clinic or offsite service delivery, psychologic supports for traumatic experiences, and offer of concurrent, additional health or prevention related services. These services will be integrated into Uganda and Kenya Ministry of Health (MoH) guidelines driven services for PrEP/PEP, augmenting local standard care procedures for the prevention of HIV. If additional prevention modalities, such as long-acting cabotegravir (CAB-LA) or the dapivirine vaginal ring, become available, they will be incorporated into the Dynamic Prevention intervention. In both arms, participants will have access to standard prevention offerings and will have study visits every 24 weeks for up to 3 years for HIV testing and outcomes measurement (including PrEP/PEP adherence assessment via small hair sample). For convenience, standard PrEP/PEP procedures are described in Section 9.2.1.5. Table 6 describes the Dynamic Prevention intervention components.

Table 6. Dynamic Prevention Intervention Components

Component	Description	Approach
PrEP/PEP counseling and services	PrEP and PEP services delivered by clinical officer, nurse, VHT, with ability to cycle between PrEP and PEP as needed. The option of a longer supply of PrEP (e.g. 3 months) will be offered.	Participants will enroll in the prevention program, which includes standard VMMC, STI screening, and reproductive health linkage. Participants can use any of the PrEP, PEP and HIV testing-only options and switch options as needed.

Choice of service location	Services for PrEP/PEP/HIV testing will be delivered at clinic, home, or community site, with option for phone/virtual visits	Participants will have a choice of service location for prevention visits
HIV testing options	Rapid HIV tests will be provided as standard of care; HIV self-testing will be offered as an alternative	Rapid HIV testing will be the primary testing modality used. Participants will have the option of using HIV self-tests kits (with instructions from provider) as an alternative testing modality during follow-up.
Mobile phone access	Provision of clinician mobile phone number for immediate PEP initiation	Participants will be able to call clinical officer or nurse trackers 7 days a week, including holidays, for immediate initiation of PEP after potential HIV exposure. They may also have access to clinicians in case of questions, side effects or other concerns.
Assessment of PrEP/PEP barriers and personalized actions	Assessment of barriers to initiation or adherence to PrEP/PEP at each visit with personalized solutions offered	After identification of barrier to initiation of PrEP/PEP, or to adherence among those already enrolled in PrEP/PEP, clinicians will be trained to offer personalized solutions, including but not limited to flexibility of service delivery location (clinic or an offsite location of participants' choosing) and the integration of additional services into PrEP/PEP services such as reproductive health interventions. Clinicians will also have access to a data dashboard to monitor follow-up visits for HIV testing, PrEP/PEP provision.
Psychologic support for traumatic experiences	Clinical officer/nurse/counselor-provided support for traumatic experiences, in consultation with regional psychologist, among PrEP/PEP participants	Participants reporting traumatic experiences (e.g. non-consensual sex or IPV) will be able to receive additional counseling provided by clinical officers, nurses or counselors by phone or at health centers. Clinic staff will provide such counseling in consultation with regional study psychologist.

9.2.1.1. Clinician Training

Prior to recruitment and enrollment of participants, staff offering PrEP/PEP in the Dynamic Prevention intervention locations will receive training that will include, but will not be limited to, the following components:

- Counseling for PrEP and PEP, including assessment of appropriateness of the prevention method and delivery of service with respect and without judgement.
- Counseling clients on performing HIV self-testing
- Successful identification of current or anticipated barriers to PrEP/PEP initiation to new participants, or adherence among participants currently receiving PrEP or PEP, and the development of personalized plans to address these barriers. Solutions to commonly encountered barriers will be reviewed, and test cases and/or role-playing exercises may be employed to reinforce means by which positive plans and solutions can be employed.
- Possible side effects of commonly prescribed PrEP and PEP medications, tailored to regimens recommended by country MoH guidelines, will be reviewed with staff, along with counseling and support mechanisms to encourage adherence as appropriate.
- Counseling to provide psychologic support and additional community resources for participants reporting traumatic experiences.

9.2.1.2. Youth Multi-Sector Interventions

The youth multi-sector interventions will consist of single arm pilot studies (N=400 total) that aim to engage youth in HIV prevention in the context of existing community organizations offering life, financial and income generating skills. All youth will be offered dynamic prevention as outlined in the protocol in the context of settings either heavily attended by or that are youth focused. The settings in which the single pilots will be conducted may include existing microfinance clubs, *boda-boda* (i.e. motorcycle taxi) community welfare groups, and at small business kiosks (e.g. partnered with Community Volunteer Initiative for Development). These groups have continued during the COVID pandemic, are community-based, are attended by youth, and are models that could be generalized to interventions in Phase B.

9.2.1.3. Informed Consent and Enrollment

Prior to consent, participants will be screened to determine eligibility in the intervention. A waiver of consent will be obtained to confirm eligibility during the screening process. Written informed consent to participate in the study will be obtained from all participants. Consent forms will be translated from the original English to the language(s) spoken in the community. The consent form will be read to participants in their local language. Participants who agree to take part and sign the consent form will be enrolled in the study. Participants aged 15-17 years old who meet the definition of ‘mature or emancipated minors’ as set by the Uganda National Council for Science and Technology and the Kenya MOH can participate in these studies and will be consented on the adult participant consent forms.

9.2.1.4. Dynamic Prevention Procedures

After consent is obtained, the following procedures will be performed among participants randomized to the intervention arm of the clinic-based and VHT trials or enrolled in the youth pilot study:

- Study clinicians will assess the participant’s current HIV prevention methods, if any, and discuss with the participant his or her HIV prevention options and the services provided by the Dynamic Choice intervention
- PrEP/PEP counseling and services:
  i. Participants who have been referred or self-referred to the clinic for PEP, or those who indicate an HIV exposure in the past 72 hours, will be assessed immediately

for eligibility and appropriateness of initiation. Their ability to obtain PEP will be assessed, and eligible persons will be started the same day.

ii. Participants who do not report a recent exposure to HIV but are considered at high risk and not on PrEP will be introduced to the PrEP prevention method, with a discussion of its benefits and whether it meets the needs of the participant. If the participant is interested and eligible, he or she will be initiated on PrEP according to standard care guidelines.

iii. Participants who are already on PrEP or PEP will be introduced to the integrated clinic services of Dynamic Prevention intervention and their options for HIV prevention methods in the future will be discussed.

iv. At each visit, study clinicians will assess participant's needs and discuss HIV prevention methods available. Participants will be offered a clinic provider phone number for immediate contact and initiation of PEP in the event of exposure, with access to the provider 7 days a week, including holidays. Participants may switch between PrEP, PEP and HIV testing-only options as needed and according to their choice, in consultation with the provider. HIV self-testing will be offered as an alternative testing method. Participants who are at high risk but decline PrEP may opt for HIV testing every 3 months, with continual discussions with clinician of HIV risk and their options.

v. Choice of service location: Participants will have the option to receive PrEP/PEP services at any of the designated locations, including any of the clinic settings and at youth multi-sector interventions.

vi. For men, staff will ask about circumcision status, and discuss the benefits of VMMC, with referral to VMMC services for those interested.

vii. At each visit, study clinicians will assess the need for specialized counseling to provide psychological support for traumatic experiences (e.g. domestic violence and rape).

- Assessment of PrEP/PEP barriers and personalized actions:
  i. At the time of the first meeting and at subsequent visits thereafter, the study clinician will discuss with participants any potential barriers to PrEP/PEP initiation and adherence, such as distance to health facility, stigma, or work/school schedules.
  ii. If barriers are identified that may inhibit study participation or adherence to medications, the clinician will work with participants to develop solutions. These solutions may include offsite visits or flexible clinic hours or additional services, such as reproductive health care, at home visits.
  iii. If solutions to barriers to PrEP/PEP use involve offsite delivery, study staff providers will facilitate routine delivery of PrEP or PEP. Nurse trackers will deliver medications and perform standard prevention procedures at participants' homes or other desired offsite location. Participants may change the location of the offsite delivery throughout the program.
- Mobile phone access: Participants will be provided access to one clinician's mobile phone number at each facility taking part in the Dynamic Prevention intervention. Participants will be told they can call the clinician any day of the week, including holidays, in the even they would like to start PEP immediately due to possible HIV exposure. The phone number may also be used for other possible issues such as questions, concerns about side effects, and for general support of the participant's adherence of PrEP/PEP.

- Participant contact information will be collected, including home location and mobile phone number(s) if available.
- Baseline information will be collected including but not limited to date of birth, sex, clinic ID, education, and occupation
- Participants' opinions regarding approaches to promote retesting for HIV among persons at increased risk for HIV will be assessed in a brief survey during a follow up visit prior to study completion
- At each visit, signs and symptoms of acute HIV infection will be reviewed by study clinicians, including fever), diffuse rash, or swollen or enlarged lymph nodes. For patients suspected of having acute HIV, clinics will have access, provided by the study, for rapid viral load (using the Cepheid GeneXpert platform) if not available by the MoH, in order to ensure treatment of presumed HIV infection until test results return. The study will work with the MoH for partner notification in these cases to ensure completion and may also do social network outreach in order to identify and treat potential acute infection chains of infection.
- HIV seroconversion: For participants who are diagnosed with HIV in the course of the study, blood and hair samples will be collected for confirmatory HIV testing, drug resistance, and adherence measurement if indicated at the seroconversion visit and at 6 and 12 months. Patients will be referred to standard HIV care and treatment, including same-day ART start.

9.2.1.5. Standard PrEP and PEP Evaluations and Procedures According to MoH Guidelines

All participants (in both study arms) will follow standard in-country guidelines for the provision of PrEP or PEP.[97, 98] PrEP and PEP evaluations and procedures per in-country standard care guidelines are described below.

<u>PrEP Standard of Care Procedures</u>

Screening for eligibility for PrEP
Patients who are eligible for PrEP will be screened as follows:

- HIV antibody testing per standard care algorithm to confirm HIV-negative status
- Symptom screen to rule out possible acute HIV infection
- Symptom screen and/or laboratory testing for hepatitis B virus
- Assessment of contraindication to use of country-recommended PrEP regimen
- Determined ready and willing to initiate PrEP

For patients suspected of having acute HIV, clinics will have access, provided by the study, for rapid viral load (using the Cepheid GeneXpert platform) if not available by the MoH, in order to ensure treatment of presumed HIV infection until test results return.

PrEP counselling and initiation
At the time of enrolment into the PrEP program, providers will provide risk-reduction and medication adherence counselling. Condoms, along with education on their use, will also be offered. The possible side effects of the medications will be explained, and PrEP will be dispensed, per country guidelines. Patients will be given a 1-month supply of PrEP (with option for longer supply, e.g. 3 months, in intervention arm) and will be evaluated in 1 month for a review of adherence and side effects.

Follow-up on PrEP

After the initial and 1-month visit, subsequent visits will take place at least every 3 months. HIV antibody testing will be performed as per Table 7. Pregnancy testing will be offered for women of child-bearing potential. Providers will review the patient's understanding of PrEP, barriers to adherence, tolerance to the medication, and risk profile; risk reduction counselling will be done as needed. Symptoms of STI will also be evaluated, with treatment provided as indicated.

Table 7. PrEP Standard Care Schedule of Evaluations

Procedure or Evaluation	Baseline	1 month	Every 3 months[1]
HIV testing per country algorithm	X	X	X
Symptom screen for possible acute HIV infection	X	X	X
Symptom screen and/or laboratory testing for hepatitis B virus	X		
Counseling and adherence plan	X		
Prescribe and dispense PrEP	X	X	X
Adherence assessment and support		X	X
Pregnancy testing offered (women only)	X	X	X
Review barriers and perform risk reduction counselling	X	X	X
Symptom screen and follow-up for STIs	X	X	X
Creatinine testing (as available)[2]	X		X[2]

[1]Study visits among participants receiving the dynamic prevention intervention will occur at least every 3 months (at baseline and weeks 4, 12, and every 12 weeks during follow-up).
[2]Routine creatinine testing is currently recommended in Kenya only (at baseline and annually) and will be done if available by government health services

PEP Standard of Care Procedures

Screening for eligibility for PEP

Patients will be screened as follows:

- HIV antibody testing per standard care algorithm to confirm HIV-negative status
- Confirmation exposure occurred within past 72 hours and source was a person of HIV-positive or unknown HIV status
- Determined ready and willing to initiate PEP

PEP counselling and initiation

At the time of enrolment into the PEP program, providers will provide medication counselling and the possible side effects of the medications will be explained. Pregnancy testing will be offered for women of child-bearing potential. A 28-day supply of PEP will be dispensed; per country guidelines.

Follow-up on PEP

Patients will be followed up by phone or in-person at 7 days, 14 days and 28 days after PEP initiation. Counseling on side effects and adherence will be provided as per Table 8. PEP will be discontinued after 28 days. HIV antibody testing will be performed as per Table 8.

Table 8. PEP Standard Care Schedule of Evaluations

Procedure or Evaluation	Baseline	Day 7	Day 14	Day 28
Assessment of exposure and eligibility for PEP	X			
HIV antibody testing per country algorithm	X			X
Dispense medications	X			
Counseling, adherence assessment and support[1]	X	X	X	X
Creatinine testing (as available)[2]	X			
Pregnancy testing offered (women only)	X			X

[1]Including hair sample collection for adherence assessment, if indicated
[2]For patients on a regimen containing TDF, as available; routine creatinine testing is optional

9.2.2. Dynamic Treatment for Mobile Populations, Alcohol Users and Hypertension

The study intervention is enhancements of service delivery for mobile populations and heavy alcohol users, an intervention to promote linkage to care among persons screening positive for hypertension during community-based screening, and provision of care in a multi-disease chronic care model that includes service for HIV, hypertension and diabetes. ART will be delivered and monitored according to country guidelines and not provided by the study. Country guidelines for unsupressed viral loads will be followed. Diabetes and hypertension care will be provided using MoH standards. Table 9 describes the Dynamic Treatment intervention components.

Table 9. Dynamic Treatment Intervention Components

Component	Description
Mobile Travel Pack	1) Access to a mobility coordinator who will assist with transfers (to and from out-of-community HIV clinics), rescheduling, and out-of-facility refills; 2) Provision of a “travel pack” with alternative ART packaging options (e.g. ziplock bags, envelopes, pill boxes), up to 14-day supply of emergency ARVs, a packing list and mobility coordinator phone contact for unplanned travel; 3) Screening at every clinic visit for planned mobility; 4) Provision of a Mobile Number (hotline) to reach Mobility coordinator, along with routine phone outreach and a welcoming environment for participants to call or text Mobility coordinators with issues; 5) Provision of longer refills (up to 6-months) for planned travel; 6) HIV viral load counseling (review and explanation of meaning of viral load results)
Alcohol intervention	1) Two in-person alcohol counseling sessions with alcohol counselors with support from a clinical psychologist; 2) monthly booster phone calls; 3) HIV viral load counseling (review and explanation of meaning of viral load results); 4) Provision of a mobile number (hotline) to reach alcohol counselor, along with routine phone outreach and a welcoming environment for participants to call or text alcohol counselors with issues

Hypertension Linkage intervention	1) Travel voucher (financial incentive) conditional on linkage to hypertensive care
Hypertension Treatment intervention	1) All participants screened with blood pressure ≥160/100 mmHg will be referred to the nearest clinic for an initial clinicial evaluation. In Phase B, participants will be offered the option of ongoing follow-up at home facilitated by a VHT/CHV or in the clinic<br>2) Participants who do not link to care at a clinic may still be followed at home with VHT/CHV supported telehealth and encouraged to present to clinic for at least one in-person evaluation by a clinician.<br>3) VHT/CHV home visit frequency determined based on severity of hypertension<br>4) Hypertension care conducted at home with VHT/CHV measuring blood pressure and phone-based clinician consultation; referral for clinic visits if clinician concerned about symptoms or based on preference<br>5) Clinician conducts telemedicine visit with patient to ask/answer questions, review blood pressure, prescribe medications<br>6) VHT/CHV delivers medication from the clinic to the patient<br>7) VHT/CHV-entered blood pressure and medication data populates dashboard to facilitate population management and CHV supervision by clinician<br>8) Patients maintain the option of clinic visits for hypertension care if desired

9.2.2.1. Informed Consent

Prior to consent, participants will be screened to determine eligibility for the Dynamic Treatment intervention trials. A waiver of consent will be obtained to confirm eligibility during the screening process. Written informed consent to participate in the study will be obtained from all participants. Consent forms will be translated from the original English to the language(s) spoken in the community. The consent form will be read to participants in their local language. Participants who agree to take part and sign the consent form will be enrolled in the study.

9.2.2.2. Mobile Travel Pack

The Dynamic Treatment intervention for mobile services will include: 1) Access to a Mobility Coordinator who will assist with out-transfers (for out-migrants to transfer HIV care to or receive ARV refills from outside locations), rescheduling and easy entry to clinic for in-migrants to establish care, and out-of-facility refills, as needed; 2) Provision of a “travel pack” with alternative ART packaging options (e.g. ziplock bags, envelopes, pill boxes), up to 14-day supply of emergency ARVs, a packing list, and either mobile minutes (airtime) and/or Mobility Coordinator phone contact (“hotline”) for unplanned/unexpected travel; 3) Routine phone outreach and a welcoming environment for participants to call or text Mobility coordinators with issues; 4) A standard screening form for each clinical encounter at ART visits to assess plans for mobility out of the

community; 5) Provision of longer refills (up to 6-months) for planned travel; and 6) HIV viral load counseling (review and explanation of meaning of viral load results).

Clinic Services

At each clinic, eligible persons will be randomized either to standard care (control) or to meet with the study Mobility Coordinator at each visit (intervention). The mobility coordinator official duties for intervention group participants include assessing travel plans, offering assistance to patients on ART with in/out transfers, providing travel packs that include alternate ART packaging emergency ARV supplies, and mobile numbers and/or mobile airtime to facilitate unexpected travel needs, assisting with scheduling and re-scheduling visits, coordinating unanticipated transfers and refills at out-of-facility locales as well as non-standard refill schedules to match anticipated mobility patterns.

Procedures

- Staff will screen potentially mobile individuals who have traveled outside the community 2 or more times in the past year for trial eligibility. Staff will assess mobility of the patient using a standard screening form at the start of each visit.
- Staff will provide intervention group participants a mobile phone number from a local carrier to reach the Mobility Coordinator as needed, and/or a minimum number of minutes, for patients to use in the event of unexpected travel and the need for assistance with medication refills, transfers, or rescheduling of visits. At each clinic visit, the Mobility Coordinator with review with patients their mobile phone availability to ensure they have maintained the mobile number and have sufficient minutes for such immediate communication needs.
- Intervention participants will also receive HIV viral load counseling (review and explanation of meaning of baseline and any follow up viral load results), routine phone outreach and a welcoming environment for participants to call or text the Mobility Coordinator with issues.
- For intervention group participants who are mobile, the mobility coordinator will work with each participants' clinician to prepare HIV, hypertension or diabetes medications for the time in which participants will be away from the community. If needed, these medications can be delivered to the participant's home.
- Staff will maintain clinic documentation of mobile status of each HIV-positive participant to help track current clinic attendance status, specific needs for medications, transfers or other services, and future plans of travel or movement from the community. Each intervention group participant will be provided with phone access to the Mobility Coordinator who will assist with their immediate needs related to travel or transfers to and from the study community.

9.2.2.3. Alcohol Screening and Counseling

Men and women who self-report alcohol use and meet study eligibility criteria will be invited to take part in an alcohol counseling program (Healthy Living) that is based on the Information, Motivation, and Behavioral skills (IMB) model and whose goal will be to reduce drinking, adapted for East Africa from efficacious alcohol counselling programs.[93, 99] The program will include 2 in-person counselling sessions with "booster" phone counselling performed between the sessions at 3, 6 and 9-weeks post-randomization. The in-person counselling sessions will be workbook guided and include components designed to provide feedback on the consequences of alcohol use, emphasize individual responsibility, provide advice, offer empathy and help set goals.[93-95] Prior to

implementation, staff will be trained on the principals of alcohol counseling using the Motivational Interviewing style and use of the workbook (minimum 2-days training plus refreshers).

Procedures

We will randomize eligible patients 1:1 either to the alcohol counseling intervention (led by an alcohol counselor) or to standard care (routine HIV care control). Patients who agree to take part in the alcohol counseling trial will provide informed written consent prior to screening and randomization. Consent forms will be translated into local languages and a copy will be provided to participants. The components of the alcohol counseling intervention include the following:

- After consent is obtained, staff will inquire about the participant's general health, assess their ART use, and administer the standardized 3-question AUDIT-C tool to assess their alcohol use. A score of ≥4 for men and ≥3 for women will be considered positive for heavy alcohol use and render them eligible for randomization either to the alcohol counseling intervention or standard care. For participants who score <4 (men) or <3 (women), staff will provide appropriate counseling and refer them to resources, as needed, but inform them they are not eligible for the specific alcohol counseling trial discussed. Participants scoring 8 and above will be invited to participate, but will also be referred to their physician for a higher level of care for potential alcohol use disorder. Participants will also complete the full AUDIT assessment. Participants scoring 20 and above will also be offered a referral to their physician for a higher level of care for potential alcohol use disorder.
- Intervention participants will then take part in the first sessions of the 2-session alcohol counseling program. A trained alcohol counselor will administer the alcohol counseling workbook that describes the harmful effects of heavy alcohol use, elicits reasons to discontinue or reduce alcohol intake, elicits and records ways in which the participant could handle risky situations, and encourages the participant to make an agreement to alter their drinking habits. A drinking diary will be provided for participants to record their daily alcohol intake.
- Intervention participants will also receive HIV viral load counseling (review and explanation of meaning of baseline and any follow up viral load results), and baseline provision of a mobile number (hotline) to reach the alcohol counselor between sessions, as well as routine phone outreach and a welcoming environment for participants to call or text alcohol counselors with issues.
- Staff will obtain intervention participant's phone numbers (or a phone number of a close contact) and remind them they will receive a call every 3-4 weeks until his or her next health facility visit for a "booster" counseling session. This counseling session will assess current drinking, compare this assessment to the participant's drinking agreement recorded in prior counseling session, trouble-shoot problems and solutions, and discuss formulating a new drinking agreement for those who did not meet the goals of their previous agreement. The goals of the booster sessions are to reinforce motivation and behavioral skills, via reviewing drinking levels, ART adherence and reinforcing progress toward goals and discussing barriers or facilitators to achieving those goals.
- At the participant's next health facility visit, staff will administer the second and final part of the in-person counseling program, which will review the participant's prior agreement on alcohol use, reaffirm reasons to reduce or discontinue drinking, and create an action plan for the future.
- Following the primary endpoint measure (viral load at 24-weeks), all intervention arm participants will receive an additional "booster" counseling sessions (at 6- and 9-months post-randomization), to reinforce the alcohol counseling intervention.

9.2.2.4. Hypertension Linkage to Care and Treatment

Linkage to care: Following community mobilization, study staff will offer community-based screening for hypertension to persons ≥25 years old during mobile outreach to community venues. Persons screened who have blood pressure measurements ≥140/90 mmHg after three repeated measurements at a single community-based screening will be eligible for enrollment into the hypertension linkage to care trial. Eligible adults who consent to participate will then choose a scratch card that reveals whether they have been randomized to receive a transport voucher that is reimbursed upon linkage to hypertension care (intervention) or randomized to read a message thanking them for screening and encouraging them to link to care for their hypertension clinic appointment (control). All participants will be provided information about hypertension and where to access hypertension care and scheduled for a clinic appointment within 14 days of screening/enrollment. Participants with blood pressure ≥160/100 will be scheduled for a clinic appointment within 3 days of screening/enrollment.

Clinic Services:

- All community members who screen positive for hypertension during community-based screening and link to the local government-run clinic (regardless of participation in the trial) will be offered repeat measurement of blood pressure, enrollment into the hypertension clinic and evaluation for anti-hypertensive treatment.

Study staff will provide transport reimbursement (up to US$10) to Hypertension Linkage trial intervention participants who link to care within 30 days of screening.

Treatment: Following community mobilization, study staff will offer community-based screening for hypertension to persons ≥40 years old during mobile outreach to community venues. Persons screened who have blood pressure measurements ≥160/100 mmHg after three repeated measurements at a single community-based screening will be eligible for enrollment into the hypertension treatment trial. All persons will have immediate evaluation at clinical center for the hypertension. For follow-up, eligible adults who consent to participate will be randomized to receive follow-up hypertension care at home (intervention) or at the clinic (control) in Phase A. In Phase B, participants will be offered the choice of care at home via VHT/CHV and telehealth, or at the clinic.. All participants will have the option of accessing hypertension care at the clinic if desired, regardless of randomization arm. Hypertension visit frequency (home or clinic-based) will be determined based on severity of hypertension and clinician judgement, with recommended frequency of every 4 weeks if blood pressure is uncontrolled (≥140/90 mmHg) and every 12 weeks if blood pressure is controlled (<140/90 mmHg). Table 14 describes the schedule of study evaluations.

As per the standard of care, participants with co-morbid diabetes and/or HIV will have the option to receive VHT/CHV medication delivery for these conditions together with their hypertension medications. Medications will be prescribed by the clinician using standard treatment guidelines after completion of phone consultation for standard clinical evaluation. For participants with diabetes, the clinician will order a point-of-care random blood glucose when indicated to guide treatment decisions (to be performed by trained VHT/CHV or self-administered by participant). The clinician will advise the participant to come to the clinic for additional evaluation at any point they feel is clinically indicated.

Table 10. Hypertension Treatment Schedule of Evaluations*

Procedure or Evaluation	Week								
	0 (Baseline)	2	4	8	12	16	24	36	48
Clinical screening for signs/symptoms of secondary hypertension	X								
Creatinine and urinalysis testing*	X								
Clinic visit (intervention)	X								
Clinic visit (control)[a]	X	(X)	X	X	X	X	X	X	X
Home/Community visit (Intervention)[a]		(X)	X	X	X	X	X	X	X
Assessment of adherence, barriers to care, preferences for care site	X						X		X

*In Phase B, creatinine and urinalysis will be done through standard of care
[a]Both Intervention and Control arms (Phase A) or home and clinic visits (Phase B) will be scheduled every 4 weeks when blood pressure is uncontrolled and every 12 weeks when blood pressure is controlled (shorter intervals of 2 week follow-up should be considered for hypertensive urgency/emergency or at provider discretion in the setting of other clinical concerns). All participants (regardless of care engagement) should have visits by study staff for study measures at week 24 (+/-4 weeks) and week 48 (+/-4 weeks).

9.2.2.5. Social Network Referral Cards for HIV testing.

To better understand strategies to reach persons at risk for HIV and with undiagnosed HIV, and to leverage social networks among persons enrolling in our Phase A studies, we may offer referral cards for HIV testing and multi-disease standard screening (e.g. hypertension) to participants in the study to distribute to their friends or family. This will be done during screening and at out-of-facility outreach testing offerings.

9.2.3. Phase A Optimization

The results of phase A will be used to finalize Dynamic Treatment and Dynamic Prevention packages for evaluation in Phase B via the following steps: 1) evaluation of short term (48 week) effects on primary and secondary outcomes to eliminate setting and subpopulation components unlikely to contribute substantially to population-level effects; 2) evaluation of implementation outcomesand, 3) structured stakeholder consultation, in which the above data are reviewed, together with a systematic review of external data on concurrent advances in HIV treatment and prevention. With the many new prevention modalities in development, we expect that some of those may be integrated into phase B study design.

9.3. Phase B Intervention

The Phase B intervention will consist of a three part intervention: **1) Community-enhanced reach activities 2) Person-centered care delivery; and, 3) Data-enhancement to improve precision of interventions (Figure 2)**

Figure 2

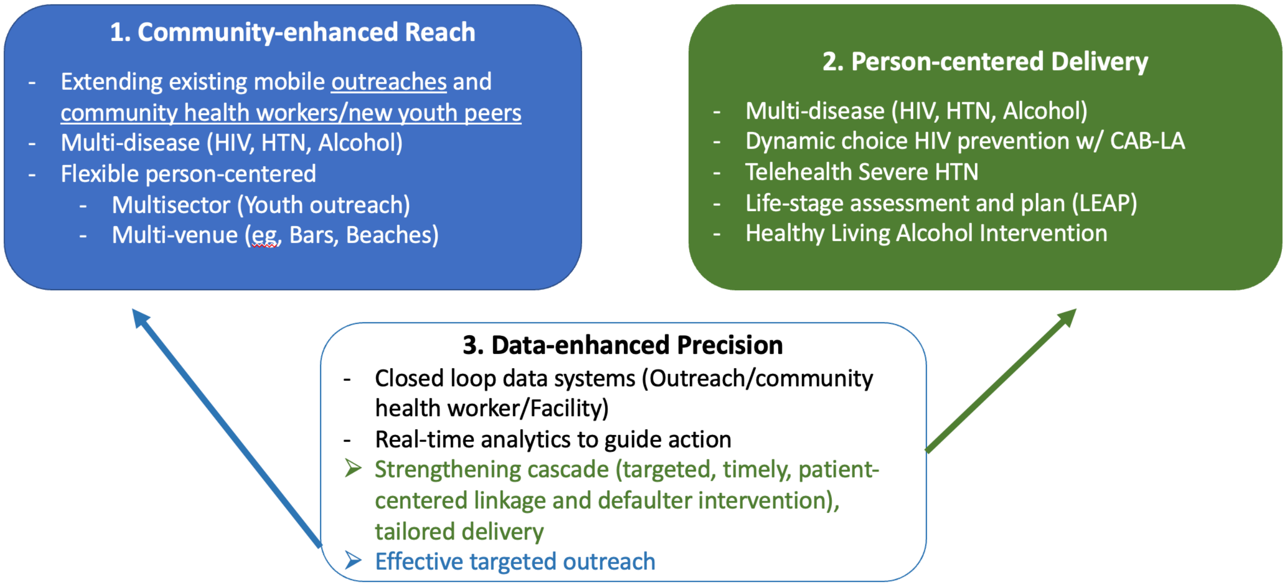

*CAB-LA will be included among Dynamic choice HIV prevention options if it is approved and available through in-country Ministry of Health programs.

Procedures

9.3.1 Community-Enhanced Reach

We will conduct a series of community engagement activities in the intervention arms designed to reach persons at risk for HIV, undiagnosed with HIV or diagnosed and fallen out of care working closely with community health system, leveraging existing mobile outreaches in the community. The aim will be to reach all residents to offer health education, referral to study intervention components, and HIV and multi-disease screening. We will partner with ongoing MOH activities to leverage existing activities.

<u>Community Engagement</u>

The community engagement process will entail the multiple meetings with community leaders, health care leaders, members of the target population and other key stakeholders discuss the study and optimal approaches to community participation and mobilization, built on the approach used in the SEARCH study.

These meetings with key leaders and stakeholders will be held in each region to introduce the study, answer questions and engage members in advising on topics that include:

- Recruitment strategies
- Identification of potential challenges and solutions to implementation
- Optimum dates, times and locations of the activities
- Populations or communities that could require special consideration
- Other issues tailored to individual regions

In preparation for Community Mobilization activities, additional meetings with village leaders throughout each community will be held. Advertising for the events may include poster, leaflet and radio advertising, with the enlistment of community-based volunteers to describe the events and encourage participation. These efforts will incorporate the principles of a study community engagement plan which may include incentives at the Community Mobilization event, such as a raffle for prizes, supported by the community.

Community Mobilization Activities

Venues: Activity venues will be driven by existing health center outreaches and target populations. The number of venues and days in operation will be adapted to the needs of the individual participating communities. The venues will generally be open to receive participants during business hours, and may include evening or early morning hours as well to reach specific populations (such as fisherfolk, transport workers, or barmaids).

Target Populations: Mobilization activities aim to engage adult men and women and adolescents living in each community who do not regularly access health care at local clinics, in order to provide health education and screening, and introduce health study interventions. Participants will meet with staff to review the services offered and provide informed verbal consent to take part. The verbal consent form will be read to them in the local language. Information will be collected from each community member, including name, village of residence, age, gender, and fingerprint biometric.

Pre-Test Counseling: After verbal consent, pre-test counseling will take place to describe to participants, in detail, the diagnostic services offered and answer questions. Education on the diseases for which community members are being screened will be provided, either in a group setting or on an individual basis.

Multi-Disease Screening: Multiple diagnostic services will be offered. HIV Antibody testing will be offered for all participants and additional measures and testing below may be offered and tailored to the population age and demographic.

- HIV testing will be performed according to country guidelines for those who do not self-report as HIV-positive according to country policy:
    i. Initial rapid HIV test
    ii. Participants with a negative result will be informed that they are HIV-negative
    iii. Participants with an initial positive result will undergo a second rapid HIV test. If the result is positive, participants will be informed of their HIV-positive status.
    iv. Participants with discordant rapid test results (first test positive, second test negative) will undergo a third “tie-breaker” HIV test. Participants with a positive “tie-breaker” test will be informed that they are HIV positive. Participants with a negative “tie-breaker” test will be informed that they are HIV-negative.
- Blood pressure: Blood pressure measurement for all ≥18 years
- Finger-stick blood random plasma glucose measure
- TB Screening: Symptom screening with referral to clinic for further evaluation via chest x-ray, sputum microscopy, and/or Xpert MTB/RIF assay[64, 100]
- Malaria rapid diagnostic test (RDT) for participants with a temperature of $\geq 38^{o}C$

- STI testing, including syphilis and gonorrhea testing

Post-test Counselling: All participants, regardless of test results, will receive standard post-test counselling per country guidelines, including participants newly diagnosed with HIV. For known HIV-positive participants already engaged in care, counselling will be adapted to meet their needs.

Linkage: Study staff will refer individual participants with a positive screening test (i.e. HIV, hypertension for same or next day clinic appointments via a mobile phone call from the clinician to the client will be made at time of diagnosis to facilitate linkage.

HIV: All HIV-positive participants will be referred to the local health facility for care. Participants who are known or self-reported HIV-positive may be provided with specialized counselling, including targeted information on the benefits of linking to care and addressing participants' specific fears. To increase outreach to persistent driver populations not yet in care, the study may coordinate with partner notification programs to embed free multi-disease "health checks" for sexual and social contacts of newly-identified HIV-positive individuals. HIV-negative participants will be offered PrEP referral if they consider themselves at high risk of infection.

Hypertension and diabetes: Participants who screen positive for hypertension or diabetes will be referred to the local health facility for follow-up. Other diseases: Referrals to health facilities for hypertension, TB and malaria and STIs will be made as with other referrals, and staff will provide a "warm handoff" to clinicians from local health facilities present at the venue, as available and appropriate.

Other services offered at Community Mobilization events will include sexual health education, referral to reproductive health services, and, in the event of a recent HIV exposure, referral to PEP services.

Table 11. Mobilization activities that may be performed, tailored to HIV epidemic and region

Target Population	Location	Frequency	Service menu
Adults with heavy alcohol use & female sex workers (FSWs)	Drinking venues	Every 3 months (weekend & night per venue)	HIV, multi-disease testing w/ transport vouchers
Adolescent girls and young women (AGYW - ages ≥15- ≤24), especially out of school	Community venues (pop-up tents)	Every 6 months	HIV and STI testing, menstrual hygiene support, pregnancy testing, life-skills training, solar access
Breastfeeding women	Immunization outreach venues	Every month	Infant/early childhood immunizations, HIV self-testing & on-site rapid test, early infant diagnosis, nutrition support, on-site PrEP/PEP and ART linkage & education, ART start for breast-feeding/pregnant women plus urgent referral

Pregnant women	Immunization outreach venues	Every month	Referrals to ANC, HIV self-testing, nutrition support, HTN measurements, on-site PrEP, ART start for breast-freeding/pregnant women plus urgent referral
Migrant workers	Outreach venues	Every 6 months	HIV and multi-disease testing, linkage to OPD, solar access
Fisherfolk	Beaches/fish markets	Every 3 months	HIV testing, HTN measurements, sexual health education, alcohol education, solar lanterns
Young men in transport	Truck/boda stages	Every 6 months	HIV testing, HTN measurements, alcohol education, STIs screening, life-skills and IT training, solar access

9.3.2 Person-Centered Delivery

HIV care delivery has advanced significantly incorporating more patient-centered approaches and differentiated care. In SEARCH SAPPHIRE Phase B, our intervention communities will add additional interventions aimed to achieve the following outcomes, including: Increase biomedical prevention coverage of those at highest risk, increase viral suppression, improve HTN control, reduce heavy alcohol use, strengthen MTCT cascade, and expand TB preventive therapy for children. The intervention procedures are described in sections 9.2.1 and 9.2.2 and in Table 12 below.

Table 12. Intervention Procedures and Target Population

Intervention	Procedures	Target Population
Dynamic Choice Prevention	Structured approach to provide patient-centered biomedical options for prevention. See section 9.2.1. CAB-LA will be incorporated into PrEP offerings when it becomes available in-country.	Persons at risk for HIV, age ≥15 years
Life events assessment and plan (LEAP) for youth, pregnant and breast-feeding women, and unsuppressed persons receiving treatment	We will conduct trainings with all providers to incorporte a life-stage assessment and plan (LEAP form) adapted from a randomized study showing that addressing life issues before delving into medical check-ins can improve viral suppression<br><br>LEAP forms will be utilized for youth, pregnant women, and persons without viral suppression	Persons with HIV and ≥15 to ≤24 years, or pregnant/ breastfeeding, or unsuppressed viral load (≥400 c/mL) at initiation or at treatment failure
Youth VHT to re-engage defaulters	New youth VHT will be trained to facilite re-engagment of youth who have defaulted from ART treatment or CAB-LA	Youth (ages ≥15 to ≤24 years) defaulters for ART

		treatment or CAB-LA
Severe Hypertension Treatment Telehealth	Persons with severe hypertension will have the option to participate in our telehealth model or standard care. Clinicians direct HTN management and VHT facilitate through telehealth – see section 9.2.2.4, Treatment section.	Persons ≥ 40 with severe uncontrolled hypertension (BP ≥ 160/100)
VHT Child TB Prevention Intervention	VHTs will be notified of children ≤ 5 years in need of TB prevention due to diagnosis of TB in the household; they will facilitate referral and engagement for TB prevention as per country guidelines	Children ≤ 5 where TB diagnosed in the household

9.3.3 Overview: Data Enhanced Community Precision Health

Both community-enhanced reach and person-centered delivery will be strengthened through the strategic collection and use of data. A mobile device-based app will enable VHT/CHVs and persons working at mobile outreaches to electronically record key participant measures made during home visits and at mobile outreaches (as described in prior sections), including (depending on context) HIV test results, blood pressure measurements, self-reported HIV risk, childhood vaccine coverage, and HIV care, HTN care, and biomedical prevention status. The app will be developed in partnership with country data teams charged by the Ministries of Health with implementation of country electronic medical record (EMR) systems and will be designed to harmonize with these systems, including harmonization with current country data collection instruments. Information from the EMR will further be used to enhance capability of the VHT/CHV to support linkage of persons with HIV or HTN not in care or at risk for HIV and not in prevention program and defaulters from care by providing data back to VHTs/CHVs via the app about persons failing to link or defaulting from care. Building on existing country efforts, the app will also allow for electronic data capture of existing CHV/VHT ongoing household census activities, including capture of GPS coordinates for each household. Finally, data collected during mobile outreach and VHT/CHV home visits will provide a population-based database, which will be analyzed to enhance the effectiveness of ongoing outreach activities by identifying high yield venues and locations and local regions and persons who would benefit form more frequent or intensive outreach or services due to, eg, elevated risk of HIV infection, unsuppressed viral load, and uncontrolled HTN.

Figure 3

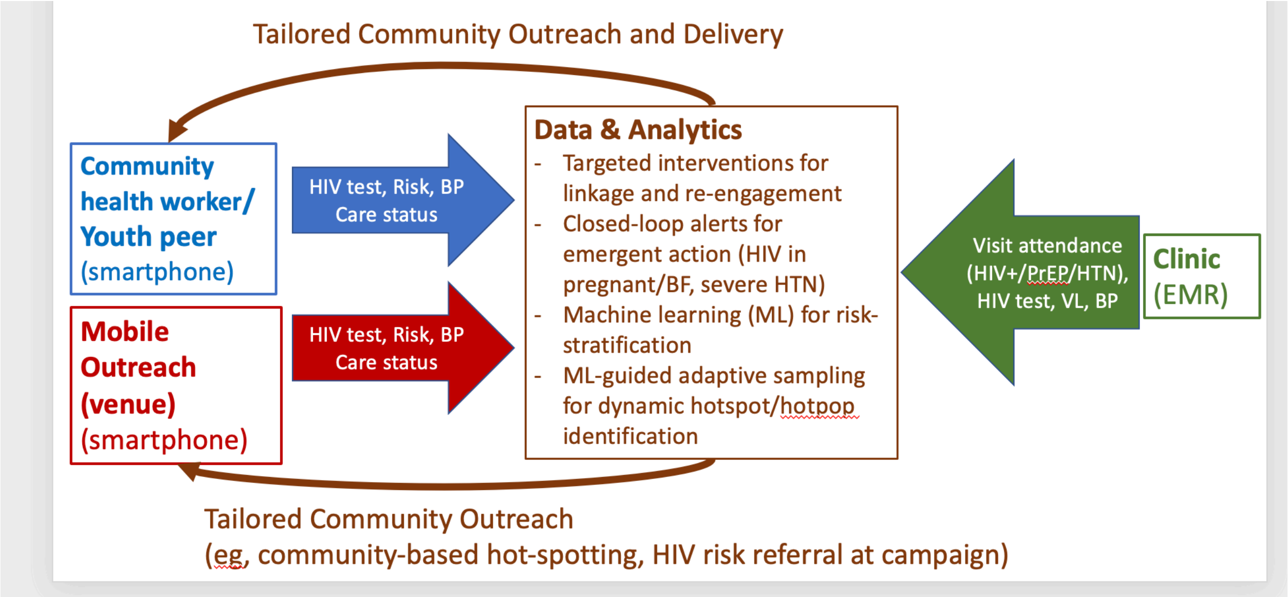

9.4. Phase A Measurements

9.4.1. Dynamic Prevention

The primary outcome will be PrEP/PEP "prevention coverage" defined as the proportion of follow up that an individual is protected from HIV infection with either PrEP or PEP, as assessed by self-report (with secondary analyses integrating drug levels from hair samples[101] and prescription refills). These data will be collected from surveys, Ministry of Health medical records and pharmacy records.

Process and implementation measures will be obtained from clinic-based records and logs. Examples of measures which may be obtained are in Table 13. Additional information on mediators will be determined by surveys, focus groups and in-depth interviews (Table 14).

Table 13. Phase A Dynamic Prevention Process Measures

Setting	Intervention Fidelity	Patient-level Intervention Adoption & Process Outcomes
**Outpatient clinic**	Proportion of patients who receive:<br>• Counseling & education about PrEP/PEP options upon enrollment<br>• 3-month HIV testing offered<br>• Mobile PEP/sexual health hotline<br>• Offer of delivery site options<br><br>Proportion of visits with:<br>• Structured barrier & plan assessment completed<br>• On time offsite delivery completed if requested<br>• Delivery of reproduction health services if requested	Adoption<br>• Number of calls to hotline and topics of calls<br>• Proportion of patients who select offsite delivery option<br>• HIV tests performed at 3-month intervals and results provided<br><br>Process<br>• Proportion of patients who initiate PrEP<br>• Proportion of patients who initiate PEP
**Family planning/antenatal clinic**	Core PrEP/PEP Fidelity Measures<br>*(defined for PrEP/PEP clinic above)*	Core PrEP/PEP Adoption & Process Outcomes<br>*(defined for PrEP/PEP clinic above)*

	Additional setting-specific measures<br>• Proportion of patients who receive offer of offsite delivery of reproductive health services<br>• Proportion of visits with delivery of reproductive health services if requested	Additional setting – specific measures<br>• Proportion of patients who select offsite delivery of reproductive health services
**Youth Multi-Sector Interventions**	Core PrEP/PEP Fidelity Measures<br>*(defined for PrEP/PEP clinic above)*<br><br>Additional setting – specific measures<br>• Proportion of persons attending youth multi-sector events who received health information on dynamic prevention	Core PrEP/PEP Adoption & Process Outcomes<br>*(defined for PrEP/PEP clinic above)*<br><br>Additional setting – specific measures<br>• Proportion (self-report) of youth who refer social network contacts for HIV prevention services.

Social and behavioral research using quantitative, qualitative and mixed methods will be embedded within each trial to measure pathways of intervention action and implementation factors that affect outcomes.

Pathways of intervention action. We posit individual-level, interpersonal and structural determinants of uptake of DCP, a selected number of which will be the focus of this research. These will be explored in the study population of DCP trial participants using in-depth interviews in sub-samples of participants in the trials, and in a sample of DCP providers. Key constructs to be measured, and domains that may be explored in qualitative inquiry are outlined in Table 14:

Table 14. Phase A Pathways of Intervention Action

Mediator
• Health knowledge, beliefs, and behaviors (including sexual risk behavior and alcohol use)<br>• Sexual and reproductive health empowerment<br>• Mental health and social support<br>• Structural barriers to care and prevention (including mobility, transport costs, and food insecurity)<br>• Perceived quality of care and trust in health care providers

9.4.2. Dynamic Treatment

- The primary outcome for the Mobile Travel Pack and Alcohol Counseling interventions will be viral suppression (HIV RNA<400 cps/ml) at 48 weeks for the mobility trial and 24 weeks for the alcohol trial. Secondary outcomes for the Mobility ("travel pack") intervention trial may include time retained in care (non-retention defined as the period of time following >2 weeks from a missed clinic visit until return to clinical care) and ART-covered time over 48 weeks. Secondary outcomes for the Alcohol Counseling intervention trial may include viral suppression at 48 weeks, heavy alcohol use at 24 and 48 weeks (measured by self-report and corroborated by phosphatidylethanol [PEth], a valid biomarker of prior 2-3 weeks' alcohol consumption[102,102]), and time retained in care (non-retention defined as the period of

time following >2 weeks from a missed clinic visit until return to clinical care) over 24 weeks. The primary outcome for the Hypertensive Linkage Intervention will be linkage to hypertension care at the local government clinic within 30 days of screening positive for high blood pressure during community screening. Secondary outcomes for the Hypertension Linkage intervention trial will include linking to hypertension care on the date of their scheduled appointment and blood pressure control (defined as systolic blood pressure <140 mmHg and diastolic blood pressure <90 mmHg) at 3, 6 and 12 -months post-enrollment. The primary outcome for the hypertension treatment trial will be HTN control at 24 weeks (blood pressure <140/90 mmHg). Secondary outcomes will include time to attaining hypertension control, HTN control at 12 months, proportion retained in care at 24 and 48 weeks.

Process and implementation measures for the Dynamic Treatment measures will be obtained from clinic-based records and logs. Examples that may be evaluated are shown in Table 15. Additional information on mediators will be determined as described in Table 14, with the addition of a sexual behavior and mobility survey.

Table 15. Phase A Dynamic Treatment Process Measures

Setting	Intervention Fidelity	Patient-level Intervention Adoption & Process Outcomes
**Mobile Travel Pack**	Proportion of intervention participants who receive:<br>• Education about support for travel by the Mobility Coordinator<br>• Hotline for unplanned travel & mobile minutes<br>• Screening for travel at each visit<br>• Offer of travel pack, including alternative medication packaging<br>• Offer of longer refills<br>• Assistance with entry to clinic (in-migrants) or transfer to out-of-community clinics (out-migrants)	Adoption (intervention)<br>• Number of calls to hotline and topics<br>• Proportion of patients selecting travel pack, including alternative packaging<br>• Proportion of patients selecting longer refills<br>• Facilitated planned and unplanned transfers completed<br>• Out-of-facility refills attempted and filled<br>Process Outcomes (intervention & control)<br>• ART covered time (Medication Possession Ratio)<br>• Number of missed visits
**Alcohol Intervention**	Proportion of intervention participants who receive:<br>• Initial counseling session, follow-up counseling session<br>• Booster phone calls attempted	Adoption (intervention)<br>• Engagement on counseling sessions & booster phone calls<br>Process Outcomes (intervention & control)<br>• ART covered time (Medication Possession Ratio)<br>• Number of missed visits
**Hypertension Linkage Intervention**	Proportion of intervention participants who receive transport reimbursement upon linkage to care	Process Outcomes<br>• Linkage to scheduled appointment
**Hypertension Treatment**	Proportion of intervention participants who receive the home/community follow-up visits.	Process Outcomes<br>• Home/community follow-up visits<br>• Clinical officer participation in off-site visit via mobile phone

9.4.3. Additional controls

To quantify the potential spillover between randomized arms and to understand secular trends, we may take 2 approaches. We may review retrospective records for primary outcomes among

participants (pre and post intervention, internal control). We may also enroll external controls for outcome comparisons with the clinic-based intervention trials for Dynamic Prevention (outpatient department and antenatal/family planning clinic) and for Dynamic Treatment (mobile populations and heavy drinkers). In communities otherwise not participating in Phase A, we will select clinics meeting the inclusion/exclusion criteria of each of the respective trials. In each site, individual participants will be recruited, consented, enrolled, and measured using analogous procedures of each of the respective trials. Outcomes will be compared between each trial's intervention arm, each trial's control arm, and the external controls using difference-in-differences.

9.5. Phase B Measurements

Baseline and interim measures (Intervention only):

Through community-enhanced outreach, described above (Section 9.3.1), we aim to obtain baseline measures on all community residents (N=~80,000 persons; ~40,000 adults) in the intervention arm at the start of Phase B. Specific measures include multidisease screening (e.g., HIV, hypertension, TB) and corresponding care status. During the course of person-centered delivery, described above (Section 9.3.2), we will also collect and utilize data to improve intervention delivery. For example, providers will utilize life events assessment and plan (LEAP) forms to identify and address barriers to HIV viral suppression. Likewise, through the data-enhanced community precision health model, described above (Section 9.3.3), we will also use data collected in both the community and at clinics to inform targeted interventions for screening, linkage, and re-engagement. As detailed below (Section 9.6.2), we will also measure and assess intervention implementation.

There will be no study-specific measures prior to endline in control communities. We may obtain community-level data at baseline from existing MoH records.

9.5.1 Phase B endpoint measures and evaluation

At month 30, we aim to obtain the following measures on all residents in both intervention and control communities (N=~160,000 persons; ~80,000 adults) to assess the primary and secondary endpoints of Phase B. The following Table provides an overview of endpoint definition, target population, and key metrics.

Table 16. Phase B Study Outcomes

Endpoint	Definition and Timing	Target Population	Measurement
HIV Incidence	HIV incidence rate (per 100PY), assessed at month 30	All community members aged ≥ 15 yrs	HIV rapid Antibody testing + Recency Assay
PrEP/PEP Coverage	Proportion of HIV-negative follow-up months during which PrEP/PEP (inclusive CAB-LA) was used, assessed month 24-30	All community members aged ≥ 15 yrs	Self-report + CAB-LA dispensing; validation based on TDF in hair
Population level Viral Suppression	Proportion of HIV+ adults with plasma HIV RNA level <400 cps/mL at month 30	HIV+ community members aged ≥ 15 yrs	HIV rapid antibody + plasma HIV RNA level
Mortality	Mortality rate (per 100 PY)	All community members aged ≥ 15 yrs	Comprehensive vital status survey at month 30
Tuberculosis (TB)	Annual incidence of active TB disease	All community members	TB registry
Hypertension Control	HTN control (BP <140/90 mmHg) at month 30	All community members aged ≥30 yrs	Sphygmomanometry
Heavy Alcohol Use	Proportion with AUDIT-C [≥3/female; ≥4/male] at month 30	HIV+ community members aged ≥18 yrs	AUDIT-C + HIV rapid antibody testing
Infant HIV-free survival	Proportion of infants born to HIV+/unknown status mothers who are alive and HIV-negative at month 30	Infants born to women HIV+ or unknown status during months 6-30	HIV rapid antibody testing and pediatric HIV testing
Pediatric immunization coverage	Proportion of children < 5 years with up-to-date vaccination at month 30	Children < 5 yrs	Vaccine records

CAB-LA will be included in PrEP/PEP coverage measurements if it is approved and available through in-country Ministry of Health programs by the month-30 evaluations. Prior to the start of month-30 measures, a households will be enumerated via remote GPS and through community health workers.

Month 30 Evaluation

At the time of the first visit to a household, all adult members present will be approached by study staff who will describe the measurement cohort activities and offer participation in the study. Households will be visited up to 3 times to identify all eligible adults (≥15 years) for participation. Written consent will be obtained from each individual participating, using a private area of the home for the consent discussion. Consent forms will be translated into local languages and a copy will be provided to participants. Identifying information will be collected from each household member, including name, age, sex, and digital fingerprint biometrics.

Demographic, Health and Economic Data Collection

During visits to each household, study staff will enumerate household members, identify relationships between household members and the head of household (as well as identify couples/partners within household members), and obtain demographic information (i.e. age, sex, occupation, highest level of education attained, school attendance, and marital status), health information (including self-reported HIV status, ART use, circumcision status (men only), any prior diagnosis and treatment for hypertension or diabetes and household economic data on workforce participation (i.e. income-generating activities) and income.

Testing

Pre-Test Counseling: After obtaining consent, pre-test counseling will take place to describe to participants in detail the diagnostic services offered and answer questions. Education on the diseases being screened will be provided.

Multi-Disease Screening: The following tests will be performed:

- HIV antibody testing will be performed on adults who do not self-report being HIV-positive according to country policy:
    i. Initial rapid HIV test
    ii. Participants with a negative result will be informed that they are HIV-negative
    iii. Participants with an initial positive result will undergo a second rapid HIV test. If the result is positive, participants will be informed of their HIV-positive status.
    iv. Participants with discordant rapid test results (first test positive, second test negative) will undergo a third "tie-breaker" HIV test. Participants with a positive "tie-breaker" test will be informed that they are HIV positive. Participants with a negative "tie-breaker" test will be informed that they are HIV-negative.
    v. Participants who test HIV-positive by antibody measurement will have a sample collected for future Geenius testing for study endpoint confirmation only.
- All adult community members will be assessed for recent HIV infection using a multi-assay algorithm (MAA).
- All adult participants who are self-reported HIV-positive or who test HIV-positive will have a sample collected for HIV RNA measurement and be assessed for alcohol use via AUDIT-C if aged ≥18 years. On a subset of participicants, we may also obtain dried blood spot samples for phosphatidylethanol (Peth) testing.
- All adult participants will be administered a questionnaire to assess HIV risk and use of oral PrEP or PEP. This will be the same questionnaire used to succesfuly assess HIV prevention coverage in the Phase A Dynamic Choice Prevention trials. Staff will also review clinical records for CAB-LA dispensing (if CAB-LA is approved and available through in-country Ministry of Health programs), and a subset of participants reporting recent PrEP/PEP use may be selected for hair sample collection to assess tenofovir levels. Hair tenofovir level testing: Hair samples collected from members of HIV-negative cohort who are active PrEP users (as determined by clinical records)
- Blood pressure measurements will be performed on all community members age ≥30 years.

Post-test Counselling: All participants, regardless of test results, will receive standard post-test counselling per country guidelines for point-of-care measurements, including participants newly diagnosed with HIV. For known HIV-positive participants already engaged in care, counselling will be adapted to meet their needs.

HIV RNA test results will be delivered to the participant's local health centre, if already receiving care, or the health centre they were referred to (see below) if recently diagnosed or out of care. PEth test and hair sample results, if obtained, will not be provided to participants.

Linkage: Study staff will refer individual participants with a positive screening test (i.e. HIV, hypertension) with facilitated linkage, as described previously (Section 9.3.1).

<u>Mortality, TB, MTCT and Immunization Surveillance</u>

Surveillance for mortality, TB, MTCT and childhood immunization coverage will be performed as follows:

- <u>Mortality</u>: A Vital Status Survey will be administered to the head of each household to collect information on any deaths that occurred among household members. We will use a

Vital Status survey previously utilized in the SEARCH "test and treat" trial,[103] which ascertained vital status on over 90% of participants and collected data on date of death (month/year) and cause of death within four categories (illness, accident/trauma, pregnancy/childbirth, or suicide). We will also collect data on location (if known) at time of death. This survey will also capture births into the household and migrations (i.e., persons moving in and out of the household).

- TB: Study staff will review local Ministry of Health (MoH) TB registries for information on incident cases of TB disease. Study staff will name-match TB cases (documented in MoH TB registries) to study participants (all ages). The government is the sole supplier of anti-tuberculosis antibiotics in Uganda and Kenya, and therefore TB therapy is only available through government-run or associated clinics and hospitals. All TB clinic dispensaries keep government registries with diagnostic and treatment-related outcomes (including death) for every case, as well as name, age, HIV status, date of TB diagnosis, date of TB treatment initiation, and residence (detailed to the village level). Staff may also review registries for data on TB prevention and treatment among study participants.
- Mother-to-child HIV Transmission: In the Vital Status Survey (see mortality, above), we will ask about any infants/children born over the study period. All children <3 years of age born to women with HIV or unknown HIV status will be assessed for vital status and undergo appropriate pediatric HIV testing.

9.5.2. RE-AIM Implementation Evaluation

RE-AIM-based implementation basic descriptive metrics (Table 17) will be calculated leveraging available medical record and pharmacy stock data, and study CRFs (to capture, for example use of the life stage assessment tool in youth clinics, frequency of mobile population outreach) at health centers and other locations.

Table 17. RE-AIM Dynamic Prevention Metrics, Phase B

	Target Populations	Evaluation Metrics	Measures & Data Sources
**Reach**<br>"The absolute number, proportion, and representativeness of individuals who are willing to participate in a given initiative"	1. All persons who are HIV-negative and community residents aged ≥ 15 years at study baseline<br>2. Subset of HIV-negative persons who are "at risk" for HIV transmission defined by self-report	Proportion of target population screened for Dynamic Prevention<br><br>Proportion of target population ever enrolled in Dynamic Prevention	Screening and enrollment study CRFs over 3-year study period<br><br>Socio-demographics (including mobility) and HIV rapid tests
**Effectiveness**<br>"The impact of an intervention on important outcomes, including potential negative effects, quality of life, and economic outcomes."	1. Baseline HIV-negative residents<br>2. Month 30 HIV-negative residents<br>3. Subset of HIV-negative residents "at risk" for HIV infection (*defined as above*)	Prevention coverage: proportion of target population with follow-up time covered by either PrEP or PEP<br><br>*See section 4 of SAP for additional details and secondary effectiveness outcomes*	Primary: Pharmacy refill data, clinical records over 30 month study period<br><br>Secondary: self-reported adherence; Tenofovir hair levels in subsample of HIV-measurement cohort at month 30

**Adoption**<br>"The absolute number, proportion, and representativeness of settings and intervention agents who are willing to initiate a program."	<u>Patients:</u> Persons enrolling in Dynamic Prevention<br><br><u>Providers:</u> PrEP/PEP providers at each of the delivery settings (HIV clinic, ANC clinic<br><br><u>Clinics:</u> All PrEP/PEP delivery sites in communities randomized to Dynamic Prevention	<u>Patient:</u> Proportion of target population making use of each of the choice-based options provided (e.g. mobile phone visits, self-test, etc...)<br>• *See Table 13 for additional detail on subpopulation-specific patient-level adoption measures, which will also be implemented in Phase B*<br><br><u>Provider:</u> Proportion of providers with a) knowledge of the core components of the Dynamic Prevention package; and, b) who have enrolled persons in Dynamic Prevention; and, c) who have made use of each of the components of dynamic treatment for their patients (provider level summaries of the individual adoption measures above)<br><br><u>Clinic:</u> Proportion of HIV clinics sites that have initiated Dynamic Prevention; clinic- level summaries of the individual-level adoption measures above<br><br><u>All:</u> Provider, Staff and Patient perceptions of the importance and utility of Dynamic Prevention and its components.	Screening and enrollment study CRFs over 30 month study period<br><br>Provider, staff, and patient surveys conducted at study baseline and month 30
**Implementation**<br>"At the setting level, implementation refers to the intervention agents' fidelity to the various elements of an intervention's protocol. This includes consistency of delivery as intended and the time and cost of the intervention."	<u>Patients:</u> Persons enrolling in Dynamic Prevention<br><br><u>Providers:</u> PrEP/PEP providers at each of the delivery settings (HIV clinic, ANC clinic<br><br><u>Clinics:</u> All PrEP/PEP delivery sites in communities randomized to Dynamic Prevention	**Fidelity** of intervention delivery<br><br><u>Patient:</u> Proportion of target population who receive each setting-specific core intervention component as scheduled (e.g. PEP hotline provided, offsite after hour visits offered)<br>• *See Table 13 for additional detail on patient level fidelity measures, which will also be implemented in Phase B*<br><br><u>Provider:</u> Proportion of providers and staff who complete scheduled trainings; provider-level summaries of the individual fidelity measures above<br><br><u>Clinic:</u> Timing and coverage with which clinics implement core intervention components; clinic-level summaries of the individual fidelity measures above<br><br>**Cost:**<br>• Component-specific costs of intervention (e.g., costs of PEP hotline, offsite visits)	**Fidelity:** implementation CRFs throughout study period, direct observation of intervention activities in clinic<br><br>**Cost:** direct observation, time in motion, clinic & administrative records, patient surveys, conducted during Phase B

		• Patient opportunity and transportation costs to access intervention/ prevention services<br>• Health systems costs (including drugs and clinical monitoring)<br>• *See Protocol Section 8 for additional details*	
**Maintenance**<br>"The extent to which a program or policy becomes institutionalized or part of the routine organizational practices and policies. Maintenance in the RE-AIM framework also has referents at the individual level. At the individual level, maintenance has been defined as the long-term effects of a program on outcomes after 6 or more months after the most recent intervention contact."	Communities randomized to Dynamic Prevention	Core components of each setting specific intervention in place 6 months after completion of the 30 month study	Brief clinic-level assessment via direct observation and review of clinic records.<br><br>Staggered initiation of follow up for communities in Phase B will allow for 6-month evaluation in 6 sites. For longer-term maintenance evaluation of interventions adapted, we will seek supplementary funding

9.6. Behavioral Measures

Phase A: We will use longitudinal mixed methods to understand behavioral mediators of the study outcomes in Phase A. We will conduct surveys, in-depth interviews (IDI), and/or focus group discussions (FGD) with participants and health care providers in the trials. .

Phase B: We will conduct qualitative research in SAPPHIRE Phase B to understand implementation processes and contexts and their potential influence on trial outcomes. This will include:

1) eliciting perceptions and understanding experiences of interventions among a) selected target populations and b) community-based practitioners (VHTs/peers) engaged in intervention delivery to evaluate facilitators and barriers to uptake of services;
2) observing out-of-clinic mobile outreach and service delivery strategies across diverse venues to understand social dynamics, spatial and environmental contexts, and any unforeseen aspects of implementation that may affect success of the strategies.

A focus on key features of Phase B not yet studied in Phase A. Our approach in Phase B is to focus qualitative inquiry on key design features and components of Phase B not previously explored in depth in our prior work in Phase A. These include:

1) an expansion of the cadre and role of VHT/CHVs and youth peers in
   - identifying persons with HIV or HTN not in care or at risk for HIV and not in prevention program and (re)linking them to services
   - supporting outreach to high risk persons to strengthen care cascades (VHT-based linkage and defaulter support) and support hybrid delivery (HTN telehealth, VHT DCP, alcohol intervention, other services)

2) an expansion of entry points to services, including not VHT/peer home visits but also mobile facility services, and venue-based outreach targeting specific target populations, including
   - adults w/ heavy alcohol intake and FSWs at drinking venues
   - AGYW (esp out of school) at community 'pop up' canopies
   - young men in transport at trucking/boda boda stages
   - pregnant and breastfeeding women at immunization clinics

3) A new prevention technology, CAB-LA, that has not before been rolled out at population-level. If CAB-LA is not approved and available through in-country Ministry of Health programs during Phase B, it will not be included.

9.6.1. Procedures

9.6.1.1. Mixed Methods Research Staff

Quantitative surveys and qualitative interviews, FGDs and participant observation will be administered by study staff trained in qualitative research methods and fluent in the local languages of each community.

9.6.1.2. Sampling for Mixed Methods Measures

In Phase A, quantitative surveys will be conducted, and FGDs and IDIs will be conducted in randomized controlled trials between 23 and 36 weeks of Phase A and among 45 HIV-uninfected young adults and 10 health care providers. Additionally, we will conduct qualitative interviews (IDIs)

with participants in Phase A treatment trials who are identified with HIV non-viral suppression at trial endpoint measurement.

In Phase B, Table 19 summarizes target populations and evaluations.

We will conduct in-depth interviews among purposive samples of participants and community-based practitioners, and conduct observations at venues, to pursue our research objectives as show in the tables below:

Table 18. In-depth interviews

**Behavior / Research domain**	**Study population**	**Purposive sampling category**	**Timing**
PrEP/PEP uptake<br>a) Perceptions of mobilization strategies<br>b) Barriers/facilitators of biomedical HIV prevention uptake	HIV- adults recruited from community venues who are eligible for PrEP/PEP (N=60)	1. Sex: male/female (including patrons and female sex workers)<br>2. Uptake: initiated or declined PrEP/PEP<br>3. multi-disease or HIV-focused recruitment	Following clinic-based screening for HIV and offer of PrEP/PEP
PrEP/PEP uptake<br>a) Perceptions of mobilization strategies<br>b) Barriers/facilitators of biomedical HIV prevention uptake	HIV- youth who are eligible for PrEP/PEP (N=20-30)	1.Sex: male/female<br>2.Age band: 15-19, 20-24<br>2. Uptake: initiated or declined PrEP/PEP<br>3. Outreach modality (peer home visit, community pop-up canopy outreach)	Following clinic-based screening for HIV and offer of PrEP/PEP
Linkage and retention in HIV treatment<br>a) Perceptions of intervention, outreach strategy<br>b) Barriers and facilitators to retention and adherence	HIV+ adults undiagnosed or out of care<br>(n=20-30)	1. Sex: male / female<br>2. interim outcome: linked to care and suppressed, not linked, linked but not virally suppressed<br>3. Outreach modality (CHV/VHT home visit, mobile facility-based outreach)	2-12 weeks after linkage referral
Linkage and retention in HIV treatment<br>a) Perceptions of intervention, outreach strategy<br>b) Barriers and facilitators to retention and adherence	HIV+ youth undiagnosed or out of care<br>(n=20-30)	1. Sex: male / female<br>2. interim outcome: linked to care and suppressed, not linked, linked but not virally suppressed<br>3. Outreach modality (peer home visit, community pop-up canopy outreach)	2-12 weeks after linkage referral
Perceptions of social role, motivations and intervention experiences for HIV and HTN of CHVs and Peers	CHVs (n=10-20)<br>Peers (n=10-20)	1. Sex: male / female<br>2. Worker type	Weeks 24-72 of study

Table 19. Observation during implementation

Research domain	Target population	Venue type
• Physical attributes of space and setting<br>• Appearances and behaviors of individuals in venues<br>• Social interactions (implementation team, patrons, and both)<br>• Public reactions to implementation strategies<br>• Informal public conversations related to strategies	• Adults with unhealthy alcohol use<br>• Bar maids/female sex workers	Bars
	• Migrant workers<br>• Young men in transport (truck driver, boda boda)	Truck/ Boda boda stands
	• Pregnant & Breastfeeding women<br>• Out of school youth	Mobile facility-based outreaches / immunization campaigns / pop-up canopies

9.6.1.3. Consent

Study staff will obtain written consent for participation in quantitative surveys, IDIs and FGDs.

9.6.1.4. Quantitative Survey Measures

Survey measures will include instruments that evaluate the following:

  - Health Knowledge, Beliefs, and Practices Survey: Administered to a subset of the HIV+ and HIV- Cohorts, collecting information on health knowledge, preferences and behaviors.
  - Sexual and Reproductive Empowerment Survey for Adolescents and Young Adults: Administered to a subset of the HIV+ and HIV- Cohorts under age 25, measuring perceived psychological empowerment.
  - Felt Stigma Survey: Administered to a subset of the HIV+ and HIV- Cohorts, the HIV Felt Stigma survey collecting information on HIV-related stigma.
- In-Depth Interview (IDI) & Focus Group Discussions (FGDs): Administered to a subset of members of the HIV-negative and HIV-positive cohorts by staff trained in qualitative interview practices, with topics including barriers to care, engagement in HIV prevention and/or care services, stigma, sexual risk-taking, and other subjects

9.7. Costing Evaluations

9.7.1. Overview

We will use standard micro- costing techniques and time-and-motion studies to measure the cost of each intervention in Phase A (between weeks 12 and 36 of Phase A) and the combination packages in Phase B (week 24 and week 120 of Phase B). Data will be collected from care providers, patient medical records, clinic and study administrative and financial records. Patient questionnaires will be used to elicit expenditures for transportation to participate in the intervention and receive care, expenditures for additional health services received (e.g. hospitalization), and the value of the time lost to participate in the intervention and access care.

9.7.2. Costing team procedures

Costing team composition and training: Cost data collection will be conducted by two teams, one in Uganda and one in Kenya. Each Cost Data Team will consist of a medically-trained person, a person trained in finance, economic or accounting and a Costing Officer, who has prior experience conducting costing data collection under the SEARCH trial. The study's Costing Co-Investigator will train the team on the tools and procedures to collect costing data and do time in motion studies. After training, the costing team will schedule visits to all 4 sites (Phase A) and 8 intervention and 8 control communities (Phase B). Upon completion of cost data collection, in-country teams will enter and submit data to the Costing Co-Investigator for review to clarify any aspects of data collection. The Costing Co-Investigator will complete costing data report will be generated.

Costing data collection. At the site visits, the costing team will collect information on expenditures for each of the study interventions in Phase A and for each of the packages (Dynamic Prevention, Dynamic Treatment) in Phase B classified in one of four categories; (i) personnel (including fringe benefits); (ii) recurring supplies and services; (iii) capital and equipment; and (iv) facility space (as appropriate). We will also collect retrospective expenditure data to document program start-up costs. The costs of each program activity will be identified through interviews with administrative, finance and human resources officers, supplemented by direct observation in a limited number of formal time-and-motion studies. The costing approach will emphasize resources utilized, rather than out-of-pocket costs. For example, where expenditures do not fully reflect the opportunity cost of the resources used (e.g., donations or transfer payments), we will adjust the valuations accordingly. Costs for capital items will be amortized on a straight-line basis over their expected useful life, and assuming no salvage value. Facility space required by the interventions will be valued at the market rental rate. Following assignment of expenditures to these four broad categories, we will further allocate each expenditure item across three areas, (i) service delivery; (ii) staff training directly related to service delivery; (iii) indirect costs consisting of intervention overhead and administration.

Personnel Costs and the Allocation of Overhead Across Activities: Overhead and administrative costs will be allocated to the programs in proportion to the full-time equivalent staff (FTEs) that study intervention service providers constitute of all service provider FTEs at the study sites. We expect that the preponderance of intervention costs will be personnel time. The appropriate approach to measuring personnel time will depend upon the way services are organized at the study sites. For example, if dedicated staff is hired specifically for these interventions, costs can be obtained directly from compensation data. In the more likely case that service providers have multiple responsibilities, the time dedicated to these interventions can be obtained via interviews supplemented by direct "time and motion" observations, including completion by staff of logs recording major activities, for one-week periods during each assessment period.

Measuring Unit Costs: Outputs (denominator of the unit cost) include the numbers of participants receiving each type of study-supported services. Unit costs are defined as the relevant program costs divided by each of these outputs, respectively. To supplement this information, we will also collect information on participant-level contact hours, to allow us to examine the importance of participant and intervention-level factors related to variation in unit cost. We will also assess the variation in unit costs across the study sites and identify the major determinants of that variation. These findings are intended to provide program managers with insights into costs structures that may be used to enhance program efficiency.

9.8. Mathematical Modeling

The study will adapt an existing, individual-based simulation model of HIV sexual transmission, progression and effects of prevention and treatment (HIV Synthesis Model) to model the effects (and costs) of the Phase A interventions in order to inform the interventions to move into the Phase B trial and, after Phase B, to inform an Investment Case.

9.8.1. Procedures

The HIV Synthesis Model updates in 3-month periods and simulates age, gender, number of condomless sex partners, HIV testing and HIV acquisition risk and occurrence (dependent on viral load in condomless sex partnerships had by people of the opposite sex, considering alternative age-sex mixing patterns). Health impact is conveyed in terms of viral suppression, morbidity and mortality, and also brought together as DALYs in the whole adult population. TB risk in HIV infected and uninfected persons will be included in the model. The model has previously been used to evaluate the potential long-term effectiveness and cost effectiveness of several treatment and prevention interventions, including HIV testing initiatives, oral TDF/FTC PrEP, VMMC, choice of specific ART, and adherence and monitoring strategies for ART. For the purposes of this study, the model will be calibrated to local historic epidemic data. The study will add in to the structure a risk of hypertension with its effect on cardiovascular disease risk, and the beneficial effects of antihypertensive medication on hypertension. Effects of interventions to modify alcohol use will be modelled as the effect of those interventions on ART adherence (and PrEP and PEP adherence).

After Phase B the study will build on this first phase of the modelling to inform an investment case for a final intervention package shown to be beneficial in Phase B. The underlying modelling approach to the Investment Case will be as used above when modelling the potential effects and cost effectiveness of intervention packages but will be additionally informed by effectiveness data from Phase B. If our intervention proves to be sufficiently impactful on HIV incidence we will put together an Investment Case to reduce incidence sustainably below 1 / 1000 person years. This will likely be arguing the case for additional resources to achieve this. We will additionally use the results to inform the current breakdown of HIV program resources from the current budget envelope. We will convey long term health impacts in terms of morbidity and mortality from AIDS and non-AIDS conditions and DALYs, projecting over several decades.

9.9. Participant Discontinuation

Participants are free to withdraw from active participation in the study at any time upon request. An investigator may discontinue a participant from the study for the following reasons:

- The participant meets a previously unrecognized exclusion criterion
- Significant non-compliance with the study
- Any event or situation that occurs such that participation would not be in the best interest of the participant

10. ASSESSMENT OF SAFETY

10.1. Safety Assessment Overview

All medications provided in this study are standard care for the intervention described. HIV testing and care will be performed according to MoH guidelines, including regular safety assessments such as VL for HIV-positive individuals in care, and other standard measurements. HIV RNA will be performed every 24 weeks in the Phase A dynamic prevention trials.

Care will be taken to protect the privacy of participants in this study. However, there is a risk that others may inadvertently see participants research or medical information, and thus privacy may be compromised.

10.2. Adverse Event Procedures and Reporting Requirements

As all intervention procedures in this study are standard care and prevention procedures recommended by the Uganda and Kenya MoH, and risks to participation in general are minimal, this study will not monitor or collect data on all adverse events. In the unlikely event that a serious adverse event (SAE) is considered possibly, probably or definitely related to the study, or in the event of unexpected incidents or protocol violations, reporting to University of California-San Francisco CHR, Makerere University SOMREC, and Kenya Medical Research Institute SERU IRBs will be done as outlined in Table 20. Investigators will be monitoring events the intervention arms, and suspected events will be reviewed by study investigators prior to submission to the IRB.

Table 20. Event Reporting Timeline

Institution	Type of Events	When to Report
**UCSF-Committee on Human Subjects Research (CHR)**	• External (off-site) adverse event that UCSF PI determines changes the study risks or benefits, OR necessitates modification to the CHR-approved consent document(s) and/or the CHR-approved application/ protocol<br>• On-site protocol violations (events occurring at UCSF)	• Within 10 working days of PI's awareness
**Makerere University School of Medicine Research Ethics Council (SOMREC)**	• All Serious or Unexpected events considered possibly, probably or definitely related	• Fatal or life-threatening events within 3 working days of awareness<br>• All other SAEs within 7 calendar days
**KEMRI Scientific and Ethics Review Unit (SERU)**	• All Serious or Unexpected events considered possibly, probably or definitely related	• Notification within 24 hours of awareness; report within 5 working days
**National Institute of Allergy and Infectious Diseases (NIAID)**	• All Serious or Unexpected events considered possibly, probably or definitely related	• At the time of reporting to IRBs

11. DATA HANDLING AND RECORDKEEPING

11.1. Data Management Responsibilities

Data management for this study will be overseen by the Infectious Diseases Research Collaboration (IDRC) Data Management Center located in the main IDRC research complex in Nakasero, Kampala, Uganda. The Data Management Center (DMC) is also responsible for providing IT support to all staff members.

Data Management System
For any study components using paper data collection, IDRC has designed and developed a custom Data Management System that is used to manage the data, using Microsoft SQL Server as the backend for all data storage. MS Access is used for double data entry and SAS programs are used for comparing 1st and 2nd entry and generating discrepancy reports. In addition, the DMC has developed custom Visual Basic programming for direct survey and data entry of data in the field. Data Transformation Services/ SQL Server Integration Services (DTS/SSIS) packages are used to automatically import/export any new data and stored procedures written in T-SQL are used to automatically generate new data queries on a daily basis. There is a web interface to the whole system written in ASP.NET to allow users to view the data, view reports, and even modify the data they are authorized to access. With regular backups and a full audit trail, the whole system is regulatory compliant (21 CFR Part 11).

11.2. Essential/Source Documents and Access to Source Data/Documents

Data is collected as a mixture of paper Case Report Forms (CRFs) and logs and electronic data capture from health facility databases and field data tablets. Data is entered locally and uploaded to the DMC after quality assurance measures have taken place. Only study investigators and staff affiliated with the study will have access to source data.

External data sharing will be made through a data request proposal procedure coordinated by the PIs and the IDRC Data Department. A standardized data request sheet will be completed by outside investigators and reviewed by the senior management and/or the Principal Investigator. Data and associated documentation will be available to users under a data-sharing agreement that provides for: (1) a commitment to using the data only for research purposes and not to identify any individual participant; (2) a commitment to securing the data using appropriate computer technology; and (3) a commitment to destroying or returning the data after analyses are completed. User registration is required to access or download files. As part of the registration process, users must agree to the conditions of use governing access to the public release data, including restrictions against attempting to identify study participants, destruction of the data after analyses are completed, reporting responsibilities, restrictions on redistribution of the data to third parties, and proper acknowledgement of the data resource.

11.3. Quality Control and Quality Assurance

The data management system is designed to collect, transfer and store data for Data Management Center studies. Data from any paper records will be 100% double entered into the system via Microsoft Access Databases or Web-based data entry screens. Locally collected data is compressed, password-protected, and then securely uploaded to a cloud based server (Network Solutions, Inc.) using secure-FTP via the FileZilla application. Cloud-based uploads are downloaded daily to the Kampala DMC. After the data has been input into the Data Management Center server, it is electronically verified and is then written to the SQL database. Edit checks or queries are run

nightly and the results are posted to a secure clinical trials website so that the sites can electronically address problems with the data the next day. The site corrects data via the website and the database is updated automatically.

In order to ensure data security and integrity, the following measures will be implemented:

- All members of the study team will be educated in the study protocol prior to the onset of the study.
- Detailed Standard Operating Procedures (SOPs) will be written for all project activities and be provided to relevant team members.
- Team members will be thoroughly trained on the SOP's.
- Where applicable, team members will receive additional training on the use of tablet computers.
- All data transcribed from paper will be double data entered or verified.
- All electronic data will be backed up regularly.
- All data will be transferred to the main Data Center in Kampala to the secure server. This sever is backed up on a daily basis and a monthly backup is stored off-site.
- All computers, including the tablets, will be password protected.
- All computers, including tablets, will be locked in a secure room each night.
- Any Log Books and CRF's will be locked in a secure room each night.

12. CLINICAL SITE MONITORING

Site monitors under contract to the University of California, San Francisco will visit participating clinical research sites to review participants records, including consent forms, CRFs, and laboratory records to ensure protection of study participants, compliance with IRB approved protocol/amendments, and accuracy and completeness of records. The monitors will inspect sites' regulatory files to ensure that local regulatory requirements, in addition to U.S. Federal regulations, are being followed.

13. ADMINISTRATIVE PROCEDURES

13.1. Regulatory Oversight

The proposed research study will be reviewed and approved by the IRBs of all the participating institutions in the U.S., Uganda and Kenya. This includes the UCSF Committee on Human Research (CHR), the Makerere University School of Medicine - Research and Ethics Committee (SOMREC), the Uganda National Council of Science and Technology (UNCST), the Kenya Medical Research Institute (KEMRI). As described in Section 15.10, this study will employ four levels of data and participant safety monitoring including Study Steering Committee oversight, US and host country Institutional Review Board (IRB) review, Data Safety Monitoring Board (DSMB) Review, and External Clinical Site Monitoring. Section 10.2 describes event reporting guidelines to individual IRBs overseeing this study.

13.2. Study Implementation

No study activities will commence prior to approval of US and host country IRBs and all other required local and international institutions. This study is funded and supported by the National Institutes of Health.

13.3. ClinicalTrials.gov

This protocol will be registered in ClinicalTrials.gov.

14. HUMAN SUBJECTS PROTECTIONS

14.1. Institutional Review Board/Ethics Committee

The proposed research study will be reviewed and approved by the IRBs of all the participating institutions in the U.S., Uganda and Kenya. This includes the UCSF Committee on Human Research (CHR), the Makerere University School of Medicine - Research and Ethics Committee (SOMREC), the Uganda National Council of Science and Technology (UNCST), the Kenya Medical Research Institute (KEMRI).

14.2. Vulnerable Participants

Many persons living in these regions of East Africa are economically or educationally disadvantaged, by American or African standards. The investigative team believes that the opportunity to participate in this study should be made available to people independent of economic and educational attainment. Participants in rural populations and those who are educationally disadvantaged are among those most at risk in their country for poor HIV care outcomes, and it is important to provide opportunities to participate in research to all people independent of their literacy. If a potential study participant is unable to read or write, his or her fingerprint will substitute for a signature, and a signature from a witness to the informed consent procedures will be obtained. Participants taking part in activities for which a verbal consent is required will have the consent text read to them in their language. None of the diagnostic tests or procedures provided in the study are more harmful to children or pregnant women as compared to others. All tests and treatments provided are standard of care. Prisoners (incarcerated persons) will not be included in the research during incarceration.

14.2.1. Pregnant Women and Fetuses

Pregnant women may take part in the study and could benefit from their participation through testing or intervention activities. None of the diagnostic tests or procedures provided in the study are more harmful to pregnant women and fetuses as compared to other groups.

14.2.2. Children

Youth aged 15 years and up are among the populations most in need of improved HIV outcomes and are a group targeted for participation this study. The Uganda National Council for Science and Technology (UNCST) has determined that individuals aged ≥14 years are able to provide independent consent for research if they have a sexually transmitted infection, and the National AIDS and STI Control Programme (NASCOP) and Kenya Medical Research Institute (KEMRI) state that individuals aged ≥12 years are able to provide independent consent for research. In both Uganda and Kenya, children of the age represented in this study can obtain HIV testing services and HIV care without parental consent, and including parental accompaniment could significantly inhibit youth from accessing these services. As with adults, participants <18 years will be given sufficient time to read or be read the consent form in their language and to ask any questions. None of the diagnostic tests or procedures provided in the study are more harmful to youth as compared to other groups.

14.2.3. Illiterate Participants

They study will enroll participants who may be economically or educationally disadvantaged. Participants in rural populations and those who are educationally disadvantaged are among those

most at risk in their country for poor HIV care outcomes, and it is important to provide opportunities to participate in research to all people independent of their literacy. If a potential study participant is unable to read or write, his or her fingerprint will substitute for a signature, and a signature from a witness to the informed consent procedures will be obtained. Participants taking part in activities for which a verbal consent is required will have the consent text read to them in their language. As with other groups, illiterate participants will be provided sufficient time to be read the consent form or ask questions in order to understand study procedures.

14.3. Informed Consent

14.3.1. Informed Consent Process

All written and verbal consent forms will be translated into the local language and back translated into English to ensure correct use of language. Consent forms will be read aloud to participants by trained staff. The informed consent will describe the purpose of the study, all the procedures involved, and the risks and benefits of participation. Interviewers will ask participants to summarize the study and explain the reasons why they want to participate. Either a signature or a thumbprint (for those who cannot read) will be acceptable to confirm informed consent for participation in the study, in the case of written consent forms. Witnesses independent from the study will be required to be present for consent discussion and co-sign consent forms for participant who are illiterate. Affirmation of verbal consent will be obtained through recording to responses on verbal consent logs. Consent documentation may be tablet versus paper based to maintain optimal infection control.

To minimize the likelihood of persons feeling pressured to participate in research we will emphasize the concepts of individual voluntary choice to participate in research and the need for research assistants to respect the voluntary choice of others during the process of obtaining informed consent. Study participants will also be informed that participation can be stopped at any point during the study at their request. Descriptions of individual consent forms and procedures are below.

<u>Written consent for Phase A Dynamic Prevention Intervention components</u>: Participants taking part in the randomized controlled trials of PrEP/PEP activities in Phase A will meet with study staff to review the services offered and provide informed written consent to take part after being given adequate time to review the consent form have all questions answered. Participants who are aged ≥15 to ≤17 years and are not deemed mature or independent minors by the governments of Uganda and Kenya will require parental co-consent on the same written consent document in order to participate.

<u>Written consent for Phase A Dynamic Treatment Intervention components</u>: Participants taking part in the randomized controlled trials of the Mobile Travel Pack and Alcohol Use intervention activities in the Phase A clinics, or in the Hypertension Linkage or Treatment trials, will meet with study staff to review the services offered and provide written informed consent to take part after being given adequate time to review the consent form have all questions answered. As described in Section 15.2.2, both Uganda's UNCST and Kenya's NASCOP and KEMRI institutions state that individuals who are aged ≥15 to ≤17 years may consent independently if the research involves study of a sexually transmitted infections.

Verbal Consent for Hypertension Screening in Phase A: Participants taking part in screening for the hypertension linkage or treatment studies will meet with study staff to review the services offered and provide informed verbal consent to take part. The verbal consent form will be read to them in the local language, confirmation of consent will be documented as affirmation of their agreement to participate.

Verbal Consent for HIV Screening and Referral for Alcohol Intervention in Phase A: Participants who are candidates for the alcohol intervention study will be offered multi-disease screening including HIV testing and alcohol use-related questions at the health facility and will provide informed verbal consent to take part. The verbal consent form will be read to them in the local language, confirmation of consent will be documented as affirmation of their agreement to participate.

Written Consent for Surveys, In-Depth Interviews and Focus Group Discussions in Phase A and Phase B: Written consent will be obtained from each youth and adult participating in: 1) stigma, mobility, sexual behavior, youth life-skills and behavior surveys, and each youth and adult selected for in-depth interviews and focus groups. Consent forms will be translated into local languages and a copy will be provided to participants. Participants who are aged ≥15 to ≤17 years and are not deemed mature or independent minors by the governments of Uganda and Kenya will require parental co-consent on the same written consent document in order to participate.

Verbal Consent for Health Testing Activities in Phase B: Participants taking part in the Community Mobilization and Mobile Outreach testing activities will meet with study staff to review the services offered and provide informed verbal consent to take part. The verbal consent form will be read to them in the local language, confirmation of consent will be documented as affirmation of their agreement to participate.

Written consent for the Dynamic Prevention Intervention in Phase B: Participants taking part in the Dynamic Prevention intervention for PrEP (including CAB-LA if approved in-country), PEP, or HIV-testing services with VHT/CHV re-engagemnet of youth defaulters will meet with staff to review the services offered and provide informed written consent to take part after being given adequate time to review the consent form have all questions answered. Participants who are aged ≥15 to ≤17 years and are not deemed mature or independent minors by the governments of Uganda and Kenya will require parental co-consent on the same written consent document in order to participate.

Written consent for the Person-Centered Treatment Delivery (Dynamic Treatment Intervention) in Phase B – LEAP Form, Mobile Travel Pack, and Alcohol Use in Phase B: Youth and adults taking part in the LEAP form guided discussion, mobile services and/or alcohol counseling intervention for those who screen positive for heavy alcohol use, including VHT/CHV re-engagement of youth defaulters, will meet with staff or counselors to learn more about the research and provide informed written consent to take part in the specific study activities. As described in Section 15.2.2, both Uganda's UNCST and Kenya's NASCOP and KEMRI institutions state that individuals who are aged ≥15 to ≤17 years may consent independently if the research involves study of a sexually transmitted infections.

Written consent for Severe Hyptertension Treatment Telehealth in Phase B: Eligible adults taking part in Severe Hypertension Treatment Telehealth will first meet with staff to learn more abou the research and provide informed written consent to take part.

Verbal consent for Volunteer Health Team/Community Health Volunteer (VHT/CHV) staff: Youth VHT/CHV members who are taking part in activities related to the engagement of defaulters of ARV treatment or HIV prevention therapies will be consented to have minimal demographic data and work history data about them collected. The verbal consent form will be read to them in the local language by the study coordinator or study staff, and confirmation of consent will be documented as affirmation of their agreement to participate.

Verbal Consent for Measurement Outcomes at Month 30 in Phase B: At the time of the first visit to a selected Measurement Cohort household, all adult members present will be approached by study staff who will describe the Measurement Cohort activities and offer participation in the study. Households will be visited up to 3 times to identify all eligible adults (≥15 years) for participation in the measurement cohorts. Consent forms will be translated into local languages and a copy will be provided to participants. Identifying information will be collected from each household member, including name, age, and gender. As described in Section 15.2.2, in both Uganda and Kenya children of the age represented in this study can obtain HIV testing services and HIV care without parental consent, and including parental accompaniment could significantly inhibit youth from accessing these services. The verbal consent form will be read to them in the local language, confirmation of consent will be documented as affirmation of their agreement to participate.

Written Consent for Hair Sample Collection Phase B: For the subset of those participants encountered during Measurement Outcome activities who are HIV-negative and who are currently on ARV-based prevention (e.g. PrEP or PEP) and selected for hair sample collection and testing for ARV drug levels, study staff will review the purpose of the hair collection and testing, the procedures for the hair collection and that the testing will be done at a later date and that the results will not be shared with them. Participants who are aged ≥15 to ≤17 years and are not deemed mature or independent minors by the governments of Uganda and Kenya will require parental co-consent on the same written consent document in order to participate. Participants will also be consented upon enrollment in the Phase A prevention trials for blood and hair sample collection (for HIV diagnostics, resistance testing, and PK) in a subsection of the Phase A Dynamic Prevention Intervention ICF.

Table 21. Consent Populations and Procedures

Study Population	Consent	Number of participants	Data Collection Tools
**Phase A Dynamic Prevention RCTs**	1. Written consents for PrEP/PEP RCTs in, antenatal/FP clinics, outpatient clinics, VHT households, and Youth multi-sector interventions	Approximately 2200 control/intervention participants	. CRFs, PrEP/PEP MoH records and logs, surveys

**Phase A Dynamic Treatment RCTs**	1. Written consent for Mobile Travel Pack RCT<br>2. Verbal consent for health and alcohol screening; written consent for Alcohol use RCT<br>1. Verbal consent for Hypertension screening; Written consent for Hypertension Linkage and treatment RCTs	1.Mobile study: up to 250 (control and intervention) participants<br>2. Alcohol study: up to 400 (control and intervention) participants<br>3. Hypertension linkage: up to 250 (control and intervention) participants<br>4. Hypertension treatment: Up to 200 (control and intervention) participants	CRFs, MoH care records and logs, surveys
**Behavioral measures, Phase A**	1. Written consent for survey on stigma, mobility, sexual behavior, and socioeconomic topics<br>2. Written consents for in-depth interviews and for focus group discussions	1. A subset of participants taking part in Phase A RCTs<br>2. A subset of participants and providers taking part in Phase A activities	1. Survey containing modules on stigma, mobility, sexual behavior, and socioeconomic topics<br>2. In-depth interview guides and focus group discussion guides
**Behavioral measures, Phase B**	Written consents for in-depth interviews	1. HIV-negative adults from drinking venues: 80<br>2. HIV-negative youth: 20-30<br>3. Adults with heavy alcohol use at risk for HIV: 120<br>4. HIV-positive adults undiagnosed/out of care: 20-30<br>5. HIV-positive youth undiagnosed/out of care: 20-30<br>6. VHTs: 8-12<br>7. Peers: 8-12	1. In-depth interview guides
**Community Mobilization (Community-Enhanced Reach)**	Verbal consent for testing activities	All residents ≥15 years in intervention communities	Testing data on tablets
**Dynamic Prevention Phase B – PrEP/PEP at HIV clinic , antenatal/family planning clinic and Youth Multi-Sector Interventions**	1. Written consent for enrollment in Dynamic Prevention	All participants enrolled PrEP/PEP/HIV-testing/CAB-LA programs in intervention communities (8 communities) who agree to take part	PrEP MoH records, PEP MoH records, data on tablets
**Person-Centered Treatment Delivery for Youth, Pregnant/Breastfeeding**	Written consent for enrollment at the clinic or venue where activities are taking place	Youth, pregnant/ breastfeeding women and/or unsuppressed/no VL in past year/newly HIV-diagnosed	MoH care records and data on tablets

**Women and Unsuppressed – Phase B**		who agree take part in LEAP activites	
**Severe Hypertension Treatment Telehealth – Phase B**	Written consent for enrollment in Severe Hypertension Telehealth	Eligible participants with severe hypertension who agree to take part	MoH care records
**Measurement Outcomes**	1. Verbal consent for Measurement Outome testing and data collection activies<br>2. Written consent for subset of PrEP/PEP recipients	1. All community residents – approximately 40,000 adults, 80,000 people total<br>2. Subset of PrEP/PEP recipients	Testing, survey, and MoH data

14.3.2. Assent Process

For participants aged ≥15 to ≤17 years taking part in youth-based surveys, interview guides and focus group discussions, and participants of this age who are taking PrEP or PEP and have agreed to hair sample collection for pharmacokinetic analysis, parental co-consent will be obtained on the same consent form that adults sign. Separate assent forms will not be used in this study. The consent discussion will take place with both the participant and one parent or guardian present, and the informed consent process will follow the guidelines described in Section 15.3.1.

As individuals over the age of 14 are allowed to independently consent for research in Kenya and Uganda, without the involvement of parents or guardians, if the research concerns the testing or treatment of a sexually transmitted disease. As noted above, the Uganda National Council for Science and Technology (UNCST) has determined that individuals aged ≥ 14 years are able to provide independent consent for research if they have a sexual transmitted infection, and the National AIDS and STI Control Programme (NASCOP) states that individuals aged ≥ 12 years are able to provide independent consent for HIV and STI screening and treatment while Kenya Medical Research Institute (KEMRI) allows mature minors to consent independently for research.

14.3.3. Documentation of Informed Consent

Documentation of written informed consent and verbal consent will be recorded on informed consent logs for all study activities. The logs will contain the participant’s study ID, clinic ID, initials, name, language of consent used, and whether the individual signed the document or provided a fingerprint in the case of written consents, or verbally affirmed their consent in the case of verbal consents.

14.3.4. Waiver of Informed Consent

A waiver of consent will be obtained from all IRBs for the collection of data from medical records related HIV, prevention and other disease care and prevention records, from participants in both intervention and control communities, as shown in Table 21.. The waiver of consent will meet the following criteria: (1) the research involves no more than minimal risk to the subjects; (2) the waiver of consent will not adversely affect the rights and welfare of the subjects; and (3) the research could not practicably be carried out without the waiver.

14.3.5. Stored Samples and Associated Data Considerations

Hair samples collected from HIV-negative members on PrEP modalities (oral PrEP, CAB LA, or dapivirine ring if available in-country) or PEP in Phase A and at month 30 in Phase B will be collected and stored for later measurements of ARV drug levels (e.g. TFV, CAB, or DPV), which will be conducted at the UCSF Hair Analytical Lab in the United States. Blood samples from persons who acquire HIV in Phase A prevention trials or Phase B HIV-negative members on PrEP modalities or PEP will be stored for additional testing for HIV diagnostics (if indicated), HIV drug resistance, and PrEP/PEP drug levels in-country or at the UCSF Hair Analytical Lab in the United States. Samples for PEth testing will be collected at Baseline and Year 3 in Phase B and stored until shipment to the United States Drug Testing Laboratory (USDTL) in the United States. All samples will labeled only with the participants' study ID number, initials, and the date of collection. A database that links these samples to other identifiers will be accessed only by study investigators and personnel and will be password protected.

All other biological samples collected during the study will be tested shortly after collection and any remaining specimen discarded.

14.4. Risks

Privacy: The primary risk to study participation is breach of privacy. Care will be taken to protect the privacy of participants and parents/guardians, as described in Section 14.8. However, there is a risk that others may inadvertently see patients' medical information, and thus their privacy may be compromised. Further details on the social impact of such potential privacy breaches are described in section 14.5.

Psychological Discomfort: There is the possibility of some psychological discomfort that may arise from questions and sensitive issues assessed in the stigma, sexual behavior, and youth life-skills and behaviors surveys and the in-depth qualitative interviews. We will train all study staff involved with surveys and qualitative interviews to recognize signs and symptoms of psychological discomfort and in the use of strategies to minimize such discomfort. This will include reminders that an individual may always choose to not answer any specific question that makes them uncomfortable or to stop surveys and interviews at any time. Referral resources for significant issues will also be made available to study staff should subject referrals to care be desired.

Venipuncture and fingerprick sample collection: Study sampling during testing activities will be done by venipuncture or fingerprick, depending on the assay. The total volume of blood taken per participant, including blood taken for standard health facility evaluations, is too small to for participants to experience any serious side-effects, although a mild and transient feeling of discomfort or bruising may occur at the site of sampling and bruising.

Ensuring Necessary Medical or Professional Intervention in the Event of Adverse Effects: Procedures will be put in place for the referral and care, free of charge, for necessary medical or professional intervention in the event of the unlikely occurrence of severe adverse effects. Care will be provided at local Ministry of Health care clinics or nearby referral hospitals as necessary.

14.5. Social Impact Events

Individuals enrolled in this study may experience personal problems resulting from the study participation. Such problems are termed social impact events. Although study sites will make every effort to protect participant privacy and confidentiality, it is possible that participants' involvement in the study could become known to others, and that participants may experience stigmatization, discrimination or, in rare cases, intimate partner violence as a result of being perceived as being HIV-infected or at risk for HIV infection. For example, participants could be treated unfairly, could have problems being accepted by their families and/or communities, or could experience abuse by their partner. Problems may also occur in circumstances in which study participation is not disclosed, such as impact on employment related to time taken for study visits.

In the event that a participant reports a social impact event, every effort will be made by study staff to provide appropriate assistance, and/or referrals to appropriate resources. Social impact events that are judged by the investigators to be serious, unexpected, or more severe or frequent than anticipated, will be reported to the relevant IRBs.

14.6. Benefits

Participants may receive no direct benefit to participation. Some participants may learn of their HIV status or receive diagnoses of other diseases through testing activities implemented by the study sooner than they would otherwise, or link to appropriate facilities for the treatment or prevention of diseases which might not have been known to them. HIV-negative individuals may benefit from the enhanced services in the Dynamic Prevention intervention. Youth, men and mobile populations may benefit from facilitated treatment options available in the Dynamic Treatment intervention, including increased or facilitated access to the clinic, counseling based on alcohol use or better outcomes for viral suppression related to the service delivery model. From a societal perspective, knowledge gained from this study could improve strategies for reducing HIV incidence and improving community health.

14.7. Compensation

Participants will not receive compensation or payment in this study for taking part in research activities, except those randomized to the travel voucher intervention who link to care within 30 days of screening for the Hypertension Linkage trial or those participating in in-depth interviews. For those individuals referred for social network health screening, transportation reimbursement will be provided upon linkage to the location of the health screening venue.

14.8. Participant Privacy and Confidentiality

Care will be taken to protect the privacy of participants and parents/guardians. Study participants will be identified only by their unique identification number on study documents except those maintained for contact information or consent documents. Participant study documents will be kept in individual files in secure filing cabinets in the study facilities. Testing results and sample and results transport documentation will be maintained in the local labs but will likewise not contain participant identifiers and will be accessed by study personnel only. After the study is over, all files containing personal identifiers such as participants' names, names of parents, guardians or relatives, and phone numbers and home locations will be destroyed.

In order to ensure data security and integrity, the following measures will be implemented:

- All members of the study team will be educated in the study protocol prior to the onset of the study.

- Detailed Standard Operating Procedures (SOPs) will be written for all project activities and be provided to relevant team members.
- Team members will be thoroughly trained on the SOP's.
- Where applicable, team members will receive additional training on the use of tablet computers.
- All data transcribed from paper will be double data entered or verified.
- All electronic data will be backed up regularly.
- All data will be transferred to the main Data Center in Kampala to the secure server. This sever is backed up on a daily basis and a monthly backup is stored off-site.
- All computers, including the tablets, will be password protected.
- All computers, including tablets, will be locked in a secure room each night.
- Log books and CRF's will be locked in a secure room each night.

14.9. Study Discontinuation

The study may be discontinued at any time by the IRB, NIAID, or other government entities as part of their duties to ensure that research participants are protected.

14.10. Community Advisory Board and Other Relevant Stakeholders

We will utilize a multilayered set of boards. These include: a) **stakeholder advisory board** consisting of representatives from the Uganda and Kenya Ministries of Health, IDRC and KEMRI research organizations, and PEPFAR implementing partners at the national and regional level, who will meet after Phase A is complete and prior to initiation of Phase B, and again at the conclusion of Phase B; b) formal **external scientific advisory board** meeting annually that includes experts in HIV prevention and treatment; experts in PrEP and youth in Uganda and Kenya; and experts in economics/development in East Africa; and c) **in-country advisory board** meeting annually with representation of MoH leads from HIV, non-communicable disease, the World Bank, PEPFAR implementing partners and HIV+ persons. Finally, **local community advisory boards** will provide invaluable input and communication with biannual meetings. We will have ongoing communication with the Kenya and Uganda National AIDS program, and with the PEPFAR implementing partners.

15. STATISTICAL ANALYSIS

This section provides an overview of the statistical considerations.

15.1 Overview

This is a two-phase study designed to first optimize interventions and then evaluate the population-level effects of combination intervention packages. In Phase A, we will conduct and analyze data from 6 individually randomized trials, one cluster randomized trial, and single arm pilot studies. We will evaluate Dynamic Prevention delivered in four settings (outpatient department, antenatal/family planning clinic, community (VHT) and youth multi-sector interventions) on prevention coverage among persons at risk of HIV acquisition. Two trials will evaluate Dynamic Treatment for two subpopulations (mobile persons and heavy drinkers) on viral suppression among HIV+ persons with or at risk of unsuppressed viral replication. Two trials will evaluate interventions (linkage and treatment) for adults with hypertension.

In Phase B, we will evaluate the population-level effects of a precision community health model, including community-enhanced reach, person-centered delivery, and data-enhanced precision, on HIV outcomes (HIV incidence, PrEP/PEP coverage, viral suppression, MTCT) and community health (mortality, TB, hypertension control, alcohol use, and pediatric immunization coverage).

Here, we describe the analytic approach for the primary outcomes in each trial. Analyses of secondary outcomes will be implemented analogously.

15.2 Individually randomized trials in Phase A

For all studies, the community-level inclusion/exclusion criteria, individual-level inclusion/exclusion criteria, recruitment process, study interventions, and study procedures have previously been described. Briefly in each trial, consenting participants will be randomized in a 1:1 ratio to the intervention or standard-of-care. Blocked randomization will be stratified on site and further stratified by sex for certain trials enrolling both sexes. All analyses of Dynamic Prevention in Phase A will restrict to participants enrolled before September 1, 2021.

The primary outcome for the individual-level prevention trials will be biomedical covered time defined as the proportion of follow-up time that the participant is protected from HIV infection with either PrEP or PEP. Follow-up time for the primary outcome will be over 48 weeks; follow-up time will be censored at HIV seroconversion, death, or withdrawal. Secondary and sensitivity analyses will examine alternative definitions of covered time, including restricting follow-up to periods of self-reported HIV risk.

The primary outcome for the individual-level treatment trials is an indicator of viral suppression (HIV RNA<400 cps/mL). The primary outcome is viral suppression at 48 weeks for the mobility intervention trial and 24 weeks for the alcohol intervention trial. In both trials, the primary analysis will exclude individuals who die or withdraw during follow-up; the alcohol intervention study will additionally exclude participants who move out of the study region. In the primary analysis, participants who do not have a viral load measurement within the ascertainment window will be assumed to be unsuppressed; secondary analyses will adjust for incomplete ascertainment of viral loads. Additional sensitivity analyses will assess the robustness of these analytic choices.

The primary outcome for the hypertension linkage trial is an indicator of linkage to hypertension care by 30 days. The primary definition will rely on clinical records; individuals without records will be assumed to never have linked. To assess the impact of the voucher, we will

examine, as a secondary outcome, linkage to the scheduled appointment. **The primary outcome for the hypertension treatment trial is blood pressure control (<140/90 mmHg) at 24 weeks**. The primary definition will rely on three repeated blood pressure measurements using standard procedures, with participants considered controlled if the average of the 2nd and 3rd measurements is <140/90 mmHg. In the primary analysis, participants who do not have their blood pressure measured within the ascertainment window will be assumed to be uncontrolled; secondary analyses will adjust for incomplete ascertainment of blood pressure measures.

In these trials, we will evaluate the interventions using targeted maximum likelihood estimation (TMLE), which provides precision and power gains over an unadjusted approach (e.g., the Student's t-test) by adjusting for stratification factors and for chance imbalance between randomized arms on additional baseline predictors of the outcome.[104] In secondary analyses, we will also implement an unadjusted estimator, the contrast of arm-specific average outcomes. In the prevention trials, we will test the null hypothesis of no changes in covered time due to the intervention with a two-sided test at the 5% significance level. In the treatment and linkage trials, we will test the null hypothesis of no improvements in outcomes due to the intervention with a one-sided test at the 5% significance level. We will also report point estimates and 95% confidence intervals for each effect measure and the arm-specific average outcomes.

15.2.1 Power calculations for individual-level trials

Power and sample size calculations for Phase A individual-level trials were based on standard formulas for a two-sample t-test (dynamic prevention) and two-sample test of proportions (dynamic treatment and linkage), using *power.t.test* and *power.prop.test* in *R*, respectively.[105] All input parameters were informed by SEARCH data, when possible. We expect these calculations to be conservative, because of the precision gained through stratified randomization and through covariate adjustment during the analysis.[104]

Since the prevention trials at antenatal clinics and outpatient departments have the same outcome (prevention coverage), we use the same sample size and power calculations for both. For each outcome, **Figure 4** provides the effect size, on the absolute scale, detected with 80% power varying the number of participants per arm and outcome under the standard-of-care with a two-sided hypothesis test at the 5% significance level.

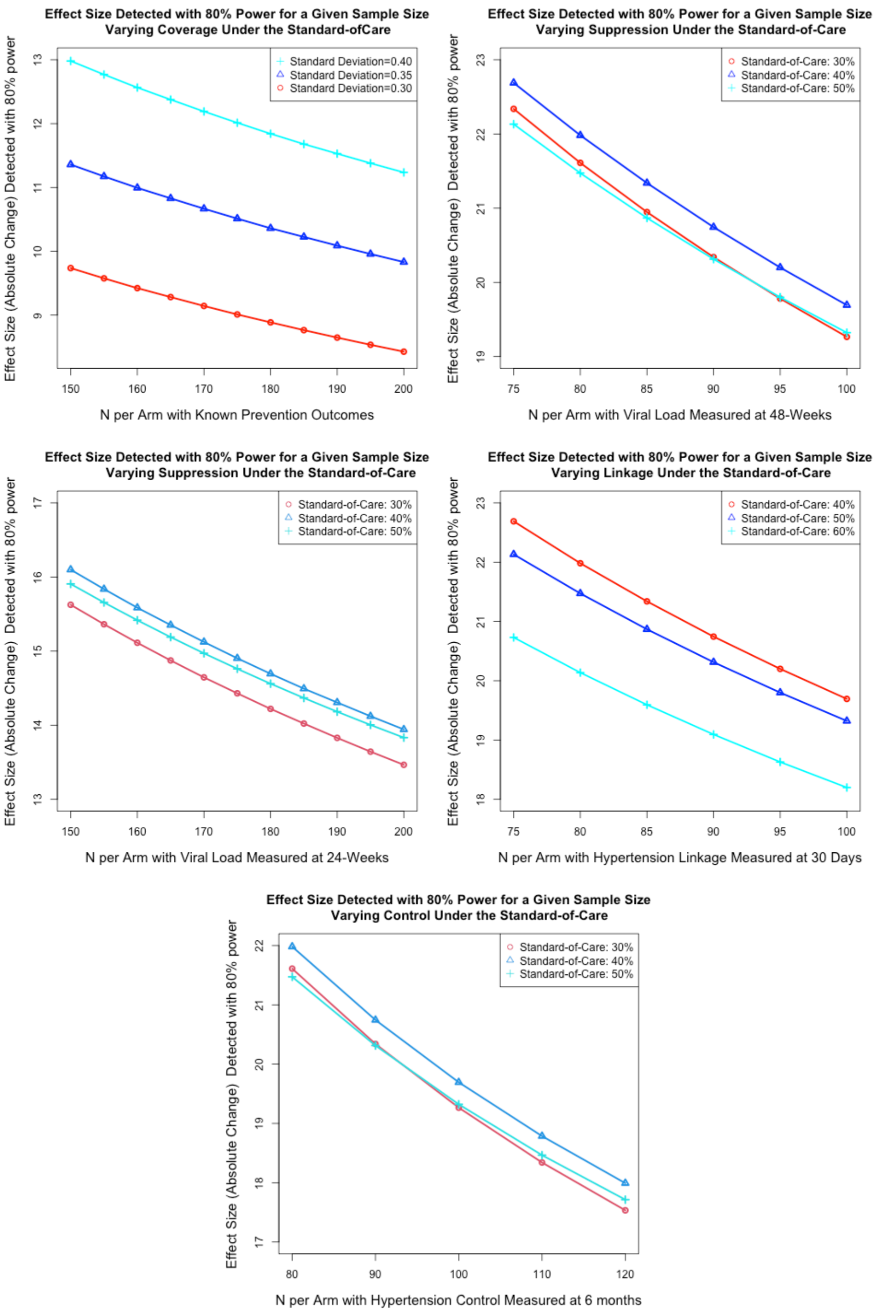

Figure 4: *Effect size (in percent improvement) detected with 80% power for a given sample size and outcome under the standard-of-care for the prevention trials at antenatal clinics and the outpatient department (top left), the mobility intervention trial (top right), alcohol intervention trial (middle left), the HTN linkage trial (middle right), and the HTN treatment trial (bottom). For example, an effect size of 10% corresponds with an increase in prevention coverage from 30% to 40%.*

For the individually randomized prevention trials (delivered at the antenatal clinic and outpatient department), we anticipate having 80% power to detect at least a 9.8% absolute increase in prevention coverage with a standard deviation of 0.35 and 200 participants/arm. Even with 25% attrition (from 200 to 150 participants/arm) and higher than expected variability (e.g., standard deviation=0.40), these calculations suggest we would be well-powered to detect at least a 13%

absolute increase in prevention coverage. To ensure sufficient power, we will thus enroll at least 400 participants per prevention trial for measurement of the primary endpoint.

For the mobility trial, we anticipate having 80% power to detect at least a 19.7% absolute increase in viral suppression at 48 weeks from 40% under the standard-of-care and with 100 participants/arm. Even with 25% attrition (from 100 to 75 participants/arm) and lower or higher suppression under the standard-of-care, these calculations suggest we would be well-powered to detect at least a 22.3% absolute increase in suppression. To ensure sufficient power, we will thus enroll at least 200 participants in the trial.

For the alcohol trial, we anticipate having 80% power to detect at least a 14% absolute increase in viral suppression at 24 weeks from 40% under the standard-of-care and with 200 participants/arm. Even with 25% attrition (from 200 to 150 participants/arm) and lower or higher suppression under the standard-of-care, these calculations suggest we would be well-powered to detect at least a 16% absolute increase in suppression. To ensure sufficient power, we will thus enroll at least 400 participants in the trial.

For the hypertension linkage trial, we anticipate having 80% power to detect at least a 19.3% absolute increase in linkage from 50% under the standard-of-care and with 100 participants/arm. Even with 25% fewer participants enrolled (from 100 to 75 participants/arm) and lower or higher linkage under the standard-of-care, these calculations suggest we would be well-powered to detect at least a 22.7% absolute increase in linkage. To ensure sufficient power, we will thus enroll at least 200 participants in the trial. For the hypertension treatment trial, we anticipate having 80% power to detect at least a 20% absolute increase in hypertension control from 40% under the standard-of-care (estimated based on SEARCH) with 100 participants/arm. Even with 20% fewer participants enrolled (from 100 to 80 participants/arm) and lower or higher control under the standard-of-care, these calculations suggest we would be well-powered to detect at least a 22% absolute increase in control. To ensure sufficient power, we will thus enroll at least 160 participants (and a maximum of 200 participants) in the trial.

15.3 Cluster randomized trials for Phase A

For the VHT cluster randomized trial (CRT), the community-level inclusion/exclusion criteria, individual-level inclusion/exclusion criteria, recruitment process, study interventions, and study procedures have previously been described. Briefly, N=16 villages (served by VHTs) will be randomized in a 1:1 ratio to the intervention or standard-of-care, after matching on country. Within each cluster, participants will be consented and enrolled in the measurement cohort if HIV-uninfected and at risk of HIV acquisition. Analyses of the VHT trial will restrict to participants enrolled before September 1, 2021.

The **primary outcome for the cluster-level prevention trial will be biomedical covered time,** defined as in the individually randomized prevention trials. As before, follow-up time for the primary outcome will be over 48 weeks; follow-up time will be censored at HIV seroconversion, death, or withdrawal. Secondary and sensitivity analyses will examine alternative definitions of covered time, including restricting follow-up to periods of self-reported HIV risk.

Prevention coverage will be compared between arms using a TMLE, adapted to appropriately account for the dependence of outcomes within clusters.[5, 106 107] Given the limited number of clusters, we will use Adaptive Pre-specification to optimally adjust for covariate imbalance between randomized arms, while preserving Type-I error control.[108] In secondary analyses, we will implement the unadjusted estimator as the contrast of the average arm-specific outcomes. We will

test the **null hypothesis** that the Dynamic Prevention did not improve prevention coverage, as compared to standard-of-care, with a two-sided test at the 5% significance level.

15.3.1 Power calculations for the cluster-level trial

Figure 3 provides the sample size and power calculations for the VHT trial based on standard formulas for CRTs (Chapter 7 of Hayes and Moulton).[107] All input parameters were informed by SEARCH data, when possible. We expect these calculations to be conservative, because of the precision gained through stratified randomization and through covariate adjustment during the analysis.

For Dynamic Prevention delivered via VHTs, we anticipate an average (harmonic mean) 20 HIV-uninfected cohort members per cluster. Assuming 10% coverage under the standard-of-care, a standard deviation of 0.35, and matched coefficient of variation of *km*=0.25, we anticipate 80% power to detect at least a 15% absolute increase in prevention coverage with N=16 clusters (N=8 clusters/arm). Even with 20% fewer participants (from 20 to 16 participants/cluster), these calculations suggest we would be well-powered to detect at least a 16.5% absolute increase in prevention coverage.

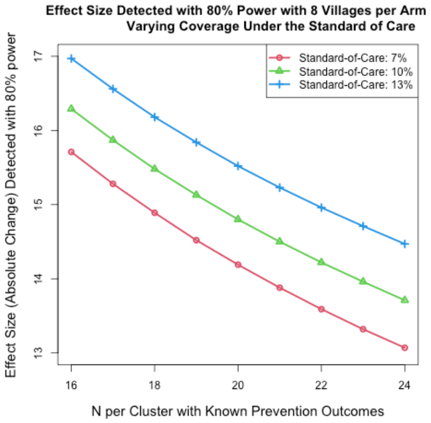

Figure 5: *For the cluster-level prevention trial, the effect size (in percent improvement) detected with 80% power for a given cluster size N=16 villages total. Calculations assume a standard deviation of 0.35, a matched coefficient of variation of km=0.25 (informed by SEARCH data) and vary coverage under the standard-of-care.*

15.4 Additional analyses to optimize dynamic prevention and treatment

For each trial, we will assess the fidelity to intervention implementation, intervention adoption, and process outcomes. We will also examine feasibility and prevention coverage in the youth multisectoral intervention pilot studies.

To understand effect heterogeneity, we will repeat the analyses outlined above within the following pre-specified subgroups: country, sex (for trials enrolling both sexes), age group (for trials enrolling adults of all ages), and may include mobility (for trials enrolling both mobile and non-mobile adults).

For each of the trials, we will provide descriptive statistics of populations and outcomes, overall as well as stratified by intervention arm and country. We will further evaluate predictors of sub-optimal outcomes using TMLE (primary) and with univariate associations (secondary). For interventions with suggested efficacy, we may examine pathways of intervention action using formal mediation analyses.[109, 110] These analyses will be complemented with participant and provider qualitative data and mixed method approaches.

Finally, to quantify the potential spillover between randomized arms and to understand secular trends in the clinic-based intervention trials for Dynamic Prevention (outpatient department and antenatal/family planning clinic) and for Dynamic Treatment (mobile populations and heavy drinkers), we may conduct retrospective record review to obtain endpoint measures before the trials' initiation and compare them with endpoints measured during the trials' follow-up through a pre-post analysis. Additionally, we may use difference-in-differences to compare each trial's intervention arm and each trial's control arm to the external controls.[111]

15.5 Community randomized trial in Phase B

In Phase B, we will randomize the *N=16* study communities pair-matched on predictors of the primary endpoint (HIV incidence) and stratified by country.

We will assess population-level effects with Two-Stage TMLE, an approach appropriate for cluster randomized trials.[5, 106] In Stage I, we estimate the outcome for each community. Then in Stage II, we use a cluster-level TMLE to estimate intervention effectiveness by comparing the community-specific endpoints between randomized arms. Given the limited number of clusters, we will use Adaptive Pre-specification to optimally adjust for covariate imbalance between randomized arms, while preserving Type-I error control.[108] In secondary analyses, we will implement the unadjusted estimator as the contrast of the arm-specific mean outcomes. For each endpoint, we will test the **null hypothesis** of *no improvement* from the intervention with a one-sided test at the 5% significance level. We will also report point estimates and two-sided 95% confidence intervals for each effect measure and the arm-specific mean outcomes.

The **primary study outcome** is HIV incidence among adult community members and assessed at month 30 with a recency assay. Stage I estimates of HIV incidence (per 100 person-years) will account for assay characteristics, such as mean duration of recent infection (MDRI) and false recent rate (FRR). Stage II estimates will use leave-one-out cross-validation to select from the following community-level covariates the combination that maximizes empirical efficiency: baseline HIV prevalence, baseline proportion with HIV who are suppressing viral suppression (<400 c/mL), or nothing (unadjusted).

The following secondary outcomes will be compared between arms using the analogous two-stage approach:

- **Biomedical prevention coverage:** proportion of HIV-negative follow-up time during which the participant was protected from HIV by PrEP or PEP (inclusive of CAB-LA)
- **Population-level viral suppression:** proportion of adults with HIV who are suppressinv viral suppression (HIV RNA level < 400 c/mL) at month 30
- **Mortality:** the incidence (per 100 person-years) of death to illness and incidence of death due to all causes among adults
- **Annual incidence of active TB disease**, overall and stratified by age group and HIV-association
- **Hypertension control** (BP<140/90 mmHg) among community members aged 30+ years at month 30
- **Prevalence of heavy alcohol use** (AUDIT-C value of 3+ among women and 4+ among men) among persons with HIV and age 18+ years at month 30
- **Infant HIV-free survival**: the proportion of infants boarn to women with HIV or HIV-unknown status who are alive and without HIV at month 30

  Pediatric immunization coverage: the proportion of children (aged < 5 years) with up-to-date vaccination at month 30

15.5.1 Power calculations for Phase B

For primary endpoints of HIV incidence, we present the following power calculations based on *inctools R* package,[1] which implements and extends the HIV incidence methods of Kassanjee et al.[112] The *R* package is a more robust implementation of the prior ABIE (Assay Based Incidence Estimation) spreadsheet tool, developed by the CEPHIA (Consortium for the Evaluation and Performance of HIV Incidence Assays). When possible, we used prior data to inform key parameters, including (i)community size, (ii) HIV prevalence, (iii) expected testing coverage, (iv) expected outcome under the standard-of-care, and (v) recency assay characteristics.[2, 3] We expect these calculations to be conservative, because of the precision gained through our pre-specified choice of a one-sided hypothesis test and through covariate adjustment during the analysis.[5, 104, 106]

With 16 communities (8 per arm) each with ~5000 adults, 10% HIV prevalence, and 90% testing coverage, we anticipate being powered at 80% to detect a 40% relative reduction in annual HIV incidence from 0.5% in the standard-of-care (control) with a two-sided hypothesis test (α=0.05), a design effect of 1.3, mean duration of recent infection (MDRI) of 238, and false recent rate (FRR) of 0%. As shown in **Figure 6**, these calculations are fairly robust to the test coverage achieved and HIV incidence under the standard-of-care.

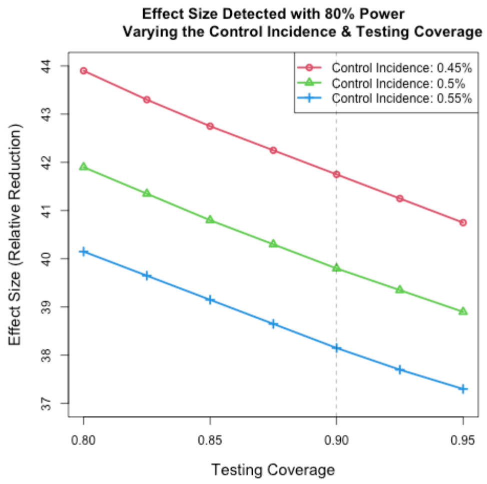

Figure 6: *For the Phase B cluster randomized trial, the effect size (in relative reduction in HIV incidence) detected with 80% power varying testing coverage and HIV incidence under the standard-of-care (control).*

15.6 Additional explanatory and descriptive analyses for Phase B

For the 16 communities participating in Phase B, we will provide descriptive statistics of the baseline characteristics of study participants, overall and stratified by country. Characteristics reported will include sex, age, marital status (single, married, widowed, divorced/separated), education (no school, primary, secondary, post-secondary), occupation, and mobility. For each study outcome compared between arms, we will also report formal consort diagrams as well as participant flow diagrams enumerating inclusions and exclusions for all clusters and individuals contributing to each analysis.

Implementation evaluation will be based on the RE-AIM framework. We will also conduct multivariable analyses to evaluate risk factors for poor outcomes, including viral non-suppression, low prevention coverage among persons at risk of HIV, and HIV seroconversion. Variable importance measures will be estimated with a pooled individual-level TMLE with the community

treated as the independent unit.[106] We will implement analogous predictor analyses for other community health outcomes.

To inform our understanding of mechanisms of action, we may specify hierarchical causal models to represent the relationships between key study variables, including the community-level intervention, community-level mediators, individual-level mediators, and individual-level outcomes.[104, 109, 110] We may assess mediation at the community-level, using methods analogous to the individual-level mediation analyses. We may augment these cluster-level analyses with multi-level mediation analyses.

16. PUBLICATION POLICY

The findings from this study may be published in a medical journal. No individual identities will be used in any reports or publications resulting from the study. The researchers will publish results of the study in accordance with NIH, UCSF, UNCST, KEMRI and Makerere University guidelines.

17. REFERENCES

1. Grebe E, Welte A, A. M, Bäumler P, Kassanjee R, Brand H, Van Schalkwyk C, Yuruo L, Daniel S, Ongarello S, Asai Y, Eaton J. inctools: Incidence Estimation Tools. R package version 1.0.15 ed. CRAN2019. <https://CRAN.R-project.org/package=inctools>.
2. Klock E, Wilson E, Fernandez RE, Piwowar-Manning E, Moore A, Kosloff B, Bwalya J, Bell-Mandla N, James A, Ayles H, Bock P, Donnell D, Fidler S, Hayes R, Eshleman SH, Laeyendecker O, team Hs. Validation of population-level HIV-1 incidence estimation by cross-sectional incidence assays in the HPTN 071 (PopART) trial. J Int AIDS Soc. 2021;24(12):e25830. Epub 2021/12/14. doi: 10.1002/jia2.25830. PubMed PMID: 34897992; PMCID: PMC8666582.
3. Laeyendecker O, Konikoff J, Morrison DE, Brookmeyer R, Wang J, Celum C, Morrison CS, Abdool Karim Q, Pettifor AE, Eshleman SH. Identification and validation of a multi-assay algorithm for cross-sectional HIV incidence estimation in populations with subtype C infection. J Int AIDS Soc. 2018;21(2). Epub 2018/03/01. doi: 10.1002/jia2.25082. PubMed PMID: 29489059; PMCID: PMC5829581.
4. Global AIDS Update 2019: Communities at the centre. UNAIDS, 2019 2019/07/16/. Report No.
5. Havlir DV, Balzer LB, Charlebois ED, Clark TD, Kwarisiima D, Ayieko J, Kabami J, Sang N, Liegler T, Chamie G, Camlin CS, Jain V, Kadede K, Atukunda M, Ruel T, Shade SB, Ssemmondo E, Byonanebye DM, Mwangwa F, Owaraganise A, Olilo W, Black D, Snyman K, Burger R, Getahun M, Achando J, Awuonda B, Nakato H, Kironde J, Okiror S, Thirumurthy H, Koss C, Brown L, Marquez C, Schwab J, Lavoy G, Plenty A, Mugoma Wafula E, Omanya P, Chen YH, Rooney JF, Bacon M, van der Laan M, Cohen CR, Bukusi E, Kamya MR, Petersen M. HIV Testing and Treatment with the Use of a Community Health Approach in Rural Africa. The New England journal of medicine. 2019;381(3):219-29. Epub 2019/07/18. doi: 10.1056/NEJMoa1809866. PubMed PMID: 31314966.
6. Iwuji CC, Orne-Gliemann J, Larmarange J, Balestre E, Thiebaut R, Tanser F, Okesola N, Makowa T, Dreyer J, Herbst K, McGrath N, Barnighausen T, Boyer S, De Oliveira T, Rekacewicz C, Bazin B, Newell ML, Pillay D, Dabis F, Group ATS. Universal test and treat and the HIV epidemic in rural South Africa: a phase 4, open-label, community cluster randomised trial. Lancet HIV. 2018;5(3):e116-e25. doi: 10.1016/S2352-3018(17)30205-9. PubMed PMID: 29199100.
7. Hayes RJ, Donnell D, Floyd S, Mandla N, Bwalya J, Sabapathy K, Yang B, Phiri M, Schaap A, Eshleman SH, Piwowar-Manning E, Kosloff B, James A, Skalland T, Wilson E, Emel L, Macleod D, Dunbar R, Simwinga M, Makola N, Bond V, Hoddinott G, Moore A, Griffith S, Deshmane Sista N, Vermund SH, El-Sadr W, Burns DN, Hargreaves JR, Hauck K, Fraser C, Shanaube K, Bock P, Beyers N, Ayles H, Fidler S, Team HS. Effect of Universal Testing and Treatment on HIV Incidence - HPTN 071 (PopART). The New England journal of medicine. 2019;381(3):207-18. Epub 2019/07/18. doi: 10.1056/NEJMoa1814556. PubMed PMID: 31314965; PMCID: PMC6587177.
8. Makhema J, Wirth KE, Pretorius Holme M, Gaolathe T, Mmalane M, Kadima E, Chakalisa U, Bennett K, Leidner J, Manyake K, Mbikiwa AM, Simon SV, Letlhogile R, Mukokomani K, van Widenfelt E, Moyo S, Lebelonyane R, Alwano MG, Powis KM, Dryden-Peterson SL, Kgathi C, Novitsky V, Moore J, Bachanas P, Abrams W, Block L, El-Halabi S, Marukutira T, Mills LA, Sexton C, Raizes E, Gaseitsiwe S, Bussmann H, Okui L, John O, Shapiro RL, Pals S, Michael H, Roland M, DeGruttola V, Lei Q, Wang R, Tchetgen Tchetgen E, Essex M, Lockman S. Universal Testing, Expanded Treatment, and

Incidence of HIV Infection in Botswana. The New England journal of medicine. 2019;381(3):230-42. Epub 2019/07/18. doi: 10.1056/NEJMoa1812281. PubMed PMID: 31314967.
9. 90-90-90. An ambitious treatment target to help end the AIDS epidemic. UNAIDS. 2014.
10. UGANDA POPULATION-BASED HIV IMPACT ASSESSMENT. UPHIA 2016–2017. Summary Sheet: Preliminary Findings. August 2017. Available at: https://www.afro.who.int/sites/default/files/2017-08/UPHIA%20Uganda%20factsheet.pdf. 2017.
11. Sovershaeva E, Shamu T, Wilsgaard T, Bandason T, Flaegstad T, Katzenstein D, Ferrand RA, Odland J. Patterns of detectable viraemia among children and adults with HIV infection taking antiretroviral therapy in Zimbabwe. Int J Infect Dis. 2019;78:65-71. doi: 10.1016/j.ijid.2018.10.019. PubMed PMID: 30391420.
12. ZIMBABWE POPULATION-BASED HIV IMPACT ASSESSMENT. ZIMPHIA 2015–2016. Summary Sheet: Preliminary Findings. December 2016. Accessed at: https://phia.icap.columbia.edu/wp-content/uploads/2016/11/ZIMBABWE-Factsheet.FIN_.pdf.
13. Petersen M, Balzer L, Kwarsiima D, Sang N, Chamie G, Ayieko J, Kabami J, Owaraganise A, Liegler T, Mwangwa F, Kadede K, Jain V, Plenty A, Brown L, Lavoy G, Schwab J, Black D, van der Laan M, Bukusi EA, Cohen CR, Clark TD, Charlebois E, Kamya M, Havlir D. Association of Implementation of a Universal Testing and Treatment Intervention With HIV Diagnosis, Receipt of Antiretroviral Therapy, and Viral Suppression in East Africa. JAMA. 2017;317(21):2196-206. doi: 10.1001/jama.2017.5705. PubMed PMID: 28586888; PMCID: PMC5734234.
14. Grobler A, Cawood C, Khanyile D, Puren A, Kharsany ABM. Progress of UNAIDS 90-90-90 targets in a district in KwaZulu-Natal, South Africa, with high HIV burden, in the HIPSS study: a household-based complex multilevel community survey. Lancet HIV. 2017;4(11):e505-e13. doi: 10.1016/S2352-3018(17)30122-4. PubMed PMID: 28779855.
15. Huerga H, Van Cutsem G, Ben Farhat J, Puren A, Bouhenia M, Wiesner L, Dlamini L, Maman D, Ellman T, Etard JF. Progress towards the UNAIDS 90-90-90 goals by age and gender in a rural area of KwaZulu-Natal, South Africa: a household-based community cross-sectional survey. BMC public health. 2018;18(1):303. doi: 10.1186/s12889-018-5208-0. PubMed PMID: 29499668; PMCID: PMC5833029.
16. Floyd S, Ayles H, Schaap A, Shanaube K, MacLeod D, Phiri M, Griffith S, Bock P, Beyers N, Fidler S, Hayes R, Team HS. Towards 90-90: Findings after two years of the HPTN 071 (PopART) cluster-randomized trial of a universal testing-and-treatment intervention in Zambia. PLoS ONE. 2018;13(8):e0197904. doi: 10.1371/journal.pone.0197904. PubMed PMID: 30096139; PMCID: PMC6086421.
17. Larmarange J, Diallo MH, McGrath N, Iwuji C, Plazy M, Thiebaut R, Tanser F, Barnighausen T, Pillay D, Dabis F, Orne-Gliemann J, Group ATS. The impact of population dynamics on the population HIV care cascade: results from the ANRS 12249 Treatment as Prevention trial in rural KwaZulu-Natal (South Africa). J Int AIDS Soc. 2018;21 Suppl 4:e25128. doi: 10.1002/jia2.25128. PubMed PMID: 30027600; PMCID: PMC6053480.
18. Edwards JK, Arimi P, Ssengooba F, Mulholland G, Markiewicz M, Bukusi EA, Orikiiriza JT, Virkud A, Weir S. The HIV care continuum among resident and non-resident populations found in venues in East Africa cross-border areas. J Int AIDS Soc.

2019;22(1):e25226. doi: 10.1002/jia2.25226. PubMed PMID: 30675984; PMCID: PMC6344908.
19. Puryear SB, Balzer LB, Ayieko J, Kwarisiima D, Hahn JA, Charlebois ED, Clark TD, Cohen CR, Bukusi EA, Kamya MR, Petersen ML, Havlir DV, Chamie G. Associations between alcohol use and HIV care cascade outcomes among adults undergoing population-based HIV testing in East Africa. AIDS (London, England). 2019. Epub 2019/11/15. doi: 10.1097/QAD.0000000000002427. PubMed PMID: 31725431.
20. Velloza J, Kemp CG, Aunon FM, Ramaiya MK, Creegan E, Simoni JM. Alcohol Use and Antiretroviral Therapy Non-Adherence Among Adults Living with HIV/AIDS in Sub-Saharan Africa: A Systematic Review and Meta-Analysis. AIDS Behav. 2019. Epub 2019/11/02. doi: 10.1007/s10461-019-02716-0. PubMed PMID: 31673913.
21. San Francisco HIV Epidemiology: Annual Report 2017. San Francisco Department of Public Health, Population Health Division. HIV Epidemiology Section. 2018.
22. Grulich AE, Guy R, Amin J, Jin F, Selvey C, Holden J, Schmidt HA, Zablotska I, Price K, Whittaker B, Chant K, Cooper C, McGill S, Telfer B, Yeung B, Levitt G, Ogilvie EE, Dharan NJ, Hammoud MA, Vaccher S, Watchirs-Smith L, McNulty A, Smith DJ, Allen DM, Baker D, Bloch M, Bopage RI, Brown K, Carr A, Carmody CJ, Collins KL, Finlayson R, Foster R, Jackson EY, Lewis DA, Lusk J, O'Connor CC, Ryder N, Vlahakis E, Read P, Cooper DA, Expanded Pr EPIiCNSWrg. Population-level effectiveness of rapid, targeted, high-coverage roll-out of HIV pre-exposure prophylaxis in men who have sex with men: the EPIC-NSW prospective cohort study. Lancet HIV. 2018;5(11):e629-e37. doi: 10.1016/S2352-3018(18)30215-7. PubMed PMID: 30343026.
23. https://www.prepwatch.org/resource/global-prep-tracker/. Last accessed April 2019. 2019.
24. Hodges-Mameletzis I, Dalal S, Msimanga-Radebe B, Rodolph M, Baggaley R. Going global: the adoption of the World Health Organization's enabling recommendation on oral pre-exposure prophylaxis for HIV. Sex Health. 2018;15(6):489-500. doi: 10.1071/SH18125. PubMed PMID: 30496718.
25. Koss CA, Charlebois ED, Ayieko J, Kwariisima D, Kabami J, Balzer LB, Atukunda M, Mwangwa F, Peng J, Mwinike Y, Owaraganise A, Chamie G, Jain V, Sang N, Olilo W, Brown LB, Marquez C, Zhang K, Ruel TD, Camlin CS, Rooney J, Black D, Clark TD, Gandhi M, Cohen CR, Bukusi EA, Petersen ML, Kamya M, Havlir D. Uptake, engagement, and adherence to pre-exposure prophylaxis offered after population HIV testing in rural Kenya and Uganda: 72 week interim observational data from the SEARCH trial. The Lancet HIV. 2020:(in press).
26. Jennings L, Mathai M, Linnemayr S, Trujillo A, Mak'anyengo M, Montgomery BEE, Kerrigan DL. Economic Context and HIV Vulnerability in Adolescents and Young Adults Living in Urban Slums in Kenya: A Qualitative Analysis Based on Scarcity Theory. AIDS Behav. 2017;21(9):2784-98. doi: 10.1007/s10461-017-1676-y. PubMed PMID: 28078495; PMCID: PMC5505815.
27. MacCarthy S, Saya U, Samba C, Birungi J, Okoboi S, Linnemayr S. "How am I going to live?": exploring barriers to ART adherence among adolescents and young adults living with HIV in Uganda. BMC Public Health. 2018;18(1):1158. doi: 10.1186/s12889-018-6048-7. PubMed PMID: 30286746; PMCID: PMC6172755.
28. Nash K, O'Malley G, Geoffroy E, Schell E, Bvumbwe A, Denno DM. "Our girls need to see a path to the future" --perspectives on sexual and reproductive health information among adolescent girls, guardians, and initiation counselors in Mulanje district, Malawi.

Reprod Health. 2019;16(1):8. doi: 10.1186/s12978-018-0661-x. PubMed PMID: 30683127; PMCID: PMC6347744.
29. Yakubu I, Salisu WJ. Determinants of adolescent pregnancy in sub-Saharan Africa: a systematic review. Reprod Health. 2018;15(1):15. doi: 10.1186/s12978-018-0460-4. PubMed PMID: 29374479; PMCID: PMC5787272.
30. Mani A, Mullainathan S, Shafir E, Zhao J. Poverty impedes cognitive function. Science (New York, NY. 2013;341(6149):976-80. doi: 10.1126/science.1238041. PubMed PMID: 23990553.
31. Haushofer J, Fehr E. On the psychology of poverty. Science (New York, NY. 2014;344(6186):862-7. doi: 10.1126/science.1232491. PubMed PMID: 24855262.
32. Shah AK, Mullainathan S, Shafir E. Some consequences of having too little. Science (New York, NY. 2012;338(6107):682-5. doi: 10.1126/science.1222426. PubMed PMID: 23118192.
33. Oshri A, Hallowell E, Liu S, MacKillop J, Galvan A, Kogan SM, Sweet LH. Socioeconomic hardship and delayed reward discounting: Associations with working memory and emotional reactivity. Dev Cogn Neurosci. 2019;37:100642. doi: 10.1016/j.dcn.2019.100642. PubMed PMID: 31004982.
34. Camlin CS, Ssemmondo E, Chamie G, El Ayadi AM, Kwarisiima D, Sang N, Kabami J, Charlebois E, Petersen M, Clark TD, Bukusi EA, Cohen CR, M RK, Havlir D, Collaboration S. Men "missing" from population-based HIV testing: insights from qualitative research. AIDS Care. 2016;28 Suppl 3:67-73. doi: 10.1080/09540121.2016.1164806. PubMed PMID: 27421053.
35. Brown LB, Ayieko J, Mwangwa F, Owaraganise A, Kwarisiima D, Jain V, Ruel T, Clark T, Black D, Chamie G, Bukusi EA, Cohen CR, Kamya MR, Petersen ML, Charlebois ED, Havlir DV. Predictors of Retention in HIV Care Among Youth (15-24) in a Universal Test-and-Treat Setting in Rural Kenya. J Acquir Immune Defic Syndr. 2017;76(1):e15-e8. doi: 10.1097/QAI.0000000000001390. PubMed PMID: 28394821; PMCID: PMC5828506.
36. Hoddinott G, Myburgh H, de Villiers L, Ndubani R, Mantantana J, Thomas A, Mbewe M, Ayles H, Bock P, Seeley J, Shanaube K, Hargreaves J, Bond V, Reynolds L, Team HS. Households, fluidity, and HIV service delivery in Zambia and South Africa - an exploratory analysis of longitudinal qualitative data from the HPTN 071 (PopART) trial. J Int AIDS Soc. 2018;21 Suppl 4:e25135. doi: 10.1002/jia2.25135. PubMed PMID: 30027687; PMCID: PMC6053477.
37. Eshun-Wilson I, Rohwer A, Hendricks L, Oliver S, Garner P. Being HIV positive and staying on antiretroviral therapy in Africa: A qualitative systematic review and theoretical model. PLoS ONE. 2019;14(1):e0210408. doi: 10.1371/journal.pone.0210408. PubMed PMID: 30629648; PMCID: PMC6328200.
38. Mavegam BO, Pharr JR, Cruz P, Ezeanolue EE. Effective interventions to improve young adults' linkage to HIV care in Sub-Saharan Africa: a systematic review. AIDS Care. 2017;29(10):1198-204. doi: 10.1080/09540121.2017.1306637. PubMed PMID: 28325077.
39. Green LW, Krueter M. Health Program Planning - An Educational and Ecological Approach. Philadelphia: McGraw-Hill; 2005.
40. Kennedy CE, Fonner VA, O'Reilly KR, Sweat MD. A systematic review of income generation interventions, including microfinance and vocational skills training, for HIV prevention. AIDS Care. 2014;26(6):659-73. doi: 10.1080/09540121.2013.845287. PubMed PMID: 24107189; PMCID: PMC3943565.
41. Bermudez LG, Ssewamala FM, Neilands TB, Lu L, Jennings L, Nakigozi G, Mellins CA, McKay M, Mukasa M. Does Economic Strengthening Improve Viral Suppression

Among Adolescents Living with HIV? Results From a Cluster Randomized Trial in Uganda. AIDS Behav. 2018;22(11):3763-72. doi: 10.1007/s10461-018-2173-7. PubMed PMID: 29846836; PMCID: PMC6204092.
42. Harrison A, Colvin CJ, Kuo C, Swartz A, Lurie M. Sustained High HIV Incidence in Young Women in Southern Africa: Social, Behavioral, and Structural Factors and Emerging Intervention Approaches. Curr HIV/AIDS Rep. 2015;12(2):207-15. doi: 10.1007/s11904-015-0261-0. PubMed PMID: 25855338; PMCID: PMC4430426.
43. Dunbar MS, Kang Dufour MS, Lambdin B, Mudekunye-Mahaka I, Nhamo D, Padian NS. The SHAZ! project: results from a pilot randomized trial of a structural intervention to prevent HIV among adolescent women in Zimbabwe. PLoS One. 2014;9(11):e113621. doi: 10.1371/journal.pone.0113621. PubMed PMID: 25415455; PMCID: PMC4240618.
44. Bandiera O, Buehren N, Burgess R, Goldstein M, Gulesci S, Rasul I, Sulaiman M. Empowering Adolescent Girls : Evidence from a Randomized Control Trial in Uganda. World Bank, Washington, DC. © World Bank. https://openknowledge.worldbank.org/handle/10986/25529 License: CC BY 3.0 IGO.2012.
45. Gibbs A, Jacobson J, Kerr Wilson A. A global comprehensive review of economic interventions to prevent intimate partner violence and HIV risk behaviours. Glob Health Action. 2017;10(sup2):1290427. doi: 10.1080/16549716.2017.1290427. PubMed PMID: 28467193; PMCID: PMC5645712.
46. Pronyk PM, Kim JC, Abramsky T, Phetla G, Hargreaves JR, Morison LA, Watts C, Busza J, Porter JD. A combined microfinance and training intervention can reduce HIV risk behaviour in young female participants. AIDS. 2008;22(13):1659-65. doi: 10.1097/QAD.0b013e328307a040. PubMed PMID: 18670227.
47. Saul J, Bachman G, Allen S, Toiv NF, Cooney C, Beamon T. The DREAMS core package of interventions: A comprehensive approach to preventing HIV among adolescent girls and young women. PLoS ONE. 2018;13(12):e0208167. doi: 10.1371/journal.pone.0208167. PubMed PMID: 30532210; PMCID: PMC6285267.
48. The United States President’s Emergency Plan for AIDS Relief (PEPFAR): 2019 Annual Report to Congress. 2019.
49. Koss CA, Ayieko J, Mwangwa F, Owaraganise A, Kwarisiima D, Balzer LB, Plenty A, Sang N, Kabami J, Ruel TD, Black D, Camlin CS, Cohen CR, Bukusi EA, Clark TD, Charlebois ED, Petersen ML, Kamya MR, Havlir DV, study S. Early Adopters of HIV Preexposure Prophylaxis in a Population-based Combination Prevention Study in Rural Kenya and Uganda. Clin Infect Dis. 2018. doi: 10.1093/cid/ciy390. PubMed PMID: 29741594.
50. Eakle R, Venter F, Rees H. Pre-exposure prophylaxis (PrEP) in an era of stalled HIV prevention: Can it change the game? Retrovirology. 2018;15(1):29. doi: 10.1186/s12977-018-0408-3. PubMed PMID: 29609619; PMCID: PMC5879931.
51. Haberer JE. Current concepts for PrEP adherence in the PrEP revolution: from clinical trials to routine practice. Curr Opin HIV AIDS. 2016;11(1):10-7. doi: 10.1097/COH.0000000000000220. PubMed PMID: 26633638; PMCID: PMC4801217.
52. Lindberg L, Santelli J, Desai S. Understanding the Decline in Adolescent Fertility in the United States, 2007-2012. J Adolesc Health. 2016;59(5):577-83. doi: 10.1016/j.jadohealth.2016.06.024. PubMed PMID: 27595471; PMCID: PMC5498007.
53. WHO. Family Planning Evidence Brief – Expanding contraceptive choice WHO/RHR/17.14 Rev.1. Available at: https://www.who.int/reproductivehealth/publications/family_planning/expanding-contraceptive-choice/en/. 2017.

54. Nel A, van Niekerk N, Kapiga S, Bekker LG, Gama C, Gill K, Kamali A, Kotze P, Louw C, Mabude Z, Miti N, Kusemererwa S, Tempelman H, Carstens H, Devlin B, Isaacs M, Malherbe M, Mans W, Nuttall J, Russell M, Ntshele S, Smit M, Solai L, Spence P, Steytler J, Windle K, Borremans M, Resseler S, Van Roey J, Parys W, Vangeneugden T, Van Baelen B, Rosenberg Z, Ring Study T. Safety and Efficacy of a Dapivirine Vaginal Ring for HIV Prevention in Women. The New England journal of medicine. 2016;375(22):2133-43. doi: 10.1056/NEJMoa1602046. PubMed PMID: 27959766.
55. Baeten JM, Palanee-Phillips T, Brown ER, Schwartz K, Soto-Torres LE, Govender V, Mgodi NM, Matovu Kiweewa F, Nair G, Mhlanga F, Siva S, Bekker LG, Jeenarain N, Gaffoor Z, Martinson F, Makanani B, Pather A, Naidoo L, Husnik M, Richardson BA, Parikh UM, Mellors JW, Marzinke MA, Hendrix CW, van der Straten A, Ramjee G, Chirenje ZM, Nakabiito C, Taha TE, Jones J, Mayo A, Scheckter R, Berthiaume J, Livant E, Jacobson C, Ndase P, White R, Patterson K, Germuga D, Galaska B, Bunge K, Singh D, Szydlo DW, Montgomery ET, Mensch BS, Torjesen K, Grossman CI, Chakhtoura N, Nel A, Rosenberg Z, McGowan I, Hillier S, Team M-AS. Use of a Vaginal Ring Containing Dapivirine for HIV-1 Prevention in Women. The New England journal of medicine. 2016;375(22):2121-32. doi: 10.1056/NEJMoa1506110. PubMed PMID: 26900902; PMCID: PMC4993693.
56. Tucker JD, Tso LS, Hall B, Ma Q, Beanland R, Best J, Li H, Lackey M, Marley G, Rich ZC, Sou KL, Doherty M. Enhancing Public Health HIV Interventions: A Qualitative Meta-Synthesis and Systematic Review of Studies to Improve Linkage to Care, Adherence, and Retention. EBioMedicine. 2017;17:163-71. doi: 10.1016/j.ebiom.2017.01.036. PubMed PMID: 28161401; PMCID: PMC5360566.
57. Rachlis B, Naanyu V, Wachira J, Genberg B, Koech B, Kamene R, Akinyi J, Braitstein P. Identifying common barriers and facilitators to linkage and retention in chronic disease care in western Kenya. BMC public health. 2016;16:741. doi: 10.1186/s12889-016-3462-6. PubMed PMID: 27503191; PMCID: PMC4977618.
58. Mukumbang FC, Mwale JC, van Wyk B. Conceptualising the Factors Affecting Retention in Care of Patients on Antiretroviral Treatment in Kabwe District, Zambia, Using the Ecological Framework. AIDS Res Treat. 2017;2017:7356362. doi: 10.1155/2017/7356362. PubMed PMID: 29250442; PMCID: PMC5700481.
59. Bajunirwe F, Tumwebaze F, Akakimpa D, Kityo C, Mugyenyi P, Abongomera G. Towards 90-90-90 Target: Factors Influencing Availability, Access, and Utilization of HIV Services-A Qualitative Study in 19 Ugandan Districts. Biomed Res Int. 2018;2018:9619684. doi: 10.1155/2018/9619684. PubMed PMID: 29750175; PMCID: PMC5884295.
60. Zanoni BC, Sibaya T, Cairns C, Haberer JE. Barriers to Retention in Care are Overcome by Adolescent-Friendly Services for Adolescents Living with HIV in South Africa: A Qualitative Analysis. AIDS Behav. 2019;23(4):957-65. doi: 10.1007/s10461-018-2352-6. PubMed PMID: 30535836; PMCID: PMC6459720.
61. Ayieko J, Brown L, Anthierens S, Van Rie A, Getahun M, Charlebois ED, Petersen ML, Clark TD, Kamya MR, Cohen CR, Bukusi EA, Havlir DV, Camlin CS. "Hurdles on the path to 90-90-90 and beyond": Qualitative analysis of barriers to engagement in HIV care among individuals in rural East Africa in the context of test-and-treat. PLoS ONE. 2018;13(8):e0202990. doi: 10.1371/journal.pone.0202990. PubMed PMID: 30161172; PMCID: PMC6116983 Gilead Sciences, during the conduct of the study; grants from National Institutes of Health, grants from Gates Foundation, outside the submitted work. EDC reports grants from National Institutes of Health, during the conduct of the study. We

would like to confirm that this does not alter our adherence to PLOS ONE's policies on sharing data and materials.
62. Brown LB, Getahun M, Ayieko J, Kwarisiima D, Owaraganise A, Atukunda M, Olilo W, Clark T, Bukusi EA, Cohen CR, Kamya MR, Petersen ML, Charlebois ED, Havlir DV, Camlin CS. Factors predictive of successful retention in care among HIV-infected men in a universal test-and-treat setting in Uganda and Kenya: A mixed methods analysis. PLoS ONE. 2019;14(1):e0210126. doi: 10.1371/journal.pone.0210126. PubMed PMID: 30673744; PMCID: PMC6343966 following competing interests: LBB, CRC, EDC, MLP, MRK, DVH, and CSC have received grants from the National Institutes of Health. DVH has received non-financial support (donation of study drug Truvada) from Gilead Sciences. All other authors declare that no competing interests exist.
63. Ataklte F, Erqou S, Kaptoge S, Taye B, Echouffo-Tcheugui JB, Kengne AP. Burden of undiagnosed hypertension in sub-saharan Africa: a systematic review and meta-analysis. Hypertension. 2015;65(2):291-8. doi: 10.1161/HYPERTENSIONAHA.114.04394. PubMed PMID: 25385758.
64. Chamie G, Kwarisiima D, Clark TD, Kabami J, Jain V, Geng E, Petersen ML, Thirumurthy H, Kamya MR, Havlir DV, Charlebois ED. Leveraging Rapid Community-Based HIV Testing Campaigns for Non-Communicable Diseases in Rural Uganda. PLoS ONE. 2012;7(8):e43400. Epub 2012/08/24. doi: 10.1371/journal.pone.0043400. PubMed PMID: 22916256; PMCID: 3423366.
65. Kwarisiima D, Balzer L, Heller D, Kotwani P, Chamie G, Clark T, Ayieko J, Mwangwa F, Jain V, Byonanebye D, Petersen M, Havlir D, Kamya MR. Population-Based Assessment of Hypertension Epidemiology and Risk Factors among HIV-Positive and General Populations in Rural Uganda. PLoS One. 2016;11(5):e0156309. doi: 10.1371/journal.pone.0156309. PubMed PMID: 27232186; PMCID: PMC4883789.
66. Price AJ, Crampin AC, Amberbir A, Kayuni-Chihana N, Musicha C, Tafatatha T, Branson K, Lawlor DA, Mwaiyeghele E, Nkhwazi L, Smeeth L, Pearce N, Munthali E, Mwagomba BM, Mwansambo C, Glynn JR, Jaffar S, Nyirenda M. Prevalence of obesity, hypertension, and diabetes, and cascade of care in sub-Saharan Africa: a cross-sectional, population-based study in rural and urban Malawi. Lancet Diabetes Endocrinol. 2018;6(3):208-22. doi: 10.1016/S2213-8587(17)30432-1. PubMed PMID: 29371076; PMCID: PMC5835666.
67. Global Status Report on Alcohol and Health. Geneva: World Health Organization. 2018.
68. Filmer D, Fox L. Youth Employment in Sub-Saharan Africa. Africa Development Series. Washington, DC: World Bank. doi:10.1596/978-1-4648-0107-5. License: Creative Commons Attribution CC BY 3.02014.
69. Glasgow RE, Vogt TM, Boles SM. Evaluating the public health impact of health promotion interventions: the RE-AIM framework. American journal of public health. 1999;89(9):1322-7. doi: 10.2105/ajph.89.9.1322.
70. Bradley KA, Bush KR, Epler AJ, Dobie DJ, Davis TM, Sporleder JL, Maynard C, Burman ML, Kivlahan DR. Two brief alcohol-screening tests From the Alcohol Use Disorders Identification Test (AUDIT): validation in a female Veterans Affairs patient population. Archives of internal medicine. 2003;163(7):821-9. Epub 2003/04/16. doi: 10.1001/archinte.163.7.821. PubMed PMID: 12695273.
71. Bradley KA, DeBenedetti AF, Volk RJ, Williams EC, Frank D, Kivlahan DR. AUDIT-C as a brief screen for alcohol misuse in primary care. Alcohol Clin Exp Res.

2007;31(7):1208-17. Epub 2007/04/25. doi: 10.1111/j.1530-0277.2007.00403.x. PubMed PMID: 17451397.
72. Kadede K, Ruel T, Kabami J, Ssemmondo E, Sang N, Kwarisiima D, Bukusi E, Cohen CR, Liegler T, Clark TD, Charlebois ED, Petersen ML, Kamya MR, Havlir DV, Chamie G, team S. Increased adolescent HIV testing with a hybrid mobile strategy in Uganda and Kenya. AIDS (London, England). 2016;30(14):2121-6. doi: 10.1097/QAD.0000000000001180. PubMed PMID: 27258399; PMCID: PMC5007167.
73. Rotheram-Borus MJ, Lightfoot M, Kasirye R, Desmond K. Vocational training with HIV prevention for Ugandan youth. AIDS Behav. 2012;16(5):1133-7. Epub 2011/07/30. doi: 10.1007/s10461-011-0007-y. PubMed PMID: 21800180; PMCID: PMC3947885.
74. Pronyk PM, Hargreaves JR, Kim JC, Morison LA, Phetla G, Watts C, Busza J, Porter JD. Effect of a structural intervention for the prevention of intimate-partner violence and HIV in rural South Africa: a cluster randomised trial. Lancet. 2006;368(9551):1973-83. Epub 2006/12/05. doi: 10.1016/S0140-6736(06)69744-4. PubMed PMID: 17141704.
75. Ssewamala FM, Ismayilova L, McKay M, Sperber E, Bannon W, Jr., Alicea S. Gender and the effects of an economic empowerment program on attitudes toward sexual risk-taking among AIDS-orphaned adolescent youth in Uganda. J Adolesc Health. 2010;46(4):372-8. Epub 2010/03/24. doi: 10.1016/j.jadohealth.2009.08.010. PubMed PMID: 20307827; PMCID: PMC2844862.
76. Erulkar A, Ferede A, Girma W, Ambelu W. Evaluation of “Biruh Tesfa” (Bright Future) program for vulnerable girls in Ethiopia. Vulnerable Children and Youth Studies. 2013;8(2):182-92.
77. Chamie G, Sang N, Kwarisiima D, Kabami J, Kadede K, Bagala I, Mucunguzi A, Clark TD, Balzer LB, Charlebois ED, Cohen CR, Bukusi EA, Petersen ML, Havlir D, Kamya M, editors. Yield of HIV Testing and Re-engagement of Key Populations in Uganda and Kenya. Abstract #896. Conference on Retroviruses and Opportunistic Infections (CROI); 2019; Seattle, Washington.
78. Mayer CM, Owaraganise A, Kabami J, Kwarisiima D, Koss CA, Charlebois ED, Kamya MR, Petersen ML, Havlir DV, Jewell BL. Distance to clinic is a barrier to PrEP uptake and visit attendance in a community in rural Uganda. J Int AIDS Soc. 2019;22(4):e25276. Epub 2019/05/01. doi: 10.1002/jia2.25276. PubMed PMID: 31037845; PMCID: PMC6488759.
79. Jewell B, Ayieko J, Owaraganise A, Mwangwa F, Kwarisiima D, Koss CA, Plenty A, Black D, Bukusi E, Cohen CR, Clark T, Charlebois E, Kamya M, Petersen M, Havlir D, editors. Community delivery increases PrEP program retention in SEARCH study in Uganda and Kenya. Abstract WEPEC247. 22nd International AIDS Conference. 23-27 July. Amsterdam, The Netherlands.2018.
80. Olilo WA, Petersen ML, Koss CA, Wafula E, Kwarisiima D, Kadede K, Clark TD, Cohen CR, Bukusi EA, Kamya MR, Charlebois ED, Havlir DV, Ayieko J, Collaboration S. Pre-exposure Prophylaxis (PrEP) Uptake Among Older Individuals in Rural Western Kenya. Journal of acquired immune deficiency syndromes (1999). 2019;82(4):e50-e3. Epub 2019/09/07. doi: 10.1097/QAI.0000000000002150. PubMed PMID: 31490343; PMCID: PMC6831040.
81. Camlin CS, Koss CA, Getahun M, Owino L, Itiakorit H, Akatukwasa C, Maeri I, Bakanoma R, Onyango A, Atwine F, Ayieko J, Kabami J, Mwangwa F, Atukunda M, Owaraganise A, Kwarisiima D, Sang N, Bukusi EA, Kamya M, Petersen ML, Cohen CR, Charlebois ED, Havlir D. Understanding demand for PrEP and early experiences of PrEP

use among young adults in rural Kenya and Uganda: A qualitative study. AIDS Behav. 2020;(in press).
82. Koss C, Ayieko J, Owaraganise A, Mwangwa F, Wallenta J, Kwarisiima D, Ruel T, Black D, Cohen CR, Bukusi E, Clark T, Charlebois E, Petersen M, Kamya M, Camlin C, Havlir D. Survey of early barriers to PrEP uptake among clients and community members in the SEARCH study in rural Kenya and Uganda. Abstract WEPEC0913. International AIDS Society Conference; Paris, France2017.
83. Camlin CS, Charlebois ED, Geng E, Semitala F, Wallenta J, Getahun M, Kampiire L, Bukusi EA, Sang N, Kwarisiima D, Clark TD, Petersen ML, Kamya MR, Havlir DV. Redemption of the "spoiled identity:" the role of HIV-positive individuals in HIV care cascade interventions. J Int AIDS Soc. 2017;20(4). doi: 10.1002/jia2.25023. PubMed PMID: 29210185; PMCID: PMC5810337.
84. Brown LB, Balzer LB, Kabami J, Kwarisiima D, Sang N, Ayieko J, Chen Y, Chamie G, Charlebois ED, Camlin CS, Cohen CR, Bukusi E, Kamya MR, Moody J, Havlir DV, Petersen ML. The Influence of Social Networks on Antiretroviral Therapy Initiation Among HIV-Infected Antiretroviral Therapy-Naive Youth in Rural Kenya and Uganda. Journal of acquired immune deficiency syndromes (1999). 2020;83(1):9-15. Epub 2019/12/07. doi: 10.1097/QAI.0000000000002199. PubMed PMID: 31809357.
85. Knight J, Wachira J, Kafu C, Braitstein P, Wilson IB, Harrison A, Owino R, Akinyi J, Koech B, Genberg B. The Role of Gender in Patient-Provider Relationships: A Qualitative Analysis of HIV Care Providers in Western Kenya with Implications for Retention in Care. AIDS Behav. 2019;23(2):395-405. doi: 10.1007/s10461-018-2265-4. PubMed PMID: 30168005; PMCID: PMC6368874.
86. Mwamba C, Sharma A, Mukamba N, Beres L, Geng E, Holmes CB, Sikazwe I, Topp SM. 'They care rudely!': resourcing and relational health system factors that influence retention in care for people living with HIV in Zambia. BMJ Glob Health. 2018;3(5):e001007. doi: 10.1136/bmjgh-2018-001007. PubMed PMID: 30483408; PMCID: PMC6231098.
87. Wachira J, Naanyu V, Genberg B, Koech B, Akinyi J, Kamene R, Ndege S, Siika AM, Kimayo S, Braitstein P. Health facility barriers to HIV linkage and retention in Western Kenya. BMC Health Serv Res. 2014;14:646. doi: 10.1186/s12913-014-0646-6. PubMed PMID: 25523349; PMCID: PMC4276260.
88. Mburu G, Ram M, Siu G, Bitira D, Skovdal M, Holland P. Intersectionality of HIV stigma and masculinity in eastern Uganda: implications for involving men in HIV programmes. BMC public health. 2014;14:1061. doi: 10.1186/1471-2458-14-1061. PubMed PMID: 25304035; PMCID: PMC4200195.
89. Ndyabakira A, Chamie G, Emperador D, Marson K, Kamya MR, Havlir DV, Kwarisiima D, Thirumurthy H. Men's Beliefs About the Likelihood of Serodiscordance in Couples with an HIV-Positive Partner: Survey Evidence from Rural Uganda. AIDS Behav. 2019. Epub 2019/05/11. doi: 10.1007/s10461-019-02531-7. PubMed PMID: 31073946.
90. Camlin CS, Akullian A, Neilands TB, Getahun M, Bershteyn A, Ssali S, Geng E, Gandhi M, Cohen CR, Maeri I, Eyul P, Petersen ML, Havlir DV, Kamya MR, Bukusi EA, Charlebois ED. Gendered dimensions of population mobility associated with HIV across three epidemics in rural Eastern Africa. Health Place. 2019;57:339-51. Epub 2019/06/04. doi: 10.1016/j.healthplace.2019.05.002. PubMed PMID: 31152972; PMCID: PMC6589115.
91. Jonas DE, Garbutt JC, Amick HR, Brown JM, Brownley KA, Council CL, Viera AJ, Wilkins TM, Schwartz CJ, Richmond EM, Yeatts J, Evans TS, Wood SD, Harris RP. Behavioral counseling after screening for alcohol misuse in primary care: a systematic

review and meta-analysis for the U.S. Preventive Services Task Force. Annals of internal medicine. 2012;157(9):645-54. Epub 2012/09/26. doi: 10.7326/0003-4819-157-9-201211060-00544. PubMed PMID: 23007881.
92. Scott-Sheldon LAJ, Carey KB, Johnson BT, Carey MP, Team MR. Behavioral Interventions Targeting Alcohol Use Among People Living with HIV/AIDS: A Systematic Review and Meta-Analysis. AIDS Behav. 2017;21(Suppl 2):126-43. Epub 2017/08/24. doi: 10.1007/s10461-017-1886-3. PubMed PMID: 28831609; PMCID: PMC5660648.
93. Chander G, Hutton HE, Lau B, Xu X, McCaul ME. Brief Intervention Decreases Drinking Frequency in HIV-Infected, Heavy Drinking Women: Results of a Randomized Controlled Trial. Journal of acquired immune deficiency syndromes (1999). 2015;70(2):137-45. doi: 10.1097/QAI.0000000000000679. PubMed PMID: 25967270; PMCID: PMC4634011.
94. Nadkarni A, Weobong B, Weiss HA, McCambridge J, Bhat B, Katti B, Murthy P, King M, McDaid D, Park AL, Wilson GT, Kirkwood B, Fairburn CG, Velleman R, Patel V. Counselling for Alcohol Problems (CAP), a lay counsellor-delivered brief psychological treatment for harmful drinking in men, in primary care in India: a randomised controlled trial. Lancet. 2017;389(10065):186-95. Epub 2016/12/19. doi: 10.1016/S0140-6736(16)31590-2. PubMed PMID: 27988144; PMCID: PMC5236065.
95. Nadkarni A, Weiss HA, Weobong B, McDaid D, Singla DR, Park AL, Bhat B, Katti B, McCambridge J, Murthy P, King M, Wilson GT, Kirkwood B, Fairburn CG, Velleman R, Patel V. Sustained effectiveness and cost-effectiveness of Counselling for Alcohol Problems, a brief psychological treatment for harmful drinking in men, delivered by lay counsellors in primary care: 12-month follow-up of a randomised controlled trial. PLoS medicine. 2017;14(9):e1002386. Epub 2017/09/13. doi: 10.1371/journal.pmed.1002386. PubMed PMID: 28898239; PMCID: PMC5595289.
96. Chingono A, Lane T, Chitumba A, Kulich M, Morin S. Balancing science and community concerns in resource-limited settings: Project Accept in rural Zimbabwe. Clin Trials. 2008;5(3):273-6. doi: 10.1177/1740774508091576. PubMed PMID: 18559417.
97. Ministry of Health, National AIDS & STI Control Program. Guidelines on Use of Antiretroviral Drugs for Treating and Preventing HIV Infection in Kenya 2018 Edition. Nairobi, Kenya: NASCOP, August. 2018.
98. Consolidated Guidelines for the Prevention and Treatment of HIV and AIDS in Uganda. Second Edition - September 2018. Uganda Ministry of Health. 2018.
99. Fleming MF, Barry KL, Manwell LB, Johnson K, London R. Brief physician advice for problem alcohol drinkers. A randomized controlled trial in community-based primary care practices. JAMA. 1997;277(13):1039-45. PubMed PMID: 9091691.
100. Ssemmondo E, Mwangwa F, Kironde JL, Kwarisiima D, Clark TD, Marquez C, Charlebois ED, Petersen ML, Kamya MR, Havlir DV, Chamie G, Collaboration S. Population-based active TB case finding during large-scale mobile HIV testing campaigns in rural Uganda. Journal of acquired immune deficiency syndromes (1999). 2016. doi: 10.1097/QAI.0000000000001142. PubMed PMID: 27509252.
101. Gandhi M, Murnane PM, Bacchetti P, Elion R, Kolber MA, Cohen SE, Horng H, Louie A, Kuncze K, Koss CA, Anderson PL, Buchbinder S, Liu A. Hair levels of preexposure prophylaxis drugs measure adherence and are associated with renal decline among men/transwomen. AIDS (London, England). 2017;31(16):2245-51. doi: 10.1097/QAD.0000000000001615. PubMed PMID: 28832411; PMCID: PMC5633521.
102. Hahn JA, Anton RF, Javors MA. The Formation, Elimination, Interpretation, and Future Research Needs of Phosphatidylethanol for Research Studies and Clinical

Practice. Alcohol Clin Exp Res. 2016;40(11):2292-5. doi: 10.1111/acer.13213. PubMed PMID: 27716960; PMCID: PMC5117827.
103. Havlir D, Balzer L, Charlebois E, Clark T, Kwarisiima D, Ayieko J, Kabami J, Sang N, Liegler T, Chamie G, Camlin CS, Jain V, Kadede K, Atukunda M, Ruel T, Shade S, Ssemmondo E, Byonanebye D, Mwangwa F, Owaraganise A, Olilo W, Black D, Snyman K, Burger R, Getahun M, Achando J, Awuonda B, Nakato H, Kironde JL, Schwab J, Lavoy G, Plenty A, Wafula EM, Omanya P, Rooney J, Bacon MC, van der Laan M, Cohen C, Bukusi E, Kamya M, Petersen M. Trial of HIV Test and Treat via a Community Health Model in Rural Africa. New England Journal of Medicine. 2019(*In press.*).
104. van der Laan M, Rose S. Targeted Learning: Causal Inference for Observational and Experimental Data. New York Dordrecht Heidelberg London: Springer; 2011.
105. R Core Team. R: A Language and Environment for Statistical Computing. R Foundation for Statistical Computing; 2019. http://www.R-project.org2019.
106. Balzer LB, Havlir D, Schwab J, van der Laan M, Petersen M. Statistical Analysis Plan for SEARCH Phase I: Health Outcomes among Adults. Accessible at: arXiv; https://arxiv.org/abs/1808.032312018.
107. Hayes RJ, Moulton LH. Cluster Randomized Trials. Boca Raton: Chapman and Hall; 2009.
108. Balzer LB, van der Laan MJ, Petersen ML, Collaboration S. Adaptive pre-specification in randomized trials with and without pair-matching. Stat Med. 2016;35(25):4528-45. doi: 10.1002/sim.7023. PubMed PMID: 27436797; PMCID: PMC5084457.
109. Petersen ML, Sinisi SE, van der Laan MJ. Estimation of direct causal effects. Epidemiology. 2006;17(3):276-84. Epub 2006/04/18. doi: 10.1097/01.ede.0000208475.99429.2d. PubMed PMID: 16617276.
110. Didelez V, Dawid AP, Geneletti S, editors. Direct and Indirect Effects of Sequential Treatments. Proceedings of the 22nd Annual Conference on Uncertainty in Artificial Intelligence; 2006.
111. Abadie A. Semiparametric Difference-in-Differences Estimators. The Review of Economic Studies. 2005;72(1):1-19.
112. Kassanjee R, McWalter TA, Barnighausen T, Welte A. A new general biomarker-based incidence estimator. Epidemiology. 2012;23(5):721-8. Epub 2012/05/26. doi: 10.1097/EDE.0b013e3182576c07. PubMed PMID: 22627902; PMCID: PMC3500970.

18. APPENDIX Extension of Dynamic Prevention Studies and Procedures for measuring implementation on Cabotegravir Injectable Suspension (CAB-LA)

National Clinical Trial (NCT) Identified Number: NCT05549726

This Appendix describes the continuation of the 3 Dynamic Prevention Randomized studies (refer to section 9.2.1 of protocol) for a 48 week follow up period following re-consent for this CAB-LA extension. Persons who are already enrolled in the trials and were randomized to the intervention arm will be eligible to receive CAB-LA as one of the options in the "Dynamic prevention" model. Persons in the control arms will be eligible to receive CAB-LA if and when it is rolled out through in country programs. If CAB-LA is not available in country at the 48-week endpoint, all participants in the intervention arm will have the opportunity to continue in the study until Week 96, and participants who are currently on CAB-LA may continue through the extension. The study will end at Week 96, and by that time participants will be transitioned to in country prevention care. At 96 weeks, persons on CAB-LA will be transitioned to in country CAB-LA or to other prevention options if CAB-LA is not available. Prior to this transition, available in country options will be assessed by the study team. HIV prevention options will be discussed with each study participant by their Week 88 visit, with a plan established for the transition to the participant's preferred prevention method in coordination with local health facilities to avoid interruption after their 96 weeks of study participation is completed.

The overall purpose of this extension is to 1) Determine if adding the option of CAB-LA as a prevention choice using a patient-centered HIV prevention delivery model increases prevention coverage (i.e. proportion of time that is covered by a biomedical prevention option, such as PrEP or PEP) compared to the standard-of-care in 3 ongoing randomized trials of Dynamic Choice Prevention in rural Uganda and Kenya; and, 2) Conduct a hybrid implementation study (Primary Objective Clinical and Secondary Objective Implementation) focusing on initial implementation of a patient-centered model for CAB-LA using the RE-AIM evaluation framework among persons randomized to the intervention arms.

A. Background and Rationale

Background: SEARCH Sapphire is an ongoing, NIH-funded study that is testing multi-disease and multi-sector interventions aimed at reducing HIV burden and improving health in rural Uganda and Kenya. Phase A is a portfolio of randomized prevention and treatment implementation studies that will inform a population-level study in Phase B (refer to section 3.0 of protocol).

This appendix describes a 48-week extension of 3 ongoing randomized trials in Phase A studying a patient-centered "Dynamic Choice Prevention" (DCP) implementation strategy for delivering existing evidence-based biomedical prevention interventions vs. standard of care (SOC). The Dynamic prevention model offers participants choices on prevention modality on an ongoing basis: oral PrEP, or oral PEP, and the option to switch between products. The trials are being conducted in 3 settings: antenatal (ANC) clinics, the outpatient department (primary care clinics), and in rural community settings in Western Kenya and Uganda (refer to section 9.2.1 of protocol). All clinic and village health team staff (i.e., Clinical Officers, nurses, coordinators, health workers) are trained and equipped for HIV prevention care in the clinical setting, appropriate to their role (refer to section 9.2.1.1 of the protocol).

In the CAB-LA extension, participants in the intervention and control arms are reconsented and remain in their initial randomized arm. Persons in the intervention arms who remain HIV

negative will be offered CAB-LA as an additional biomedical prevention option, if eligible. CAB-LA will be delivered at health clinics. The primary 48-week endpoint for this extension is proportion of time covered by a biomedical prevention product. As of May 9, 2022, a total of 1213 HIV-negative persons aged 15 years and older and at risk for HIV acquisition were enrolled in the studies. (Table 1). New participants are being enrolled at the present to time to replace those participants who have moved and have been unable to continue in the CAB-LA study. The total number of participants in the study eligible for CAB-LA will not exceed the 593 listed in Table 1.

Table 1. Enrollment in 3 SEARCH Sapphire Dynamic Prevention Trials as of May 9, 2022

Dynamic Prevention Study	Intervention and Control, (N) Uganda	Intervention (N) Uganda	Intervention and Control, (N) Kenya	Intervention (N) Kenya	Total Enrolled (N), Intervention and Control	Total Enrolled Intervention (N)
Antenatal/SRH Clinic	200	100	201	103	401	203
Outpatient Department Clinics	201	100	201	97	402	197
Household Study Community Village Health Team (VHT)	200	83	210	110	410	193
All Trials Together	601	283	612	310	1213	593

<u>Population:</u> The persons eligible for participation in the extension are those who were enrolled in the 3 ongoing trials. Persons for the ANC study are recruited and enrolled through offering study participation at ANC clinics at government sponsored health facilities. Persons for the Outpatient department are recruited and enrolled through offering study participation at Outpatient department clinics at government sponsored health facilities. Persons for the community study are recruited via home visits by village health teams/community health workers (refer to section 7.5.1 of protocol).

Inclusion criteria for the Extension include:

1) Enrollment in a SEARCH Sapphire Dynamic prevention study
2) HIV negative at start of extension
3) Residing in study region

Additional inclusion criteria to access CAB-LA as a prevention option

1) Not pregnant at time of initial CAB-LA injection
2) Participant weighs at least 35kg

Additional Exclusion Criteria to access CAB-LA as a prevention option

1) Participant has Hepatitis B or chronic Hepatitis C Diagnosis
2) Participant has ALT >=5x ULN
3) Participant has clinical history of liver cirrhosis or current clinical evidence of cirrhosis or severe liver disease
4) Previous hypersensitivity reaction to cabotegravir

5) Receiving the following co-administered drugs for which significant decreases in cabotegravir plasma concentrations may occur due to uridine diphosphate glucuronosyltransferase:
   i. Anticonvulsants: Carbamazepine, oxcarbazepine, phenobarbital, phenytoin
   ii. Antimycobacterials: Rifampin, rifapentine
6) Participants with a current or anticipated need for chronic systemic anticoagulation or a history of known or suspected bleeding disorder, including a history of prolonged bleeding, except for the use of anticoagulation for deep vein thrombosis (DVT) prophylaxis (e.g., postoperative DVT prophylaxis) or the use of low dose acetylsalicylic acid (≤325 mg).

Study Intervention Clinic Locations: All participating ANC and OPD clinics in the DCP trial will offer CAB-LA. For VHT/CHV communities, the nearest health facility will offer CAB-LA for all participants
1) In Uganda, the sites for each of the trials:
   a. OPD: Itojo Hospital, Kitagata Hospital
   b. ANC: Bwizibwera HCIV, Bushenyi HCIV
   c. VHT: Ndeija Health Center III
2) In Kenya, the sites for each of the trials:
   a. OPD: Magunga Level IV Hospital, Oyani Hospital
   b. ANC: Oyani Hospital, Sena Health Centre
   c. CHV: Sibouche Health Centre, Ogongo Sub-District Hospital

The demographic characteristics of the persons enrolled in the intervention arm as of May 9, 2022 currently who will be eligible for CAB-LA patient-centered intervention are shown in Table 2.

Table 2. Demographics of persons in intervention arm eligible for CAB-LA in extension phase of trial as of May 9, 2022.

	**ANC Clinic**	**Outpatient Department**	**Community**
Total Enrolled	203	197	193
Female Participants	203 (100%)	119 (60.4%)	111 (57.2%)
Age years number and %			
15-25 years	120 (56.9%)	90 (44.1%)	75 (38.9%)
26- 30	47 (22.3%)	33 (25.0%)	29 (25.4%)
31-40	35 (16.6%)	51 (16.2%)	49 (18.7%)
41-60	9 (4.3%)	26 (12.7%)	36 (15.0%)
61+	0 (0%)	4 (2.0%)	4 (2.1%)

Rationale: HIV prevention services that offer choice of prevention products (PrEP & PEP) and flexibility in their use and location of delivery over time allow for a dynamic, patient-centered model of prevention. This approach is responsive to the reality of an individual's changing needs, thereby facilitating continued engagement. CAB-LA is highly efficacious for HIV prevention that was recently approved by the US FDA based on two large, randomized trials HPTN 083 and HPTN 084. As CAB-LA is rolled out across rural sub-Saharan Africa, it is

important to determine **how the addition of this option impacts prevention coverage in real-world settings** for <u>women and men</u> at risk for HIV. In addition, it is critical to design, evaluate, and improve **<u>patient-centered CAB-LA delivery</u>** strategies that maximize uptake and impact of this new product.

<u>Ethical Considerations:</u> The HPTN 084 study that showed efficacy of CAB LA for prevention included sites in both Uganda and Kenya. Applications for approval of CAB LA are under review in each of these countries. Thus, the information generated in this study on evaluating a dynamic prevention implementation strategy will be directly applicable. *During this proposed study extension, CAB LA is expected to become publicly available through Ministry of Health ("MoH") sponsored programs (in both Kenya and Uganda). UCSF is developing an additional extension to 96 weeks, which will examine longer-term safety and implementation data of CAB-LA. Should there be delay in rollout of the MoH programs, participants could continue in this additional extension phase. After the endpoint of the 96-week extension is reached, participants will be transitioned to a standard of care regimen, via local healthcare system, based on the investigator's clinical judgment and local country guidelines*

B. Hypothesis and Objectives

<u>Hypotheses:</u> A dynamic choice prevention model that includes a patient-entered CAB-LA delivery option will have higher levels of biomedical prevention coverage (assessed over 48 weeks following re-consent for the CAB-LA extension study) compared to the standard of care.

This hypothesis will be tested by comparing covered time between the intervention and control arms in the 3 ongoing randomized trials during the extension phase.

<u>The secondary hypothesis</u>: Addition of patient-centered CAB-LA into an ongoing dynamic choice prevention will improve biomedical prevention coverage (compared to dynamic choice prevention without CAB-LA).

These hypotheses will be tested by comparing covered time during the extension with the CAB-LA option to covered time prior to the extension among intervention arm participants.

We expect that incorporating the option of CAB-LA with patient-centered delivery for persons who self-identify at risk for HIV living in rural Uganda and Kenya will increase overall biomedical covered prevention time compared to the standard of care. In a population that has been offered -- and some of whom have taken up -- oral PrEP and PEP, we also expect to learn characteristics of who is most interested in CAB-LA, how our patient-centered CAB-LA delivery models work in different settings, and the cost of the delivery models in different settings.

<u>Primary Objective:</u> To compare biomedical prevention coverage achieved using a Dynamic prevention model that includes a patient-centered CAB-LA delivery intervention to biomedical prevention coverage under the standard of care over 48 weeks.

<u>Secondary Objectives:</u> To determine the reach, effectiveness, adoption, implementation and maintenance of a patient-centered CAB-LA program embedded in 3 ongoing trials in the setting of antenatal clinic, outpatient clinic, and community.

<u>Tertiary Objectives:</u> To evaluate change in knowledge, awareness and acceptability/satisfaction at the staff and provider level with CAB-LA before and after provider and staff training and education in CAB-LA with patient-centered delivery model.

C. Study Design and Schema

Study Design: This proposal is an **extension of 3 ongoing randomized trials comparing a patient-centered "Dynamic Choice" implementation strategy for delivering biomedical prevention options compared to standard of care** (Figure 1). Each trial is enrolling persons 15 years of age and older who are HIV uninfected and at risk of acquiring HIV. The Dynamic Choice Prevention intervention allows choice of PEP/PrEP, refill schedule, service delivery location (e.g. clinic, community, home), and HIV testing (self or staff). Persons can move from PEP to PrEP as they prefer (refer to section 9.2.1.4 of protocol).

Figure 1. Dynamic Choice Implementation Strategy

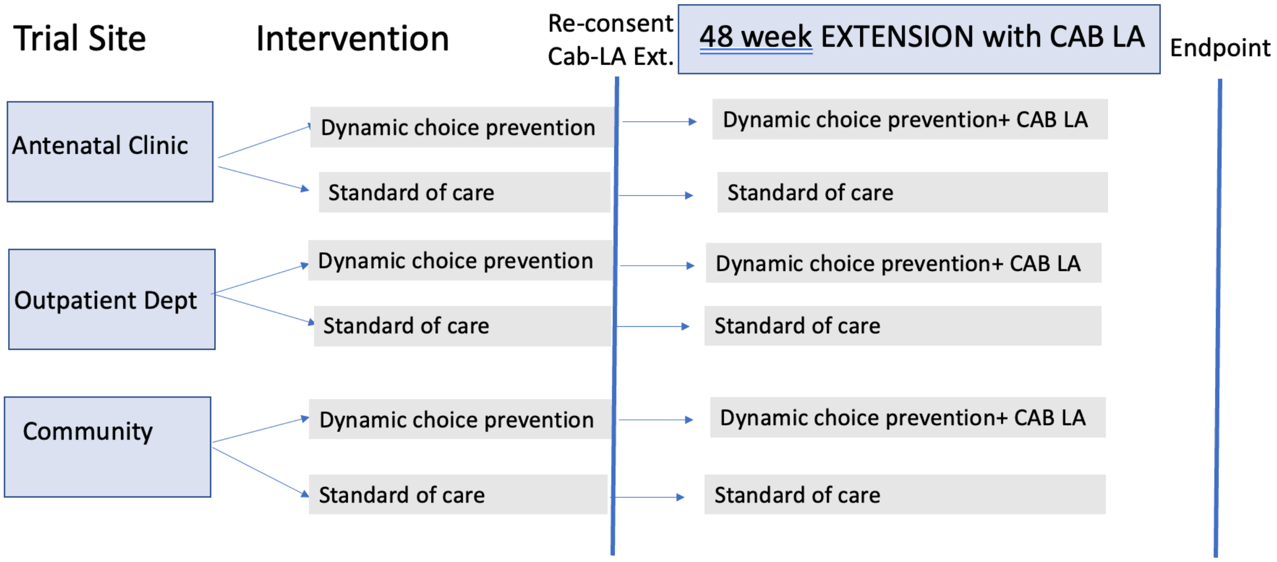

Intervention: In the extension phase, participants will remain in their initial randomization arm. For persons in the intervention arm, CAB-LA will be integrated into the dynamic choice delivery model as an additional biomedical prevention option in a patient-centered delivery model based on the precede framework.

The Dynamic Choice Delivery Model includes integrated PrEP and PEP services at outpatient clinics, antenatal clinics, and via VHT workers in community households. The procedures for each trial include PrEP/PEP counseling and services, choice of service location, HIV testing options, option for longer PrEP refills, provision of a clinical officer's or nurse's mobile telephone number for immediate PEP starts any day of the week, assessment of PrEP/PEP barriers and personalized actions, psychologic supports for traumatic experiences, and offer of concurrent, additional health or prevention related services (Table 10 of Main Protocol). For participants that chose CAB-LA as the biomedical prevention option, the patient-centered delivery model (Table 3) will be implemented. The standard of care differs according to country but does not routinely offer PEP or PrEP to clients seeking services, does not offer choice of service location, HIV testing option or access to medical provider mobile phone number. Clients are required to return

after one month for a refill for PREP, whereas in our model, there is an option from the start for 3 month medication supply.

Table 3. Patient-Centered Delivery Model

Intervention	Population and frequency	Purpose
**Education, case studies and discussion** on concept of dynamic prevention and on the profile of each prevention option product	Health center leadership and staff, provider, participants; initial and ongoing	Predisposing
**Integration** into antenatal, OPD clinics, **IPV/GBV support services, travel packs, 24 hour hotline for participants**<br>For oral PrEP/PEP only- HIV testing choice (self or staff) and home or clinic delivery	Participants; ongoing	Enabling
**Provider text/check-in to participant** one week after starting new option, **data on biomedical prevention covered time** (reinforcing)	Participants and staff; ongoing data feedback	Reinforcing

Outcomes: The primary outcome for the extension is the proportion of time covered by a biomedical prevention option during the 48 week follow-up period of the CAB-LA Extension; follow-up time will be censored at HIV diagnosis, death, or withdrawal. Biomedical covered time is defined as the proportion of follow-up time that each participant is covered by either PrEP (TDF/3TC or CAB-LA) or PEP. For TDF/3TC, covered time is defined using self-reported use; in a subset of participants who report PrEP use we will measure tenofovir drug levels using small hair samples to allow for calibration of self-reported measures. For CAB-LA, coverage begins 3 days after initial injection and continues 2 months + 7 days after last injectionFor PEP, covered time is defined using self-reported use; in a subset of participants who report PEP use we will measure drug levels using hair samples to allow for calibration of self-reported measures. A secondary outcome for the extension is “at-risk covered time”, defined as the proportion of time that a participant reports being at risk of HIV infection during which they are covered by a biomedical prevention option. Incident HIV infections, defined by a prespecified testing algorithm, will be an additional secondary outcome.

Secondary outcomes of reach, effectiveness, adoption, implementation and maintenance will be evaluated using a mixed methods approach that includes surveys of participants, and health center providers and staff. Tertiary outcomes of knowledge, awareness and acceptability/satisfaction at the staff and provider level will be measured by surveys at the baseline, midpoint and end of the study. Measurement details delineated in Table 4 .

In addition to these surveys, trained staff will conduct in depth interviews (IDI) to provide insights into the intervention implementation and attitudes of participants and staff. These qualitative in-depth interviews will be conducted among purposively-selected samples of study participants and providers, to ascertain the perceptions of and experiences with CAB-LA use and delivery. Participants who are clients will be asked about 1) Experiences with counseling, method/service preferences and decision-making to go on CAB-LA; 2) Method/services satisfaction, barriers and facilitators to uptake and maintenance, and social networks and support as a facilitator or hindrance; and 3) Recommendations to improve CAB-LA Delivery. Health care providers engaged in CAB-LA service delivery will be asked about their 1) Professional identity and motivation, as a potential factor influencing implementation; 2) First impressions and knowledge

of CAB-LA; 3) Clinical training experiences relevant to CAB-LA provision; 4) Experiences with and acceptability of individual elements of CAB-LA; and 5) Recommendations for improving CAB-LA delivery.

For all research activities related to qualitative evaluation of SEARCH SAPPHIRE, written informed consent will be obtained from all selected for recruitment to the study.

Qualitative IDIs for CAB-LA will be conducted between 2-6 months from enrolment. The specific data collection activities to be conducted during the phase are listed below. For Seroconverts, IDIs are will continue until the maximum number of participants is reached or the study intervention ends, whichever comes first.

i. CAB-LA Patient IDI cohort: We will systematically select ~n=30 patients per each region (South West Uganda and Kenya) (total 40 < ~n < 72), with samples within region to be balanced by original trials (Out- patient, ANC/PNC, VHT); further balance to be observed by gender and community, proportional to overall intervention enrolment.
ii. CAB-LA Provider IDI cohort: We will select a sample of care providers who administer CAB-LA from the CAB-LA trial health facilities or who inform the community about that option (VHTs / CHVs per each region. I.e. n=2-3 OPD providers, n=2-3 ANC/PNC providers, n-1-2 VHTs per region (total 8 < ~n < 12 providers per region).
iii. Seroconverts Patient IDI cohort: We aim to interview all participants in the intervention arm who received a diagnosis of HIV during the course of the DCP study and who are willing to be interviewed, up to a total of 36 per country.

For this study, IDIs will be recorded using the TASCAM DR-05 digital recorder. This digital recording device was selected for use in this study because it has a high quality microphone that captures high quality sound even in settings with a lot of ambient background noise.

For this study, the interviews will be conducted in the participant's preferred language. The Qualitative study team will then transcribe and translate the audio recorded data into English for analysis within 14 days of the interview. Staff will use either Express Scribe or VideoLAN Client (VLC) software for this process. QRCs/QRAs will share responsibility for coding all transcriptions of recorded IDIs. Coding will be conducted in Dedoose using a codebook developed iteratively by the team. Analysis will follow a modified framework analysis of key code excerpts exported from Dedoose after coding.

Costing of CAB-LA implementation will be conducted in each region (Kenya and Uganda) and location type (Antenatal Clinic, Outpatient Department, Community) at Baseline/Screening and at two injection visits during follow-up. Costing exercises will employ an activity-based approach and include site visits and employ rigorous costing methods using standardized micro-costing tools. We will also conduct time-and-motion activities using self-administered tools with on-site staff to estimate the amount of time spent implementing CAB-LA. We will interview study staff to identify resources used to implement the intervention and estimate time spent providing oversight and management of implementation activities.

Implementation Research Design: The secondary objective of the extension is an implementation evaluation of the intervention focused on the initial implementation of patient-centered CAB-LA using the RE-AIM framework. The domains of interest and key outcomes are summarized in the table below. Throughout, study weeks are used to refer to weeks in CAB-LA

extension (eg, “week 0” is date of re-consent for the CAB-LA extension, “week 48” is 48 weeks after re-consent for the CAB-LA extension).

Table 4. RE-AIM Evaluation Framework.

Domain /Outcome	Definition	Measurement	Timing
**Reach** (Participant level)	Proportion of eligible participants who initiate CAB-LA weeks 0-48. Characteristics (including prior PrEP and PEP use) and attitudes (knowledge, acceptability and feasibility) of participants using CAB-LA	Demographics, CAB-LA dispensing logs Knowledge, Acceptability and Feasibility (Survey and IDI). Evaluation includes focus on equitable access by gender, education and SES.	CAB LA dispensing throughout follow-up Surveys and IDI: Week 0, 24 and week 48
**Effectiveness** (Participant level)	Impact of adding CAB-LA to Dynamic choice prevention on biomedical covered time (comparison of follow-up during the CAB-LA extension vs. follow-up time in Phase A trials prior to CAB-LA extension).	CAB-LA dispensing logs, PrEP and PEP use as defined for primary endpoint in extension.	Throughout follow-up
**Adoption** (Provider and staff level in different settings)	% of staff and providers invited that participate in the interventions, where participation is defined as i) participation in CAB-LA trainings; and ii) offering CAB-LA to participants. We will further assess the characteristics of participant vs. non-participant staff.	Training attendance rosters; CAB-LA dispensing logs	Throughout follow-up
**Implementation** (Provider and systems level)	Fidelity: Proportion of providers offering CAB-LA on appropriate schedule, appropriate provision of oral bridging<br>Costing: Incremental cost of adding CAB-LA to dynamic choice prevention<br>Adaptations: Type, timing and reasons they were made	CAB-LA and oral bridging dispensing logs Time in motion and program costing Adaptation logs	Throughout follow-up
**Maintenance** (adapted from standard RE-AIM to accommodate shorter follow-up) (Participant level)	Persistence on CAB-LA among participants who initiate CAB-LA Reasons for discontinuation of biomedical prevention or switching to other biomedical prevention option	CAB-LA dispensing logs Participant survey on reasons for discontinuation and switching	Throughout follow up

As a tertiary objective, we will also assess provider and staff Knowledge, Acceptability and Awareness via provider and staff surveys (Likert scales) and IDI at weeks 0, 24, and 48.

D. Schedule of Evaluations

For all participants, from the time of reconsenting at the start of study CAB-LA availability (Week 0), there will be study visits every 24 weeks for the duration of their participation.

At the time of writing of this protocol version, the standard of care for both Kenya and Uganda is to offer biomedical prevention as part of the HIV prevention package that includes provision of PrEP for persons at risk of HIV acquisition, such as persons with partners living with HIV or of unknown HIV status, or those with a history of exchange/transactional sex, recent STIs, or recurrent use of PEP (more than 3 times a year). PEP is offered primarily after occupational exposures or in cases of sexual assault. For participants consenting in the extension, in the control arm, they will continue to be seen by government staff in the HIV clinic or other service location, like a drop-in centre, and receive prevention interventions according to standard of care guidelines recommended by the country's Ministry of Health. There will be no change to their HIV prevention healthcare (refer to section 9.2.1.5 of protocol)

Control participants will be seen for a study visit every 24 weeks for HIV testing (RNA and rapid), weight, and a survey on which prevention method(s) they have been using and self-reported risk. If control participants report using medications such as PrEP or PEP at the interim visits, they will be asked to provide hair, blood, or urine samples in order to measure HIV prevention medication levels.

For intervention participants who do not choose CAB-LA, there will be study visits every 12 weeks (refer to section 9.2.1 of the protocol); however, they can decide to begin CAB-LA at any time during the initial 48-week extension. Participants will not be able to initiate or restart CAB-LA in the additional extension period between Week 48 and Week 96; only those who are taking CAB-LA at the time of the Week 48 visit may continue. Intervention participants that choose CAB-LA will have study visits that align with their injection visits (every 4 weeks for the first two months and then every 8 weeks thereafter), but can return to an alternate DCP intervention if they decide to stop CAB-LA.

For participants that select CAB-LA, the extension visits will also include (Table 6):

- A separate qualitative interview (At time of CAB-LA start, Week 24 and Week 48)
- Safety Labs:
  - HBsAg at baseline
  - ALT at Weeks 0 and 24; optionally at Weeks 8 and 48
- Pregnancy tests
- In addition to the Week 24 and 48 extension visits, injection visits will occur in clinic every 4 weeks for the first two months and then every 8 weeks thereafter
- HIV-RNA Cepheid test at baseline, Week 24, Week 48, Week 72, Week 96 and reinduction
- Plasma will be stored at each visit for future use to evaluate use of alternate HIV assays and cabotegravir PK. Small hair samples will be stored at visit weeks 8, 24, 32, and 48 to assess cabotegravir PK.
- Participants that stop CAB-LA prior to the end of the extension time period will continue to be followed in the study but *not* be actively followed beyond the 96 week followup time of the study.
  - Oral PrEP (TDF/3TC) will be recommended to cover tail
  - Participants will switch back to Q12 week follow-up

Table 5. Schedule of Evaluations Dynamic Choice Prevention Extension – Baseline to Week 48

	Screening/ Baseline	Week 12	Week 24	Week 36	Week 48[4]
**DCP Intervention (For persons not receiving CAB-LA)[1]**					
Study visit	X	X	X	X	X
DCP intervention, including:	X	X	X	X	X
- Offer PrEP, PEP, prevention options[2]	X	X	X	X	X
- HIV testing options	X	X	X	X	X
- Visit location choice	X	X	X	X	X
- Structured assessment of barriers	X	X	X	X	X
- GBV screening	X		X		X
HIV self-test (alternative to rapid)		X	X	X	
Rapid (blood-based) HIV test	X	X	X	X	X
STI symptom screen	X	X	X	X	X
Pregnancy testing (optional)	X	X	X	X	X
**DCP (including those persons on CAB-LA) and SOC**					
Measure height (baseline only) and weight	X		X		X
Assess self-reported PrEP/PEP use and HIV risk over prior 6 months	X		X		X
Complete Week 24 or 48 Visit eCRF			X		X
Hair sample collection if any PrEP or PEP doses taken in last 30 days			X		X
HIV RNA			X		X
**SOC Only**					
Study Visit	X		X		X
Referral to standard prevention options	X		X		X
Rapid (blood-based) HIV test	X		X		X
**Suspected seroconversion[3]**					

[1]Late visits for routine assessments will not be considered Protocol Violations, allowing flexibility during the COVID-19 pandemic
[2] This assessment will be done on all intervention participants at baseline
[3] See Figure 2
[4] Week 48 visits may occur between 40 and 56 weeks after Baseline

If CAB-LA is not available in country at the 48-week endpoint, all participants in the intervention arm will have the opportunity to conitinue on the study until week 96 or until CAB-LA is available in country, whichever occurs first, according to the schedules of evaluation described in Tables 5a and 6. Participants who are currently on CAB-LA at the Week 48 visit may continue on CAB-LA through Week 96. CAB-LA may not be initiated or restarted during the additional 48 week extension to Week 96. Prior to conducting procedures for visits after Week 48, participants in the intervention arm will be reconsented to extend their participation up to Week 96.

Table 5a. Schedule of Evaluations for Extension of Intervention Arm – Week 60 to Week 96

	Week 60	Week 72	Week 84	Week 96[3]
**DCP Intervention (For persons not receiving CAB-LA)[1]**				
Study visit	X	X	X	X
DCP intervention, including:	X	X	X	X
- Offer PrEP, PEP, prevention options[2,*]	X	X	X	X
- HIV testing options	X	X	X	X
- Visit location choice	X	X	X	X
- Structured assessment of barriers	X	X	X	X
- GBV screening		X		X
HIV self-test (alternative to rapid)	X	X	X	
Rapid (blood-based) HIV test	X	X	X	X
STI symptom screen	X	X	X	X
Pregnancy testing (optional)	X	X	X	X
Measure weight		X		X
Assess self-reported PrEP/PEP use and HIV risk over prior 6 months		X		X
Complete Week 72 or 96 Visit eCRF		X		X
HIV RNA		X		X
**Suspected seroconversion[2]**				

[1]Late visits for routine assessments will not be considered Protocol Violations, allowing flexibility during the COVID-19 pandemic or for other outbreaks.
[2] See Figure 2
[3] Week 96 visits may occur between 88 and 104 weeks after Baseline
*Participants will not be given the option to initiate or restart CAB-LA after the Week 48 visit; only other prevention options will be available.

Table 6. Schedule of Evaluations for CAB-LA users (weeks indexed by date of initial CAB LA injection) – Baseline to Week 96

	CAB Screening/ Baseline	CAB Week 4	CAB Week 8	CAB Week 16	CAB Week 24	CAB Week 32	CAB Week 40	CAB Week 48[7]	CAB Week 56	CAB Week 64	CAB Week 72	CAB Week 80	CAB Week 88	CAB Week 96[7]
**Measure weight**	X				X			X			X			X
**CAB LA injection**	X	X	X	X	X	X	X	X	X	X	X	X	X	
**STI Symptom Screen**	X	X	X	X	X	X	X	X	X	X	X	X	X	X
**Suspected Seroconversion**	X	X	X	X	X	X	X	X	X	X	X	X	X	X
**HIV Testing**														
-Rapid test (Country std. algorithm)	X	X	X	X	X	X	X	X	X	X	X	X	X	X
-HIV RNA[6]	X				X			X			X			X
-Stored plasma (2cc) for future HIV testing	X	X	X	X	X	X	X	X	X	X	X	X	X	X
**CAB PK**														
-Hair sample (if CAB-LA received in last 120 days)			X		X	X		X						
-Stored plasma (from 2 cc sample above)		X	X	X	X	X	X	X						
**Safety Labs**														
-Pregnancy testing[1]	X	X	X	X	X	X	X	X	X	X	X	X	X	X
-ALT Monitoring[4, 5]	X		X*		X			X*			X*			X*
**Adverse Event Monitoring**	X	X	X	X	X	X	X	X	X	X	X	X	X	X
**GBV Screening**	X				X			X			X			X
**Survey and IDI**														
-Participant CAB-LA Survey and IDI[2]	X				X			X						
-Provider and Clinic Staff DCP-LA Survey and IDI	X				X			X						
**Costing**[3]	X	X				X								
**For participants who stop CAB LA before end of extension study**														
-For 48 weeks from last injection: Recommend oral PrEP (TDF/3TC) to cover tail														
-Switch back to Q12 week visits - HIV RNA and pregnancy testing Q12 weeks														

[1]For women who become pregnant, repeat pregnancy tests are not needed. Women who become pregnant during the study will be followed to delivery for assessment of mother and infant status

[2] Participant survey/IDI will be conducted at start of CAB-LA and Weeks 24 and 48 from time of CAB-LA start or discontinuation.

[3]Costing will occur between weeks 24-48.

[4]ALT Monitoring will occur at baseline, Week 24 for all participants. *Per provider discretion, ALT monitoring will occur at Weeks 8, 48, 72 and 96.

[5]Bilirubin and additional labs indicated will be tested in the case of ALT increase.

[6]HIV RNA will be drawn at CAB-LA reinduction, as described in Table 6.

[7] Week 48 visits may occur between 40 and 56 weeks after Baseline; Week 96 visits may occur between 88 and 104 weeks after Baseline; only participants on CAB-LA at the Week 48 visit may take CAB-LA between weeks 48 and 96.

E. Extension Consent Process

All participants in the DCP trials will be offered participation in the CAB-LA Extension. Consent forms will be translated from the original English to the language(s) spoken in the community. The consent form will be read to participants in their local language. Participants who agree to take part and sign the consent form will continue in the study. Participants aged 15-17 years old who meet the definition of 'mature or emancipated minors' as set by the Uganda National Council for Science and Technology and the Kenya MOH can participate in these studies and will be consented on the adult participant consent forms (refer to section 9.2.1.3 of the protocol). Participants aged 15-17 that do not meet the definition of 'mature or emancipated minors' may participate with parental co-signature. All participants will be asked to re-consent for the CAB-LA extension. In this consent, women will be informed about the risks and benefits of CAB-LA during pregnancy and breastfeeding In the case that participants become pregnant while using CAB-LA, they will be asked for an additional re-consent to follow-up for ascertainment of pregnancy and infant outcomes.

Participants will remain in the randomization arm they were assigned to at their initial DCP visit in Phase A (refer to section 9.1.1 of the protocol). Participants in the SOC arm will consent to return for two additional follow-up visits at weeks 24 and 48 following re-consent for the CAB-LA extension. Intervention participants that do not choose CAB-LA will consent to continue the quarterly study visits and Intervention participants that choose CAB-LA will have study visits that align with their injection visits (every 4 weeks for the first two months and then every 8-weeks thereafter). Intervention participants will have flexibility to switch between PrEP, PEP, and CAB-LA. Unlimited switches between biomedical prevention options, including between CAB-LA and oral, will continue to be permitted during the extension, both to reflect participant choice and to bridge planned missed doses of CAB-LA. Clients and providers will discuss options for prevention and risk during regular visits and interim visits, in addition to continuous provider trainings on offering choice and refreshers for clients. Changes in prevention choices will be documented in study eCRF forms, which will include the CAB-LA option. DCP intervention participants that choose the CAB-LA option will be informed about the visit schedule, side effects, and risks and benefits, before consenting. If CAB-LA becomes available via the Ministry of Health during the trial, persons in the control arm will be eligible as per standard of care.

Documentation of written informed consent and verbal consent will be recorded on informed consent logs for all study activities. The logs will contain the participant's study ID, clinic ID, initials, name, language of consent used, and whether the individual signed the document or provided a fingerprint in the case of written consents, or verbally affirmed their consent in the case of verbal consents.

Stored Samples and Associated Data Considerations

Plasma samples will be collected and stored for retrospective HIV testing and PK.

Hair samples collected from participants on PrEP, CAB-LA or PEP in the extension will be collected and stored for later measurements of HIV-prevention drug levels, which will be conducted at the UCSF Hair Analytical Lab in the United States.

Blood samples from persons who acquire HIV in Phase A prevention trials will be stored for additional testing for HIV diagnostics (if indicated0 and HIV drug resistance. Testing will be performed in-country when available (e.g. standard genotyping for HIV drug resistance).

Specialized research assays for diagnostic and resistance testing (e.g. next-generation sequencing) will be performed at the University of Pittsburgh Parikh Lab in the United States. Blood and urine samples will be stored for testing CAB-LA/PrEP/PEP drug levels performed at the UCSF Hair Analytic Lab in the United States.

Specimens may be held up to five years in order to complete HIV diagnostics, resistance, or PK tests as outlined in this protocol. The study may hold specimens to test in batches at later time since these tests are being performed solely for research, not for clinical care, or for a biobank for future use. Specimens will be destroyed after tests are performed.

All other biological samples collected during the study will be tested shortly after collection and any remaining specimen discarded.

F. Cabotegravir (GSK1265744A) Injectable Suspension- for intramuscular use: Product information

2. CAB-LA will be supplied by the ViiV Healthcare Limited Pharmaceutical Company
3. **Indications and Usage.** CAB-LA is indicated in at-risk adults and adolescents weighing at least 35 kg for pre-exposure prophylaxis (PrEP) to reduce the risk of sexually acquired HIV-1 infection. Individuals must have a negative HIV-1 test prior to initiating CAB-LA. CAB-LA will not be used as a supplement for PEP.
4. **Administration Overview.**
   a. CAB-LA will be offered as direct to inject only (no oral lead-in)
   b. Injection: Single-dose vial containing 600 mg/3 mL (200 mg/mL) of cabotegravir is a white to light pink, free-flowing, extended-release injectable suspension
   c. CAB-LA will be administered by trained healthcare staff by gluteal intramuscular injection only. Body Mass Index will be considered to ensure appropriate needle length is sufficient to reach the gluteal muscle.
   d. Study participants will be tested for HIV infection prior to initiating CAB-LA, and with each subsequent injection of CAB-LA, including HIV RNA testing (Cepheid).
   e. Prior to starting CAB-LA, healthcare staff will counsel study participants about the importance of adherence to scheduled dosing visits to help reduce the risk of acquiring HIV infection and development of resistance.
5. **Pharmacy**
   a. The study sponsor, UCSF, will receive CAB-LA directly from ViiV Healthcare and distribute to the country pharmacies per country study drug shipping procedures
   b. In-country pharmacists will receive and log the CAB-LA per country procedure and detailed in the CAB-LA Pharmacy SOPs
   c. As described in 6c below, CAB-LA will be stored at 2°C to 30°C (36°F to 86F) at clinic sites; proper storage and handling will be monitored by in-country pharmacy teams and validated by accountability logs and other source

documents

d. Details on these procedures are outlined in the Standard Operating Procedures.
    i. CABPHARM-01 Study Drug Ordering and Acquisition
    ii. CABPHARM-02 Study Drug Accountability
    iii. CABPHARM-03 Study Drug Storage
    iv. CABPHARM-04 Dispensing and Administration of Study Drug
    v. CABPHARM-05 Disposition and Destruction of Study Drug
    vi. CABPHARM-06 Communication and Dissemination of Information to the Study Pharmacist

6. **Injection Dosing**

    a. The initial injection dose of CAB-LA is a single 600-mg (3-mL) intramuscular injection of CAB-LA given 4-weeks apart for 2 consecutive times

    b. After the 2 initiation injection doses given consecutively 4-weeks apart, the continuation injection dose of CAB-LA will be a single 600-mg (3-mL) intramuscular injection of CAB-LA, every 8-weeks.

    c. There is a +/- 7 day window for before or after the date the participant is scheduled to receive their next injection

7. **Missed Doses.** Study staff will provide adherence support to CAB-LA participants. When doses are missed, study staff will follow the applicable guidelines. Providers will work pro-actively and prospectively identify periods during which participants are likely to miss doses (eg travel). Participants will be provided with oral bridging prevention medication (TDF-FTC) to cover these periods. Upon return to clinic for planned or unplanned missed doses, CAB-LA dosing will be as per protocol below.

    a. If a scheduled injection visit is missed or delayed by more than 7 days, the study clinician will determine when resumption is appropriate.

    b. Recommended Dosing after a missed visit is detailed in **Table 7**.

Table 7. Missed Dosage Recommendations

Time since Last Injection	Recommendation
**If second injection is missed and time since first injection is:**	
Less than or equal to 8-weeks	Administer 600-mg (3-mL) gluteal intramuscular injection of CAB-LA as soon as possible, then continue to follow the every 8-weeks injection dosing schedule.
Greater than 8-weeks	Restart with 600-mg (3-mL) gluteal intramuscular injection of CAB-LA, followed by a second 600-mg (3-mL) initiation injection 4-weeks later. Then continue to follow the every 8-weeks injection dosing schedule thereafter.

If third or subsequent injection is missed and time since prior injection is:	
Less than or equal to 12 weeks	Administer 600-mg (3-mL) intramuscular injection of CAB-LA as soon as possible, then continue with the every 8-weeks injection dosing schedule.
Greater than 12-weeks	Restart with 600-mg (3-mL) gluteal intramuscular injection of CAB-LA, followed by the second 600-mg (3-mL) initiation injection dose 4-weeks later. Then continue with the every 8-weeks injection dosing schedule thereafter.

4. **Contraindications.** CAB-LA is contraindicated in individuals:
   a. With unknown or positive HIV-1 status
   b. With previous hypersensitivity reaction to cabotegravir
   c. Receiving the following co-administered drugs for which significant decreases in cabotegravir plasma concentrations may occur due to uridine diphosphate glucuronosyltransferase:
      i. Anticonvulsants: Carbamazepine, oxcarbazepine, phenobarbital, phenytoin
      ii. Antimycobacterials: Rifampin, rifapentine
5. **Drug Interactions**
      i. **Use of Other Antiretroviral Drugs after Discontinuation of CAB-LA.** Residual concentrations of cabotegravir may remain in the systemic circulation of individuals for prolonged periods (up to 12 months or longer). These residual concentrations are not expected to affect the exposures of antiretroviral drugs that are initiated after discontinuation of CAB-LA
      ii. **Potential for Other Drugs to Affect CAB-LA.** Cabotegravir is primarily metabolized by UGT1A1 with some contribution from UGT1A9. Drugs that are strong inducers of UGT1A1 or 1A9 are expected to decrease cabotegravir plasma concentrations; therefore, coadministration of CAB-LA with these drugs is contraindicated
      iii. **Established and Other Potentially Significant Drug Interactions.** Information regarding potential drug interactions with cabotegravir is provided in **Table 8** below.

Table 8. Drug Interactions with CAB-LA

Concomitant Drug Class: Drug Name	Effect on Concentration	Clinical Comment

**Anticonvulsants:**<br>Carbamazepine<br>Oxcarbazepine<br>Phenobarbital<br>Phenytoin	↓ Cabotegravir	Coadministration is contraindicated with CAB-LA due to potential for significant decreases in plasma concentration of CAB-LA.
**Antimycobacterials:**<br>Rifampin Rifapentine	↓ Cabotegravir	
**Antimycobacterial:**<br>Rifabutin	↓ Cabotegravir	When rifabutin is started before or concomitantly with the first initiation injection of CAB-LA, the recommended dosing of CAB-LA is one 600-mg<br>(3-mL) injection, followed 2 weeks later by a second 600-mg (3-mL) initiation injection and monthly thereafter while on rifabutin.<br>When rifabutin is started at the time of the second initiation injection or later, the recommended dosing schedule of CAB-LA is 600 mg (3 mL) monthly while on rifabutin.<br>After stopping rifabutin, the recommended dosing schedule of CAB-LA is 600 mg (3 mL) every 8-weeks.

6. **Drugs without Clinically Significant Interactions with Cabotegravir.** The following drugs can be co-administered with cabotegravir (non-antiretrovirals) or given after discontinuation of cabotegravir (antiretrovirals and non-antiretrovirals) without a dose adjustment: etravirine, midazolam, oral contraceptives containing levonorgestrel and ethinyl estradiol, and rilpivirine.
   a. **Special Populations**
      i. CAB-LA will be used during pregnancy if the expected benefit justifies the potential risk to the fetus. Factors to consider include the long acting nature of CAB-LA, the actual gestational age, adherence, the risk of seroconversion and available data around the safety of study drugs and alternative PrEP regimens in pregnancy and breastfeeding.
      ii. In the HPTN 084 trial evaluating cabotegravir long-acting (LA) for pre-exposure prophylaxis (PrEP) in women in sub–Saharan Africa, no birth defects were reported of the 63 women that became pregnant while taking CAB-LA. Given the need for more

data on pregnancy and breastfeeding while taking CAB-LA, participants will be monitored closely for side effects during pregnancy and breastfeeding during the study and up to 8 weeks post-partum.

iii. Pregnant participants will sign a consent to be monitored for up to 8-weeks postpartum to ascertain the pregnancy and infant outcomes

iv. Participants will be registered in the Antiretroviral Pregnancy Registry to contribute to the global scientific community's understanding of CAB-LA use in pregnancy and breastfeeding

v. It is recommended that participants of child bearing potential taking CAB LA should use an effective contraceptive method during the study, through any additional extension phase and for 52 weeks after the last injection if transitioning off CAB-LA, to avoid pregnancy. Participants that are currently on a contraceptive method at CAB-LA start will be informed of the risks and benefits of CAB-LA during pregnancy, if they chose to stop using contraceptives. Participants not on a contraceptive method when starting CAB-LA will be informed about the risks and benefits to CAB-LA during pregnancy if they become pregnant. CAB-LA will not be given to women who are pregnant at time of initial injection; women who become pregnant after starting CAB-LA will be allowed to remain on CAB-LA during pregnancy, as deemed appropriate by their provider, and will be re-consented for additional follow-up of pregnancy and infant outcomes.

vi. It is not known if cabotegravir is present in human breast milk, affects human milk production, or has effects on the breastfed infant. Participants who are currently breastfeeding will be informed of the risks and benefits in the consenting process, and unknown effects on infants of exposure to CAB-LA through breastmilk.

vii. No dosage adjustment of CAB-LA is necessary for participants with mild (creatinine clearance 60 to <90 mL/min) or moderate renal impairment (creatinine clearance 30 to <60 mL/min). In participants with severe renal impairment (creatinine clearance 15 to <30 mL/min) or end-stage renal disease (creatinine clearance<15 mL/min), increased monitoring for adverse effects will be provided

viii. No dosage adjustment of CAB-LA is necessary for participants with mild or moderate hepatic impairment

b. **Overdosage.** There is no known specific treatment for overdose with CAB-LA. If overdose occurs, the participant will be treated appropriately, and further CAB-LA continuation determined by the Investigator.

c. **Storage and Handling.**

   i. CAB-LA will be stored at 2°C to 30°C (36°F to 86°F) in the original carton until ready to use. Exposure up to 30°C is permitted, but freezing is not. CAB-LA cannot be mixed with any other product or diluent.

   ii. If the pack has been stored in the refrigerator, the vial will be brought to room temperature prior to administration (not to exceed 30°C [86°F]).

   iii. Once the suspension has been drawn into the syringe, the injection will be administered as soon as possible, but may remain in the syringe for up to 2 hours. The filled syringes will not be placed in the refrigerator. If the medicine remains in the syringe for more than 2 hours, the filled syringe and needle will be discarded

d. **Possible Side effects:**

   i. Allergic reactions:
      - generally ill feeling
      - blisters
      - tiredness
      - muscle or joint aches
      - trouble breathing
      - fever
      - blisters or sores in the mouth
      - redness or swelling of eves
      - swelling of the mouth, face lips or tongues

   ii. Liver problems

   iii. Depression or Mood Changes

   iv. Most Common Side Effects:
      - Pain, tenderness, hardened mass or lump, swelling, bruising, redness, itching, warmth, loss of sensation at the injection site, abscess, and discoloration
      - stomach pain
      - vomiting
      - diarrhea
      - muscle pain
      - headache
      - rash
      - fever
      - loss of appetite
      - tiredness
      - drowsiness
      - sleep problems
      - back pain
      - nausea
      - upper respiratory infection

G. Toxicity Management.

CAB-LA was very well tolerated in the registration studies. For this extension, SAEs will be captured in the intervention arm and Grade 3 and 4 AEs, and non-serious ADRs leading to withdrawal will be captured for participants on CAB-LA. Grade 3 and 4 AEs will be graded and recorded using the NIAID Adverse Event Grading of Severity scale (https://rsc.niaid.nih.gov/clinical-research-sites/daids-adverse-event-grading-tables). Clinical Adverse Events not identified elsewhere are described in **Table 10.** The Clinical Management Team (CMT) will be notified of SAEs as soon as possible and will be reported per regulatory guidelines as stated in **Table 9** below.

Table 9. Reporting Guidelines

Institution	Contact information	Type of Event	Reporting Timeframe	Reporting Method
**UCSF IRB**	UCSF Committee for Human Research<br>3333 California Street, Suite 315<br>University of California<br>San Francisco, CA 94118<br>Campus Mailbox: 0962<br>Facsimile: (415) 502-134	SAEs/ Grade 3 or higher AEs that change the study risks or benefits, OR necessitates modification to the IRB-approved consent document(s), and/or the IRB-approved application/protocol	Within 10-working days of awareness	Via online reporting system
**NIAID**	Melanie Bacon, Program Officer, NIH	SAEs or Harm determined to be possibly, probably or definitely related to study intervention<br><br>Participants administered CAB-LA:<br><br>All pregnancies	At the time of reporting to IRBs<br><br>One-week of awareness (Pregnancies)	Email
**ViiV**	GSK Medical Information, Safety Services & Vendor Management Group<br><br>oax37649@gsk.com	Participants administered CAB-LA:<br><br>All SAEs and Significant Safety Issues<br><br>All pregnancies<br><br>All Liver Events Grade 3 and higher	Within 72 hours of awareness (SAEs and Significant Safety Issues)<br><br>One-week of awareness (Pregnancies and Liver Events)	All SAEs and Significant Safety Issues: Email notification with SEARCH SAPPHIRE AE CRF, Country-specific SAE Reporting Forms, and attachment with supplemental information<br><br>Pregnancies: Email notification with

				Pregnancy Reporting CRF<br><br>Liver Events (Grade 3+): Email notification with Liver Reporting CRF
**KEMRI-SERU**	The Scientific and Ethics Review Unit Through The Deputy Director CMR at seru@kemri.org.	All reportable SAEs, SUSARs, and Harm	Notify/ report to SERU secretariat within 48 hours upon learning of the event and submit the full report within 5 working days of awareness	Via online reporting system to SERU secretariat
**PPB**	Pharmacy and Poisons Board	All reportable Grade 3 or higher SAEs, SUSARs, and Harm	Report to PPB within 7 working days of awareness	Via online reporting system to PPB ECCT (Expert Committee on Clinical Trials)
**MU-SOMREC**	Executive Secretary Faculty of Medicine, Research and Ethics Committee P.O. Box 7072 Kampala	All serious and unexpected events irrespective of relationship	Fatal and life threatening events within 3 working days of awareness and all other SAEs within 7 calendar days	Via Regulatory Manager
**NDA**	National Drug Authority	All serious and unexpected events irrespective of relationship	Within 7 days of awareness	Via online reporting

<u>Grade 3</u>

Any grade 3 or higher clinical or laboratory AE observed prior to their first injection of active CAB will prompt consultation with the clinical officer (CO) prior to any injectable dosing.

At any time, participants who develop a Grade 3 AE or toxicity that is not specifically addressed in the Table below and is judged to be <u>related to CAB-LA by the Investigator</u>, CAB-LA use will be temporarily discontinued. In general, the investigator will re-evaluate the participant until resolution of the toxicity. For Grade 3 AEs deemed <u>related to CAB-LA</u>, will be permanently discontinued if improvement to severity ≤Grade 2 cannot be documented within 4 weeks of awareness. If CAB-LA is resumed after holding for a Grade 3 AE and the same Grade 3 AE recurs without alternative explanation, CAB-LA will be permanently discontinued. For Grade 3 AEs deemed unrelated to CAB-LA, the participant may continue with appropriate clinical management by the site, per local standards of care.

<u>Grade 4</u>

Any grade 4 or higher clinical or laboratory AE observed prior to first injection will prompt evaluation and may requiring holding of dosage, as determined by the CO and CMT. Participants who develop a Grade 4 AE or toxicity that is not specifically addressed in **Table 11** (regardless of relationship to CAB-LA) will have the CAB-LA injections temporarily discontinued. CAB-LA use will not be resumed if the Grade 4 AE is considered by the Investigator to be related to the injections. If CAB-LA use is resumed and the same Grade 4 AE recurs at Grade 4 level at any time, CAB-LA will be permanently discontinued, unless an alternative explanation for the recurrence is clearly documented.

Pregnancy

Any pregnancy that occurs during study participation must be reported using the Pregnancy Reporting Case Report form to NIAID and ViiV Healthcare within **one week** of learning of its occurrence. Pregnancies that are associated with SAEs will be reported within 72 hours using the Adverse Event CRF, Country-specific SAE Reporting form, and supplemental information attached.

The pregnancy must be followed up to determine outcome (including premature termination) and status of mother and child(ren), which must also be reported to NIAID and ViiV Healthcare using the Pregnancy Outcomes case report form. The pregnancy outcome should also be reported to ViiV Healthcare in accordance with the terms of the contract.

Pregnancy complications and elective terminations for medical reasons must be reported as an adverse event or serious adverse event. Spontaneous abortions must be reported as a serious adverse event in accordance with SAE reporting timelines.

Any serious adverse event occurring in association with a pregnancy brought to the investigator's attention after the participant has completed the study and considered by the investigator as possibly related to the investigational product, must be promptly reported to NIAID and ViiV Healthcare.

Table 10. DAIDS Table for Grading the Severity of Adult and Pediatric Adverse Events

PARAMETER	GRADE 1 MILD	GRADE 2 MODERATE	GRADE 3 SEVERE	GRADE 4 POTENTIALLY LIFE-THREATENING
**Clinical** adverse event **NOT** identified elsewhere in the grading table	Mild symptoms causing no or minimal interference with usual social & functional activities with intervention not indicated	Moderate symptoms causing greater than minimal interference with usual social & functional activities with intervention indicated	Severe symptoms causing inability to perform usual social & functional activities with intervention or hospitalization indicated	Potentially life-threatening symptoms causing inability to perform basic self-care functions with intervention indicated to prevent permanent impairment, persistent disability, or death

General Criteria for Discontinuation of CAB-LA

Participants may voluntarily discontinue CAB-LA for any reason at any time. Investigators will permanently discontinue participants from CAB-LA per protocol for any of the specific

criteria below. Investigators also may permanently discontinue participants for reasons not shown here (e.g., to protect participants' safety and/or if participants are unable or unwilling to comply with CAB-LA use procedures).

The criteria for permanent discontinuation of CAB-LA use for an individual participant are:

- CAB-LA -related toxicity requiring permanent discontinuation per the guidelines above and below
- Request by participant to permanently terminate use of CAB-LA
- Clinical reasons determined by the Investigator
- Acquires HIV infection

CAB-LA will be temporarily withheld from a participant for any of the following reasons:

- Report of use of prohibited concomitant medications. CAB-LA injections may resume when the participant reports that he/she is no longer taking the prohibited medication, provided other reasons for temporary hold/permanent discontinuation do not apply.
- The participant is unable or unwilling to comply with required study procedures such as HIV testing and routine laboratory assessments, or otherwise might be put at undue risk to their safety and well-being by continuing CAB-LA use, according to the judgment of the Investigator.
- The participant has one or more reactive HIV test results or expresses a concern about having acute HIV infection.

Guidance on Toxicity Management for Specified Toxicities:

Table 11: Nausea, Vomiting, and Diarrhea

CONDITION AND SEVERITY	IMMEDIATE ACTION	FOLLOW-UP AND MANAGEMENT
**Nausea, Vomiting, and Diarrhea**		
Grade 1 and 2	Continue CAB-LA	Will be treated symptomatically with hydration, oral antiemetic therapies or antiemetic suppositories at the discretion of the Investigator. The Investigator will order any clinically relevant laboratory analyses (per judgment of the Investigator).

Grade ≥ 3	Discontinue CAB-LA temporarily	Participants with Grade 3 nausea, vomiting, or diarrhea, for which an alternative etiology is not established, will discontinue CAB-LA temporarily until Grade 2 or lower and be treated symptomatically. Should condition(s) not improve to Grade ≤ 2 within 7 days, the Investigator will consider temporary discontinuation or progressing to permanent discontinuation of CAB-LA.

Liver chemistry monitoring

Liver chemistry monitoring is at the discretion of the provider and will occur at baseline and Week 24. Additional ALT measurements will be taken and Weeks 8 and 48 per provider discretion. Cabotegravir will be stopped if certain protocol specified liver event stopping criteria are met, or hepatotoxicity is suspected.

Liver Event Stopping Criteria

CAB must be stopped if any of the following Liver Event Stopping Criteria are met, or if the Investigator believes that it is in the best interests of the participant:

- ALT >= 3xULN **AND** bilirubin >= 2xULN
- ALT >=5xULN for more than 2 weeks
- ALT >=8xULN
- ALT >=3xULN (or >=3x baseline ALT) with symptoms of worsening of acute hepatitis or hypersensitivity such as fatigue, nausea, vomiting, right upper quadrant pain or tenderness, fever, rash or eosinophilia

Consideration should also be given to stopping CAB in any participant with aminotransferase elevations > 3x ULN who cannot be monitored every 1-2 weeks.

When a Liver Event Stopping Criterion is met, the treating physician must do the following:

- **Immediately stop CAB-LA**
- If ALT >= 3xULN **and** bilirubin >=2xULN, report as a serious adverse event (SAE) within **72 hours** using the SAE case report form.
- For **all** events meeting Liver Event Stopping Criteria, perform repeat liver chemistries and liver event follow up assessments (ideally within 24 hours of knowledge of the liver chemistry elevations) as described in the Follow-Up Assessment below and report to UCSF, NIAID, and ViiV Healthcare using the Liver Event Case Report form within one week of first becoming aware of the event.
- A specialist or hepatology consultation is recommended if available.

- Monitor participants 2 times a week until liver chemistries resolve, stabilise or return to within baseline values.

Follow up Assessment following ANY Liver Stopping Event

Make every attempt to carry out liver event follow-up assessments as described below for the laboratory tests that are available locally.

- Viral hepatitis serology, including:
- Hepatitis A immunoglobulin M (IgM) antibody;
- HBsAg and hepatitis B core antibody;
- Hepatitis C RNA;
- Hepatitis E IgM antibody.
- Cytomegalovirus IgM antibody.
- Epstein-Barr viral capsid antigen IgM antibody (or if unavailable, obtain heterophile antibody or monospot testing).
- Syphilis screening.
- Drugs of abuse screen, including alcohol. Record alcohol use on the liver event case report form if using.
- Serum acetaminophen/paracetamol levels
- Serum creatinine phosphokinase (CPK) and lactate dehydrogenase (LDH).
- Fractionate bilirubin, if total bilirubin >=1.5xULN.
- Obtain complete blood count with differential to assess eosinophilia.
- Anti-nuclear antibody, anti-smooth muscle antibody, Type 1 anti-liver kidney microsomal antibodies, and quantitative total immunoglobulin G (or gamma globulins).
- Gamma glutamyl transferase (GGT), glutamate dehydrogenase (GLDH), and serum albumin
- International normalised ratio (INR)
- Liver imaging (ultrasound, magnetic resonance, or computerised tomography) and /or liver biopsy to evaluate liver disease. [A Liver Imaging and/or Liver Biopsy Case Report Form is available if required].
- Obtain history and record the appearance or worsening of clinical symptoms of liver injury, or hypersensitivity, fatigue, decreased appetite, nausea, vomiting, abdominal pain, jaundice, fever rash as relevant on the AE/SAE report form.
- Obtain history and record use of concomitant medications including acetaminophen, herbal remedies, other over the counter medications or putative hepatotoxins.

Liver-Safety- CAB-LA Restart

“Drug restart” refers to resuming study treatment in which there is a clear cause other than Drug Induced Liver Injury (DILI). For long acting agents, a restart may actually be a continuation of therapy rather than a true restart due to the timeframe between dose administration relative to decision making on allowing a restart.

Participants who meet Liver Event Stopping Criteria should NOT restart CAB-LA unless there is a compelling alternative cause for the liver chemistry abnormalities.

“Drug restart” with CAB-LA in a participant who has a Liver Stopping Event may be permitted when liver chemistries improve to within 1.5x baseline and ALT < 3 xULN, provided the criteria set out in the Liver Safety Restart Guidelines are met. This should take into account when the next scheduled injection of CAB LA is due. If the restart criteria are not met by the time window for the next injection, consideration should be made to transitioning participants onto an alternative regimen.

If restart is **not allowed or not granted** by the Study’s Governing Body or Principal Investigator, permanently discontinue study treatment. Participants may continue participation in the study for any protocol specified follow up assessments.

Liver Safety Restart Guidelines

If a causal relationship between the liver event and CAB-LA cannot be ruled out, then CAB-LA must be permanently discontinued and the participant not restarted.

Note: Any decision regarding potential restart should take into account when the next scheduled injection of CAB LA is due. If the restart criteria below are not met by the time window for the next injection(s), consideration should be made to transitioning participants onto an alternative regimen.

CAB-LA Restart Following Transient Resolving Liver Events Not Related to CAB-LA

Restart can be considered when liver chemistries improve to within 1.5x baseline and ALT<3xULN where:

- Liver chemistries have a clear underlying cause other than drug-induced liver injury (e.g. biliary obstruction, pancreatic events, hypotension, acute viral hepatitis). Furthermore, there should be no evidence of alcoholic hepatitis or hypersensitivity, and the drug is not associated with HLA markers of liver injury.
- The participant is receiving compelling benefit and the benefit of drug restart exceeds risk. The long acting nature of CAB LA should be taken into consideration.
- Approval from the Principal Investigator and Ethics Committee or Institutional Review Board for the drug restart has been obtained.
- The participant has been provided with a clear description of the possible benefits and risks of drug restart, including the possibility of recurrent, more severe liver injury or death and the long acting nature of CAB injections.
- The participant has also provided signed informed consent specifically for the restart. Documentation of informed consent must be recorded in the study file.

- Following drug restart, the participant will return to the clinic once a week for liver chemistry tests for one month or for as long as clinically indicated and then laboratory monitoring may resume as per protocol. If protocol defined stopping criteria for liver chemistry elevations are met, study drug must be permanently stopped.

Hypersensitivity

Hypersensitivity reactions have been reported in association with other integrase inhibitors and were characterized by rash, constitutional findings and sometimes organ dysfunction, including liver injury. Administration of CAB oral lead-in was used in clinical studies to help identify patients who may be at risk of a hypersensitivity reaction. While there have been no confirmed occurrence of a severe hypersensitivity reactions attributable to cabotegravir in clinical trials to date, investigators should remain vigilant regarding their occurrence and should discontinue CAB-LA and other suspect agents immediately if signs or symptoms of hypersensitivity reactions develop (including, but not limited to, severe rash, or rash accompanied by fever, general malaise, fatigue, muscle or joint aches, blisters, oral lesions, conjunctivitis, facial oedema, hepatitis, eosinophilia or angioedema. Clinical status, including laboratory parameters with liver transaminases, should be monitored and appropriate therapy initiated.

All rash and suspected hypersensitivity events should be assessed with special attention to systemic symptoms, laboratory abnormalities, or mucosal involvement. Close clinical follow-up, including follow-up of laboratory abnormalities, and appropriate medical intervention, including referral to dermatologist as appropriate, should be instituted for these events. The prolonged release characteristics of CAB LA should be taken into consideration when these products are discontinued.

Participants should be instructed to contact the Investigator as soon as possible if they develop a rash or allergic symptoms on study.

Suicidal Ideation

There have been reports of depression and suicidal ideation and behaviours (particularly in patients with a pre-existing history of depression or psychiatric illness) in some people living with HIV being treated with integrase inhibitors. Depression has also been reported in individuals receiving cabotegravir for PrEP.

Participants should be monitored appropriately for any unusual changes in behaviour, before and during the course of the study. Participants with depressive symptoms or suicidal ideation and behaviours on CAB-LA should be promptly evaluated. It is recommended that the investigator consider mental health consultation or referral for participants who experience signs of suicidal ideation or behaviour.

Confirmation of suspected HIV infection

In cases of suspected HIV-infection, clinic staff are trained and use a seroconversion SOP for reference to proceed as depicted in Figure 2. For all cases, the clinical management team is

notified immediately, as simultaneous confirmatory testing, additional sample collection (including stored samples for further HIV testing and PK), counseling, and linkage to ART care are initiated.

Figure 2. HIV Seroconversion Algorithm and Sample Collection

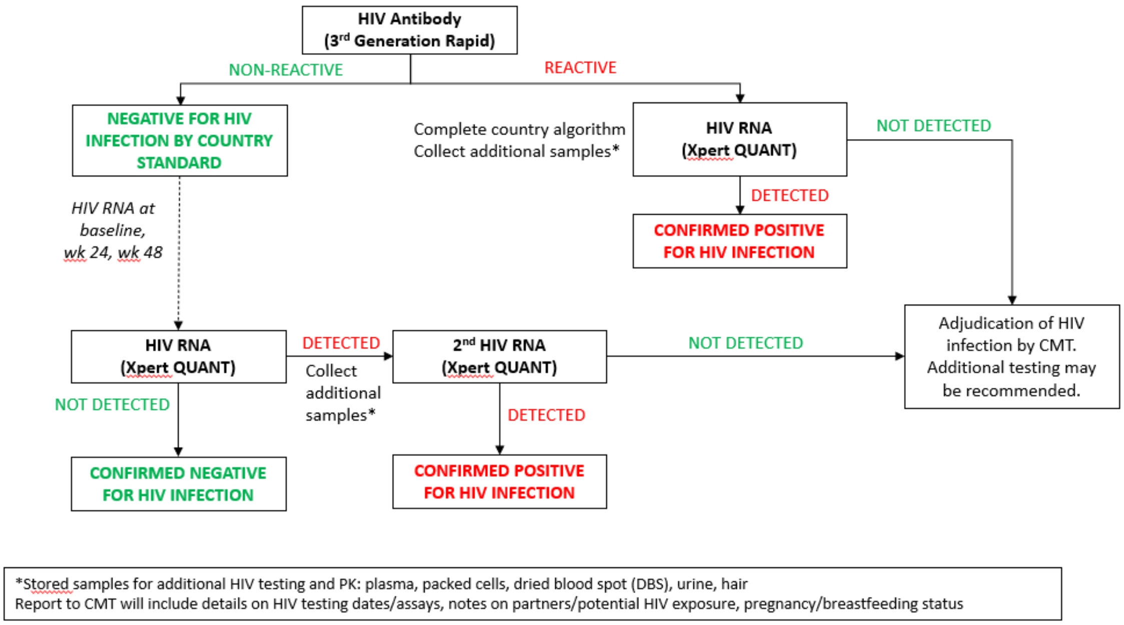

For persons receiving CAB-LA, the following approach will be used for guidance on testing and administration of CAB-LA injections (Figure 3).

Figure 3. Algorithm for participants receiving CAB-LA Injections

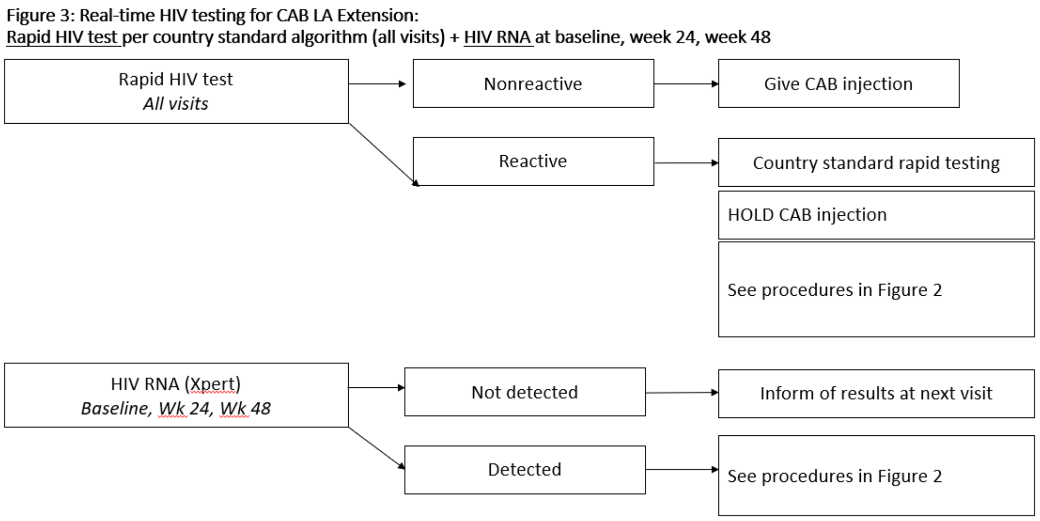

H. Analyses

This section provides an overview of the stastical considerations.

The primary study endpoint in this extension will be biomedical covered time defined as the proportion of follow-up time that the participant is protected from HIV infection by any PEP or PrEP (inclusive of CAB-LA). Follow-up will be over the 48 weeks following re-consent for the CAB-LA extension. At risk covered time will be a secondary outcome, defined as the proportion of at-risk follow-up time that the participant is protected from HIV infection by any PEP or PrEP (inclusive of CAB-LA). We will also conduct sensitivity analyses, varying the definitions of coverage and risk. For example, we will also examine covered time from CAB-LA only (i.e., exclusive of oral PrEP or PEP), and we will conduct a sensitivity analysis in which we treat time after loss to follow-up among persons not known to have moved out of the study region and time after study withdrawal as presumed to be non-covered.

For our primary objective in this extension, we will compare covered time during the 48 week CAB-LA extension between the intervention and control arms. For improved efficiency, Targeted Maximum Likelihood Estimation (TMLE) will be used to adjust for differences in baseline participant characteristics between the two trial arms, accounting for the stratified randomization design. In secondary analyses, we will further use TMLE to i) adjust for measured differences between arms at time of start of the CAB-LA extension period; and, ii) adjust for baseline and time-varying factors that are predictive of the outcome and that differ between persons that do vs. do not have their follow-up time censored. The primary analysis will pool over the trials, accounting for clustering inherent in the community trial; secondary analyses will stratify by trial site. We will test the null hypothesis that covered time is the same between arms with a two-sided test at the 5% significance level.

Our secondary objective to evaluate implementation will follow the RE-AIM framework and corresponding measures (defined in Table 4). Analyses assessing the primary measures of Reach, Adoption, Implementation and Maintenance will be based on standard descriptive statistics. Analyses to evaluate Effectiveness will compare covered time over the 48 week CAB-LA extension period to covered time prior to the CAB-LA extension among intervention participants. We will adjust for measured changes in participant characteristics between these two follow-up periods, using a TMLE designed for serial measures. Measures of control participants will also be used to adjust for temporal trends. We will test the null hypothesis that coverage of the DCP model with CAB-LA is the same as coverage of the DCP model without CAB-LA. We will again use a two-sided test at the 5% significance level. Primary analyses will again pool over trials, while secondary analyses will be stratified by trial. In addition, we will evaluate marginal and adjusted predictors of CAB-LA uptake and persistence. Our tertiary objective to evaluate changes in knowledge, awareness, and acceptability/satisfaction will be based on applying TMLE to survey data, accounting for repeated measures.

These analyses will be complemented with costing evaluations as well as participant and provider qualitative data from IDI.

All analyses of the community-based trial will account for clustering.

Pharmacokinetic analyses:
We will perform pharmacokinetic (PK) analyses of cabotegravir concentrations in plasma and small hair samples with two objectives. 1) To examine hair levels of CAB as predictors of effectiveness; 2) To examine factors associated with CAB PK variability and study correlations between CAB hair and plasma levels at the end of dosing intervals and during the PK tail. In the phase 3 trials of CAB LA, plasma concentrations of CAB did not fully explain CAB breakthrough infections (especially since failures occurred despite on-time injections) and therefore PK metrics, such as hair, that reflect longer-term exposure, may provide important insights regarding CAB exposure in addition to plasma. Drug concentrations of cabotegravir will be analyzed via liquid chromatography tandem mass spectrometry (LS-MS/MS) at the Hair Analytical Laboratory at UCSF. Plasma samples will be collected prior to each injection, while hair samples will be collected at weeks 8, 24, 32, and 48 after CAB LA start.

First, we will analyze hair concentrations of cabotegravir in plasma and small hair samples as predictors of effectiveness. We will take the proximal 2 cm of hair from the scalp and segment the hair into two 1.0 cm segments (reflecting the prior two months of drug exposure in 1 month intervals), analyzing CAB levels in each segment. We will analyze hair levels from each collection visit from all participants who acquire HIV infection after starting CAB LA and three randomly selected controls who did not acquire HIV to compare exposure metrics from those for with and without HIV acquisition. We use sampling fraction weights to properly combine data from participants who acquire HIV (sampled with certainty) and controls in order to obtain accurate estimates for the entire study population.

Second, to examine factors associated with PK variability, we will perform univariate linear mixed effects regression using hair levels of CAB assessed as continuous outcomes with candidate predictor variables that may influence exposure, such as age, sex and body mass index (BMI), as well as plasma levels, and delays in injection visits. We will then build multivariate models to assess how associations with hair levels change when controlled for other factors. We will examine scatterplots and Spearman correlation coefficients between hair and plasma concentrations of CAB, including at the end of the dosing interval (using the hair concentration in the proximal 0.5 cm of hair since hair is collected before injections) and during the PK tail for participants who elect to stop CAB LA or who acquire HIV and start an oral ART regimen.

I. Analyses of In-Depth Interviews

Qualitative in-depth interviews will be conducted among purposively-selected samples of study participants and providers, to ascertain the perceptions of and experiences with CAB-LA use and delivery. Data will analyzed using a framework analysis approach, wherein transcribed and translated data are inductively coded by a cross-regional team of trained qualitative researchers, the initial sets of findings are consolidated and grouped into conceptual categories, and a framework matrix is used to further condense findings tied most salient to the research questions.

J. Costing

Costs will be estimated as the sum of the product of resources (e.g. staff minutes) times unit costs (e.g. compensation levels). "Economic" costs (the true value of resources consumed or "opportunity costs") will be assessed by identifying the value of subsidized or donated resources with information from databases (e.g. wage rates) and donors. We will use prices prevailing at the time of study onset and will convert any locally incurred costs using the Kenya or Uganda Shilling / US dollar exchange rate at that time. Costs of CAB-LA implementation will be compared to the costs of oral PrEP implementation estimated during the primary trial to estimate the incremental cost of CAB-LA implementation.

Power and Sample size considerations

For each ongoing trial, we assume 90% of participants consent to the extension (reflecting the high retention in the ongoing trials seen to date). Of those, we assume 90% are retained through the extension. In the individually randomized ANC and OPD trials, we anticipate >80% power to detect a 11% or greater absolute increase in prevention coverage between arms with 162 participants/arm (assuming σ =0.35). In the cluster-randomized community-based trial, we anticipate >80% power to detect a 15% or greater absolute increase in prevention coverage between arms with ~20 participants in each of the 16 clusters (assuming mean prevention coverage in the standard of care arm of 10%, σ =0.35, and a matched-pair coefficient of variation km=0.25). These calculations are expected to be conservative given the ongoing enrollment and primary approach to conduct a pooled analysis as well as the increased precision from stratified randomization and through covariate adjustment with TMLE. Minimum detectable effect sizes varying these assumptions (including allowing for lower retention in either the ongoing or extension studies) are shown in Figure 4.

Figure 4. Minimum detectable effect sizes for the primary hypothesis

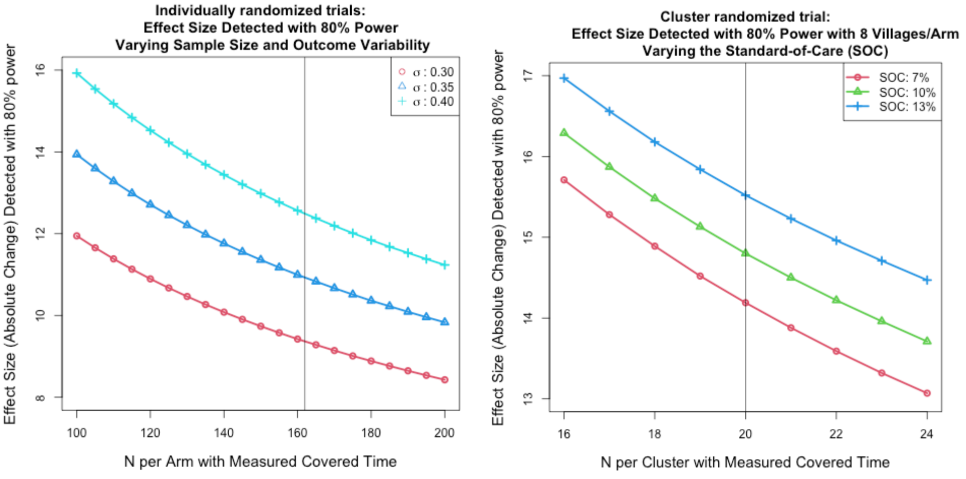

Human participants and data protection. This is an implementation study; the primary risks for a client would be any loss of confidentiality, or risk from blood draw in measurement of the HIV outcome. For risk mitigation, all participants undergo informed consent in their native language, staff are fully trained on confidentiality. The SEARCH Sapphire team has SOPs and procedures for data collection and security. Because CAB-LA is not yet approved in Uganda or Kenya, we will be recording Grade 3 and 4 AEs and reporting to these as required to regulatory authorities.

K. REFERENCES

1. Delany-Moretlwe S, Hughes JP, Bock P, Ouma SG, Hunidzarira P, Kalonji D, Kayange N, Makhema J, Mandima P, Mathew C, Spooner E, Mpendo J, Mukwekwerere P, Mgodi N, Ntege PN, Nair G, Nakabiito C, Nuwagaba-Biribonwoha H, Panchia R, Singh N, Siziba B, Farrior J, Rose S, Anderson PL, Eshleman SH, Marzinke MA, Hendrix CW, Beigel-Orme S, Hosek S, Tolley E, Sista N, Adeyeye A, Rooney JF, Rinehart A, Spreen WR, Smith K, Hanscom B, Cohen MS, Hosseinipour MC; **HPTN 084 study group. Cabotegravir for the prevention of HIV-1 in women: results from HPTN 084, a phase 3, randomised clinical trial.** *Lancet*. 2022 May 7;399(10337):1779-1789. PMID: 35378077.

2. Landovitz RJ, Li S, Eron JJ Jr, Grinsztejn B, Dawood H, Liu AY, Magnus M, Hosseinipour MC, Panchia R, Cottle L, Chau G, Richardson P, Marzinke MA, Eshleman SH, Kofron R, Adeyeye A, Burns D, Rinehart AR, Margolis D, Cohen MS, McCauley M, Hendrix CW. **Tail-phase safety, tolerability, and pharmacokinetics of long-acting injectable cabotegravir in HIV-uninfected adults: a secondary analysis of the HPTN 077 trial.** Lancet HIV. 2020 Jul;7(7):e472-e481. doi: 10.1016/S2352-3018(20)30106-5. Epub 2020 Jun 1. PMID: 32497491.

3. Landovitz RJ, Donnell D, Clement ME, Hanscom B, Cottle L, Coelho L, Cabello R, Chariyalertsak S, Dunne EF, Frank I, Gallardo-Cartagena JA, Gaur AH, Gonzales P, Tran HV, Hinojosa JC, Kallas EG, Kelley CF, Losso MH, Madruga JV, Middelkoop K, Phanuphak N, Santos B, Sued O, Valencia Huamaní J, Overton ET, Swaminathan S, Del Rio C, Gulick RM, Richardson P, Sullivan P, Piwowar-Manning E, Marzinke M, Hendrix C, Li M, Wang Z, Marrazzo J, Daar E, Asmelash A, Brown TT, Anderson P, Eshleman SH, Bryan M, Blanchette C, Lucas J, Psaros C, Safren S, Sugarman J, Scott H, Eron JJ, Fields SD, Sista ND, Gomez-Feliciano K, Jennings A, Kofron RM, Holtz TH, Shin K, Rooney JF, Smith KY, Spreen W, Margolis D, Rinehart A, Adeyeye A, Cohen MS, McCauley M, Grinsztejn B; HPTN 083 Study Team. **Cabotegravir for HIV Prevention in Cisgender Men and Transgender Women.** *N Engl J Med.* 2021 Aug 12;385(7):595-608. PMID: 34379922.

4. Eshleman SH, Fogel JM, Piwowar-Manning E, Chau G, Cummings V, Agyei Y, Richardson P, Sullivan P, Haines CD, Bushman LR, Petropoulos C, Persaud D, Kofron R, Hendrix CW, Anderson PL, Farrior J, Mellors J, Adeyeye A, Rinehart A, St Clair M, Ford S, Rooney JF, Mathew CA, Hunidzarira P, Spooner E, Mpendo J, Nair G, Cohen MS, Hughes JP, Hosseinipour M, Hanscom B, Delany-Moretlwe S, Marzinke MA. **Characterization of Human Immunodeficiency Virus (HIV) Infections in Women Who Received Injectable Cabotegravir or Tenofovir Disoproxil Fumarate/Emtricitabine for HIV Prevention: HPTN 084.** *J Infect Dis.* 2022 Mar 18:jiab576. Epub ahead of print. PMID: 35301540.

5. Marzinke MA, Grinsztejn B, Fogel JM, Piwowar-Manning E, Li M, Weng L, et al. **Characterization of HIV infection in cisgender men and transgender women who have sex with men receiving injectable cabotegravir for HIV prevention: HPTN 083.** *J Infect Dis.* 2021 Nov 16;224(9):1581-1592. PMID: **33740057.**

6. Markowitz M, Frank I, Grant RM, Mayer KH, Elion R, Goldstein D, et al**. Safety and tolerability of long-acting cabotegravir injections in HIV-uninfected men (ECLAIR):**

a multicentre, double-blind, randomised, placebo-controlled, Bhase 2a trial. *Lancet HIV.* 2017 Aug;4(8):e331-e40. PMID: 28546090.

7. Eshleman S, Fogel JM, Halvas EK, Mellors JW, Piwowar-Manning EM, Rinehart AR, et al. **CAB-LA PrEP: Early Detection of HIV Infection May Reduce In STI Resistance Risk.** *Virtual Abstract presented at Retroviruses and Opportunistic Infections Confernce, February 12-16, 2022.*

8. Delany-Moretlwe S, Hughes J, Guo X, Hanscom B, Hendrix CW, Farrior J, et al. **Evaluation of CAB-LA Safety and PK in Pregnant Women in the Blinded Phase of HPTN 084. Abstract 700.** *Virtual Conference presented at Retroviruses and Opportunistic Infections Confernce, February 12-16, 2022.*

9. Blair CS, Li S, Chau G, Cottle L, Richardson P, Marzinke MA, Eshleman SH, Adeyeye A, Rinehart AR, Margolis D, McCauley M, Hendrix CW, Landovitz RJ; **HPTN 077 Study Team. Brief Report: Hormonal Contraception Use and Cabotegravir Pharmacokinetics in HIV-Uninfected Women Enrolled in HPTN 077***. J Acquir Immune Defic Syndr.* 2020 Sep 1;85(1):93-97. PMID: 32452972.
10. National AIDS and STI Control Programme, Kenya Medical Research Institute. **Guidelines for Conducting Adolescent HIV Sexual and Reproductive Health Research in Kenya;** 2015.

11. Uganda National Council for Science and Technology. **National Guidelines for Research involving Humans as Research Participants;** 2014.

12. Delany-Moretlwe S, Hughes JP, Bock P, Dadabhai S, Hunidzarira P, Innes S, Kalonji D, Makhema J, Mandima P, Mathew C, Mpendo J, Mukwekwerere P, Mgodi N, Nahira Ntege P, Nakabiito C, Nuwagaba-Biribonwoha H, Panchia R, Angria F, Singh N, Siziba B, Spooner E, Farrior J, Rose S, Berhanu R, Agyei Y, Eshleman SH, Marzinke MA, Piowar-Manning E, Beigel-Orme S, Hosek S, Adeyeye A, Rooney J, Rinehart A, Hanscom B, Cohen MS, Hosseinipour MC. **Long Acting Injectable Cabotegravir: Updated Efficacy and Safety Results from HPTN 084.** AIDS 2022 July 29 - Aug 2; 24th Intl AIDS Conference; 2022. Accessed August 1, 2022. https://www.natap.org/2022/IAC/IAC_22.htm

L. List of Investigators

Diane Havlir, MD
Chair, University of California, San Francisco
Address: UCSF Box 0874, San Francisco, CA 94143-0874, U.S.A.
Phone Number: +1-415-476-4082 ext. 424
Email: diane.havlir@ucsf.edu

Moses R. Kamya, MBChB, MMed, PhD
Co-Chair, Makerere University
Address: Department of Medicine, P.O. Box 7072, Kampala, Uganda
Phone Number: +256-414-533200
Email: mkamya@infocom.co.ug

Maya L. Petersen, MD, PhD
Co-Chair, University of California, Berkeley
Address: School of Public Health, 5402 Berkeley Way West, Berkeley, CA 94720-7358, U.S.A.
Phone Number: +1-510-642-0563

Gabriel Chamie, MD, MPH
Vice-Chair, University of California, San Francisco
Address: UCSF Box 0874, San Francisco, CA 94143-0874, U.S.A.
Phone Number: +1-415-476-4082 ext. 445
Email: gabriel.chamie@ucsf.edu

Laura Balzer, PhD
Investigator and Protocol Statistician, University of California, Berkeley
Address: School of Public Health, 5317 Berkeley Way West, Berkeley, CA 94720-7358, U.S.A.
Phone Number: +1-203-558-3804
Email: laura.balzer@berkeley.edu

Andrew Phillips, MSc, PhD
Investigator, Modeling, University College London, London
Address: Institute for Global Health, Glower Street, WC1 6BT, U.K.
Phone Number: +20-7830-2886
Email: andrew.phillips@ucl.ac.uk

Florence Mwangwa, MBChB, MPH
Investigator, Adult and Adolescents Studies, Infectious Diseases Research Collaboration
Address: P.O. Box 4745, Kampala, Uganda
Phone Number: +256-779-386616
Email: fmwangwa@idrc-uganda.org

Jane Kabami, MPH
Investigator, Infectious Diseases Research Collaboration

Address: P.O. Box 7475, Kampala, Uganda
Phone Number: +256-706-315810
Email: jkabami@idrc-uganda.org

Elijah Kakande, MBChB
Investigator, Infectious Diseases Research Collaboration
Address: P.O. Box 4745, Kampala, Uganda
Phone Number: +256-779-386616
Email: rkakande@idrc-uganda.org

Asiphas Owaraganise, MBChB
Investigator, Infectious Diseases Research Collaboration
Address: P.O. Box 4745, Kampala, Uganda
Email: asiphas@gmail.com

Elizabeth Bukusi, MBChB, M.Med, MPH, PhD
Investigator, Youth and Integration of HIV and Reproductive Health Services, Kenya Medical Research Institute
Address: Box 54840, Nairobi, Kenya
Phone Number: +254-733-617503
Email: ebukusi@rctp.or.ke

James Ayieko, MBChB, MPH, PhD
Investigator, Adult and Adolescent Studies, Kenya Medical Research Institute
Address: P.O. Box 517-20107, Njoro, Kenya
Phone Number: +254-720-925262
Email: jimayieko@gmail.com

Norton Sang, MA
Investigator, Kenya Medical Research Institute
Address: P.O. Box 35-4132, Mega City, Kisumu, Kenya
Phone Number: +254-715-778930
Email: nortonsang@gmail.com

Marilyn Nyabuti, MBChB
Investigator, Kenya Medical Research Institute and the University of Washington, Seattle
Address: P.O. Box 35-4132, Mega City, Kisumu, Kenya
Phone Number: +254-715-778930
Email: mnyanduko@gmail.com

Judith Hahn, PhD, MA
Investigator, Alcohol Use Studies, University of California, San Francisco
Address: UCSF Box 1224, San Francisco, CA 94158
Phone: +1-415-476-5815
Email: judy.hahn@ucsf.edu

Carol Camlin, PhD, MPH
Investigator, Social Sciences/Qualitative Studies, University of California, San Francisco
Address: Center for AIDS Prevention Studies, San Francisco, CA 94143, U.S.A.
Phone Number: +1-415-597-4998
Email: carol.camlin@ucsf.edu

Matt Hickey, MD
Investigator, Hypertension Studies, University of California, San Francisco
Address: UCSF Box 0874, San Francisco, CA 94143-0874, U.S.A.
Phone Number: +1-628-206-8494
Email: matt.hickey@ucsf.edu

Starley Shade, PhD
Investigator, Cost Effectiveness, University of California, San Francisco
Address: Box 0886, San Francisco, CA 94143-0886, U.S.A.
Phone Number: +1-415-476-6362
Email: starley.shade@ucsf.edu

Harsha Thirumurthy, PhD
Investigator, Behavioral Economics Studies, University of Pennsylvania
Address: Department of Medical Ethics and Health Policy, Philadelphia, PA 19104
Phone Number: +1-215-746-0410
Email: hthirumu@upenn.edu

Edwin Charlebois, PhD, MPH
Investigator, University of California, San Francisco
Address: UCSF Box 0874, San Francisco, CA 94143-0874, U.S.A
Phone Number: +1-415-476-6241
Email: edwin.charlebois@ucsf.edu

Priscilla Hsue
Investigator, Cardiovascular Studies, University of California, San Francisco
Address: UCSF Box 0846, San Francisco, CA 94110
Phone: +1+415-206-8257
Email: Priscilla.hsue@ucsf.edu

Theodore Ruel, MD
Investigator, HIV Adolescent and Young Adult Studies, University of California, San Francisco
Address: UCSF Box 0434, San Francisco, CA 94143-0136, U.S.A.
Phone Number: +1-415-476-9197
Email: theodore.ruel@ucsf.edu

Craig Cohen, MD, MPH
Investigator, University of California, San Francisco
Address: Reproductive Sciences, Box 1224., San Francisco, CA 94158 - youth and integration of HIV and reproductive health services
Phone Number: +1-415-476-5874
Email: craig.cohen@ucsf.edu

Eric Goosby, MD
Investigator, University of California, San Francisco
Address: UCSF Box 0874, San Francisco, CA 94143-0874, U.S.A.
Phone Number: +1-415-476-5483
Email: eric.goosby@ucsf.edu

Catherine Koss, MD

Investigator, University of California, San Francisco
Address: UCSF Box 0874, San Francisco, CA 94143-0874, U.S.A.
Phone Number: +1-415-476-4082
Email: catherine.koss@ucsf.edu

Urvi M Parikh, PhD
Virologist, University of Pittsburgh, Pittsburgh
Address: S830 Scaife Hall, 3550 Terrace Street, Pittsburgh, PA 15261
Phone Number: 412-648-3103
Email: ump3@pitt.edu

Melanie Bacon, RN, MPH
Medical Officer, National Institute of Allergy and Infectious Diseases, NIH
Address: 5601 Fishers Lane 9G74, Bethesda, MD 20892, U.S.A.
Phone Number: +1-240-627-3215
Email: mbacon@niaid.nih.gov

Bishop Opira, Bpharm, MPH
Pharmacist, Infectious Diseases Research Collaboration, Kampala, Uganda
Address: Plot 2C Nakasero Hill Road,P.O. Box 7475 Kampala, Uganda
Phone: +256 (0)312 281 479
Email: opibish@idrc-uganda.org

Greshon Rota, Bpharm
Pharmacist, Kenya Medical Research Institute
Address:P.O. Box 54840 00200 Off Raila Odinga Way. Nairobi, Kenya.
Phone: +254 722205901
Email: director@kemri.go.ke

Alex Rinehart, PhD
Medicine Development Leader, Prevention, ViiV Healthcare
Address: Five Moore Drive, Research Triangle Park, North Carolina USA
Phone: 919-483-2085
Email: alex.r.rinehart@viivhealthcare.com

Maggie Czarnogorski
Head, Innovation and Implementation Science, ViiV Healthcare
Address: 1012 14th St Nw, Suite 700, Washington, District Of Columbia
Phone: 202-638-0750
Email: maggie.x.czarnogorski@viivhealthcare.com
