# Supplementary material for: Community health worker–facilitated telehealth for moderate–severe hypertension care in Kenya and Uganda: A randomized controlled trial
Source: PLoS Med. 2025 Jun 5;22(6):e1004632. doi: 10.1371/journal.pmed.1004632 (PMC12165344; doi:10.1371/journal.pmed.1004632)
Supplement: Attachment — Submitted filename: response to reviewers v8.docx [file pmed.1004632.s011.docx]

We thank the editors and reviewers for their thoughtful consideration of our manuscript. Please find our responses in blue font below each comment.

-----------------------------------------------------------
**Comments from the academic editor:**

Overall, the trial seems quite well done. However, there are some aspects that seem a little unclear.

It is not 100% clear why the trial is described as a pilot trial when it is fully powered for blood pressure outcomes. How do the authors think about why this is a pilot trial and what is the scope of the subsequent, presumably larger, trial?

Thank you for the positive feedback and for this question. We consider this a pilot study due to the small size and focus on effectiveness at the individual patient level, rather than broader scale up and evaluation of implementation at a population level. Our subsequent larger trial that was informed by this pilot and is currently ongoing includes population-level hypertension screening of ~30,000 adults aged ≥40 across 16 communities in Kenya and Uganda with choice hypertension care through CHW-facilitated telehealth or clinic-based care for those identified with hypertension. We have added further clarification to these points in the limitations section of the discussion:

New text (Discussion, paragraph 7 – Limitations, new text in italics):

*First, though fully powered for our primary outcome, this was a pilot study with a relatively small sample size which limits inferences about effectiveness at scale.* However, by conducting the study at three government primary care clinics that are broadly representative of primary health centers in rural Kenya and Uganda, our findings are likely generalizable to similar rural primary health care settings. We are currently conducting a follow-up population-level study to understand intervention effects at scale and when integrated with population-level screening for HIV and HIV risk (NCT05768763).

One of the biggest areas of lack of clarity is the precise specification of what constitutes the intervention and what constitutes the control arm. A table or figure might help here. It would be useful to know how people in both arms of the trial were treated - whether they were treated as part of the routine government health care system, or whether they were treated by trial clinicians funded by the project, and how often they were seen. It would also be helpful to know whether the medications were paid for by the patients, the health system or the research team, and whether this differed between arms. One overarching request is that the authors should be much more specific about the implementation strategy they believe they are using in this trial. It seems that there is much more than just a telehealth intervention; there is medication delivery, home care, task sharing with community health workers, and a number of other elements that together could form a package that includes telehealth. More contextual information about the health system would be helpful. But as written, it is a bit difficult for the reader to understand exactly what was done and who paid for it. In implementation science jargon, the implementation strategies are underspecified (Proctor EK, et al. Implement Sci 2013;8:139).

Thank you for this important point. We have added a new table to the supplemental materials to explicitly describe all implementation strategies according to the Proctor framework.

*New Text (Methods, Implementation strategies):*

*The Telehealth intervention included multiple implementation strategies on top of shared strategies for both arms. Telehealth strategies included CHW home visits for blood pressure measurement and medication delivery based on clinician prescription, clinician telehealth consultation that was facilitated by the CHW, CHW training, and CHW stipend payment to support time delivering the intervention. Shared strategies in both telehealth and clinic-based arms included baseline CHW home-based hypertension screening of all adults in the community, transport reimbursement to link to the clinic for the initial visit, free hypertension medications, implementation of a country guideline-based standardized hypertension treatment algorithm, use of an electronic health record for hypertension visits, and case-based provider training on universal positive regard and friendly services. We provide a detailed list of strategies in S1 Supplement Table A.*

## Table A. Implementation strategies employed for Telehealth and Clinic-based hypertension care

| **Strategy^*^** | **Description** | **Telehealth arm** | **Clinic arm** |
| --- | --- | --- | --- |
| Provide community-based services | CHW home visits for BP measurement, and delivery of hypertension medications based on clinician prescription; visits every 4-12 weeks | X |  |
| Provide telemedicine consultation; Use technology to provide real-time support; Alter/improve communication between providers | During each home visit, CHWs facilitate telemedicine consultation with clinician based in primary health center | X |  |
| Provide health education | CHW counseling on lifestyle modification and hypertension medication adherence at each visit | X |  |
| Change payment methods for health workers | Stipend payment to CHWs to compensate for work delivering hypertension intervention^†^ | X |  |
| Provide community-based services | CHW home-based hypertension screening at baseline | X | X |
| Provide transportation | One-time transport reimbursement to patients to facilitate linkage from community screening to primary care clinic for initial clinic visit | X | X |
| Adjust patient/consumer fees | Hypertension medications provided free of charge to patients at each visit | X | X |
| Change physical structure and equipment | BP cuff provision in clinics and for CHWs one time at study baseline | X | X |
| Conduct ongoing training | Training of CHWs and clinicians on hypertension at study baseline and 6-month refresher training | X | X |
| Provide training on patient-centered communication | Clinician and CHW training on patient-centeredness, friendly care at baseline | X | X |
| Increase the quantity of medication dispensed | Dispensation of 3-month medication supply at visits when BP controlled | X | X |
| Institute or improve procurement and storage systems | Enhancement of hypertension medication supply chain to avoid stock-outs throughout study | X | X |
| Introduce/alter health information systems | Use of electronic health record for hypertension visits, allowing review of longitudinal clinical data by clinicians at each visit | X | X |
| Provide guidance document | Standardized country-guideline based hypertension treatment algorithm to simplify treatment decisions for clinicians | X | X |
| Provide multiple types of services by one provider | Provision of both hypertension and diabetes care during same visit | X | X |
| Provide multiple types of services by one provider | Provision of both hypertension and HIV care during same visit |  | X |

* Implementation strategies used for the two trial arms, based on the Expert Recommendations for Implementing Change (ERIC)^1^ and Effective Practice and Organization of Care (EPOC)^2^ implementation strategy compilations, as further refined in a recent review by Lujintanon et al.^3^

† Approximately $40 US dollars per month in 2022-2023. Stipend amount is same as that adopted by the Kenya Ministry of Health for Community Health Promoters beginning January 2024 (after completion of trial).

There are also some important missing reporting items: medication adherence or pill count, intensity of intervention vs. control in terms of visits or some indication of how many contacts patients had, and exact medication usage in each arm, including average dose. Was adherence assessed? It would be good to see the mean blood pressure in each arm reported in Table 1 (perhaps some of this data was not collected).

**Medication adherence**: this was assessed by self-report at each clinical visit (assessed by CHWs in the telehealth intervention arm and by clinicians in the clinic-based arm). We have added two new tables describing adherence data to the supplement (Tables H and I).

**Intensity of intervention vs control**: visits were scheduled with the same frequency in intervention and control - every 4 weeks if blood pressure was uncontrolled and every 12 weeks if controlled (see Methods: Study enrollment and hypertension care delivery). Telehealth intervention participants had more visits due to increased retention in care in the intervention arm. To further understand telehealth intervention effects on visit adherence, we have added a composite measure of the total proportion of follow-up time that participants were adherent to clinic visit schedules: time in care. This measure approximates the time that participants were in possession of hypertension medications. We have now added this outcome to the manuscript text and to Table 2.

**Medication usage in each arm**: the treatment algorithm used was the same in both telehealth intervention and clinic-based control arms. We have added new tables to the supplement to show the number and type of medications prescribed in both arms (Table F) along with the specific medication and dosing combinations (Table G).

**Mean blood pressure in each arm**: we have added mean blood pressure in each arm at baseline to Table 1 as requested.

New text (Results, Primary and secondary outcomes):

*In ad hoc effectiveness analyses, time in care over 48 weeks was 89% in the telehealth arm and 63% in the clinic arm (difference 26%, 95% CI 19,34%).*

New text (Results, Hypertension care delivery):

*Patient-reported medication adherence assessed during clinical visits was high in both arms (S1 Supplement Tables I and F).*

## Supplemental Table H. Hypertension medication adherence by participant

Within-participant mean self-reported adherence across all visits, among participants with at least one post-baseline visit and prescribed ≥1 hypertension medication. Adherence assessed at each visit by asking the number of days hypertension medications were taken over the prior 7 days.

|  | **Intervention (N=92)** | **Control (N=85)** | **Overall (N=177)** |
| --- | --- | --- | --- |
| **Number of days meds taken over past week** |  |  |  |
| Mean (SD) | 5.7 (1.7) | 5.9 (1.9) | 5.8 (1.8) |
| Median (Q1, Q3) | 6 (5, 7) | 7 (6, 7) | 7 (5, 7) |
| Missing adherence assessment for all visits | 2 (2.2%) | 30 (35.3%) | 32 (18.1%) |

## Supplemental Table I. Hypertension medication adherence across all post-baseline visits

Across all post-baseline visits where participant had previously been prescribed ≥1 hypertension medication. Adherence assessed at each visit by asking the number of days hypertension medications were taken over the prior 7 days.

|  | **Intervention (N=417)** | **Control (N=322)** | **Overall (N=739)** |
| --- | --- | --- | --- |
| **Number of days meds taken over past week** |  |  |  |
| Mean (SD) | 5.6 (2.2) | 6.1 (1.9) | 5.8 (2.1) |
| Median [Min, Max] | 7 (4, 7) | 7 (6, 7) | 7 (5, 7) |
| Missing adherence assessment at current visit | 128 (30.7%) | 141 (43.8%) | 269 (36.4%) |

## Supplemental Table F. Number and type of hypertension medications prescribed over 48 weeks*

|  | **Intervention (N=98)** | **Control (N=102)** | **Overall (N=200)** |
| --- | --- | --- | --- |
| **Number of medications*** |  |  |  |
| Mean (SD) | 1.6 (0.8) | 1.7 (0.7) | 1.6 (0.7) |
| Median (Q1, Q3) | 2 (1, 2) | 2 (1, 2) | 2 (1, 2) |
| **Number of medications** |  |  |  |
| 0 | 9 (9.2%) | 7 (6.9%) | 16 (8.0%) |
| 1 | 30 (30.6%) | 25 (24.5%) | 55 (27.5%) |
| 2 | 51 (52.0%) | 63 (61.8%) | 114 (57.0%) |
| 3 | 8 (8.2%) | 7 (6.9%) | 15 (7.5%) |
| **Calcium channel blocker†** | 73 (74.5%) | 79 (77.5%) | 152 (76.0%) |
| **Thiazide diuretic†** | 30 (30.6%) | 31 (30.4%) | 61 (30.5%) |
| **ACE/ARB†** | 18 (18.4%) | 17 (16.7%) | 35 (17.5%) |

* maximum number of drugs prescribed at any clinical hypertension visit (either telehealth or in-clinic)

† proportion prescribed each drug class at any given visit over 48-week study

## Supplemental Table G. Medication combinations prescribed at last attended visit

| **Study Arm** | **Hypertension medications (total daily dose in milligrams)*** | **n** | **%** |
| --- | --- | --- | --- |
| Control | Lifestyle counseling (no medications) | 13 | 12.7% |
|  | Nifedipine: 40 | 7 | 6.9% |
|  | Nifedipine: 20 | 6 | 5.9% |
|  | Bendroflumethiazide: 5 | 3 | 2.9% |
|  | Hydrochlorothiazide: 25 | 3 | 2.9% |
|  | Nifedipine: 40; Hydrochlorothiazide: 25 | 25 | 24.5% |
|  | Nifedipine: 20; Bendroflumethiazide: 5 | 12 | 11.8% |
|  | Nifedipine: 40; Bendroflumethiazide: 5 | 12 | 11.8% |
|  | Amlodipine: 10; Losartan: 50 | 3 | 2.9% |
|  | Amlodipine: 5; Losartan: 50 | 2 | 2.0% |
|  | Bendroflumethiazide: 5; Captopril: 50 | 2 | 2.0% |
|  | Nifedipine: 80; Hydrochlorothiazide: 25 | 2 | 2.0% |
|  | Bendroflumethiazide: 10; Captopril: 50 | 1 | 1.0% |
|  | Bendroflumethiazide: 5; Captopril: 25 | 1 | 1.0% |
|  | Nifedipine: 40; Bendroflumethiazide: 10 | 1 | 1.0% |
|  | Nifedipine: 40; Captopril: 50 | 1 | 1.0% |
|  | Nifedipine: 80; Hydrochlorothiazide: 50 | 1 | 1.0% |
|  | Nifedipine: 40; Bendroflumethiazide: 5; Captopril: 25 | 3 | 2.9% |
|  | Nifedipine: 40; Bendroflumethiazide: 5; Captopril: 50 | 2 | 2.0% |
|  | Amlodipine: 5; Bendroflumethiazide: 5; Losartan: 50 | 1 | 1.0% |
|  | Nifedipine: 20; Bendroflumethiazide: 5; Captopril: 25 | 1 | 1.0% |
| Intervention | Lifestyle counseling (no medications) | 10 | 10.2% |
|  | Nifedipine: 40 | 14 | 14.3% |
|  | Nifedipine: 20 | 7 | 7.1% |
|  | Bendroflumethiazide: 5 | 4 | 4.1% |
|  | Hydrochlorothiazide: 25 | 4 | 4.1% |
|  | Nifedipine: 40; Hydrochlorothiazide: 25 | 23 | 23.5% |
|  | Nifedipine: 20; Bendroflumethiazide: 5 | 14 | 14.3% |
|  | Bendroflumethiazide: 5; Captopril: 25 | 7 | 7.1% |
|  | Nifedipine: 20; Captopril: 25 | 2 | 2.0% |
|  | Amlodipine: 10; Hydrochlorothiazide: 80 | 1 | 1.0% |
|  | Amlodipine: 5; Losartan: 50 | 1 | 1.0% |
|  | Nifedipine: 20; Losartan: 50 | 1 | 1.0% |
|  | Nifedipine: 40; Bendroflumethiazide: 5 | 1 | 1.0% |
|  | Nifedipine: 40; Hydrochlorothiazide: 60 | 1 | 1.0% |
|  | Nifedipine: 40; Hydrochlorothiazide: 90 | 1 | 1.0% |
|  | Nifedipine: 20; Bendroflumethiazide: 5; Captopril: 25 | 7 | 7.1% |

* prescribed at last attended clinical hypertension visit (either telehealth or in-clinic) prior to week 48 study visit

One striking aspect missing from the discussion is any comment or speculation on the mechanism for the trial's putative success. In particular, the top-line results show a very large effect size, but the number of drug prescriptions was essentially the same in each arm. So the mechanism for this extremely large effect size is confusing. Was it a result of access to medication after prescription, or was it an adherence problem where patients who had access to medication did not take it? It seems unlikely that lifestyle counselling alone could account for such a dramatic difference in blood pressure control.

Thanks for these questions and for the opportunity to clarify. We postulate that the primary mechanism of effect is that the CHW telehealth intervention facilitated more consistent attendance of hypertension visits (through telehealth), leading to higher retention in care, more consistent medication possession, and thereby more sustained adherence to antihypertensive medications. Medications prescribed were the same, but sustained adherence was higher in the telehealth intervention arm – as shown by our retention and time-in-care outcomes. We comment on potential mechanisms for intervention success in paragraph two of the discussion and have now expanded this discussion.

Revised text (Discussion, paragraph 2):

*We postulate that the primary mechanism through which the telehealth intervention led to improved outcomes was through improved retention in care. In particular, the CHW-facilitated telehealth intervention reduced transportation barriers to care engagement, resulting in a greater proportion of time patients attended follow-up visits on time and were in possession of and adherent to hypertension medications.* Nearly all participants reported that transportation to clinic was a challenge to consistent care engagement, due to a combination of transportation costs, time, and mobility challenges. Additionally, the CHW-facilitated telehealth model of care resulted in fewer participants reporting concerns about taking medications for an asymptomatic condition or disbelief that they truly had hypertension, highlighting the value added by offering home visits with supportive adherence counseling by a local community member (CHW) in addition to counseling by a clinician.

Authors should make sure that everything reported in the abstract is also reported in the results of the manuscript. For example, the number of antihypertensive drugs prescribed at 48 weeks is reported in the abstract, but not in the manuscript.

Thank you. We have removed the number of antihypertensive drugs prescribed at 48 weeks from the abstract.

It is very difficult to follow the protocol and the clinicaltrials.gov page because this is one of many trials that are all registered and protocolised together. It seems that it would be very useful for readers if the protocol could be consolidated to be for this trial only. Clarity and readability is really important and this would make it easier.

Thank you for this suggestion. We have added a new supplemental file that provides a summary of the trial protocol elements for the trial reported in this manuscript (S2 Protocol Summary).

-----------------------------------------------------------
**Comments from the editorial team:** 

In line with the comments of the academic editor and the reviewers, the editorial team agrees that there is room for more clarity and detail throughout the manuscript. We also agree that, in addition to providing the full original trial protocol, it would be helpful to provide an additional supplementary file that summarises the protocol information specifically for this trial.

Thank you for this suggestion. We have added a new supplemental file that provides a summary of the trial protocol elements for the trial reported in this manuscript (S2 Protocol Summary).

-----------------------------------------------------------
Comments from the reviewers: 

**Reviewer #1:** Thank you for the opportunity to review this randomized clinical trial evaluating the effects of a HCW facilitated and clinician led telehealth intervention on blood pressure control in rural Kenya and Uganda. The report was interesting, and I appreciated the inclusion of patient feedback through end line qualitative surveys. I had some comments in reading, which are itemized below. 
Introduction
1.      Please add context about how hypertension is treated in this setting. 'Clinic' covers a wide variety of healthcare settings - in this context, are these community-based primary health care centers, regional clinics, or tertiary care centers? How are the facilities staffed, and how would a patient with hypertension typically be diagnosed and treated? Are primary care physicians always on site at the clinic?

This study was conducted in rural settings in Kenya and Uganda at level 4 government health centers provide primary care and basic inpatient and surgical services. Typical staffing includes one physician who provides administrative oversight and supervises inpatient and surgical care and several clinical officers (advanced practice providers) and nurses who deliver general primary care, maternal/child health, and HIV care. These rural facilities are located within the communities they serve, though patients often need to walk an hour or more to reach the health facility. Hypertension treatment can be delivered in these health facilities, though blood pressure cuffs and hypertension medications are often lacking, and general outpatient clinics are not well set up to deliver longitudinal care for chronic conditions. Our clinic-based (control) arm We have revised the introduction to provide this information and added details about the health center to the methods.

New text (Introduction, paragraph 2):

*In rural Kenya and Uganda, hypertension care is predominantly available in specialty clinics within regional referral hospitals, but accessing these specialty clinics poses a significant challenge due to long travel distances and extended wait times.^7^ Although multiple studies have demonstrated successful delivery of hypertension treatment in community-based primary care clinics by nurses or advanced practice providers,^8–10^ these clinics often face barriers such as lack of functional blood pressure cuffs and antihypertensive medications.^7^ Additionally, primary care clinics in this setting typically focus on episodic care for acute conditions and lack the infrastructure needed for robust, ongoing management of chronic conditions such as hypertension.*

New text (Methods: Setting and Participants*)*

*Each community had a government-run level 4 health center, which was the closet facility where community members could access primary health services. Level 4 health facilities typically provide primary care and basic inpatient and surgical services and are staffed by one physician and several clinical officers (advanced practice providers) and nurses.*

2.      There are others studies in low-income settings which have evaluated CHW-led hypertension care within a home/community setting. The closing arguments in this section could be expanded to include other examples of the positive effects of CHW-led hypertension care - see Joshi et al, 2019, American Heart Journal; Jafar et al, 2020 The New England Journal of Medicine; Khetan et al, 2019 Global Heart journal; O'Neil et al, 2016, Health Policy and Planning.

We agree that these are important studies demonstrating the potential benefits of enhancing CHW services for hypertension and CVD screening, behavioral counseling, adherence support, and linkage to clinical care. We have revised our introduction to include these references.

New Text (Introduction, paragraphs 3-4):

*Two large studies in lower-middle income countries in south Asia (India, Bangladesh, Pakistan, Sri Lanka) demonstrated substantial reductions in blood pressure and improvements in hyperension control with CHW home visits to provide hypertension screening, behavioral counseling, and adherence support alongside robust referrals to clinic-based treatment.^21,22^ Another study in India found that while CHW home visits for adherence support improved medication adherence, counseling alone without medication delivery was not enough to improve hypertension control.^23^*

*Other studies have evaluated expansion of hypertension treatment into the community, without requiring clinic attendance. A study in Uganda showed that in-person visits by a clinician and a CHW in the community improved access to care and reduced blood pressure more effectively than district hospital-based care.^24^ We aimed to build on this prior work to determine whether community-based hypertension treatment, delivered through CHW home visits and supervised by a clinician via telehealth, could improve hypertension control more effectively than primary care clinic-based treatment among adults with moderate-severe hypertension in rural Uganda and Kenya.*

Methods
3.      Can you clarify the exclusion of young adults in this population? i.e. aged 18-39.

We focused our trial on adults aged ≥40 years, rather than all adults aged ≥18 years in our pilot study for several reasons: 1) adults ≥40 years of age are at greater risk of cardiovascular disease and therefore more likely to benefit, 2) higher prevalence of hypertension among older population (higher pre-test probability), therefore elevated blood pressure on initial screen is less likely to be a false positive in this population, 3) focusing on a smaller sub-set of the population for CHW screening and treatment support is more likely to be feasible at scale, and 4) younger individuals with moderate-severe hypertension may be more likely to have secondary hypertension due to an identifiable cause (as opposed to essential hypertension), and therefore may be less appropriate for management in a community-based care model. We have added clarification on this point to the methods section.

4.      Were medications always available? Were they subsidized in any way for participants?

Hypertension medications were consistently available in both intervention and control, with study supplementation in the event of stock-outs. Though patients typically must pay for hypertension medications in Kenya and Uganda, the study provided medications free of charge to trial participants. As a pilot study, our focus was on the effectiveness of the CHW-facilitated telehealth intervention for improving patient-level hypertension outcomes, rather than system level interventions to address medication supply chain interruptions or health policy and health center financing reform to intervene on medication availability or cost.

New text (Methods: Procedures)

*In primary health centers in Kenya and Uganda, hypertension medications are typically available for a small fee (~$1 US dollar per one-month supply). For the study, all medications were provided free of charge to study participants in both telehealth and clinic arms. In the event of medication stock-outs, the study provided additional medication supply to ensure uninterrupted access to medications.*

5.      "Participants without week 24 blood pressure measures were assumed to be uncontrolled."- have you performed a sensitivity analysis here to evaluate the impact of this assumption?

Yes, we conducted several sensitivity analyses to examine the robustness of our missing data assumptions, as detailed in the Statistical Analysis Plan: 1) exclusion of participants with missing outcome (i.e., conducted a complete-case analysis), and 2) use of TMLE to flexibly and efficiently control for missing data. As shown in the Table below, our results were robust to our approach to missing data. We have included these sensitivity analyses and discussion in the Supplementary Table D.

New text (Methods, Statistical Analysis):

*In sensitivity analyses, we varied our approach to missing data and covariate adjustment;*

New text (Results, Primary and Secondary Outcomes):

*Results were robust to handling of missing data and approach to covariate adjustment (S1 Supplement Tables C-D).*

## Supplemental Table D. Results and interpretation of sensitivity analyses varying the approach to missing data

|  | INTERVENTION | CONTROL | EFFECT |
| --- | --- | --- | --- |
| **HTN** **control at 24 weeks** |  |  |  |
| Primary analysis | 77% (69,85%) | 51% (42,60%) | 26% (14,38%); p<0.001 |
| Exclude missing | 78% (70,86%) | 54% (44,63%) | 25% (12,37%); p<0.001 |
| TMLE for missing data | 78% (70,86%) | 53% (43,62%) | 26% (13,38%); p<0.001 |
| **HTN** **control at 48 weeks** |  |  |  |
| Primary analysis | 86% (79,92%) | 44% (34,54%) | 42% (30,53%); p<0.001 |
| Exclude missing | 90% (84,96%) | 49% (39,59%) | 41% (30,53%); p<0.001 |
| TMLE for missing data | 90% (84,96%) | 49% (39,59%) | 41% (30,53%); p<0.001 |

The primary analysis conservatively assumed that participants without blood pressure measures were uncontrolled. We conducted two sensitivity analyses to examine the robustness of our missing data approach: (1) exclude participants with missing outcomes, and (2) use TMLE to flexibly adjust for participants with and without blood pressure measures. As shown in the above Table, these sensitivity analyses yielded very similar results for effectiveness on hypertension control at week 24 (primary endpoint) and at week 48 (secondary endpoint).

Results
6.      "and were referred to the nearest government primary health center for evaluation and treatment. Nine percent of those screened (n=266) had moderate-severe hypertension (average blood pressure ≥160/100 mmHg) and were referred to the nearest government primary health center for clinical evaluation and study enrollment." I think in the second sentence here, you mean that they were referred for study enrollment, assuming they were already at the PHC based on their prior referral for evaluation?

Sorry for the confusion. This statement is correct – nine percent (n=266/2965) of the population screened by CHWs in the community had moderate-severe hypertension potentially qualifying them for study eligibility; these individuals were referred from the community to the primary care clinic in their community clinical evaluation and study enrollment. The sentence prior to this one describes the total population with BP ≥140/90, all of whom were referred to clinical care but only a sub-set of whom were eligible for the study. We have clarified the text as follows.

Revised text (Results, paragraph 1):

*One quarter of those screened had elevated blood pressure (≥140/90 mmHg on average of three measures; 750/2965, 25%). Those with blood pressure ≥140/90 mmHg but <160/100 mmHg (n=484) were referred to the nearest government primary health center for evaluation and treatment but were not eligible for the study. Nine percent of those screened in the community (n=266/2965) had moderate-severe hypertension (average blood pressure ≥160/100 mmHg) and were referred to the nearest government primary health center for both clinical evaluation and screening for study enrollment.*

7.      Of the 750 patients who screened for hypertension in the community, how many presented at the PHC for further evaluation? There seems to be relatively high attrition from the 266 who screened for moderate-severe hypertension and the 214 who linked to clinic - was similar attrition observed at the earlier step?

We unfortunately do not have complete data on linkage to clinic for those with blood pressure 140-159/90-99 mmHg (n=750-266 = 484). These individuals were referred to the general outpatient department of the clinic for treatment by non-study clinicians employed by the Ministry of Health and comprehensive linkage to care data is not available. As above, we have clarified the two populations of individuals with mild vs moderate-severe hypertension in the manuscript text.

8.      "Of those with severe hypertension on initial screening, 214 (80%) linked to clinic and 200 were enrolled in the study (98 intervention, 102 control)." For consistency, I think this should be "moderate-severe hypertension".

Thank you. We have made this correction.

9.      "Mean blood pressure of enrolled participants was 167/100 mmHg at community-based screening and 161/96 mmHg on repeat measurement in the clinic." - these measures need an indicator of variability, i.e. SD.

We have added standard deviations both to the text and to the mean blood pressure values included in Table 1.

Revised text (Results, paragraph 2):

*Mean blood pressure of enrolled participants was 167/100 mmHg (standard deviation [SD] 17/11) at community-based screening and 161/96 mmHg (SD 20/11) on repeat measurement in the clinic.*

10.     Reasons for declining HIV testing - with numbers in each group - are reported twice. One of these segments should be deleted.

Thank you, we have removed the duplicated sentence.

11.     Please clarify in the adverse events that one patient died to due renal failure and one due to heart failure - as written, this is ambiguous.

We have revised this statement to make it clearer that one intervention participant died of renal failure and one died of heart failure. We have retained information that the participant who died of renal failure had baseline stage 5 chronic kidney disease (eGFR of 4) identified at the time of study enrollment.

Revised text (Results: Adverse events)

*Three participants died, two in the intervention arm (one with baseline stage 5 chronic kidney disease who died of renal failure and one who died of heart failure) and one in the control arm (death at home following acute illness).*

Table 1
12.     Where '0' is reported as the frequency, a decimal should not be used in the percent, i.e. report as 0 (0%).

We have made this correction throughout Table 1.

13.     CKD - the numbers in these subcategories do not sum to the top line of 'Any CKD (stage 3+ or urine protein 1+ or greater)'. I understand they may not be mutually exclusive, but the numbers in the control group, for example, sum to 18 (14+2+1+1) which is less than the top line reporting 22. Please clarify with footnotes or correct the numbers.

Thank you for catching this – we had an error in the CKD categories in our table. We have corrected the table. All data have been double-checked and we have made a few additional minor revisions to Table 1.

**Reviewer #2:** This pilot RCT sought to test whether a clinician-driven, CHW facilitated telehealth intervention would improve hypertension control among adults with severe hypertension in rural settings. 
This is a relevant strategy under study, with the potential to inform strategies to improve access to chronic disease care in areas with most need. 

Here are a few observations: 
All participants with an elevated Bp during household screening were referred to the nearest primary health center. What was the linkage rate?

The linkage rate for those with a BP ≥160/100 mmHg identified at community screening was 80% (n=214/266). Those with BP 140-159/90-99 mmHg were referred to the Ministry of Health operated outpatient department at the community primary health center for evaluation and treatment. We did not systematically capture linkage data for this population.

The enrollment clinical and laboratory evaluation was thorough. It is not clear if the management was based on the evaluation findings, eg, management of dyslipidemia, diabetes, CKD, etc among the study participants, given that the focus was on hypertension, yet ethically bound to manage diagnosed co-morbidities.  For the participants in the intervention arm with comorbidities, what was the medicine delivery option for non-hypertension diagnoses?

We relied on routinely available medications within government primary care clinics for treatment of dyslipidemia, diabetes, and CKD. All baseline laboratory values were reviewed by study clinicians and treatment recommended according to country guidelines. Metformin was available and was provided free of charge alongside hypertension medications when indicated. Statins were not available in study clinics, thus clinicians advised patients to purchase statins from outside pharmacies if indicated but we were not able to include statins as part of medication delivery. All patients with advanced CKD received referrals to a nephrologist for specialty evaluation. We have added a new supplemental file S2 Appendix that details the hypertension and diabetes clinical treatment guidelines used in the study.

What was the definition of linkage for the intervention arm?

Participants were randomized after attending an initial in-person clinic visit for evaluation by a clinician and laboratory assessment. Therefore, we do not have linkage assessment disaggregated by trial arm since all enrolled participants, by definition, regardless of study arm, were required to link to care prior to undergoing study enrollment and randomization.

It is also not clear how the medication dose adjustment was done for the participants in the intervention arm given that the prepacked medication was delivered by the CHW, yet a dosing decision should be based on the Bp at the household encounter.

Thank you for this question. We used a simplified treatment algorithm with discrete ‘steps’ defining the standard treatment intensification. Clinicians were free to deviate from this algorithm when clinically indicated, but most participants were on these standard treatment steps. In advance of visits, CHWs came to the clinic to pick up pre-packaged medication sets that included two potential options for each patient, namely 90 day supply of continuing their current treatment regimen (in the event BP was controlled), or 30 day supply of the next ‘step’ in the algorithm. We have added this example to the methods text to provide additional clarification. Treatment algorithm steps are available in S2 Appendix.

Revised text (Methods, Telehealth intervention arm, new text in italics):

Prior to the visit, clinicians packed medications on a weekly basis in anticipation of potential medication combinations that might be needed by upcoming participants, based on anticipated hypertension treatment algorithm steps depending on whether their blood pressure was controlled or not *(e.g. 90-day supply of current medications packaged in the event a participant had controlled blood pressure and a 30-day supply of the next algorithm step in the event blood pressure was uncontrolled and the clinician decided to intensify treatment, with unused medications returned to the clinic after the visit). The clinician also had the option of asking the patient to come to clinic for an in-person visit if clinically indicated.*

There is an important sub-group of enrollees; those who had been previously diagnosed with hypertension (42%) and the 19% who were on medication. How did their outcomes compare with those diagnosed for the first time through the project?  It would also be very informative to analyze the outcomes against the comorbidities.

We agree and have conducted additional subgroup analyses to examine intervention effectiveness by history of hypertension, prior use of hypertension medication, and having co-morbidities. We have now revised Figure 3 to include prior hypertension diagnosis and whether on hypertension medications at baseline and have converted Figure 3 into a panel figure including outcomes at both 24 and 48 weeks. A key finding is that those with a history of hypertension diagnosis and those on medication at baseline had attenuated telehealth intervention effect at week 24 due to improved outcomes in the clinic-based arm for these individuals. At week 48, telehealth intervention effect increased substantially for those with a prior history of hypertension, but remained small and non-significant for those on antihypertensive medications at baseline. This finding likely reflects that those who are already able to navigate clinic systems to start and maintain antihypertensive medications may benefit less from telehealth than those who are undiagnosed or are diagnosed but not on antihypertensive medications.

Revised text (Methods, Primary and secondary outcomes):

For all endpoints, we examined the intervention effect in subgroups of sex, age (<60 versus ≥60 years), country, baseline hypertension severity, *hypertension history (new versus prior diagnosis), baseline use of medication for hypertension, and presence of co-morbidities (HIV, diabetes, chronic kidney disease).*

New text (Results, Primary and secondary outcomes):

*Similar intervention effects on control at 24 and 48 weeks were seen across sub-groups, defined by age, sex, baseline hypertension severity, hypertension history, and presence of co-morbidities (HIV, diabetes, chronic kidney disease); those with baseline hypertension medication use had a smaller telehealth intervention effect, driven by improved outcomes in the clinic-based arm for this group (Fig 3, S1 Supplement Figure B-C).*

**Fig 3. Hypertension control by sub-group**

(A) Hypertension control at 24-week study visit, stratified by subgroup. (B) Hypertension control at 48-week study visit, stratified by subgroup.

## Supplemental Figure B: Hypertension control at 24 weeks by presence of co-morbidities

## Figure C: Hypertension control at 48 weeks by presence of co-morbidities

Amon the HIV clients, clarify if the HIV care remained clinic based even for those in the intervention arm. In the control arm, how can the retention be 53% when they are already coming to the clinics for HIV care, unless the clinics are independent of each other, thus negating the service integration referred to.

Thank you for raising this important point. Though we initially intended to provide HIV medication delivery and telehealth clinician review, HIV clinics required patients to come to clinic for in-person evaluation. Thus, HIV care remained clinic-based for those in the telehealth intervention arm. For the clinic-based control arm, patients received care for both HIV and hypertension when presenting to the clinic, if due for a visit for both conditions. There are several reasons why we think that retention in hypertension care for people with HIV was lower in the clinic-based arm, which we have added to the discussion as follows:

New Text (Discussion, paragraph 5):

*For people with HIV, integration of HIV and hypertension care is an important way to improve cardiovascular disease prevention.^44,45^ However, in our clinic-based arm where HIV and hypertension care were integrated, retention in hypertension care among people with HIV was only 53% at week 48, compared to 100% retention in the telehealth arm. Several factors likely explain low retention in hypertension care among people with HIV in our study. First, moderate-severe hypertension requires a different level of intensive monitoring until stabilized, often monthly visits until blood pressure is controlled. In contrast, HIV visits are often every 3-6 months for stable patients. Second, only 44% (n=12/27) of study participants with HIV and hypertension accessed HIV care at their local community primary care clinic (where the study was based). We did not assess reasons for receiving HIV care at a different clinic outside of the community, though reasons may include stigma, travel/mobility, or other preference-related factors.*

It would be great to have clarity about the payment for medication and the overall supply chain for the intervention. Whether the project meeting the cost of drugs, or the patients were paying, and what happens when the clients had no money, and the role of health insurance if any.

Thank you for this point. Please see our response to Reviewer 1 above for clarification on medication costs and supply chain. In the context where this study was conducted, health insurance for chronic conditions is not readily available. Revised text is copied below for reference.

New text (Methods: Study enrollment and hypertension care delivery):

*In primary health centers in Kenya and Uganda, hypertension medications are typically available for a small fee (~$1 US dollar per one-month supply). For the study, all medications were provided free of charge to study participants in both telehealth and clinic arms. In the event of medication stock-outs, the study provided additional medication supply to ensure uninterrupted access to medications.*

The authors can comment on the utility of a single Bp reading to determine control.

We followed procedures described in the 2020 International Society of Hypertension guidelines indicating that if the first reading is normal, no further measurement is required. Because the first reading tends to be elevated and readings decrease after repetition, a normal first reading is unlikely to be followed by elevated subsequent readings. This practice is also more practical for busy clinical environments and for subsequent scale-up efforts. We have added this clarification to the manuscript text.

Revised text (Methods, Participants):

*Consistent with guidelines and to enhance feasibility of large-scale screening, participants with a normal blood pressure on the first reading did not require subsequent readings.*^5^

In general This is  a well-designed study that generates important information that can inform NCD interventions.

We thank the reviewer for their thoughtful comments and suggestions to improve our manuscript!

**Reviewer #3:** Overall, I found the methodology and reporting clear and robust. It is great to see high retention and follow-up rates at week 24 and week 48. My main concern relates to baseline imbalances in blood pressure and to what extent this was appropriately accounted for in the analysis.

We thank the reviewer for their careful review and have now added adjustment for baseline blood pressure as described in our point-by-point responses below.

Major/general comments:
----------------------------

1.      According to table 1, it looks as if a greater proportion of patients had hypertension falling in the "160-179/100-109 mmHg" category in the control arm (39.2% in control vs 23.5% in intervention). This is a substantial baseline difference. While it may be due to chance, this important imbalance in the outcome of interest has the potential to bias the main outcome analysis in favor of the intervention if not appropriately adjusted for. I note that baseline hypertension grade was a candidate variable in the TLME approach; however, I would be keen to understand whether baseline hypertension grade was adjusted for in the final model and if the results change with and without adjustment. I would also strongly recommend adjusting for baseline blood pressure as a continuous variable in every analysis (forcing the inclusion as a covariate and not letting the TLME model make the selection). As it stands it is not clear to me whether the difference in hypertension control at week 24 and week 48 are simply a "carry-over" from the differences already present at baseline or a true effect of the intervention.

We appreciate the Reviewer’s concern about chance imbalance on baseline blood pressure by arm. At the Reviewer’s suggestion, we have conducted two additional sensitivity analyses: (1) forced adjustment for baseline hypertension grade and (2) forced adjustment for baseline systolic blood pressure as continuous variable. As shown in the below Table, which has been added to the Supplementary Material, our effectiveness analyses are very robust to the approach to covariate adjustment. These results support that the difference in hypertension control at weeks 24 and 48 are true intervention effects.

New text (Methods, Statistical Analysis):

*In sensitivity analyses, we varied our approach to missing data and covariate adjustment;*

New text (Results, paragraph 2):

*Baseline covariates were balanced by arm with the exception of hypertension stage; by chance, a higher proportion of telehealth intervention participants had Grade 1 hypertension (50%) than clinic-based arm participants (37.3%; Table 1).*

New text (Results, Primary and Secondary Outcomes):

*Results were robust to handling of missing data and approach to covariate adjustment (S1 Supplement Tables C-D).*

New text (Discussion, Paragraph 7 - Limitations):

*First, by chance there was imbalance hypertension severity at baseline between arms. However, our primary analysis adjusted for baseline hypertension grade as well as other variables, and our results were robust to the analytic approach and sensitivity analyses (Supplementary Tables C-D).*

## Table C. Results and interpretation of sensitivity analyses varying the approach to covariate adjustment

|  | INTERVENTION | CONTROL | EFFECT |
| --- | --- | --- | --- |
| **HTN** **control at 24 weeks** |  |  |  |
| Primary analysis | 77% (69,85%) | 51% (42,60%) | 26% (14,38%); p<0.001 |
| Unadjusted | 77% (68,85%) | 52% (42,62%) | 25% (12,37%); p<0.001 |
| Adjustment for HTN grade | 77% (69,85%) | 52% (42,61%) | 25% (12,38%); p<0.001 |
| Adjustment for SBP | 76% (68,85%) | 52% (42,62%) | 24% (11,37%); p<0.001 |
| **HTN** **control at 48 weeks** |  |  |  |
| Primary analysis | 86% (79,92%) | 44% (34,54%) | 42% (30,53%); p<0.001 |
| Unadjusted | 86% (79,93%) | 44% (34,54%) | 42% (30,53%); p<0.001 |
| Adjustment for HTN grade | 86% (79,92%) | 44% (34,54%) | 42% (30,53%); p<0.001 |
| Adjustment for SBP | 86% (79,93%) | 45% (35,54%) | 41% (29,53%); p<0.001 |

HTN: Hypertension. SBP: systolic blood pressure

The primary analysis used TMLE with Adaptive Pre-specification to select the adjustment approach maximizing precision, while protecting Type-I error and returning estimates of the marginal effect. (See below for brief overview of TMLE).^4^ For hypertension control at 24 weeks (primary outcome), the approach selected adjustment for all candidates (age, sex, baseline hypertension severity, and country) and was, as expected, more precise than alternative approaches. Specifically, the relative variance of the primary analysis to the unadjusted effect estimator was 89.6%; this would translate into an 11.4% savings in sample size.^4–6^ Also as expected, the results were similar when using adaptive adjustment (primary analysis) and when using fixed adjustment for baseline hypertension grade and for baseline systolic blood pressure (SBP).

Results were also robust to the analytic approach for hypertension control at 48 weeks (secondary outcome). For this secondary endpoint, the primary analytic approach (TMLE with Adaptive Pre-specification) selected adjustment for baseline hypertension grade. Therefore, the results for the primary approach are essentially equivalent to the sensitivity analysis with forced adjustment for baseline hypertension grade.

Here, we provide a brief overview of TMLE. Let *W* denote the baseline covariates, *A* be an indicator of being in the intervention arm, and *Y* be the outcome. With the following steps, TMLE returns estimates of the marginal effect on the absolute scale:

$$\mathbb{E}\left[ \mathbb{E}\left( Y | A=1,W \right) \right]\mathbb{- E[E}\left( Y | A=0,W \right)]$$

1. Estimate the expected outcome given the trial arm and covariates (a.k.a., the outcome regression): $\mathbb{E}\left( Y | A,W \right)$
2. Estimate the known propensity score: $\mathbb{P}\left( A=1 | W \right)$
3. Target initial estimates of the outcome regression using information in the propensity score: ${\hat{\mathbb{E}}}^{*}(Y|A,W)$
4. Obtain targeted predictions of the expected outcome under the intervention and control for all units: ${\hat{\mathbb{E}}}^{*}\left( Y | A=1,W_{i} \right)$ and ${\hat{\mathbb{E}}}^{*}\left( Y | A=0,W_{i} \right)$ for $i=1,\ldots,N.$
5. Obtain a point estimate by averaging the targeted predictions and contrasting on the scale of interest:

$$\frac{1}{N}\sum_{i=1}^{N} {\hat{\mathbb{E}}}^{*}\left( Y | A=1,W_{i} \right)-\frac{1}{N}\sum_{i=1}^{N} {\hat{\mathbb{E}}}^{*}\left( Y | A=0,W_{i} \right)$$

6. Obtain inference with the estimated influence curve (function).

For binary or bounded continuous outcomes, the logistic link function is recommended for the outcome regression and was used in all analyses. Importantly, TMLE is robust to specification of the working models for the outcome regression or propensity score.

In the primary analysis, we implemented TMLE with Adaptive Pre-specification to data-adaptively select the combination of estimators of the outcome regression and propensity score that maximize empirical efficiency, without sacrificing Type-I error control. Further details on the methodology are available in Balzer et al. *Biometrics* 2024.^4^ Further details for this project are provided in the Statistical Analysis Plan.

The selected adjustment approach for each endpoint were as follows.

|  | **Outcome regression** | **Propensity score** |
| --- | --- | --- |
| HTN control at 24 weeks | Main terms* | Age |
| HTN control at 48 weeks | HTN severity | HTN severity |
| Moderate-severe HTN at 24 weeks | NA** | NA** |
| Moderate-severe HTN at 48 weeks | NA** | NA** |
| Retention in care at 24 weeks | Country | HTN severity |
| Retention in care at 48 weeks | Sex | Unadjusted |
| Mean SBP at 24 weeks | Main terms* | Age |
| Mean SBP at 48 weeks | Main terms* | Unadjusted |
| Time in care | Country | Unadjusted |

HTN: Hypertension; SBP: Systolic blood pressures

*Adjusting for all candidates (age, sex, baseline HTN severity, and country) with main terms logistic regression.

**As specified in the Statistical Analysis Plan, we did not use TMLE with Adaptive Pre-specification for the proportion with moderate-severe HTN. Instead, we used an alternative TMLE to flexibly adjust for missing data.

2.      Please provide further information around the approach used to derive absolute differences and 95% confidence intervals. Given TLME was used, I expect these to be adjusted for baseline covariates rather than being raw differences in percentages. However, the modelling approach is not entirely clear to me and it currently looks as if all differences are raw (unadjusted) differences.

We appreciate the Reviewer’s interest in our primary analytic approach. TMLE is a model-robust approach for effect estimation and increases efficiency in randomized trials by adjusting for baseline covariates, while estimating marginal effects (or any other user-specified effect measure). This use of covariate adjustment has a long history, starting with R.A. Fisher in 1932, and has been endorsed by the US Food and Drug Administration as well as the European Medicines Agency.

As demonstrated in the above response, covariate adjustment often yields very similar point estimates, but can meaningfully increase precision. In fact, TMLE with Adaptive Pre-specification is guaranteed to be at least as precise as the unadjusted effect estimator. Specifically, it will avoid forced adjustment at the detriment to precision.

Use of TMLE for this purpose has been covered in several papers and books, including but not limited to:

- Moore KL, van der Laan MJ. Covariate adjustment in randomized trials with binary outcomes: targeted maximum likelihood estimation. *Stat Med*. 2009;28(1):39-64.
- Rosenblum M, van der Laan MJ. Simple, efficient estimators of treatment effects in randomized trials using generalized linear models to leverage baseline variables. *Int J Biostat*. 2010;6(1):Article 13
- van der Laan M, Rose S. *Targeted Learning: Causal Inference for Observational and Experimental Data*. Springer; 2011.
- van der Laan M, Rose S. *Targeted Learning in Data Science*. 1st ed. Springer Cham; 2018.
- Balzer LB, Cai E, Godoy Garraza L, Amaranath P. Adaptive selection of the optimal strategy to improve precision and power in randomized trials. *Biometrics*. 2024;80(1):ujad034. doi:10.1093/biomtc/ujad034

These are referenced in the main text. We provide further details and references in the Statistical Analysis Plan, included in the Supplementary Materials. As shown in the above response, we have also clarified that TMLE is estimating marginal effects and provided a step-by-step overview in the Supplementary Materials.

Minor/specific comments:
----------------------------

1.      Please clarify whether the telehealth intervention was in addition to usual clinical care or used as a replacement? In other words, were participants randomised to the intervention still able to be followed up in the clinic? If so, please consider clarifying this in the manuscript. Please also consider renaming "control" as "usual care" or "clinical care".

Intervention participants were scheduled for home-based follow-up visits by telehealth, though clinicians could recommend a clinic visit if clinically indicated (e.g. if someone had signs/symptoms of hypertensive urgency/emergency, an urgent in-person visit was recommended).

For clinically stable patients, the telehealth visits were used as a replacement for in-person clinic visits. However, the telehealth intervention included the option of scheduling patients for follow-up in clinic if the clinician determined that in-person evaluation was needed. We have revised the manuscript text to make this clearer.

We have also revised the randomized treatment arm names to “telehealth” and “clinic” or “clinic-based” groups throughout for clarity. Our clinic-based comparator group received care that was beyond that available through usual care (care that was integrated to the extent possible, clinicians provided with training on friendly services, triage to expedite visits, free medications), thus we have not elected to name this arm “usual care”.

New text (Results, Hypertension care delivery):

*In the telehealth group, 95% of post-baseline visits were conducted at home by telehealth (485/512) and only 5% of visits were conducted in the clinic due to clinician recommendation (27/512). All post-baseline visits in the clinic-based arm occurred in the clinic (373/373).*

2.      I note that participants and clinicians were not blinded to the group allocation. Please clarify whether assessors were blinded and, if not, whether this may have caused any bias.

Assessors were also not blinded due to logistical challenges in ensuring blinding among study staff. However, the primary outcome was assessed with automated blood pressure cuffs, minimizing potential impact on study outcome ascertainment. We have added this to the methods and limitations.

Revised text (Methods, Randomization and masking, new text in italics):

Participants, clinicians, *and research assistants conducting endpoint assessments* were not blinded to the randomization arm, but the study statistician (LBB) was blinded until trial completion and analysis.

New text (Discussion, limitations paragraph):

*Finally, intervention allocation was not blinded to staff delivering the interventions (CHWs, clinicians) or research assistants assessing outcomes, which could have introduced bias. However, our primary outcome of blood pressure control was assessed using automated blood pressure machines, minimizing the potential for bias in outcome assessment.*

3.      On page 6, it is written that "Regardless of the trial arm, follow-up visits were scheduled every 4 weeks if hypertension was uncontrolled and every 12 weeks if hypertension was controlled". Please clarify whether these follow-up visits were expected to be in-person clinic visits for all participants? Or was it telehealth visits for "intervention" participants and clinic visits for "control" participants?

Visits could be by telehealth or in-person for the telehealth arm, with telehealth being the default unless the clinician determined that an in-person visit was needed. All clinic arm visits were scheduled in the clinic. As described above, 95% of all post-baseline visits were by telehealth in the telehealth intervention arm. As intended, all visits for the clinic-based arm were conducted in the clinic. We have added this to the methods section for clarity.

New text (Methods, Procedures, page 7):

*Visits could be by telehealth or in-person for the telehealth arm, with telehealth being the default unless the clinician determined that an in-person visit was needed. All clinic arm visits were scheduled in the clinic.*

4.      In the outcomes section, it is written that "Participants without week 24 blood pressure measures were assumed to be uncontrolled". This is a fairly strong/conservative assumption about the missing data and I wonder whether alternative imputation strategy should be considered. I would suggest considering a tipping point analysis to check whether the results are consistent under different missing data assumptions (see for example [https://urldefense.com/v3/__https://pubmed.ncbi.nlm.nih.gov/12483769/__;!!LQC6Cpwp!vBXN6i9HZtJyXzbXEZa2BgyovL-jGT5Bs8PzeQ0FfnS3PPWcxvhKYmzp9-LU94s4851gj3VihwrBv10YFpz1$](https://urldefense.com/v3/__https:/pubmed.ncbi.nlm.nih.gov/12483769/__;!!LQC6Cpwp!vBXN6i9HZtJyXzbXEZa2BgyovL-jGT5Bs8PzeQ0FfnS3PPWcxvhKYmzp9-LU94s4851gj3VihwrBv10YFpz1$) ).

We appreciate the Reviewer’s concern that participants with blood pressure measures differed meaningfully from participants without such measures. We conducted several sensitivity analyses to examine the robustness of our missing data assumptions, as detailed in the Statistical Analysis Plan: 1) exclusion of participants with missing outcome (i.e., conducted a complete-case analysis), and 2) use of TMLE to flexibly and efficiently control for missing data. Our results were robust these sensitivity analyses to address missing data. We politely decline the Reviewer’s request to conduct additional tipping point analyses and instead have included the following Supplemental Table and explanation in the Supplementary Materials. Additionally, in the main text, we now clarify:

New text (Methods, Statistical Analysis):

*In sensitivity analyses, we varied our approach to missing data and covariate adjustment;*

New text (Results, Primary and Secondary Outcomes):

*Results were robust to handling of missing data and approach to covariate adjustment (S1 Supplement Tables C-D).*

## Supplemental Table D. Results and interpretation of sensitivity analyses varying the approach to missing data

|  | INTERVENTION | CONTROL | EFFECT |
| --- | --- | --- | --- |
| **HTN** **control at 24 weeks** |  |  |  |
| Primary analysis | 77% (69,85%) | 51% (42,60%) | 26% (14,38%); p<0.001 |
| Exclude missing | 78% (70,86%) | 54% (44,63%) | 25% (12,37%); p<0.001 |
| TMLE for missing data | 78% (70,86%) | 53% (43,62%) | 26% (13,38%); p<0.001 |
| **HTN** **control at 48 weeks** |  |  |  |
| Primary analysis | 86% (79,92%) | 44% (34,54%) | 42% (30,53%); p<0.001 |
| Exclude missing | 90% (84,96%) | 49% (39,59%) | 41% (30,53%); p<0.001 |
| TMLE for missing data | 90% (84,96%) | 49% (39,59%) | 41% (30,53%); p<0.001 |

The primary analysis conservatively assumed that participants without blood pressure measures were uncontrolled. We conducted two sensitivity analyses to examine the robustness of our missing data approach: (1) exclude participants with missing outcomes, and (2) use TMLE to flexibly adjust for participants with and without blood pressure measures. As shown in the above Table, these sensitivity analyses yielded very similar results for effectiveness on hypertension control at week 24 (primary endpoint) and at week 48 (secondary endpoint).

5.      For the TMLE approach, please clarify the model used to analyse the primary outcome. i.e. did you use a logistic regression (assuming a binomial distribution and logit link) or a linear regression (assuming a normal distribution and identity link), or something else? If using a logistic regression, please clarify how absolute differences were derived from the logistic model including the method to obtain confidence intervals.

We appreciate the Reviewer’s interest in TMLE and refer them to our response to Major Comment #2 above. Briefly, we used the logistic link, which is appropriate for binary outcomes or bounded continuous outcomes. TMLE is a model-robust approach. There is no risk of bias due to misspecification of the working models for the outcome regression or the propensity score.

6.      Please report the covariates included in the final model(s).

For the hypertension control at 24 weeks (primary outcome), TMLE with Adaptive Pre-specification selected adjustment for all candidates: age, sex, baseline hypertension severity, and country. For hypertension control at 48 weeks (secondary outcome), TMLE with Adaptive Pre-specification selected adjustment only for baseline hypertension severity. We have added this to the supplemental materials (Table C).

7.      The paragraph on HIV testing contains repetition with the reasons for declining HIV testing reported twice. Please correct.

Thank you, we have removed the duplicated sentence.

8.      In Table 1, please report denominators (the number of participants with data available). According to the text on Page 10, only 100 participants underwent HIV testing; however, based on the percentages reported in Table 1, the denominator includes all randomised participants. I believe it would be more accurate to report the proportion with HIV only in those who underwent testing unless we can be confident that those not tested were HIV negative. This may apply to other baseline variables with missing data (hence the need to report denominators and potentially adjust percentage calculations).

In Table 1, the only variable subject to missingness was HIV testing. At the Reviewer’s suggestion, we have provided numerator and denomination (%) for this variable. We provide the reason for missing data in the footnote.

New Table 1 Footnote:

*Note, the denominator for all variables in Table 1 is the same as the column total, except for HIV testing where we excluded those who had an unknown HIV status and declined testing.*

9.      When describing baseline characteristics, please comment on the (im)balance in baseline characteristics in the manuscript, mentioning the important imbalance in baseline grade of hypertension. 

At the Reviewer’s suggestion, we now comment on the balance by arm on baseline covariates in the Results and include a discussion of the imbalance and its consequences in the Limitations. Please see response to Major comment 1 above for details on adjustment for baseline imbalance.

New text (Results, paragraph 2):

*Baseline covariates were balanced by arm with the exception of hypertension stage; by chance, a higher proportion of telehealth intervention participants had Grade 1 hypertension (50%) than clinic-based arm participants (37.3%; Table 1).*

New text (Discussion, paragraph 7 – limitations):

*First, by chance there was imbalance hypertension severity at baseline between arms. However, our primary analysis adjusted for baseline hypertension grade as well as other variables, and our results were robust to the analytic approach and sensitivity analyses (Supplementary Tables C-D).*

10.     Please report SBP and DBP continuously in Table 1 i.e. showing means, standard deviations and quartiles.

We have added SBP and DBP means, standard deviations, and quartiles to Table 1. See below for excerpt from Table 1 with this addition.

Excerpt from revised Table 1:

|  | **Number of Participants (%)** | | |
| --- | --- | --- | --- |
|  | **Intervention**  (n=98) | **Control**  (n=102) | **Total**  (n=200) |
| Blood pressure at community-based screening (mmHg) |  |  |  |
| SBP (screening) |  |  |  |
| Mean(SD): | 166 (17) | 168 (17) | 167 (17) |
| Median (Q1,Q3): | 163 (154, 176) | 164 (160, 173) | 164 (159, 174) |
| DBP (screening) |  |  |  |
| Mean(SD): | 101 (10) | 99 (12) | 100 (11) |
| Median (Q1,Q3): | 102 (94, 107) | 100 (92, 108) | 101 (93, 107) |
| Blood pressure at clinic enrollment (mmHg) |  |  |  |
| SBP (enrollment) |  |  |  |
| Mean(SD): | 160 (20) | 162 (19) | 161 (19) |
| Median (Q1,Q3): | 152 (146, 172) | 160 (149, 174) | 157 (147, 173) |
| DBP (enrollment) |  |  |  |
| Mean(SD): | 96 (10) | 96 (13) | 96 (11) |
| Median (Q1,Q3): | 95 (89, 102) | 96 (89, 103) | 96 (89, 102) |

11.     Please clarify how the risk differences and 95% confidence intervals for the primary and secondary outcome results (cf. bottom of page 10 / top of page 11 + Table 2) were obtained. It reads as if these are raw differences (e.g. 77% - 51% = 26%) which is concerning given the important baseline differences in blood pressure between the two arms. As mentioned above in my first major comment, I would strongly recommend that all analyses are adjusted for baseline blood pressure as a continuous variable. This could be done using logistic regression with or without additional baseline covariates.

Please see our Response #2. Briefly, TMLE is model-robust approach and can yield estimates of marginal effects (on any scale and regardless of the link function used). As shown in Supplementary Table X (with accompanying discussion), our results were robust to the approach for covariate adjustment.

12.     Please consider reporting p-values for the primary and secondary outcome results in the text and abstract.

At the Reviewer’s request, we have added p-values.

13.     It would be helpful to see blood pressure reported as continuous outcome at baseline, week 24 and week 48. A figure (e.g. boxplot) would be helpful - this could potentially be added as a second panel to Figure 2 or in the supplement.

Examining effectiveness on mean systolic blood pressure at 24 weeks and 48 weeks was pre-specified in the Statistical Analysis Plan and included in Table 2. At the Reviewer’s request, we have added a new boxplot figure to the supplement to visually display this data (Supplemental materials: Figure A).

## Figure A. Mean SBP across study visits

14.     I would strongly recommend an analysis of covariance for week 24 SBP adjusted for baseline SBP. This could potentially take the form of a longitudinal model if including both week 24 and week 48 measurements. It is not clear whether the SBP results reported in Table 2 are adjusted for baseline SBP (please consider adding a footnote to Table 2).

As previously discussed and detailed in our Statistical Analysis Plan, the primary analysis for week 24 SBP was TMLE with Adaptive Pre-specification. In certain scenarios, TMLE is equivalent to ANCOVA. We refer the Reviewer to Rosenbum and van der Laan, *Int J Biostat*, 2010 for further discussion.

At the Reviewer’s request, we have conducted an additional sensitivity analysis with forced adjustment baseline SBP. As shown in the Table below and as expected, we see very similar results. As shown in response to Major comment 2 above, we have included the adjustment set for all endpoints in the Supplementary Materials.

|  | INTERVENTION | CONTROL | EFFECT |
| --- | --- | --- | --- |
| **At week 24** |  |  |  |
| Primary | 133.4 (129.9,136.8) | 140.9 (136.7,145.0) | -7.5 (-12.9,-2.1); p=0.006 |
| Unadjusted | 133.5 (129.8,137.2) | 140.7 (136.2,145.2) | -7.3 (-13.1,-1.4); p=0.014 |
| Adjustment for SBP | 133.8 (130.1,137.5) | 140.5 (136.1,144.8) | -6.7 (-12.4,-0.9); p=0.023 |
| **At week 48** |  |  |  |
| Primary | 133.2 (130.2,136.3) | 141.5 (138.1,144.9) | -8.2 (-12.8,-3.7); p<0.001 |
| Unadjusted | 133.1 (129.7,136.5) | 141.6 (138.0,145.2) | -8.5 (-13.4,-3.5); p<0.001 |
| Adjustment for SBP | 133.4 (130.1,136.8) | 141.3 (137.9,144.8) | -7.9 (-12.7,-3.1); p=0.001 |

15.     Figure 3. For more clarity, please consider including headers for each subgroup e.g. country, age, gender, etc… and indent the labels (e.g. Kenya and Uganda) within each subgroup header.

We have modified Figure 3 to include subgroup headers as requested. See revised Figure 3 in our response to Reviewer 2 above.

16.     Figure 3. As is recommended practice for subgroup analyses, please report interaction p-values (the p-value associated with the interaction term between the subgroup variable and the treatment group) instead of the p-value of treatment effect within the subgroup.

We appreciate this suggestion. However, we did not conduct our subgroup analyses by running an outcome regression with an interaction between the subgroup variable and the trial arm. With a logistic-link function, this approach would yield estimates of conditional odds ratios – not average treatment effects (i.e., marginal effects on the additive scale). Instead, as detailed in the Statistical Analysis Plan, our subgroup analyses were conducted by repeating the primary analysis (TMLE) within strata defined by the subgroup. Our approach returns marginal effect estimates (regardless of the link function) and is robust to model misspecification. We believe that evaluating the effect within each subgroup provides crucial information about effect heterogeneity. Because accommodating this suggestion would fundamentally change our analytic approach, we respectfully decline to make this change as suggested.

17.     I note that the subgroup analysis is conducted on hypertension control at 48 weeks. Given that the primary outcome is measured at 24 weeks, I believe it would be more logical to perform the subgroup analysis on the week 24 outcome. A subgroup analysis at 48 weeks could potentially be included in the supplemental results; however, I would suggest removing it entirely.

We agree with including sub-group analysis for the primary week 24 outcome in the main manuscript. We also think it is important to show the longer-term effects of the intervention, assessed at 48 weeks. Additionally, we pre-specified conducting subgroup analyses for both endpoints. However, at the Reviewer’s suggestion, we have included subgroup analyses for control at week-24 and week-48 as a two-part Figure (see revised Figure 3 in response to Reviewer 2 above).

18.     Please make sure to use the same legend across all figures. Figures S1 and S2 seem to have the legend reversed (i.e. grey for control in S1 vs grey for intervention in S2).

We have corrected this error.

19.     Please mention imbalances in blood pressure status at baseline as a limitation.

As described in response to major comment #1 and minor comment #9 above, we have added imbalance in baseline blood pressure to the limitations section of the discussion:

New text (Discussion, paragraph 7 – limitations):

*First, by chance there was imbalance hypertension severity at baseline between arms. However, our primary analysis adjusted for baseline hypertension grade as well as other variables, and our results were robust to the analytic approach and sensitivity analyses (Supplementary Tables C-D).*

-Laurent Billot

**Reviewer #4:** This is a small, pilot study of facilitated telehealth hypertension care in rural primary care clinics in Kenya and Uganda. The investigators showed that community health worker facilitated telehealth improved hypertension control.  This is a well-written manuscript and I have minor comments as follows:

1) The time for enrollment was from May to Nov 2022, can the authors include any outcome data of patients that achieved blood pressure control early in the study as a secondary analysis moving forward to understand the direct impact?

We agree that time to hypertension control is important. It is challenging to provide data on early control because this is a function of participants attending a clinical hypertension visit, and we designed our outcome measures to be measured in all participants at specified timepoints regardless of engagement in clinical hypertension care. As shown in the next response, we have generated a Kaplan-Meier curve depicting time to achievement of hypertension control based on blood pressure measurements at clinical hypertension visits. We have now added this Kaplan-Meier curve to the supplemental materials.

2) Can you report mean time to blood pressure control and whether this differed by severity of hypertension at enrollment?

We have generated a Kaplan-Meier plot depicting time to hypertension control, which we have added to the supplemental materials. We used blood pressure measured at clinical hypertension visits (telehealth or clinic visits) to generate this plot. Because the telehealth intervention increased visit adherence and retention in care, there is differential measurement by trial arm, which is ultimately a feature of the telehealth intervention itself. We now reference this plot in the main text by describing the cumulative incidence achieving hypertension control by 90 days and median time to control.

New text (Results, Hypertension care delivery):

Based on blood pressure measures at clinical hypertension visits (telehealth or clinic-based), the median time to hypertension control was similar in both arms (56 days in telehealth intervention arm, 57 days in clinic-based comparator arm). The curves begin to separate by 90 days, with 92% (95% CI 84-96%) of telehealth intervention participants achieving hypertension control by 180 days compared to 70% (95% CI 59-77%) for clinic-based comparator arm participants (S1 Supplement, Figure D).

# Supplemental Figure D. Time to hypertension control (Supplement)

As requested by the reviewer, we also generated a Kaplan-Meier curve depicting time to hypertension control, stratified by hypertension severity at the time of study enrollment. This demonstrates a small decrement in the cumulative incidence of achieving hypertension control as baseline severity of hypertension increases, though not statistically significant.

3) Can you also report number of medications needed to achieve blood pressure control by severity of hypertension at enrollment?

We provide a table below demonstrating the number and type of medications prescribed to persons who had controlled blood pressure at week 24, stratified by baseline hypertension severity at the time of study enrollment. As seen in this table, the number of medications prescribed to those who achieved hypertension control at week 24 (assessed cross-sectionally among all participants by research assistants) was similar by trial arm. As expected, those with more severe baseline hypertension required a greater number of medications to achieve hypertension control. We have added this as an additional supplemental table.

# Table E. Number of medications prescribed as of week 24 among those with controlled blood pressure at week 24, stratified by baseline hypertension severity and trial arm

|  | **Telehealth intervention arm** | | | **Clinic-based arm** | | |
| --- | --- | --- | --- | --- | --- | --- |
| **Hypertension severity at study enrollment** | **BP 140-159/90-99 (N=40)** | **BP 160-179/100-109 (N=19)** | **BP ≥180/110 (N=16)** | **BP 140-159/90-99 (N=20)** | **BP 160-179/100-109 (N=22)** | **BP ≥180/110 (N=11)** |
| **Number of medications** |  |  |  |  |  |  |
| Mean (SD) | 1.30 (0.72) | 1.68 (0.58) | 2.06 (0.44) | 1.40 (0.68) | 1.91 (0.61) | 2.09 (0.70) |
| Median [Min, Max] | 1.00 [0, 3] | 2.00 [1, 3] | 2.00 [1, 3] | 1.50 [0, 2] | 2.00 [1, 3] | 2.00 [1, 3] |
| **Number of medications** |  |  |  |  |  |  |
| 0 | 5 (12.5%) | 0 (0%) | 0 (0%) | 2 (10.0%) | 0 (0%) | 0 (0%) |
| 1 | 19 (47.5%) | 7 (36.8%) | 1 (6.3%) | 8 (40.0%) | 5 (22.7%) | 2 (18.2%) |
| 2 | 15 (37.5%) | 11 (57.9%) | 13 (81.3%) | 10 (50.0%) | 14 (63.6%) | 6 (54.5%) |
| 3 | 1 (2.5%) | 1 (5.3%) | 2 (12.5%) | 0 (0%) | 3 (13.6%) | 3 (27.3%) |
| **Calcium channel blocker** | 28 (70.0%) | 16 (84.2%) | 14 (87.5%) | 14 (70.0%) | 21 (95.5%) | 11 (100%) |
| **Thiazide diuretic** | 7 (17.5%) | 5 (26.3%) | 7 (43.8%) | 4 (20.0%) | 5 (22.7%) | 2 (18.2%) |
| **ACE/ARB** | 4 (10.0%) | 3 (15.8%) | 5 (31.3%) | 4 (20.0%) | 3 (13.6%) | 4 (36.4%) |

4) All the patients living with HIV were treated and virally suppressed.  Did you notice any differences in ability to achieve hypertension control among persons living with HIV compared with persons without HIV such as longer time to achieve control or more medications needed to achieve control?

We note that we have included HIV status in a pre-specified sub-group analysis of our primary outcome. The telehealth intervention improved hypertension control for both people with and without HIV, though with wide confidence intervals that were not statistically significant among people with HIV (n=27).

Due to the small number of persons with HIV in our study, it is difficult to make definitive conclusions regarding time to hypertension control or the number of medications required to achieve hypertension control in these exploratory analyses. We did not observe any difference in time to hypertension control by HIV status, as shown in the below Kaplan-Meier curve generated for the reviewer. We note that people with HIV had a slightly higher average number of medications required to achieve hypertension control.

**Number of medications prescribed as of week 24 among those with controlled blood pressure at week 24, stratified by HIV status and trial arm**

|  | **Intervention** | | **Control** | |
| --- | --- | --- | --- | --- |
|  | **no known HIV (N=68)** | **HIV (N=7)** | **no known HIV (N=46)** | **HIV (N=7)** |
| **htnmednum_24** |  |  |  |  |
| Mean (SD) | 1.51 (0.702) | 2.00 (0.577) | 1.74 (0.713) | 1.86 (0.690) |
| Median [Min, Max] | 2.00 [0, 3.00] | 2.00 [1.00, 3.00] | 2.00 [0, 3.00] | 2.00 [1.00, 3.00] |
| **htnmednumfct_24** |  |  |  |  |
| 0 | 5 (7.4%) | 0 (0%) | 2 (4.3%) | 0 (0%) |
| 1 | 26 (38.2%) | 1 (14.3%) | 13 (28.3%) | 2 (28.6%) |
| 2 | 34 (50.0%) | 5 (71.4%) | 26 (56.5%) | 4 (57.1%) |
| 3 | 3 (4.4%) | 1 (14.3%) | 5 (10.9%) | 1 (14.3%) |
| **Calcium channel blocker** | 53 (77.9%) | 5 (71.4%) | 41 (89.1%) | 5 (71.4%) |
| **Thiazide diuretic** | 17 (25.0%) | 2 (28.6%) | 8 (17.4%) | 3 (42.9%) |
| **ACE/ARB** | 9 (13.2%) | 3 (42.9%) | 8 (17.4%) | 3 (42.9%) |

5) Related to the above, were there any differences among participants with multiple co-morbidities versus not in achieving hypertension control?

In response to both reviewer 2 and 4, we have added an additional sub-group of participants with any co-morbidity (hypertension plus HIV, diabetes, or chronic kidney disease) to our sub-group analysis of the primary trial outcome, as well as our secondary outcome of hypertension control at week 48. See revised Figure 3 and Supplemental Figures B-C as described above.

The reviewer also asked this question related to above questions regarding time to hypertension control. We therefore also generated a Kaplan-Meier curve to explore differences in achievement of hypertension control, noting no differences in time to hypertension control by those with or without a comorbidity (HIV, diabetes, or chronic kidney disease). We have generated this and other Kaplan-Meier curves for the reviewer to address these excellent exploratory questions, but have not added these additional Kaplan-Meier curves to the manuscript to avoid addition of multiple post-hoc analyses.

**Reviewer #5:** The authors have produced a clear and well written manuscript describing the results of an RCT comparing fully in person HTN care to a combination of telehealth facilitated by CHWs and less frequent clinic follow-up. The results are impressive for the pilot and can inform potential options for managing individuals most at risk for complications. There were however some areas which were confusing to this reviewer and which need clarification. In addition, while this is indeed a promising model, there were significant resources needed including engaging existing CHWs, telehealth through study clinicians as examples and addressing how this can be scaled will be important

We thank the reviewer for their positive comments and agree with the importance of understanding resource utilization and scalability of our telehealth approach. As described above in response to the Editor, the present study was designed as an initial pilot study to evaluate preliminary effectiveness of a CHW-facilitated telehealth intervention to determine the utility of larger studies on effectiveness of scale. We are currently conducting a larger cluster randomized trial to test the telehealth intervention at scale and have designed an additional implementation-focused hybrid-effectiveness study focused on testing the effectiveness of implementation strategies for imbedding and normalizing the telehealth intervention within routine practice in Ministry of Health primary care clinics. We have added to the limitations of this pilot study that we are and reference to subsequent ongoing or planned studies to further understand this important issue.

New text (Discussion, paragraph 7 – Limitations, new text in italics):

*First, though fully powered for our primary outcome, this was a pilot study with a relatively small sample size which limits inferences about effectiveness at scale.* However, by conducting the study at three government primary care clinics that are broadly representative of primary health centers in rural Kenya and Uganda, our findings are likely generalizable to similar rural primary health care settings. We are currently conducting a follow-up population-level study to understand intervention effects at scale and when integrated with population-level screening for HIV and HIV risk (NCT05768763). *Future studies will also need to evaluate cost-effectiveness of additional resource inputs including CHW stipends, medication costs, and clinician time to deliver telehealth visits.*

Methods:
The intervention is described as leveraging HIV care, and integrated, but the study seems to be done at PHC centers? A description of where and how HIV care is delivered and where the HTN was done

Thank you for the opportunity to clarify. In rural health facilities in Kenya and Uganda, HIV care is imbedded within primary health centers. Our hypertension telehealth intervention did not include HIV care, thus people with HIV had to go to the clinic separately to receive HIV care. For stable patients this generally meant attending HIV clinic every 3-6 months. In the clinic-based (control) arm, participants received both HIV and hypertension care during the same visit to the clinic, if due to be seen for both conditions. More than half of people with HIV accessed HIV care at clinics outside their community (where the study was not operating) – these participants did not receive integrated hypertension and HIV care.

New text (Methods; new text in *italics*):

*Telehealth intervention arm*

*We were not able to deliver HIV care through the telehealth model, so participants with co-occurring HIV continued to receive HIV care at the local MoH primary health center or other clinic of their choice.*

*Clinic-based comparator arm*

*In rural health facilities in Kenya and Uganda, HIV care is imbedded within primary health centers, thus participants with co-occurring HIV were able to receive HIV care from the same clinic*.

It is not explicitly described the expectation for regular visits for the control arm (assume every month?)

Hypertension visits were scheduled with the same frequency in both intervention and control arms- every 4 weeks if hypertension was uncontrolled and every 12 weeks if hypertension was uncontrolled. Clinicians were free to adjust this schedule in both arms, based on clinical need (e.g. visits might be scheduled for 2 weeks in the future for a patient with hypertensive urgency). We include this in the procedures section of the methods and have now added additional clarification to indicate that this applies to both telehealth (intervention) and clinic (control/comparator) arms. See also response to reviewer 3 above.

Revised text (Methods, Study enrollment and hypertension care delivery):

*In both telehealth and clinic-based arms, follow-up visits were scheduled every 4 weeks if hypertension was uncontrolled and every 12 weeks if hypertension was controlled (defined as blood pressure <140/90 mmHg, based on country guidelines).*^6,7^ *Visits could be by telehealth or in-person for the telehealth arm, with telehealth being the default unless the clinician determined that an in-person visit was needed. All clinic arm visits were scheduled in the clinic.*

Were medications provided free to both arms or are these available free of charge regardless

Yes, medications were provided free of charge to participants in both arms. See response to reviewer 1 above for additional detail. Revised text is copied below for reference.

New text (Methods, Study enrollment and hypertension care delivery):

*In primary health centers in Kenya and Uganda, hypertension medications are typically available for a small fee (~$1 US dollar per one-month supply). For the study, all medications were provided free of charge to study participants in both telehealth and clinic arms. In the event of medication stock-outs, the study provided additional medication supply to ensure uninterrupted access to medications.*

Were stockouts an issue for the clinic-based care (versus the packaged weekly medications for CHW delivery)

The study provided buffer stocks of medications to ensure there were no stock-outs. Medications were equally available for both intervention and control arms. We have added this to the methods – see prior response and response to reviewer 1 above.

Was the clinical visit for retention in care including a CHW one or only at the clinic? If the latter, how was this adjusted based on the frequency of expected clinic visits? Measuring adherence to visits comparing a CHW-facilitated telemedicine home visit versus a clinic visit should be made more clear in the text. Was there any outreach for no-shows in the control arm?

We defined retention in care using either a CHW home visit for telehealth or an in-person clinic visit. We have now clarified this in the methods.

Revised text (Methods, Outcomes, new text in *italics*):

Retention in care was defined as not late for the most recent scheduled hypertension clinical visit *(either telehealth or in-clinic)* by 30 days or more.

Results
A consort diagram to help a reader understand differences in acceptability and accessibility -how did the 80% who came to clinic and those enrolled differed from those with HTN dxed.

The consort diagram can be viewed in Figure 1 in the original manuscript. To further explore differences between the 80% who linked vs the 20% who did not link, we have added a new supplemental table depicting characteristics of the 266 persons who were eligible for the study, stratified by linkage to care following initial community-based screening. As seen in this table, individuals who did not link had similar blood pressures but tended to be younger and less likely to have a known history of hypertension compared to those who linked to clinic (and were evaluated for enrollment).

**Table B. Characteristics of persons with severe hypertension identified on community screening stratified by linkage vs non-linkage to the community primary care clinic**

|  | **Not linked (N=52)** | **Linked (N=214)** |  |
| --- | --- | --- | --- |
| **Age (years)** |  |  |  |
| Mean (SD) | 56 (15) | 62 (13) |  |
| Median (Q1, Q3) | 51 (43, 67) | 62 (51, 72) |  |
| **Sex** |  |  |  |
| Female | 35 (67.3%) | 149 (69.6%) |  |
| Male | 17 (32.7%) | 65 (30.4%) |  |
| **Blood Pressure Ever Measured** |  |  |  |
| Yes | 23 (44.2%) | 138 (64.5%) |  |
| No | 29 (55.8%) | 76 (35.5%) |  |
| **Hypertension History** |  |  |  |
| Yes | 13 (25.0%) | 93 (43.5%) |  |
| No | 39 (75.0%) | 121 (56.5%) |  |
| **Hypertension Meds (Last 30 Days)** |  |  |  |
| Yes | 9 (17.3%) | 43 (20.1%) |  |
| No | 4 (7.7%) | 50 (23.4%) |  |
| Missing | 39 (75.0%) | 121 (56.5%) |  |
| **Diabetes Diagnosis** |  |  |  |
| Yes | 5 (9.6%) | 21 (9.8%) |  |
| No | 47 (90.4%) | 193 (90.2%) |  |
| **HIV Status*** |  |  |  |
| Yes | 2 (3.8%) | 22 (10.3%) |  |
| No | 21 (40.4%) | 102 (47.7%) |  |
| Missing | 29 (55.8%) | 90 (42.1%) |  |
| **Average Systolic BP (mmHg)** |  |  |  |
| Mean (SD) | 160 (19) | 166 (17) |  |
| Median (Q1, Q3) | 161 (146, 166) | 164 (158, 174) |  |
| **Average Diastolic BP (mmHg)** |  |  |  |
| Mean (SD) | 103 (12) | 100 (11) |  |
| Median (Q1, Q3) | 102 (96, 108) | 100 (92, 107) |  |
| **Hypertension Stage** |  |  |  |
| BP <140/90 mmHg | 0 (0%) | 0 (0%) |  |
| BP 140-159/90-99 mmHg | 0 (0%) | 2 (0.9%)† |  |
| BP 160-179/100-109 mmHg | 35 (67.3%) | 150 (70.1%) |  |
| BP ≥180/110 mmHg | 17 (32.7%) | 62 (29.0%) |  |

* Self-reported HIV status to community health worker. HIV testing was conducted after linkage to the clinic and is not depicted in this table.

† Two participants had mild hypertension at community-based screening but self-presented to the clinic and had blood pressure ≥160/100 mmHg on repeat measurement at the clinic and were therefore eligible for the study.

The close to half with previous diagnosis is an important statistic. Were differences in effectiveness seen based on previous diagnosis (or exploratory for baseline medications?)

Please see our response to Reviewer 2 and updated Figure 3 which now includes analysis of week 24 and 48 hypertension control by strata of previous diagnosis and baseline hypertension medications (reproduced below)

The section on reasons for not HIV testing, results are repeated

Thank you. We have removed this duplication.

Medication results are ? missing (and would like to know quality of care-were both arms on recommended medications?

Thank you for this important point. We have expanded our medication reporting in several ways to address this and prior reviewer comments. 1) New supplemental table reporting mean/median number of medications prescribed at last visit and the proportion on each class of medication (reproduced below and added to supplement), 2) new supplemental table reporting the mean/median number of medications required to achieve hypertension control at 24 weeks stratified by baseline hypertension severity (see response to reviewer 4 above), 3) new supplemental table providing comprehensive tabulation of medications and dosing at most recent attended visit at end of study (see response to the Editor above).

From these tables, it is apparent that medication prescribing was similar between telehealth intervention and control. As noted above in response to the Editor, these reflect the last prescribed medications and not necessarily adherence to these medications or consistent engagement in care.

# Table F. Number and type of hypertension medications prescribed over 48 weeks*

|  | **Intervention (N=98)** | **Control (N=102)** | **Overall (N=200)** |
| --- | --- | --- | --- |
| **Number of medications*** |  |  |  |
| Mean (SD) | 1.6 (0.8) | 1.7 (0.7) | 1.6 (0.7) |
| Median (Q1, Q3) | 2 (1, 2) | 2 (1, 2) | 2 (1, 2) |
| **Number of medications** |  |  |  |
| 0 | 9 (9.2%) | 7 (6.9%) | 16 (8.0%) |
| 1 | 30 (30.6%) | 25 (24.5%) | 55 (27.5%) |
| 2 | 51 (52.0%) | 63 (61.8%) | 114 (57.0%) |
| 3 | 8 (8.2%) | 7 (6.9%) | 15 (7.5%) |
| **Calcium channel blocker†** | 73 (74.5%) | 79 (77.5%) | 152 (76.0%) |
| **Thiazide diuretic†** | 30 (30.6%) | 31 (30.4%) | 61 (30.5%) |
| **ACE/ARB†** | 18 (18.4%) | 17 (16.7%) | 35 (17.5%) |

* maximum number of drugs prescribed at any clinical hypertension visit (either telehealth or in-clinic)

† proportion prescribed each drug class at any given visit over 48-week study

Discussion
The discussion is well laid out but again has some areas where clarification is needed. The paragraph about integrating HIV and HTN care requires more explanation-was this HTN integrated into HIV care? Did people without HIV got to an HIV clinic? Since the main outcome was status neutral, this terminology is confusing unless you have Hiv 9-) people coming to an HIV/HTN integrated clinic? See comments above

Thank you for these clarifications. We have revised this paragraph to focus on implications of our findings for people with co-occurring HIV and hypertension. See also response to Reviewer 2.

As described in response above, HIV care is integrated into primary care clinics in rural Kenya and Uganda where this study took place – therefore all patients received hypertension care with or without HIV care from the same clinic.

New Text (Discussion, paragraph 5):

*For people with HIV, integration of HIV and hypertension care is an important way to improve cardiovascular disease prevention.^44,45^ However, in our clinic-based arm where HIV and hypertension care were integrated, retention in hypertension care among people with HIV was only 53% at week 48, compared to 100% retention in the telehealth arm. Several factors likely explain low retention in hypertension care among people with HIV in our study. First, moderate-severe hypertension requires a different level of intensive monitoring until stabilized, often monthly visits until blood pressure is controlled. In contrast, HIV visits are often every 3-6 months for stable patients. Second, only 44% (n=12/27) of study participants with HIV and hypertension accessed HIV care at their local community primary care clinic (where the study was based). We did not assess reasons for receiving HIV care at a different clinic outside of the community, though reasons may include stigma, travel/mobility, or other preference-related factors.* At the end of the study, 74% of people with HIV *expressed a preference for receiving* at least some hypertension visits at home *through CHW-facilitated telehealth*, highlighting the role this intervention could play in a patent-centered approach to hypertension care *delivery even among people with HIV already accessing the clinic for their HIV care.*

The limitations should include the potential for the study clinicians to influence the effectiveness (although in theory should be equal in intervention and control). See above other areas where clarity is made (supply chain support, ?medication costs), leveraging an exisiting CHW system.

We have added potential for unblinded study clinicians to influence effectiveness, though agree with the reviewer that this is unlikely to have influenced trial outcomes. Clinicians were trained to provide high quality care to all participants and we saw no clinically meaningful differences in the number or type of medications that were prescribed.

New text (Discussion, paragraph 7):

*Finally, intervention allocation was not blinded to staff delivering the interventions (CHWs, clinicians) or research assistants assessing outcomes, which could have introduced bias. However, our primary outcome of blood pressure control was assessed using automated blood pressure machines, minimizing the potential for bias in outcome assessment. Study clinicians delivering hypertension care could have differentially influenced quality of care by trial arm, though we saw no differences in the number of drugs prescribed and clinicians were trained to provide high-quality care to both trial arms.*

Minor

Methods: the sentence "WithinTMLE, we used Adaptive Pre-specification to select the optimal adjustment approach via sample-splitting; candidate adjustment variables were age, sex, baseline hypertension severity, country, and unadjusted." Seems incomplete?

Thank you. This language is correct as ‘unadjusted’ is a potential candidate for the final model selected by TMLE. However, to enhance clarity, we have removed the word unadjusted to avoid reader confusion. We have also provided a new Supplementary Table (with discussion) to provide further information about our primary analytic approach and the robustness of our findings (Supplementary Table C).

**General editorial requests:**
(Note: not all will apply to your paper, but please check each item carefully)

*       We ask every co-author listed on the manuscript to fill in a contributing author statement, making sure to declare all competing interests. If any of the co-authors have not filled in the statement, we will remind them to do so when the paper is revised. If all statements are not completed in a timely fashion this could hold up the re-review process. If new competing interests are declared later in the revision process, this may also hold up the submission. Should there be a problem getting one of your co-authors to fill in a statement we will be in contact. Please do not add or remove authors without first discussing this with the handling editor. You can see our competing interests policy here: [https://urldefense.com/v3/__http://journals.plos.org/plosmedicine/s/competing-interests__;!!LQC6Cpwp!vBXN6i9HZtJyXzbXEZa2BgyovL-jGT5Bs8PzeQ0FfnS3PPWcxvhKYmzp9-LU94s4851gj3VihwrBv_ZtsVKY$](https://urldefense.com/v3/__http:/journals.plos.org/plosmedicine/s/competing-interests__;!!LQC6Cpwp!vBXN6i9HZtJyXzbXEZa2BgyovL-jGT5Bs8PzeQ0FfnS3PPWcxvhKYmzp9-LU94s4851gj3VihwrBv_ZtsVKY$) .

I will remind co-authors to complete the competing interest statement. Please send reminders as indicated above.

*       Please upload any figures associated with your paper as individual TIF or EPS files with 300dpi resolution at resubmission; please read our figure guidelines for more information on our requirements: [https://urldefense.com/v3/__http://journals.plos.org/plosmedicine/s/figures__;!!LQC6Cpwp!vBXN6i9HZtJyXzbXEZa2BgyovL-jGT5Bs8PzeQ0FfnS3PPWcxvhKYmzp9-LU94s4851gj3VihwrBv9od0LGv$](https://urldefense.com/v3/__http:/journals.plos.org/plosmedicine/s/figures__;!!LQC6Cpwp!vBXN6i9HZtJyXzbXEZa2BgyovL-jGT5Bs8PzeQ0FfnS3PPWcxvhKYmzp9-LU94s4851gj3VihwrBv9od0LGv$) . While revising your submission, please upload your figure files to the PACE digital diagnostic tool, [https://urldefense.com/v3/__https://pacev2.apexcovantage.com/__;!!LQC6Cpwp!vBXN6i9HZtJyXzbXEZa2BgyovL-jGT5Bs8PzeQ0FfnS3PPWcxvhKYmzp9-LU94s4851gj3VihwrBv7-jmaIa$](https://urldefense.com/v3/__https:/pacev2.apexcovantage.com/__;!!LQC6Cpwp!vBXN6i9HZtJyXzbXEZa2BgyovL-jGT5Bs8PzeQ0FfnS3PPWcxvhKYmzp9-LU94s4851gj3VihwrBv7-jmaIa$) . PACE helps ensure that figures meet PLOS requirements. To use PACE, you must first register as a user. Then, login and navigate to the UPLOAD tab, where you will find detailed instructions on how to use the tool. If you encounter any issues or have any questions when using PACE, please email us at [PLOSMedicine@plos.org](mailto:PLOSMedicine@plos.org).

We have uploaded all figures as EPS.

*       Please ensure that the paper adheres to the PLOS Data Availability Policy (see [https://urldefense.com/v3/__http://journals.plos.org/plosmedicine/s/data-availability__;!!LQC6Cpwp!vBXN6i9HZtJyXzbXEZa2BgyovL-jGT5Bs8PzeQ0FfnS3PPWcxvhKYmzp9-LU94s4851gj3VihwrBv-oHdthg$](https://urldefense.com/v3/__http:/journals.plos.org/plosmedicine/s/data-availability__;!!LQC6Cpwp!vBXN6i9HZtJyXzbXEZa2BgyovL-jGT5Bs8PzeQ0FfnS3PPWcxvhKYmzp9-LU94s4851gj3VihwrBv-oHdthg$) ), which requires that all data underlying the study's findings be provided in a repository or as Supporting Information. For data residing with a third party, authors are required to provide instructions with contact information (web or email address) for obtaining the data. Please note that a study author cannot be the contact person for the data. PLOS journals do not allow statements supported by "data not shown" or "unpublished results." For such statements, authors must provide supporting data or cite public sources that include it.

We have revised our data availability statement as follows:

All relevant data are within the manuscript and its Supporting Information files.

*       We expect all researchers with submissions to PLOS in which author-generated code underpins the findings in the manuscript to make all author-generated code available without restrictions upon publication of the work. In cases where code is central to the manuscript, we may require the code to be made available as a condition of publication. Authors are responsible for ensuring that the code is reusable and well documented. Please make any custom code available, either as part of your data deposition or as a supplementary file. Please add a sentence to your data availability statement regarding any code used in the study, e.g. "The code used in the analysis is available from Github [URL] and archived in Zenodo [DOI link]" Please review our guidelines at [https://urldefense.com/v3/__https://journals.plos.org/plosmedicine/s/materials-software-and-code-sharing__;!!LQC6Cpwp!vBXN6i9HZtJyXzbXEZa2BgyovL-jGT5Bs8PzeQ0FfnS3PPWcxvhKYmzp9-LU94s4851gj3VihwrBv3j9MwQO$](https://urldefense.com/v3/__https:/journals.plos.org/plosmedicine/s/materials-software-and-code-sharing__;!!LQC6Cpwp!vBXN6i9HZtJyXzbXEZa2BgyovL-jGT5Bs8PzeQ0FfnS3PPWcxvhKYmzp9-LU94s4851gj3VihwrBv3j9MwQO$)  and ensure that your code is shared in a way that follows best practice and facilitates reproducibility and reuse. Because Github depositions can
be readily changed or deleted, we encourage you to make a permanent DOI'd copy (e.g. in Zenodo) and provide the URL.

We have revised our data availability statement as follows:

The code used in the analysis is available from Github https://github.com/LauraBalzer/search_severe_htn

*       ETHICS STATEMENTS: Please provide details of the consent and whether it was written or oral. Please also provide the approval number.

As specified in our methods section (paragraph 4), “Participants completed written informed consent prior to enrollment.” We have revised our ethics statement to indicate IRB approval numbers as follows:

We obtained ethical approval from the Makerere University School of Medicine Research and Ethics Committee in Uganda (2020-029); the Kenya Medical Research Institute in Kenya (P00151/4173); and the University of California San Francisco in the United States of America (20-32144).

*       FINANCIAL DISCLOSURES: The funding statement should include: specific grant numbers, initials of authors who received each award, URLs to sponsors’ websites. Also, please state whether any sponsors or funders (other than the named authors) played any role in study design, data collection and analysis, the decision to publish, or preparation of the manuscript. If they had no role in the research, include this sentence: “The funders had no role in study design, data collection and analysis, decision to publish, or preparation of the manuscript.”

We have already included grant numbers. We modified our prior statement to reflect this exact wording:

Old text: The funder of the study had no role in study design, data collection, data analysis, data interpretation, or writing of the report.

New text: The funders had no role in study design, data collection and analysis, decision to publish, or preparation of the manuscript

*       COMPETING INTERESTS: All authors must declare their relevant competing interests per the PLOS policy, which can be seen here: [https://urldefense.com/v3/__https://journals.plos.org/plosmedicine/s/competing-interests__;!!LQC6Cpwp!vBXN6i9HZtJyXzbXEZa2BgyovL-jGT5Bs8PzeQ0FfnS3PPWcxvhKYmzp9-LU94s4851gj3VihwrBv3gieOsF$](https://urldefense.com/v3/__https:/journals.plos.org/plosmedicine/s/competing-interests__;!!LQC6Cpwp!vBXN6i9HZtJyXzbXEZa2BgyovL-jGT5Bs8PzeQ0FfnS3PPWcxvhKYmzp9-LU94s4851gj3VihwrBv3gieOsF$)
For authors with ties to industry, please indicate whether any of the interests has a financial stake in the results of the current study.

We have no competing interests to declare. As above, all authors will complete the statement.

*       DATA AVAILABILITY: PLOS Medicine requires that the de-identified data underlying the specific results in a published article be made available, without restrictions on access, in a public repository or as Supporting Information at the time of article publication, provided it is legal and ethical to do so. Please see the policy at [https://urldefense.com/v3/__http://journals.plos.org/plosmedicine/s/data-availability__;!!LQC6Cpwp!vBXN6i9HZtJyXzbXEZa2BgyovL-jGT5Bs8PzeQ0FfnS3PPWcxvhKYmzp9-LU94s4851gj3VihwrBv-oHdthg$](https://urldefense.com/v3/__http:/journals.plos.org/plosmedicine/s/data-availability__;!!LQC6Cpwp!vBXN6i9HZtJyXzbXEZa2BgyovL-jGT5Bs8PzeQ0FfnS3PPWcxvhKYmzp9-LU94s4851gj3VihwrBv-oHdthg$)  
and FAQs at [https://urldefense.com/v3/__http://journals.plos.org/plosmedicine/s/data-availability*loc-faqs-for-data-policy__;Iw!!LQC6Cpwp!vBXN6i9HZtJyXzbXEZa2BgyovL-jGT5Bs8PzeQ0FfnS3PPWcxvhKYmzp9-LU94s4851gj3VihwrBv-VAcmT5$](https://urldefense.com/v3/__http:/journals.plos.org/plosmedicine/s/data-availability*loc-faqs-for-data-policy__;Iw!!LQC6Cpwp!vBXN6i9HZtJyXzbXEZa2BgyovL-jGT5Bs8PzeQ0FfnS3PPWcxvhKYmzp9-LU94s4851gj3VihwrBv-VAcmT5$)  

The Data Availability Statement (DAS) requires revision. For each data source used in your study: 
a) If the data are freely or publicly available, note this and state the location of the data: within the paper, in Supporting Information files, or in a public repository (include the DOI or accession number).
b) If the data are owned by a third party but freely available upon request, please note this and state the owner of the data set and contact information for data requests (web or email address). Note that a study author cannot be the contact person for the data.
c) If the data are not freely available, please describe briefly the ethical, legal, or contractual restriction that prevents you from sharing it. Please also include an appropriate contact (web or email address) for inquiries (again, this cannot be a study author).

See above for revision to our data availability statement, indicating we have made our data available in the supporting information files.

FORMATTING - GENERAL
*       Abstract: Please structure your abstract using the PLOS Medicine headings (Background, Methods and Findings, Conclusions). Please combine the Methods and Findings sections into one section.

We have made this change.

*       At this stage, we ask that you include a short, non-technical Author Summary of your research to make findings accessible to a wide audience that includes both scientists and non-scientists. The Author Summary should immediately follow the Abstract in your revised manuscript. This text is subject to editorial change and should be distinct from the scientific abstract. Ideally each sub-heading should contain 2-3 single sentence, concise bullet points containing the most salient points from your study. In the final bullet point of 'What Do These Findings Mean?', please include the main limitations of the study in non-technical language. Please see our author guidelines for more information: [https://urldefense.com/v3/__https://journals.plos.org/plosmedicine/s/revising-your-manuscript*loc-author-summary__;Iw!!LQC6Cpwp!vBXN6i9HZtJyXzbXEZa2BgyovL-jGT5Bs8PzeQ0FfnS3PPWcxvhKYmzp9-LU94s4851gj3VihwrBv3hSY3jC$](https://urldefense.com/v3/__https:/journals.plos.org/plosmedicine/s/revising-your-manuscript*loc-author-summary__;Iw!!LQC6Cpwp!vBXN6i9HZtJyXzbXEZa2BgyovL-jGT5Bs8PzeQ0FfnS3PPWcxvhKYmzp9-LU94s4851gj3VihwrBv3hSY3jC$) .

We have included the author summary

*       Please express the main results with 95% CIs as well as p values. When reporting p values please report as p<0.001 and where higher as the exact p value p=0.002, for example. Throughout, suggest reporting statistical information as follows to improve clarity for the reader "22% (95% CI [13%,28%]; p</=)". Please be sure to define all numerical values at first use.

We have added p-values to the text as requested.

*       Please include page numbers and line numbers in the manuscript file. Use continuous line numbers (do not restart the numbering on each page).

We have added line numbers. Page numbers were already included.

*       Please cite the reference numbers in square brackets. Citations should precede punctuation.

We have modified citations as requested. We have moved the bracket citations before the punctuation in the clean manuscript version with flattened citations.

FIGURES AND TABLES
*       Please provide titles and legends for all figures and tables (including those in Supporting Information files).

We have included titles and legends for all figures and tables

*       Please define all abbreviations used in each figure/table (including those in Supporting Information files).

We have now defined abbreviations in all figures and tables.

*       Please consider avoiding the use of red and green in order to make your figure more accessible to those with color blindness.

We have changed Figure 2 to a monochromatic color scale to ensure accessibility for those with red-green colorblindness.

SUPPLEMENTARY MATERIAL
*       Please note that supplementary material will be posted as supplied by the authors. Therefore, please amend it according to the relevant comments outlined here.

We have reviewed the supplement for compliance with these guidelines.

*       Please cite your Supporting Information as outlined here: [https://urldefense.com/v3/__https://journals.plos.org/plosmedicine/s/supporting-information__;!!LQC6Cpwp!vBXN6i9HZtJyXzbXEZa2BgyovL-jGT5Bs8PzeQ0FfnS3PPWcxvhKYmzp9-LU94s4851gj3VihwrBv9i5UPpc$](https://urldefense.com/v3/__https:/journals.plos.org/plosmedicine/s/supporting-information__;!!LQC6Cpwp!vBXN6i9HZtJyXzbXEZa2BgyovL-jGT5Bs8PzeQ0FfnS3PPWcxvhKYmzp9-LU94s4851gj3VihwrBv9i5UPpc$)

We have revised our Supporting information citations to comply with these requirements.

REFERENCES
*       PLOS uses the numbered citation (citation-sequence) method and first six authors, et al.

We have revised our references to follow the PLOS Medicine style, using the style downloaded from Zotero.

*       Please ensure that journal name abbreviations match those found in the National Center for Biotechnology Information (NCBI) databases ([https://urldefense.com/v3/__http://www.ncbi.nlm.nih.gov/nlmcatalog/journals__;!!LQC6Cpwp!vBXN6i9HZtJyXzbXEZa2BgyovL-jGT5Bs8PzeQ0FfnS3PPWcxvhKYmzp9-LU94s4851gj3VihwrBv7oxTAJ-$](https://urldefense.com/v3/__http:/www.ncbi.nlm.nih.gov/nlmcatalog/journals__;!!LQC6Cpwp!vBXN6i9HZtJyXzbXEZa2BgyovL-jGT5Bs8PzeQ0FfnS3PPWcxvhKYmzp9-LU94s4851gj3VihwrBv7oxTAJ-$) ), and are appropriately formatted and capitalised.

See above

*       Where website addresses are cited, please include the complete URL and specify the date of access (e.g. [accessed: 12/06/2024]).

See above

*       Please also see [https://urldefense.com/v3/__https://journals.plos.org/plosmedicine/s/submission-guidelines*loc-references__;Iw!!LQC6Cpwp!vBXN6i9HZtJyXzbXEZa2BgyovL-jGT5Bs8PzeQ0FfnS3PPWcxvhKYmzp9-LU94s4851gj3VihwrBv9vHVF5A$](https://urldefense.com/v3/__https:/journals.plos.org/plosmedicine/s/submission-guidelines*loc-references__;Iw!!LQC6Cpwp!vBXN6i9HZtJyXzbXEZa2BgyovL-jGT5Bs8PzeQ0FfnS3PPWcxvhKYmzp9-LU94s4851gj3VihwrBv9vHVF5A$)  for further details on reference formatting.

See above

STUDY TYPE-SPECIFIC REQUESTS

*       PLOS Medicine requires that all trials be prospectively registered in one of registries recognized by WHO. Please ensure that study registration details are included in the Methods section.

We have included the clinicaltrials.gov registration in the methods.

*       Please structure the Methods section using the following sub-headings: Study design and participants, Randomization and masking, Procedures, Outcomes, Statistical analysis.

We have revised the methods to use these sub-headings.

*       The following outcomes measures time to attaining hypertension control (secondary outcome), hypertension control at 48 weeks, moderate-to-severe hypertension (average BP ≥160/100 mmHg) at 24 and 48 weeks, mean systolic blood pressure at 24 and 48 weeks, structured survey at 24 and 48 weeks appear to differ between the submitted manuscript and the protocol. Please clarify and explain all discrepancies between the paper and protocol. If the outcomes were not prespecified in the protocol, please define them in the Methods (Outcomes section) as post hoc and explain why they were added. Post-hoc comparisons should be presented as hypothesis generating rather than conclusive.

We have included our pre-specified analysis plan, which outlines all pre-specified analyses including those mentioned. Where relevant, we have indicated when analyses were post hoc and the reasons for this (e.g. peer reviewer requested).

*       Please ensure that all prespecified outcomes (primary, secondary, and exploratory) are listed in the Methods/Outcomes section and indicate whether there are outcomes that are not presented in the current report.

We have presented all pre-specified primary and secondary outcomes in this manuscript.

*       Please specify the dates (Month Day, Year) during which study enrollment and follow up occurred.

We have added these dates to the manuscript: Screening took place between May 12-November 1, 2022). Week 24 endpoint visits took place from October 27, 2022 to April 21, 2023. Week 48 endpoint visits took place between April 13 – October 16, 2023.

*       Please include absolute numbers wherever you report percentages; eg, n/N (%)

We have revised our manuscript to ensure that absolute numbers are included with percentages.

*       Please present the safety data for the study including numbers of specific events and whether or not adverse events are thought to be related to treatment. AEs should be reported in the abstract, per CONSORT and CONSORT-Harms.

We already included this information in the Results section on adverse events. We have now added safety data to the abstract as follows:

Three participants died (telehealth: 2, clinic: 1); all deaths were unrelated to the study interventions.

*       Please complete the CONSORT checklist ([https://urldefense.com/v3/__https://www.equator-network.org/reporting-guidelines/consort/__;!!LQC6Cpwp!vBXN6i9HZtJyXzbXEZa2BgyovL-jGT5Bs8PzeQ0FfnS3PPWcxvhKYmzp9-LU94s4851gj3VihwrBv8dNFfHp$](https://urldefense.com/v3/__https:/www.equator-network.org/reporting-guidelines/consort/__;!!LQC6Cpwp!vBXN6i9HZtJyXzbXEZa2BgyovL-jGT5Bs8PzeQ0FfnS3PPWcxvhKYmzp9-LU94s4851gj3VihwrBv8dNFfHp$) ) and ensure that all components of CONSORT are present in the manuscript, including how randomization was performed, allocation concealment, blinding of intervention, definition of lost to follow-up, power statement. When completing the checklist, please use section and paragraph numbers, rather than page numbers.

We confirm all CONSORT elements are in the manuscript. We have revised the CONSORT checklist to include section headings and paragraph numbers as requested.

*       Please report your abstract according to CONSORT for abstracts, following the PLOS Medicine abstract structure (Background, Methods and Findings, Conclusions) [https://urldefense.com/v3/__https://www.equator-network.org/reporting-guidelines/consort-abstracts/__;!!LQC6Cpwp!vBXN6i9HZtJyXzbXEZa2BgyovL-jGT5Bs8PzeQ0FfnS3PPWcxvhKYmzp9-LU94s4851gj3VihwrBv9qU_n7K$](https://urldefense.com/v3/__https:/www.equator-network.org/reporting-guidelines/consort-abstracts/__;!!LQC6Cpwp!vBXN6i9HZtJyXzbXEZa2BgyovL-jGT5Bs8PzeQ0FfnS3PPWcxvhKYmzp9-LU94s4851gj3VihwrBv9qU_n7K$)

We have modified our abstract accordingly.

*       If your trial had to undergo important modifications in response to extenuating circumstances, please complete the CONSERVE-CONSORT checklist and provide in your Supporting Information; ([https://urldefense.com/v3/__https://www.equator-network.org/reporting-guidelines/guidelines-for-reporting-trial-protocols-and-completed-trials-modified-due-to-the-covid-19-pandemic-and-other-extenuating-circumstances-the-conserve-2021-statement/__;!!LQC6Cpwp!vBXN6i9HZtJyXzbXEZa2BgyovL-jGT5Bs8PzeQ0FfnS3PPWcxvhKYmzp9-LU94s4851gj3VihwrBvx4GNp71$](https://urldefense.com/v3/__https:/www.equator-network.org/reporting-guidelines/guidelines-for-reporting-trial-protocols-and-completed-trials-modified-due-to-the-covid-19-pandemic-and-other-extenuating-circumstances-the-conserve-2021-statement/__;!!LQC6Cpwp!vBXN6i9HZtJyXzbXEZa2BgyovL-jGT5Bs8PzeQ0FfnS3PPWcxvhKYmzp9-LU94s4851gj3VihwrBvx4GNp71$) ). When completing the checklist, please use section and paragraph numbers, rather than page numbers.

Not applicable

*       In keeping with our commitment to Open Science, please include the study protocol document and analysis plan (including any amendments) as Supporting Information to be published with the manuscript if accepted.

We have included the Protocol Synopsis (as requested by the Editor) and the Statistical Analysis Plan as Supporting Information.

*       Please note that PLOS Medicine requires prospective, public registration of a data sharing plan (as part of mandatory clinical trials registration) for all clinical trials that began enrollment on or after January 1, 2019, in accordance with ICMJE requirements.

This is noted. As indicated above, our trial was registered on clinicaltrials.gov (*NCT04810650).*
