# Supplementary material for: Community health worker–facilitated telehealth for moderate–severe hypertension care in Kenya and Uganda: A randomized controlled trial
Source: PLoS Med. 2025 Jun 5;22(6):e1004632. doi: 10.1371/journal.pmed.1004632 (PMC12165344; doi:10.1371/journal.pmed.1004632)
Supplement: Attachment — Submitted filename: Response to reviewers v2.docx [file pmed.1004632.s012.docx]

------------------------------------------------------------
Comments from Reviewers:

Reviewer #3: Thank you for thoroughly addressing my previous comments. I feel re-assured that the baseline differences in hypertension grade were adequately accounted for and that the results are robust to adjustments. It is also reassuring to see that, when reported continuously, baseline differences in SBP and DBP are in fact minimal. I have no further comment.
-Laurent Billot

Reviewer #5: The authors have done a thorough and masterful response to the many reviewers and as a result, the paper is ready for acceptance.

We thank all of the reviewers for their time and for their thoughtful comments and suggestions to improve our manuscript.

------------------------------------------------------------
Requests from Editors:

GENERAL

* Please confirm that your title complies with to PLOS Medicine's style. Your title must be nondeclarative and not a question. It should begin with main concept if possible. "Effect of" should be used only if causality can be inferred, i.e., for an RCT. Please place the study design ("A randomized controlled trial," "A retrospective study," "A modelling study," etc.) in the subtitle (ie, after a colon).

We have revised the title as follows to meet PLOS Medicine’s style: Community Health Worker Facilitated Telehealth for Moderate-Severe Hypertension Care in Kenya and Uganda: A randomized controlled trial

* Please ensure that all abbreviations are defined at first use throughout the text (including statistical abbreviations). Please also check figures and tables.

We have reviewed our manuscript text, figures, and tables for abbreviations to ensure all are defined.

* Please review your text for claims of novelty or primacy (e.g. 'for the first time', ‘novel’) and remove this language.

We confirm there are no inappropriate claims of novelty or primacy. In our introduction, we specifically discuss how our work builds on prior related work in the field.

* Please check that any use of statistical terms (such as trend or significant) are supported by the data, and if not please remove them.

We have reviewed the manuscript and confirm that we have not used any unsupported statistical terms to describe our results.

* Please ensure that tables and figures, including those in supplementary files, are appropriately referenced in the main text.

We confirm that all tables and figures, including supplementary files are referenced in the main text. We have now added reference S1 Supplement Figure A in the main text. This figure was generated at the request of one of the Reviewers, but we had not yet added reference in the manuscript.

* Statistical reporting: Please revise throughout the manuscript, including tables and figures.
a) Please report statistical information as follows to improve clarity for the reader "22% (95% CI [13%,28%]; p</=)". 
b) Please separate upper and lower bounds with commas instead of hyphens as the latter can be confused with reporting of negative values. 
c) Please define statistical definitions at first use and repeat the abbreviated definitions (HR, CI etc.) for each set of parentheses.

We have made these changes as requested.

* Thank you for providing a protocol summary. Please note that we still require you to provide the full protocol as a Supporting Information file.

We have re-attached the full protocol as a supporting information file, in addition to the protocol summary. We have added a reference to the full protocol in the main text (line 163).

ABSTRACT

* Please confirm that your abstract complies with our requirements, including providing all the information relevant to this study type [https://urldefense.com/v3/__https://journals.plos.org/plosmedicine/s/submission-guidelines*loc-abstract__;Iw!!LQC6Cpwp!pYWcivDoEdVORkOz0uYI8nppXyuqtXElytE9lQ2U_bZ89CyNULtrqXhBZoPB1ghQujTk-U2Y-H5M4qL0bkgc$](https://urldefense.com/v3/__https:/journals.plos.org/plosmedicine/s/submission-guidelines*loc-abstract__;Iw!!LQC6Cpwp!pYWcivDoEdVORkOz0uYI8nppXyuqtXElytE9lQ2U_bZ89CyNULtrqXhBZoPB1ghQujTk-U2Y-H5M4qL0bkgc$)   

We have reviewed the journal’s requirements and confirm that our abstract is in compliance.

* Per CONSORT, please note that only the primary outcome of the trial should be reported in your Abstract. Secondary outcomes should only be included in the Abstract if all secondary outcomes are fully reported. For trials that have many secondary outcomes, the Abstract should be limited to reporting the primary outcome.

We have removed the additional outcomes of severe hypertension and retention in care from the abstract.

* Please ensure that all numbers presented in the abstract are present and identical to numbers presented in the main manuscript text.

We confirm that numbers presented in the abstract and main text are consistent.

* In the last sentence of the Abstract Methods and Findings section, please describe the main limitation(s) of the study's methodology.

We have added the following limitation to the abstract:

Our study was limited by its small sample size, though findings are strengthened by being conducted in three primary health centers across two countries.

* In the abstract, please include the important dependent variables that are adjusted for in the analyses.

In our pre-specified analysis (see S4 Statistical Analysis Plan), we estimated marginal effects with targeted minimum loss-based estimation (TMLE) to maximize empirical efficiency through a data-adaptive, yet, fully automated procedure for covariate adjustment. In other words, our approach to covariate adjustment was pre-specified, but not fixed. Details of this approach are beyond the scope of the abstract but are explained in detail in the methods and in S1 Supplement Table C which we added at the prior Reviewers’ request. We have revised our abstract as follows:

In intention-to-treat analyses, we compared outcomes between randomized arms with targeted minimum loss-based estimation (TMLE) using sample-splitting to select optimal adjustment covariates (candidates: age, sex, baseline hypertension severity, and country).

* When reporting age, please ensure to include a unit such as years. Also, please repeat units, e.g. for BP (e.g. l.36, 37).

We have made this change.

* Please specify who was blinded to the intervention and control.

We added the statement “Randomization assignment was not blinded, except for the study statistician.”

* Please define the intervention and control states.

We have defined trial arms in the ‘Methods and Findings’ section as follows: “telehealth by a clinician (adherence assessment, counseling, decision-making) and facilitated by a CHW in the participant’s home, compared to clinic-based hypertension care”

* Please state that analysis was intention to treat.

We have added this (line 39).

* Please provide the number of participants lost to follow up in each group.

We have added this information as follows: “Week 24 blood pressure was measured in 96/99 intervention and 99/102 control participants.” Additional detail on reasons for non-measurement (death, loss to follow-up) can be found in the consort figure (Figure 1)

AUTHOR SUMMARY

* In the final bullet point of 'What Do These Findings Mean?', please include the main limitations of the study in non-technical language.

We have added an additional bullet point as follows:

- The main study limitation of our study is that it was a small pilot study with relatively few patients. This means that results might not apply to all settings or populations. To make sure our findings are as relevant as possible, we conducted the study in three different government-run clinics located in two countries.

METHODS AND RESULTS 

* In the flow diagram, please indicate the number of individuals in each group analyzed in the ITT analysis.

In our primary ITT analysis, we included all randomized participants and assumed that those with missing blood pressure measurements had uncontrolled hypertension. We also conducted several sensitivity analyses (S1 Supplement Table D, S4 Statistical Analysis Plan). We have added an additional row in the relevant boxes of the flow diagram to indicate that all randomized participants were analyzed in the ITT analysis.

* Causal language - In trials, there is usually a distinction in the language in terms of causal vs associational for primary and secondary trial outcomes. It would be beneficial to use associational language in the discussion and other sections for secondary outcomes.

Thank you. We have reviewed the manuscript to ensure we are not overstating causal language in this randomized trial and believe we are incompliance with this request.

* The terms gender and sex are not interchangeable (as discussed in [https://urldefense.com/v3/__https://www.who.int/health-topics/gender*tab=tab_1__;Iw!!LQC6Cpwp!pYWcivDoEdVORkOz0uYI8nppXyuqtXElytE9lQ2U_bZ89CyNULtrqXhBZoPB1ghQujTk-U2Y-H5M4k2IzQ8t$](https://urldefense.com/v3/__https:/www.who.int/health-topics/gender*tab=tab_1__;Iw!!LQC6Cpwp!pYWcivDoEdVORkOz0uYI8nppXyuqtXElytE9lQ2U_bZ89CyNULtrqXhBZoPB1ghQujTk-U2Y-H5M4k2IzQ8t$)  ); please use the appropriate term.

Thank you for this important point. We have revised to ensure we consistently refer to sex throughout. We have revised the text of Figure 3 to use the heading ‘Sex’ to indicate stratified analysis by sex, rather than gender as we previously listed.

* Figure 2: Please convert any stacked bar charts to another data representation for example a table, or other type of graph. Please ensure to provide the nominators and denominators for each group per time point.

We have added an additional supplementary table that contains the underlying data for Figure 2 and reference this table in the figure caption (S1 Supplement Table K). We believe Figure 2 provides a helpful visualization of how blood pressure categories changed over time and also agree that it is important to provide the data in a table.

* ll.426-429: Could you please clarify where, i.e. in a table or figure, these results can be found?

We did not include a table of these results, but provided them in the main text to provide additional context for intervention mechanisms among this important sub-population of people living with HIV. If the Editor prefers that these be available in a figure, we can add an additional supplemental figure that includes the full forest plot of pre-specified sub-groups in which we analyzed our retention outcomes.

* Supplement Figures E-F: Does Clinic Factor equate to Quality of Care? If so, we think Quality of Care in Clinic would be a better description for the figures than Clinic Factor. We also suggest using the same description between the figures and the main text.

Thank you for this important point. This domain concerned quality of care received (regardless of whether in clinic or by telehealth). We have relabeled this domain as “Quality of Clinical Care”.

* l.453: Is there a specific reason why you chose to show the numbers from 180 days instead of, say, 200 days, which is on the x-axis?

This was a post-hoc analysis requested by the reviewers and there is no specific reason why we chose 180 days. To be more consistent with timing of our study outcome assessment, we have changed the text to report the proportion attaining hypertension control by 24 weeks and revised the figure to use weeks on the x-axis.

* ll.458-464: Please ensure to provide a reference to the relevant table(s) and/or figure(s).

These data are provided in the main text only. In this paragraph, we provide additional details on implementation outcomes.

* ll.570-472: Could you please clarify where, i.e. in a table or figure, these results can be found?

These data are provided in the main text only. In this paragraph, we provide additional details on expressed preferences of study participants during endpoint surveys.

DISCUSSION

* Please remove the 'conclusions' subheading.

We have made this change.

------------------------------------------------------------
General Editorial Requests

1) We ask every co-author listed on the manuscript to fill in a contributing author statement. If any of the co-authors have not filled in the statement, we will remind them to do so when the paper is revised. If all statements are not completed in a timely fashion this could hold up the re-review process. Should there be a problem getting one of your co-authors to fill in a statement we will be in contact. YOU MUST NOT ADD OR REMOVE AUTHORS UNLESS YOU HAVE ALERTED THE EDITOR HANDLING THE MANUSCRIPT TO THE CHANGE AND THEY SPECIFICALLY HAVE AGREED TO IT.

Thank you. Please let us know if you are unable to get signed forms by any co-authors and we will provide assistance.

2) Please ensure that the paper adheres to the PLOS Data Availability Policy (see [https://urldefense.com/v3/__http://journals.plos.org/plosmedicine/s/data-availability__;!!LQC6Cpwp!pYWcivDoEdVORkOz0uYI8nppXyuqtXElytE9lQ2U_bZ89CyNULtrqXhBZoPB1ghQujTk-U2Y-H5M4hod_vrV$](https://urldefense.com/v3/__http:/journals.plos.org/plosmedicine/s/data-availability__;!!LQC6Cpwp!pYWcivDoEdVORkOz0uYI8nppXyuqtXElytE9lQ2U_bZ89CyNULtrqXhBZoPB1ghQujTk-U2Y-H5M4hod_vrV$) ), which requires that all data underlying the study's findings be provided in a repository or as Supporting Information. For data residing with a third party, authors are required to provide instructions with contact information for obtaining the data. PLOS journals do not allow statements supported by "data not shown" or "unpublished results." For such statements, authors must provide supporting data or cite public sources that include it.

We confirm that we meet PLOS Data Availability Policy. We have included the full de-identified dataset with the supplemental materials.

3) Please review your reference list to ensure that it is complete and correct. If you have cited papers that have been retracted, please include the rationale for doing so in the manuscript text, or remove these references and replace them with relevant current references. Any changes to the reference list should be mentioned in the rebuttal letter that accompanies your revised manuscript.

We have reviewed our reference list. To our knowledge, none of the manuscripts we cite have been retracted.
